# Sustainable Joullié–Ugi and Continuous Flow Implementation Led to Novel Captopril-Inspired Broad-Spectrum Metallo-β-Lactamase Inhibitors

**DOI:** 10.1021/acs.jmedchem.5c00750

**Published:** 2025-08-15

**Authors:** Antonella Ilenia Alfano, Sveva Pelliccia, Simona Barone, Luigi Cutarella, Sacha Michèle Idriss Cancade, Valerio Baia, Emilia Cassese, Pasquale Russomanno, Nicolò Messano, Denia Frank, Lilia Weizel, Marco J. Rotter, Steffen Brunst, Thomas A. Wichelhaus, Ewgenij Proschak, Daniele Tedesco, Mattia Mori, Jean Denis Docquier, Vincenzo Summa, Margherita Brindisi

**Affiliations:** † Department of Pharmacy (Department of Excellence 2023-2027), 9307 University of Naples Federico II, Via D. Montesano 49, 80131 Naples, Italy; ‡ Department of Biotechnology, Chemistry and Pharmacy, 9313 University of Siena, Via Aldo Moro 2, 53100 Siena, Italy; § Department of Medical Biotechnologies, University of Siena, Viale Bracci 16, 53100 Siena, Italy; ∥ Magnetic Resonance Centre (CERM), Consorzio Interuniversitario Risonanze Magnetiche di Metallo Proteine (CIRMMP) and Department of Chemistry “Ugo Schiff”, 9300 University of Florence, Via L. Sacconi 6, 50019 Sesto Fiorentino, Italy; ⊥ 9173 Goethe University Frankfurt, University Hospital, Institute of Medical Microbiology and Infection Control, Paul-Ehrlich-Str. 40, 60596 Frankfurt am Main, Germany; # Institute of Pharmaceutical Chemistry, Goethe-University of Frankfurt, Max-von-Laue Str. 9, D-60438 Frankfurt am Main, Germany; ∇ Institute for Organic Synthesis and Photoreactivity (ISOF), National Research Council of Italy (CNR), Via P. Gobetti 101, 40129 Bologna, Italy

## Abstract

Metallo-β-lactamases (MBL) production is one of the most alarming bacterial resistance mechanisms, conferring broad-spectrum resistance to most β-lactam antibiotics and combinations with β-lactamase inhibitors. Since no MBL inhibitors have been approved yet, the quest for novel, safe, and effective compounds, possibly endowed with broad-spectrum activity against clinically relevant MBLs, represents an urgent clinical need. Inspired by captopril, which behaves as a weak MBL inhibitor, we herein report a continuous flow protocol for the generation of new MBL inhibitors. We employed a Joullié–Ugi multicomponent reaction for generating two indoline-based subseries, reproducing the captopril binding mode, while increasing the hydrophobic interactions within the MBL active site. Interaction between inhibitors and five clinically relevant MBL isoforms (NDM-1, VIM-1, VIM-2, IMP-1, and IMP-7) was investigated by biochemical methods and rationalized through docking studies. Furthermore, the activity in clinical isolates in synergy with β-lactam antibiotics was assessed, thus paving the way to a further optimization campaign.

## Graphical abstract

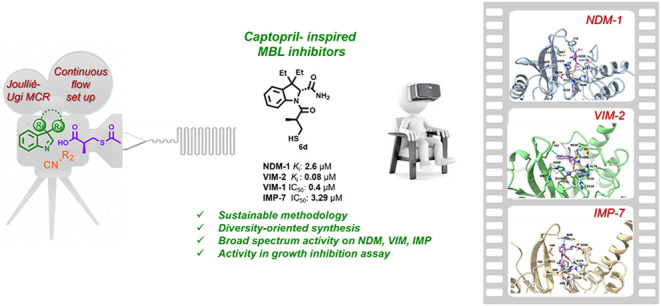

## 1 Introduction

Antimicrobial resistance (AMR) can be seen as a slow pandemic posing a huge threat to public health worldwide. β-Lactam antibiotics, including penicillins, cephalosporins, and more importantly the life-saving carbapenems, are the cornerstones of antimicrobial chemotherapy and still represent extremely valuable antibacterial drugs, although their efficacy is steadily declining.^1^ Several clinically relevant Gram-negative (diderm) pathogens (carbapenem-resistant *Klebsiella pneumoniae*, *Acinetobacter baumannii*, *Pseudomonas aeruginosa*, and *Enterobacter spp*.)—included among the acronymically dubbed ‘ESKAPE pathogens’’—have become highly resistant to the vast majority of available antibacterial drugs and are able to dodge the biocidal action of antibiotics.^2–6^ Regrettably, the situation is not expected to improve since current estimates predict that AMR, already accounting for over 1.2 million deaths globally, could represent the main cause of death in 2050 (10 million deaths per year), threatening the outcome of even the simplest medical procedure.^7–9^ The COVID-19 pandemic further aggravated this scenario, since COVID-19 patients are routinely treated with broad-spectrum antibiotics, including expanded-spectrum cephalosporins (e.g., ceftriaxone, ceftazidime, and cefepime), quinolones, and carbapenems.^10^ Notably, infections with antibiotic-resistant *Staphylococcus aureus*, *K. pneumoniae*, *P. aeruginosa*, or *A. baumannii* have been reported in patients with COVID-19 in intensive care units. The advent of mobilized colistin resistance-1 in 2015,^11^ and transferable tigecycline resistance genes in 2019,^12^ which mediate resistance to colistin and tigecycline, respectively, means that the efficacy of all clinically vital antibiotics for serious Gram-negative infections is compromised, with cefiderocol being a last-resort backup antibiotic. Regrettably, these circumstances create a so-called perfect storm for an accelerated evolution of antimicrobial resistance.^13^

Resistance to carbapenems among Gram-negative bacteria is primarily due to the production of one or more carbapenemase(s), which inactivate these life-saving drugs. These enzymes are frequently encoded by plasmids that are easily transferred among strains. Bacteria achieve these challenging chemical reactions with two families of hydrolytic enzymes: serine β-lactamases (SBLs) using a catalytic conserved serine residue and metallo-β-lactamases (MBLs) using one or two zinc ions for catalysis. The increased prevalence of serine-carbapenemases and MBLs makes β-lactam antibiotics increasingly ineffective for the treatment of infections caused by Multidrug-resistant/extensively drug-resistant (MDR/XDR) Gram-negative isolates.^14^

MBLs are of particular interest and concern given several factors: (i) their ability to hydrolyze and provide resistance to virtually all β-lactam antibiotics (except monobactams); (ii) the unavailability of clinically useful MBL inhibitors; (iii) the rapid pace at which new variants are isolated; (iv) the transferability of their encoding genes, and (v) their ubiquity, since they have been identified in both nosocomial and environmental strains.^15,16^

MBLs belong to three subclasses (B1, B2, and B3), based primarily on their metal content and different active site features. The β-lactamase genes encoding subclass B1MBL are largely plasmid-borne and are of greater clinical relevance compared with those of subclass B2 and B3 enzymes. Imipenemase (IMP), Verona Integron-encoded Metallo-carbapenemase (VIM), and New Delhi Metallo-β-lactamase (NDM) subtypes are the three most common MBLs found in clinical isolates and belong to subclass B1.^17^ Notably, NDM-type enzymes are currently the predominant MBLs in Europe.^18^ NDM-producing microorganisms can cause life-threatening infections; therefore, the fact that these microorganisms are actively disseminating outside the healthcare system is a matter of concern.

Eight compounds have been approved over the years as SBL inhibitors to be used in different combinations with antibiotics, with enmetazobactam and durlobactam being the last addition to the armamentarium.^19,20^ However, none of these inhibitors show activity against the MBL-producing strains. Combinations with MBL inhibitors are currently unavailable for clinical use; therefore, the development of broad-spectrum MBL inhibitors able to restore the efficacy of existing antibiotics represents an extremely urgent medical need.^21–24^

Although diverse chemical templates have been lately proposed as MBL inhibitors, poor sequence similarity among various members, selectivity issues toward human metalloenzymes, and the presence of shallow active sites pose a relevant hurdle to the development of safe and effective broad-spectrum MBL inhibitors.^25–29^ In this context, it is worth mentioning taniborbactam, a pan-spectrum bicyclic boronate inhibitor, developed in combination with cefepime, which completed phase 3 clinical trials in 2022,^30^ and xeruborbactam (QPX7728), an orally available inhibitor featuring a similar scaffold which completed phase 1 clinical trials in combination with the β-lactam QPX2014.^31^

As per established drug discovery practice, besides *de novo* design of novel chemical entities behaving as MBL inhibitors, approved drugs were also engaged for their inhibitory potential toward these metalloenzymes. Accordingly, as shown in Figure 1, l-captopril (**1**) and d-captopril (**2**) were unveiled as NDM-1 inhibitors, with reported IC_50_ = 202 μM and 21.8 μM, and *K_i_* = 39 and 1.3 μM, respectively.^32–34^ A cocrystal structure of **1** in complex with NDM-1 was solved (PDB: 4EXS), thus allowing us to determine the binding mode and key interaction within the enzyme active site. More recent evidence also demonstrated that other thiol-based approved drugs, namely, thiorphan (**3**), dimercaprol (**4**), and tiopronin (**5**), behave as micromolar MBL inhibitors in their racemic forms, with cocrystallization in NDM-1 confirming a binding mode superimposable to that displayed by captopril; however, none of these compounds display broad-spectrum inhibitory profile and relevant synergistic activity when assessed in combination with β-lactam antibiotics.^35–37^

**Figure 1:**
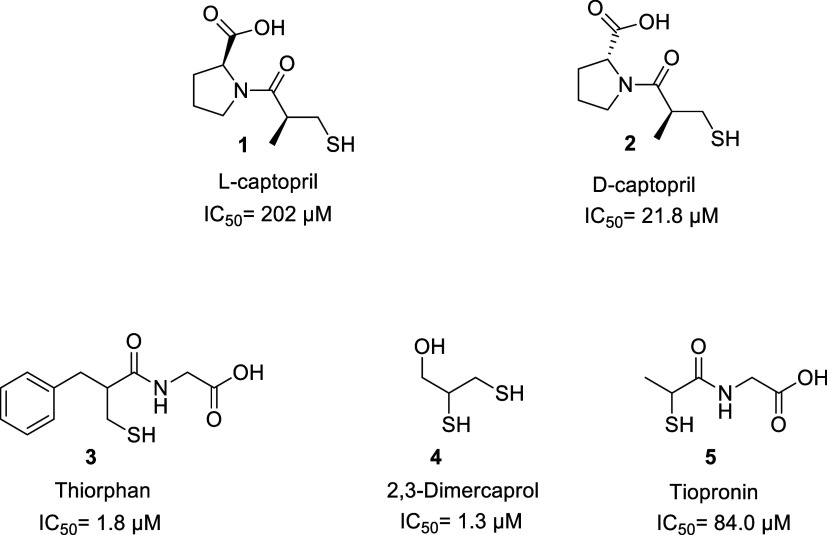
Structures of l- and d-captopril (compounds **1** and **2**, respectively) and representative thiol-based MBL inhibitors (**3–5**).

The broad-spectrum potential of captopril on other MBLs belonging to B1 class was proved by further crystal structures, including VIM-2 (PDB: 4C1D) and IMP-1 (PDB: 4C1F), although residues interacting with the carboxylic group differ based on the respective MBL and the absolute configuration of the inhibitors carboxylic group.^37^

Although a large number of thiol-based compounds have been disclosed lately, showing moderate to good *in vitro* inhibitory activity, most of them lacked broad-spectrum activity or were assessed only on a minor number of MBL isoforms. Furthermore, promising inhibitors failed or were not evaluated against clinically relevant isolates.

The aim of this study was the exploration of captopril-inspired, thiol-based MBL inhibitors. In particular, we employed a multicomponent reaction protocol in order to rapidly provide, in a one-pot fashion, indoline-based compounds rationally designed to reproduce the captopril binding mode in the MBL active site, while also increasing the hydrophobic interaction pattern. To get further insights into compounds’ interaction, we also explored different stereochemical outcomes at the 2-position of the indoline core (compounds **6a**–**f**, Table 1). We also obtained a small subset of compounds with a merged inhibitor design, i.e., with a thiorphan fragment embedded in our newly conceived indoline-based structural framework (compounds **7a**–**d**, Table 1).

**Table 1:**
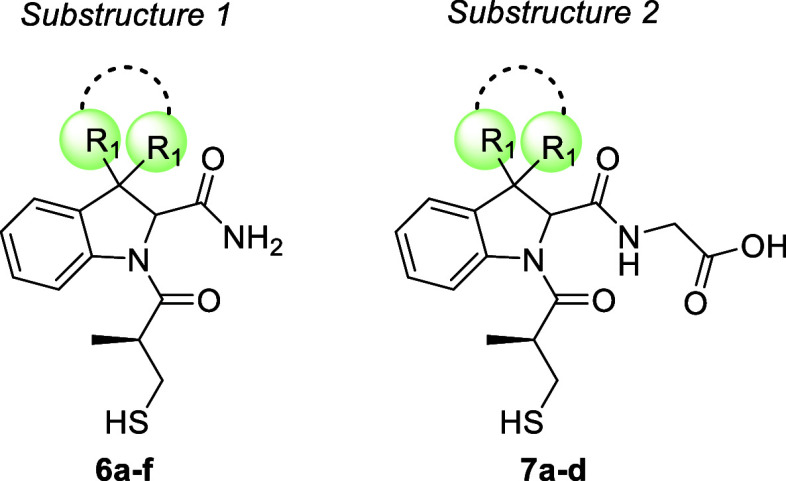

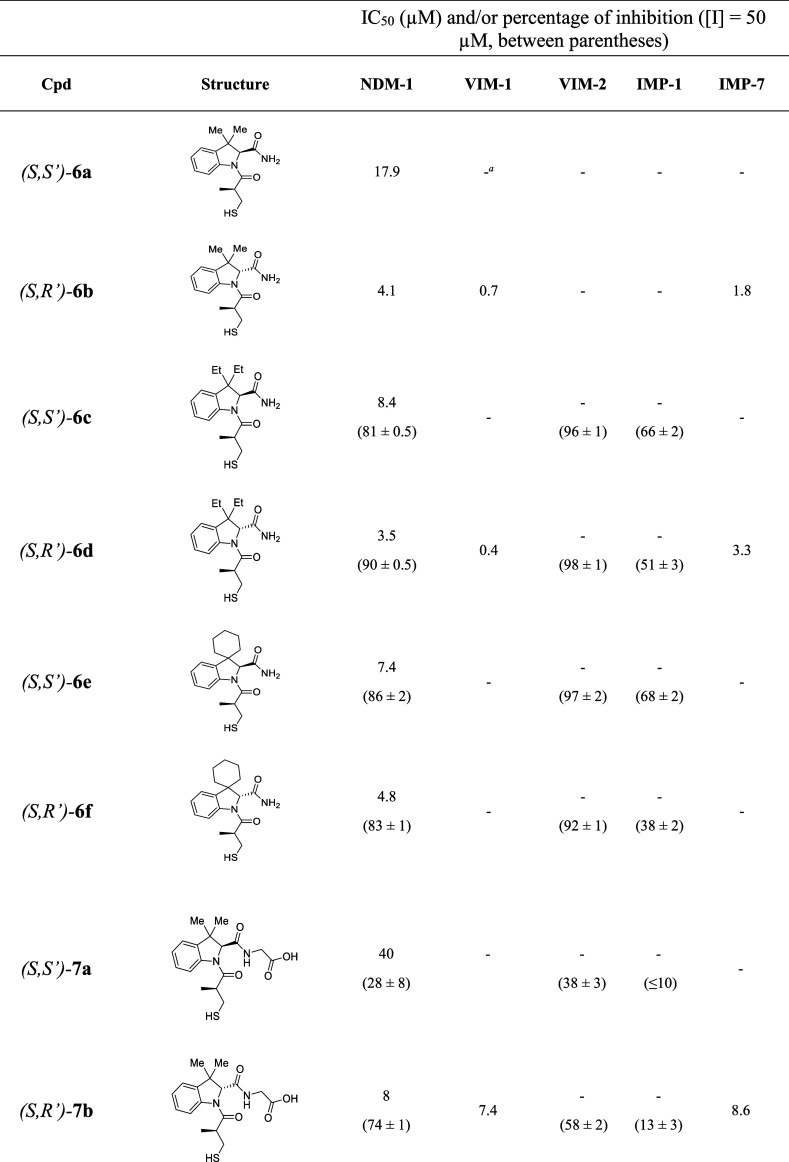

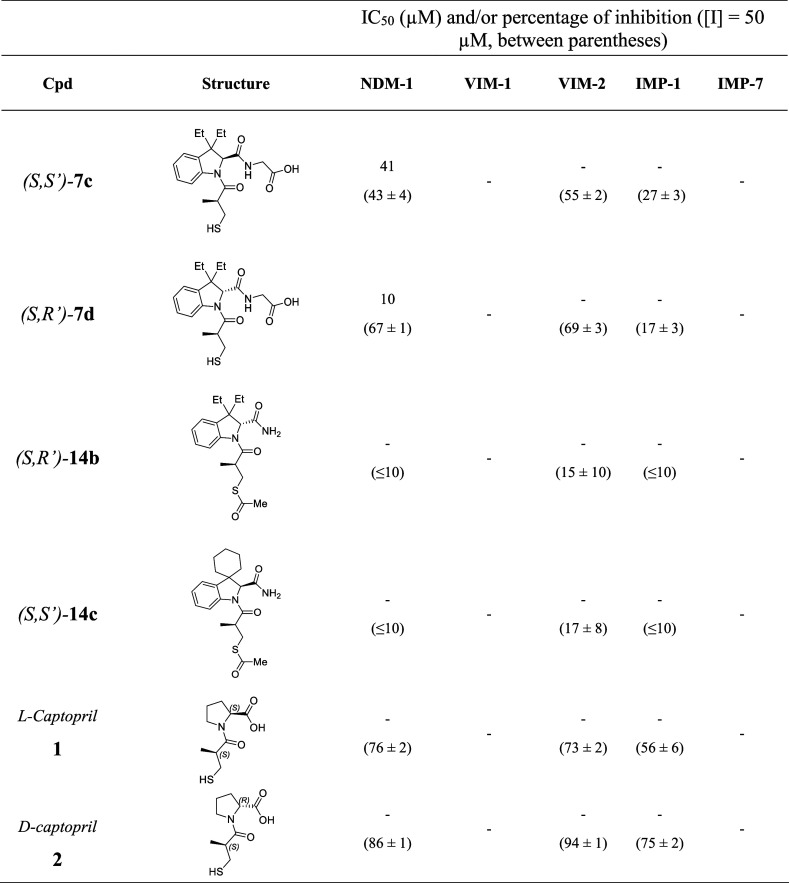
Structures and Inhibitory Activities of Captopril-Inspired Derivatives **6a**–**f**, **7a**–**d** and Thioester Derivatives **14b,c** against MBLs

a -, Not determined.

Furthermore, the overall efficiency and sustainability of our conceived chemical procedure were further increased by the implementation of a telescoped continuous flow protocol for the synthesis of the compounds’ main core. Finally, in order to validate our approach and the potential usefulness of these novel structural motifs, the inhibitory activity of these original compounds on relevant MBLs (NDM-1, VIM-1, VIM-2, IMP-1, and IMP-7) was investigated by biochemical methods, while their binding mode was rationalized through docking studies. Their synergistic activity with carbapenems was also assessed on MBL-producing clinical isolates.

## 2 Results and Discussion

### 2.1 Rational Design

As mentioned in the previous paragraph, the general objective of our work was represented by the rational design and synthesis of derivatives inspired by the drug captopril as potential broad-spectrum inhibitors of MBL. Captopril demonstrated activity in the micromolar range toward various MBL isoforms. Our work focused on the synthesis of heterocyclic derivatives in order to (i) obtain compounds with an improved inhibitory activity profile against MBLs compared to captopril, while maintaining a broad-spectrum profile; (ii) morph captopril structure such as to minimize the presence of functional groups related to its original pharmacological activity (inhibition of ACE-1, angiotensin-converting enzyme) and interaction with similar human targets; (iii) implement diversity-oriented synthetic protocols giving easy access to future extensive SAR exploration; (iv) employ sustainable and advantageous continuous flow chemical methodologies.

The rational design of the molecules first focused on the NDM-1 enzyme, since the activity of both isomers of captopril is well described on this peculiar MBL. The designed molecules, besides maintaining the thiol portion essential for the interaction with the zinc cations in the enzyme active site, were also designed to maximize interactions in the active site pocket, with particular reference to hydrogen bonds and hydrophobic interactions.

The general scheme of the rational design of the new NDM-1 inhibitors and general structures 1 and 2 is shown in Figure 2.

**Figure 2:**
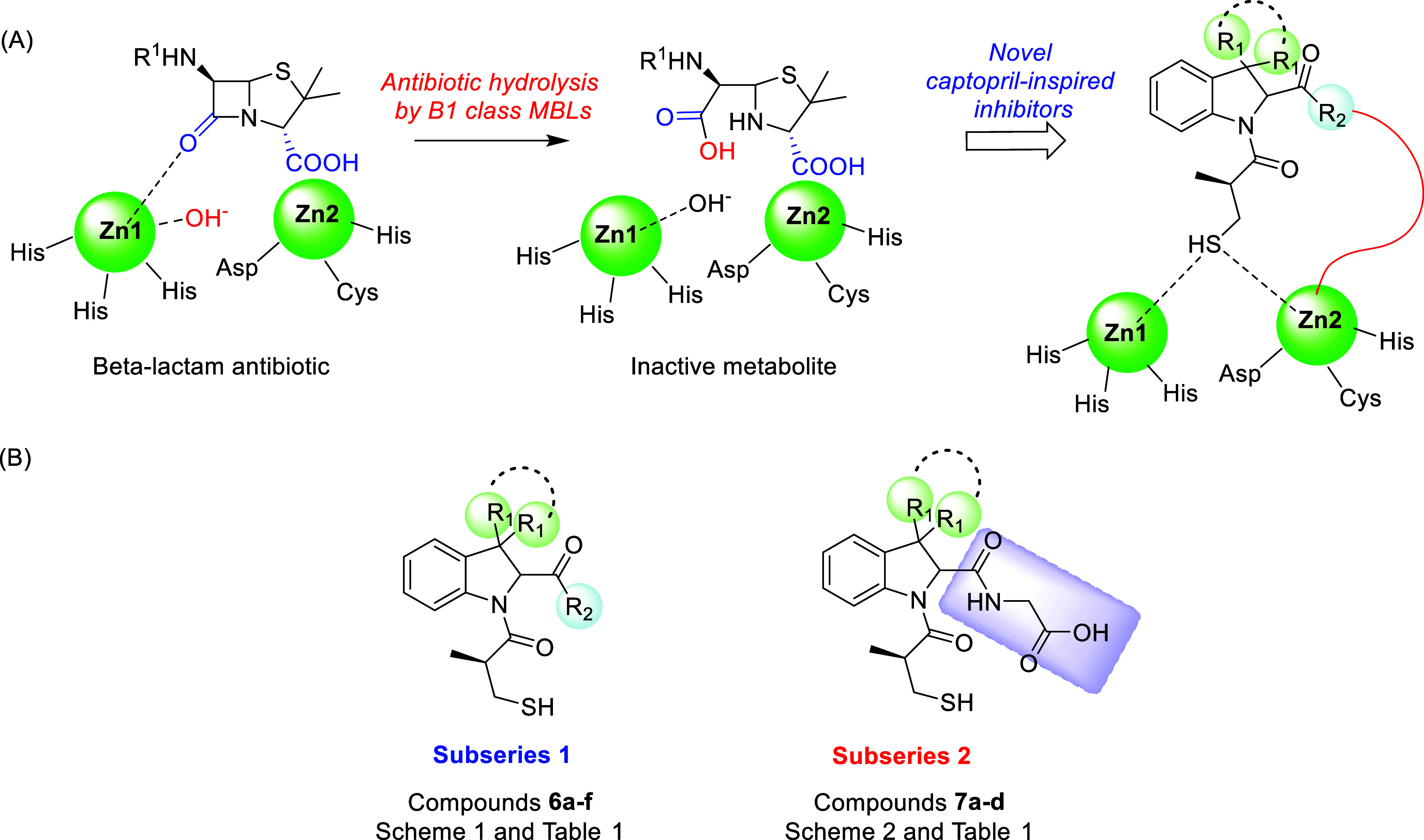
(A) Hydrolysis of a β-lactam antibiotic by MBLs and general scheme for rational design of novel MBL inhibitors; (B) subseries 1 (compounds **6a**–**f**) and subseries 2 (compounds **7a**–**d**) reported in the present work.

In compounds belonging to subseries 1, we incorporated the captopril structure within an indoline framework. According to our prediction, the indoline system, through the fusion of an aromatic system to the pyrrolidine ring of captopril, could allow our inhibitors to establish additional π–π or hydrophobic interactions within the active site of the enzyme including well-conserved residues in various MBL subtypes, such as Trp87. The second structural modification, aiming at increasing hydrophobic contacts within the active site, was performed on the 3-position of the indoline ring on which the effectiveness of either a spirocyclohexyl junction and the insertion of a 3,3-dimethyl or 3,3-diethyl groups were interrogated. Finally, the α-carboxylic functionality of the captopril proline substructure was replaced by a carboxamide moiety, keeping the hydrogen bonding potential within MBL active, while limiting the potential issue associated with many captopril carboxylic derivatives, which although to a lesser extent, can behave as inhibitors of the ACE-1 enzyme (Table 1 and Scheme 1, compounds **6a**–**f**).

**Scheme 1:**
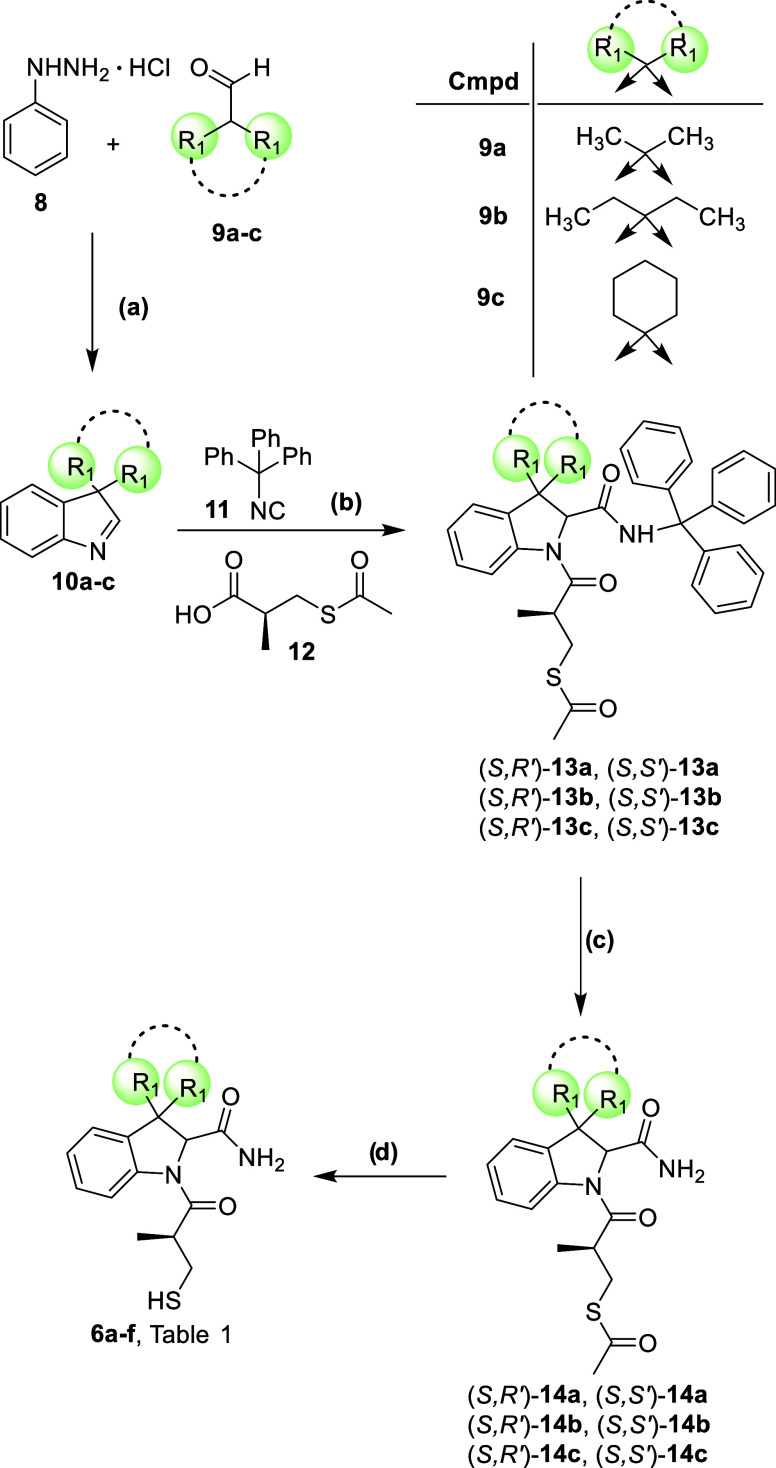
Synthesis of Compounds **6a**–**f** ^a^

a Reagents and conditions: (a) AcOH, 80 °C, 2 h; (b) CH_2_Cl_2_, sealed tube, 50 °C, 12 h; (c) TFA, DCM, 25 °C, 12 h; (d) THF, NaOH, 25 °C, 2 h.

We subsequently designed and synthesized another small subseries of compounds, obtained from a merging approach. Accordingly, we decided to verify if the introduction of a structural portion belonging to thiorphan (**3**) could be functional to obtaining new NDM-1 inhibitors. In particular, the 2-carboxamide functionality was replaced with the glycyl amide moiety of thiorphan (Table 1 and Scheme 2, compounds **7a**–**d**).

**Scheme 2:**
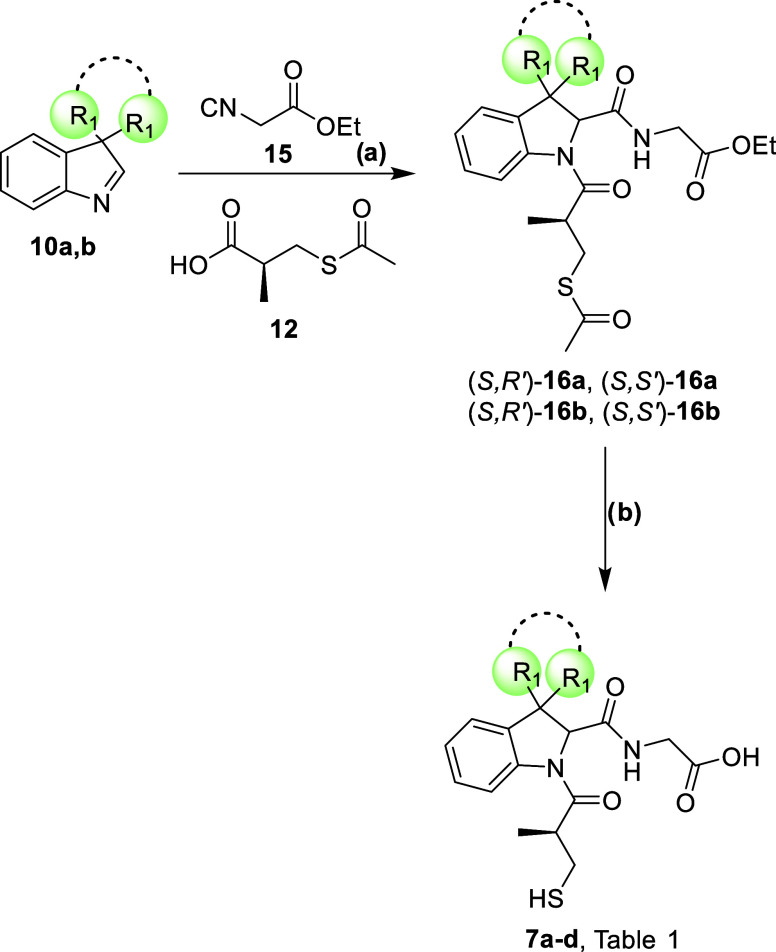
Synthesis of Compounds **7a**–**d** ^a^

a Reagents and conditions: (a) DCM, sealed tube, 50 °C, 12 h, (b) THF, NaOH, 25 °C, 2 h.

### 2.2 Synthesis of Novel Indoline-Based MBL Inhibitors

The synthesis of final compounds **6a**–**d** is reported in Scheme 1. The 3,3-disubstituted indolenines **10a**–**c** were obtained by an interrupted Fischer indolization reaction between the appropriate disubstituted α,α′-carbaldehydes **9a**–**c** and phenylhydrazine hydrochloride **8** in the presence of acetic acid with the dual role of solvent and acidic catalyst.^38^ Indolenines **10a**–**c** were then subjected to a multicomponent Joullié–Ugi reaction in the presence of trityl isocyanide **11**, prepared as previously reported, and of (*S*)-3-(acetylthio)-2-methylpropanoic acid **12** (a fragment present in the side chain of captopril), using dichloromethane as solvent under pressure at 50 °C. The reactions were stirred for 12 h at 50 °C.^39^ The multicomponent step provided compounds **13a**–**c** in the form of diasteroisomeric mixtures in approximately 1:1 *dr* (as determined by HPLC and NMR analysis). Trityl group deprotection carried out on the isolated diastereoisomers (separated by reverse phase HPLC) in the presence of trifluoroacetic acid in dichloromethane at room temperature provided the corresponding primary amides **14a**–**c**.^40^ The absolute stereochemistry of compound **14b** was verified by means of CD spectroscopy; the high similarity of the theoretical CD spectra of (*S*,*S*′)- and (*S*,*R*′)-**14b**, as obtained by DFT and TDDFT calculations, with the corresponding experimental spectra in methanol allows us to assign the absolute configuration to the chiral center on the indoline moiety without ambiguity (Figure 3 and Tables S1–S5). Final thioester hydrolysis with 2 N sodium hydroxide in tetrahydrofuran led to the final free thiols **6a**–**f**.^41^ It is worthy of note to clarify that thioester hydrolysis required a careful fine-tuning, in order to avoid epimerization issue. In particular, when performed over 12 h at room temperature, the reaction led to a relevant epimerization at position 2 of the indoline core. Satisfyingly, when reaction time was shortened to 2 h, the hydrolysis was complete and the stereochemical integrity was fully retained (<2% epimerization by NMR analysis).

**Figure 3:**
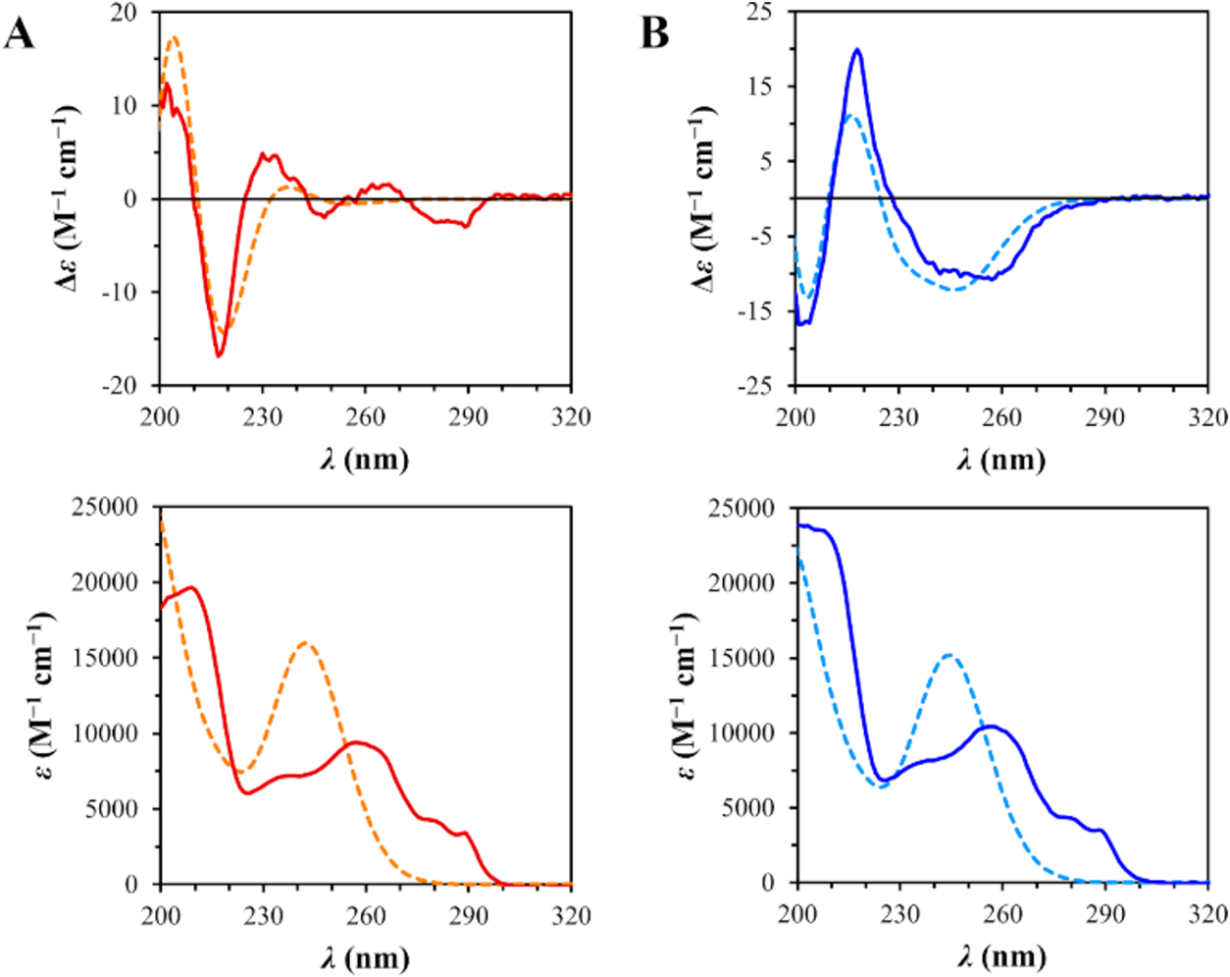
Stereochemical characterization of compound **14b**. CD (Δε) and UV (ε) spectra are shown in molar units; TDDFT-calculated theoretical spectra are here shown with a 0.2 eV blue shift for a clearer visual comparison. (A) Experimental (solid red) and theoretical (dashed orange) spectra of (*S*,*S*′)-**14b**. (B) Experimental (solid blue) and theoretical (dashed cyan) spectra of (*S*,*R*′)-**14b**.

The synthesis of final compounds **7a**–**d** is reported in Scheme 2. The formation of the indolenines was performed as described in the previous paragraph. The following Joullié–Ugi multicomponent step was performed in the presence of commercially available ethyl isocyanoacetate (**15**) and acid **12**. The mixtures of diastereomers **16a,b** were then separated using reverse phase HPLC and the generation of the final compounds **7a**–**d** was carried out on the single diastereoisomers in a single step, by alkaline hydrolysis of the ethyl ester and thioester functionalities in high yields and no detectable epimerization events.

In order to develop reproducible, efficient, and potentially more sustainable reaction conditions for the synthesis of our compounds, we have developed a flow-through procedure for the synthesis of the representative derivative **13c**.

The Joullié–Ugi multicomponent reaction was carried out in continuous flow, replacing dichloromethane with ethanol, a green solvent (Scheme 3). Accordingly, an equimolar solution of spiroindolenine **10c**, acid **12**, and trityl isocyanide **11** was prepared in ethanol (1 mL total volume) and injected into the sample loop. In our previous study, we optimized a multicomponent flow reaction by delivering isocyanide and carboxylic acid through separate streams to prevent premature side reactions and enhance the efficiency and selectivity. The solution was injected into the flow system, and pumping took place at a flow of 0.2 mL/min, at a temperature of 50 °C and a pressure of 7 bar, in a 15 mL tubular reactor. The total residence time to obtain the key intermediate **13c** was 75 min, with respect to the 720 min (12 h) required in batch mode.

**Scheme 3:**
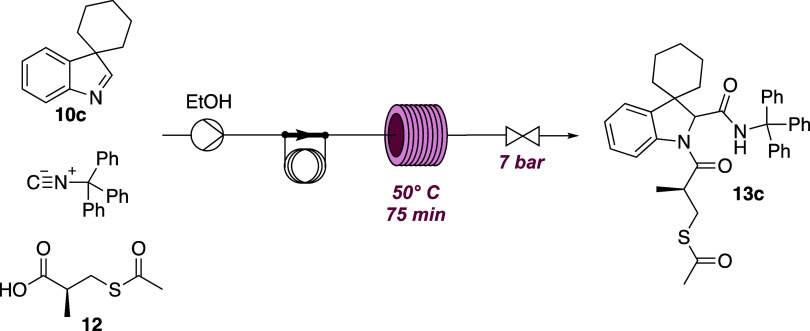
Continuous Flow Setup for the Synthesis of Intermediate **13c**

Furthermore, having already developed a procedure in telescoped mode combining interrupted Fischer reaction with subsequent Joullié–Ugi multicomponent reaction on the resulting indolenines,^39^ we applied the method for obtaining key intermediates **13a**–**c** and **16a**–**b** (Scheme 4), achieving comparable yields over two steps with respect to the batch synthesis (see Supporting Information), but avoiding, in this case, the isolation of the metastable spiroindolenine intermediates and saving 95% of batch time. The developed sustainable methodology will allow easy exploration of structure–activity relationships and further structural diversification in a sustainable fashion.

**Scheme 4:**
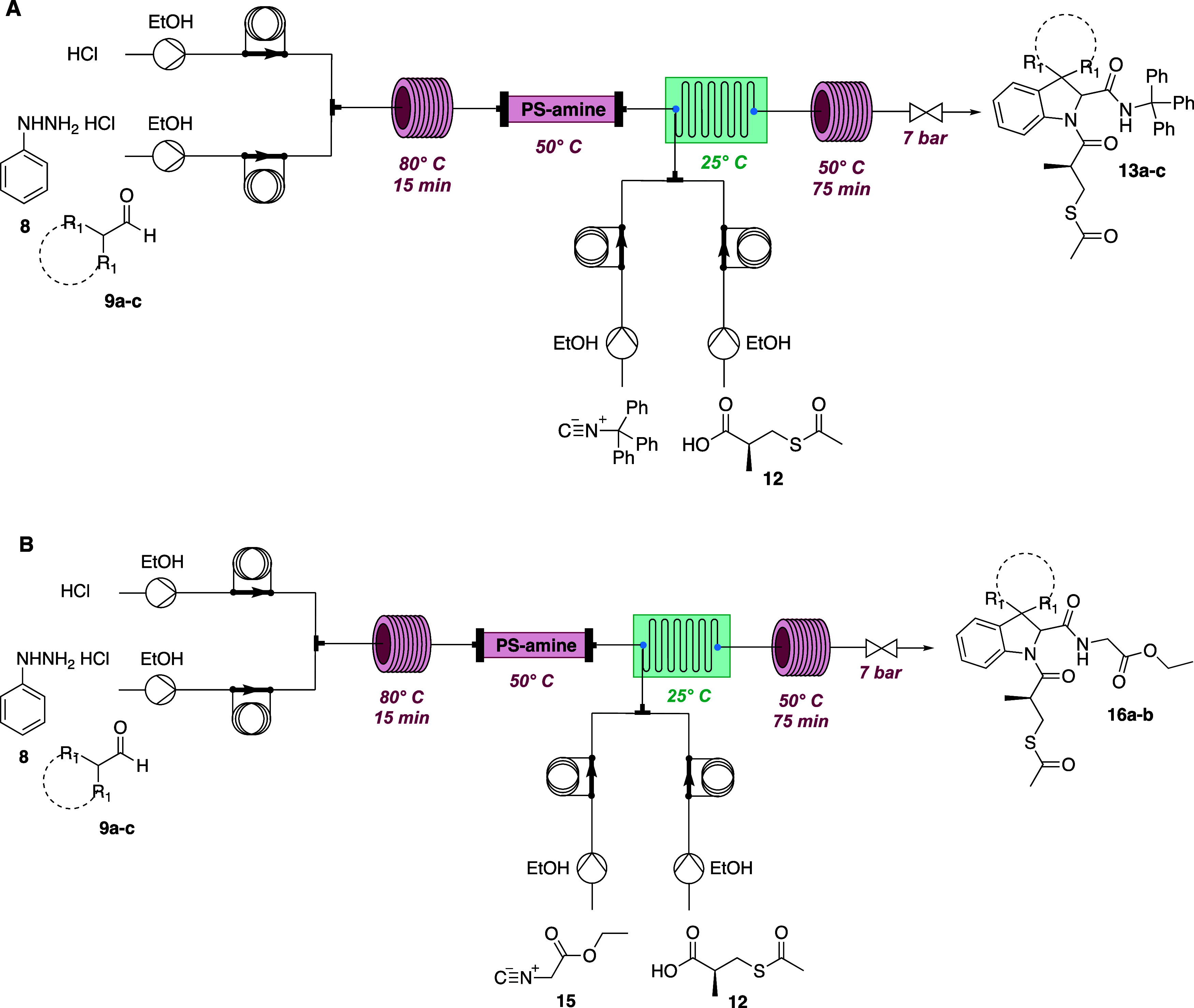
Telescoped Approach for the Synthesis of Key Intermediates (A) **13a**–**c** and (B) **16a**–**b**

### 2.3 Biological Studies and Structure–Activity Relationship Analysis

The inhibitory activity of the compounds was first evaluated against a panel of clinically relevant MBLs, including, besides NDM-1, other subclass B1MBLs (VIM-1, VIM-2, IMP-1, and IMP-7) to better assess their inhibition spectrum (Table 1). IC_50_ values were determined in a kinetic fluorescence-based *in vitro* assay as described previously.^42^ All compounds were preincubated with the recombinant protein for 30 min before addition of the fluorogenic substrate fluorocillin.^36,43^ The inhibitory activity was also determined (expressed as the percentage of inhibition in the presence of 50 μM compound) using a spectrophotometric assay in which the hydrolysis of imipenem (the reporter substrate) was monitored (see Section 4 for details).

Several considerations could be drawn from these results: (a) compounds **14b,c**, showing a protected thiol group in form of its thioester derivative, were rather expectedly not or very poorly active; (b) compounds **7a**–**d**, where the primary carboxamide functionality in position 2 was replaced with the glycyl amide present in thiorphan (**3**), are overall less active (up to 10-fold loss of inhibitory activity) than their corresponding 2-carboxamide analogues; (c) compounds with a free thiol group and an unsubstituted 2-carboxamide provided the best inhibitors, some of them showed a better activity than the reference compound D-captopril (**2**). Interestingly, the orientation of the indoline carboxamide and the nature of the hydrophobic 3-substituent (dimethyl, diethyl, or cyclohexyl) do not seem to have a major impact on the activity, suggesting that there is no specific interaction with the enzyme. Notably, compounds **6b**–**f** are the most potent and interesting compounds showing a remarkably broad spectrum of inhibition. In particular, a significant inhibition of IMP-type enzymes, considered rather structurally divergent (in terms of active site features and, more specifically, the nature of the residues present in the L3 loop at positions 61, 64, 67, and 87) from NDM- and VIM-type enzymes, was observed (Supporting Figure S1). Considering these primary results, the most promising compounds (**6c**–**f**) were selected for further investigation, and the *K*_*i*_ values were measured for NMD-1, VIM-2, and IMP-1 (Table 2). Compound **6b** was not further carried on since it displayed a lower chemical stability with respect to other compounds in the series.

**Table 2:** Inhibition Constants (*K*_*i*_) of Selected Compounds with Clinically Relevant Subclass B1MBLs

	*K*_*i*_ (μM)		
inhibitor	NDM-1	VIM-2	IMP-1
**6c**	4.5 ± 0.7	0.16 ± 0.01	4.9 ± 0.3
**6d**	2.6 ± 0.1	0.08 ± 0.01^a^	n.d.^b^
**6e**	2.0 ± 0.1	0.10 ± 0.01	3.8 ± 0.3
**6f**	2.1 ± 0.2	0.16 ± 0.01^a^	n.d.

a Measured as *K*_d_ (see text for details).

b n.d., not determined.

Interestingly, up to 30-fold lower *K*_*i*_ values were measured with VIM-2, despite the fact that these compounds were initially designed to target NDM-1. Compounds **6c** and **6e** were confirmed to inhibit the three divergent enzyme subtypes tested, confirming the rather broad spectrum of activity of these compounds. In kinetic assays, the compounds appear to rapidly inhibit both NDM-1 and IMP-1 (equilibrium was established within the time of mixing), but a different behavior was observed with VIM-2. Indeed, using a direct competition assay, a time-dependent enzyme inhibition was observed with compounds **6d** and **6f**, allowing us to measure a pseudo-first-order rate of inactivation (*k*_inact_). The analysis of the inhibitor concentration dependence of the *k*_inact_ allowed us to determine the rate constants characterizing the inhibitor-VIM-2 interaction (Table 3).

**Table 3:** Kinetic Parameters of VIM-2 Inhibition by the Selected Compounds

compound	*k*_+2_/*K* (M^–1^·s^–1^)	*K* (μM)	*k*_–2_ (s^–1^)	*K*_d_ (μM)
**6d**	1.2 × 10^4^	>30	5.0 × 10^–3^	0.08 ± 0.01
**6f**	1.5 × 10^4^	>20	2.6 × 10^–3^	0.16 ± 0.01

Such a behavior could be interpreted either with the production of the apoenzyme form after dissociation of the ternary complex (enzyme-zinc-inhibitor) or with slow and geometrically constrained rearrangements of some active site residues, leading to the formation of a more stable enzyme–inhibitor complex. The association rate (*k*_+2_/*K*) was similar for these compounds (≈10^4^ M^–1^ s^–1^), while the *k*_–2_ values were low, indicating the formation of a rather stable complex, although its formation is relatively slow. *K*_d_ values can be measured at steady state and support the slow formation of an otherwise stable and potently inhibited or inactivated form of the enzyme.

Furthermore, and to assess one of the primary aims of this study, i.e., redirecting the activity of captopril analogues from ACE-1 to MBLs, the inhibitory activity of compounds **6c**–**e**, among the most potent MBL inhibitor, was also assessed on ACE-1. Strikingly, these compounds, including analogues of both l- and d-captopril, did not show any inhibitory activity on ACE-1, even when tested at high concentration (<5% inh. at 50 μM), while several captopril preparations (pure isomers and racemic mixture) yielded the expected inhibitory activity (Table 4). Despite captopril being a rather good inhibitor of several MBLs (Table 1), obtaining derivatives showing similar or better potency on MBLs without cross-inhibition of ACE-1 represents a significant achievement.

**Table 4:** Inhibition of ACE-1 by Selected Compounds and Captopril^a^

compound	inhibition of ACE-1 activity (%)
d-captopril	8.9 ± 0.1
l-captopril	>99.5
captopril^b^	98 ± 1
**6c**	<5
**6d**	<5
**6e**	<5

a Assays were performed in the presence of 50 μM inhibitor in the reaction mixture (see the Experimental Section for details).

b Commercially available captopril, racemic mixture (Cayman Chemical cat. no. 15313).

Encouraged by these positive results, the potential synergistic activity of these compounds was tested in combination with imipenem on a panel of clinical isolates producing several different MBL enzymes (Table 5). Unfortunately, the potentiation of imipenem in the presence of most of the compounds was limited (no or unsignificant 2-fold decrease of the MIC value), indicating that, despite a good inhibitory activity of some of the tested compounds in enzyme assays (e.g., **6e**, **6f**), such molecules are apparently unable to reach periplasmic concentrations compatible with the inhibition of the MBL in the bacterial cell. Such results are not uncommon and likely rely on the lack of suitable properties of the molecules to readily diffuse through the bacterial outer membrane, whose presence still represents one of the major issues in the optimization of compounds targeting Gram-negative bacteria. However, compound **6d** showed a more significant 8-fold reduction of the imipenem MIC, although on a limited number of strains, including a VIM-1-producing *K. pneumoniae* isolate and an IMP-1-producing *P. aeruginosa* isolate.

**Table 5:** Synergistic Activity of MBLi Compounds (Tested at a Final Concentration of 32 μg/mL, Unless Otherwise Specified) with Imipenem on MBL-Producing Clinical Isolates

	imipenem MIC (μg/mL)^a^									
compound	*E. coli* SI-M001 (*bla*_NDM‑1_^+^)	*E. coli* SI-G001 (*bla*_NDM‑4_^+^)	*E. coli* SI–N003 (*bla*_NDM‑7_^+^)	*K. pneumoniae* T2301 (*bla*_NDM‑1_^+^)	*K. pneumoniae* 7023 (*bla*_VIM‑1_^+^)	*K. pneumoniae* T2216 (*bla*_VIM‑1_^+^)	*S. marcescens* SI-1591 (*bla*_VIM‑2_^+^)	*K. pneumoniae* VA*-*416/02 (*bla*_VIM‑4_^+^)	*A. baumannii* AC-54/97 (*bla*_IMP‑2_^+^)	*P. aeruginosa* T2325 (*bla*_IMP‑1_^+^)
None	64	64	64	16	128	4	16	128	128	64
**6b**	-^b^	-	-	16^c^	-	4^c^	-	-	-	64^c^
**6c**	64	64	64	-	128	-	8	128	128	-
**6d**	32	32	64	16^c^	16	2^c^	8	64	128	16^c^
**6e**	32	64	64	-	64	-	16	64	128	-
**6f**	32	64	64	-	64	-	16	128	128	-
**7a**	64	64	64	-	128	-	8	128	128	-
**7b**	64	64	64	8^c^	128	4^c^	16	128	128	32^c^
**7c**	64	64	64	-	128	-	16	128	128	-
**7d**	64	64	64	-	128	-	16	128	128	-
(*S,R*′)-**14b**	32	64	64	-	64	-	8	128	128	-
(*S,S*′)-**14c**	16	32	32	-	64	-	8	128	128	-

a Reported MIC values are the median from three independent experiments.

b -, Not determined.

c Compound tested at a final concentration of 128 μg/mL.

We further investigated the effects of the most promising compound **6d** to restore the antibacterial effect of imipenem in a growth inhibition assay (Figure 4).

**Figure 4:**
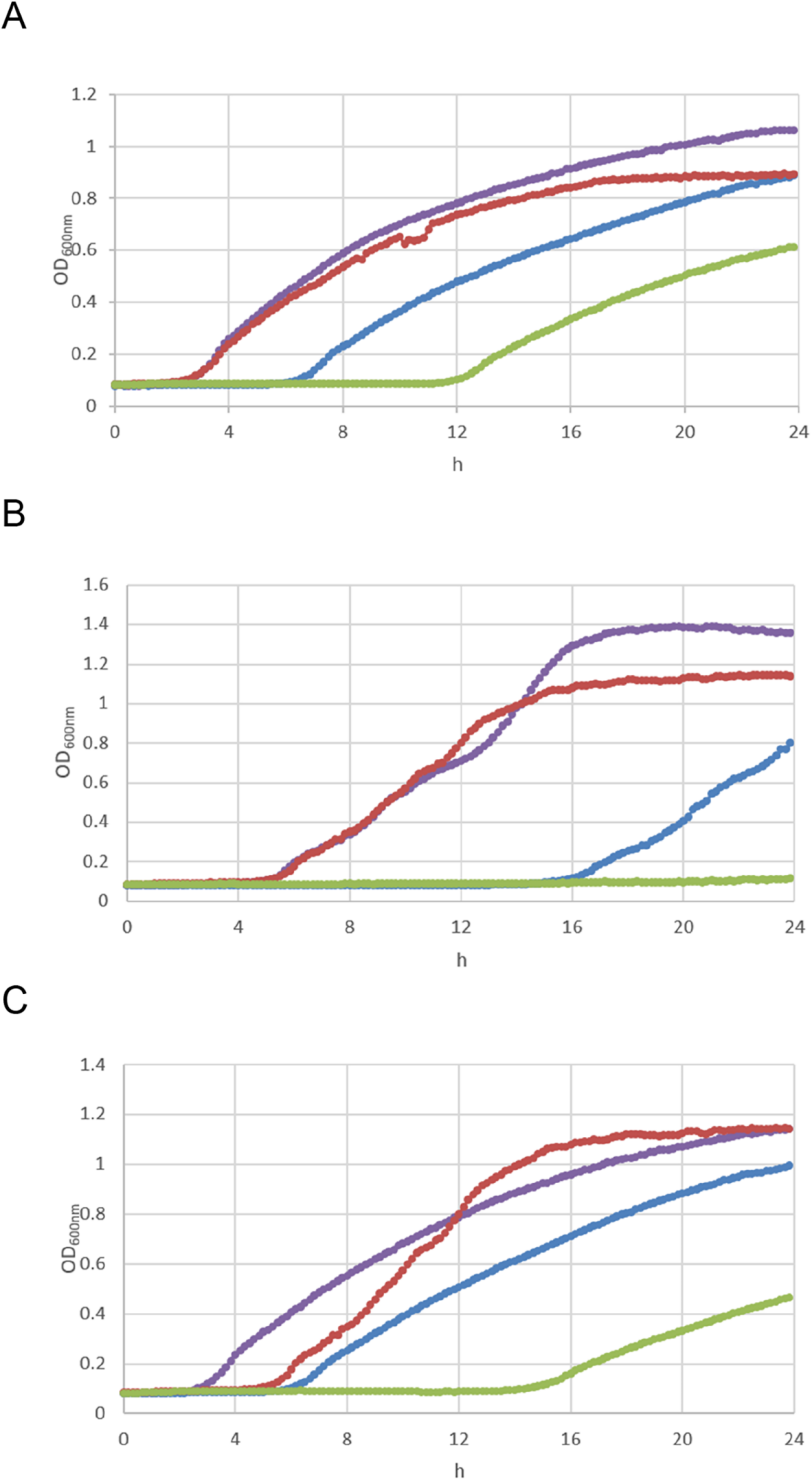
Growth inhibition assay. Growth curves of (A) NDM-1 expressing *K. pneumoniae* (T2301), (B) IMP-1 expressing *P. aeruginosa* (T2325), and (C) VIM-1 expressing *K. pneumoniae* (T2216) in the absence and presence of imipenem at 0.5 × MIC ± **6d** at 128 μg/mL. Purple = growth control; red = **6d**; blue = imipenem; green = imipenem plus **6d**.

As shown in the growth curves in Figure 4, the presence of imipenem at 0.5 × MIC significantly delayed the growth of MBL-producing strains, although little difference could be observed after 24 h of incubation in the presence of either imipenem alone or of the combination. Interestingly, when MBL inhibitor **6d** was tested at 128 μg/mL in the presence of 0.5 × MIC of imipenem, the inhibitory potential of imipenem was further prolonged for several hours. MBL inhibitor **6d** alone did not exhibit any intrinsic antibacterial activity up to 128 μg/mL. These data highlight the promising potential of **6d** and prompt a further optimization campaign in order to improve the cell-penetrating profile and this new class of broad-spectrum MBL inhibitors.

From this focused set of compounds, it is possible to infer a series of significant hints for further design and optimization: (i) most of the developed compounds showed an improved inhibition profile on NDM-1, with respect to the progenitors l- and d-captopril; (ii) as expected, compounds bearing the capping acetyl moiety on the thiol group were completely inactive against NDM-1, thus confirming the importance of the free thiol group for the interaction with the zinc ions in the catalytic site; (iii) in line with the trend observed for l- and d-captopril isomers, stereochemistry at the 2-position of the indoline system seems to be only moderately relevant in the first subseries of compounds (**6a**–**f**) and slightly more influential in the second subseries (**7a**–**d**), with the *R* configuration guaranteeing in both cases better performances in terms of inhibitory activity; (iv) compound **6d** demonstrated negligible activity on ACE, thus validating our design while averting off-target liability for this newly conceived class of compounds.

In order to elucidate and unveil the interactions of the compounds at the molecular level, the possible binding mode of selected inhibitors **6d**, **6c**, and **6e** against the catalytic site of NDM-1, VIM-1, VIM-2, IMP-1, and IMP-7 isoforms was elucidated by molecular docking and dynamics (MD) simulations, with the aim to rationalize the inhibitory activity results while obtaining key hints for further design activity.

Before docking, the evaluation of the entropic and enthalpic contributions of crystallographic water molecules within the catalytic site was carried out by SZMAP software.^23^ Water molecules estimated to provide a positive contribution to ligand binding were retained in the receptor’s structure during docking. Molecular docking was carried out with the GOLD program (The Cambridge Crystallographic Data Centre),^22,24^ whose accuracy was preliminarily verified by redocking the cocrystallized inhibitor into the X-ray structures of MBL isoforms investigated in this work (RMSD of docking vs crystallographic pose <1.00 Å, data not shown).

We first took into consideration the captopril binding to NDM-1; in particular, we considered the two solved X-ray complexes of NDM-1 protein with l-captopril (PDB code: 4EXS, Figure 5A)^33^ and d-captopril (PDB code: 5ZJ2, Figure 5B),^34^ from where it can be noticed that the L3 of NDM-1 assumes two different conformations in the presence of these two inhibitors (Figure 5C). In addition, a different orientation of the proline ring can be observed in the X-ray cocrystal structure of d-captopril, which allows further hydrophobic interactions with Met67 and especially Phe70 residues in the L3, thus supporting the closed conformation of the loop. The different orientation of the carboxylate group also allows d-Captopril to establish a water-bridged H-bond interaction with Lys211 (Lys224 in the standard numbering scheme) in loop L10 and another H-bond with the conserved Asn220 (Asn233 in the standard numbering scheme) that is known to play an important role in substrate positioning and β-lactam hydrolysis in NDM-1 and other subclass B1MBLs. These two interactions increase the binding of d-Captopril with the active site of NDM-1 and contribute to its higher potency compared to that of l-Captopril. Similar findings were observed by monitoring the interaction of d- and l-Captopril to VIM-2 in available X-ray crystallography structures (Supporting Figure S2), with the only difference relating to the direct interaction between the carboxylate moiety of d-Captopril to Arg205 in VIM-2 compared to the water-bridged interaction to Lys211 of NDM-1 such as described above.

**Figure 5:**
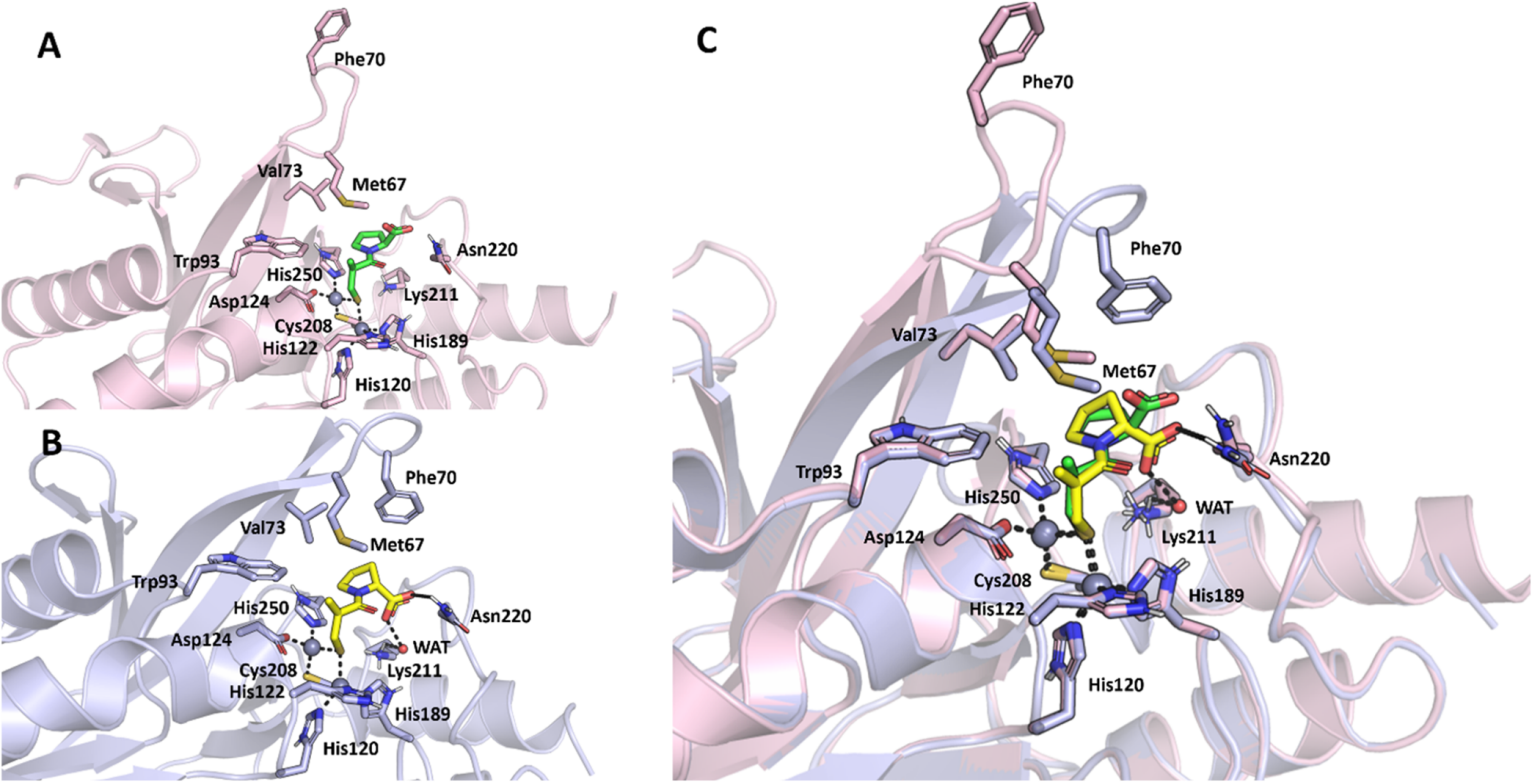
X-ray crystallography structure of NDM-1 in complex with l- and d-captopril. (A) X-ray crystallography complex of NDM-1 (pink, “open” conformation, PDB-ID: 4EXS) with l-captopril (green sticks); (B) X-ray crystallography complex of NDM-1 (light blue, “closed” conformation, PDB-ID: 5ZJ2) with d-captopril (yellow sticks); (C) superposition of NDM-1 structures in complex with l-captopril (pink) and d-captopril (light blue). Polar interactions are highlighted by black dashed lines. Residues involved in zinc coordination and in binding l- and d-captopril are shown as sticks and are labeled.

Compounds investigated in this work were first docked against the “open” conformation of NDM-1 (PDB code: 4EXS, data not shown),^33^ failing to explain the different inhibitory activities observed *in vitro* in the enzymatic assays. In contrast, performing the docking on the “closed” conformation of NDM-1 in complex with d-captopril (PDB code: 5ZJ2)^34^ provided a suitable correlation between predicted binding modes and experimental results, also supporting that compounds of both the two subseries presenting the *R* configuration at the 2-position of the indoline ring system have a more favorable binding mode and docking score compared to their *S* counterparts. Computational results obtained with the most representative compounds of the series, based on chemical structure and biological activity, are illustrated below.

Based on the IC_50_ values of Table 1, **6d** was initially docked to the active site of NDM-1, VIM-1, and IMP-7. Docking results (Figure 6) clearly indicate that the sulfhydryl moiety of **6d** coordinates the two catalytic Zn­(II) ions by positioning them in the middle of them in all tested isoforms. In NDM-1, **6d** establishes direct H-bond interactions with Asn220 and Lys211 from the loop L10, while an additional interaction to Lys211 is bridged by a water molecule (involving the amide nitrogen of **6d**). The indole ring participates in a π–π stacking interaction with His250, and it is docked within a hydrophobic region composed of Met67, Phe70, Val73, and Trp93 (Figure 6A). With VIM-1, **6d** is H-bonded to the conserved Asn210(233) and binds to L3 through a π-π stacking interaction with Tyr67 (Figure 6B). On IMP-7, **6d** establishes H-bonds with Asn185, His215, and Glu41, while it interacts with the L3 through π-π stacking interaction with Trp46 (Figure 6C). For the sake of clarity, residue numbering corresponds to the crystallographic structures used in this molecular modeling study.

**Figure 6:**
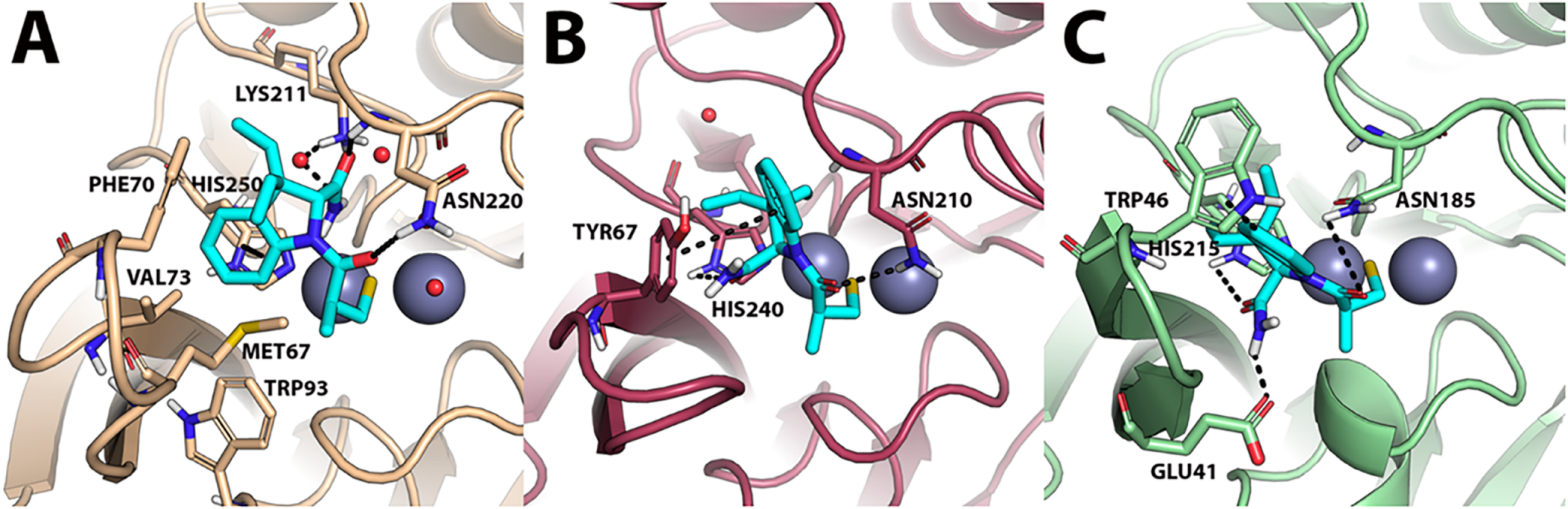
Predicted binding mode of **6d** against (A) NDM-1 (colored beige; PDB-ID: 5ZJ2), (B) VIM-1 (colored Bordeaux; PDB-ID: 7UP2) and (C) IMP-7 (colored green; homology model). **6d** is shown as cyan sticks, and the two zinc atoms as a gray sphere. Polar interactions are highlighted by black dashed lines, residues contacted by the ligand are shown as sticks, while residues within about 5 Å from the ligands are shown as lines. Water molecules are represented by small red spheres.

Based on the *K*_*i*_ data of Table 2, **6c** and **6e** were docked to the catalytic site of NMD-1, VIM-2, and IMP-1 MBLs. Also in this case, docking results clearly indicate that Zn­(II) coordination by the sulfhydryl moiety in the middle of the two zinc ions is a crucial binding feature for **6c** and **6e** to anchor the catalytic site. Notably, **6c** and **6e** have a highly superimposable binding pose to NDM-1, VIM-2, and IMP-1, which may explain their similarity in *K*_*i*_ values. In NDM-1, **6c** and **6e** are H-bonded to Asn220 and Lys211, while the indole ring participates in a T-shaped π-stacking interaction with Phe70 (Figure 7A,B). In VIM-2, both compounds establish H-bond interactions with the Zn-binding His240, Asn210, and Arg205, while an additional T-shaped π-stacking interaction with Tyr67 from the L3 loop is observed (Figures 7C and 6D). Finally, within IMP-1, **6c** and **6e** establish an H-bond interaction with Asn185, while the indole ring is π–π stacked with His215 (Figure 7E,F).

**Figure 7:**
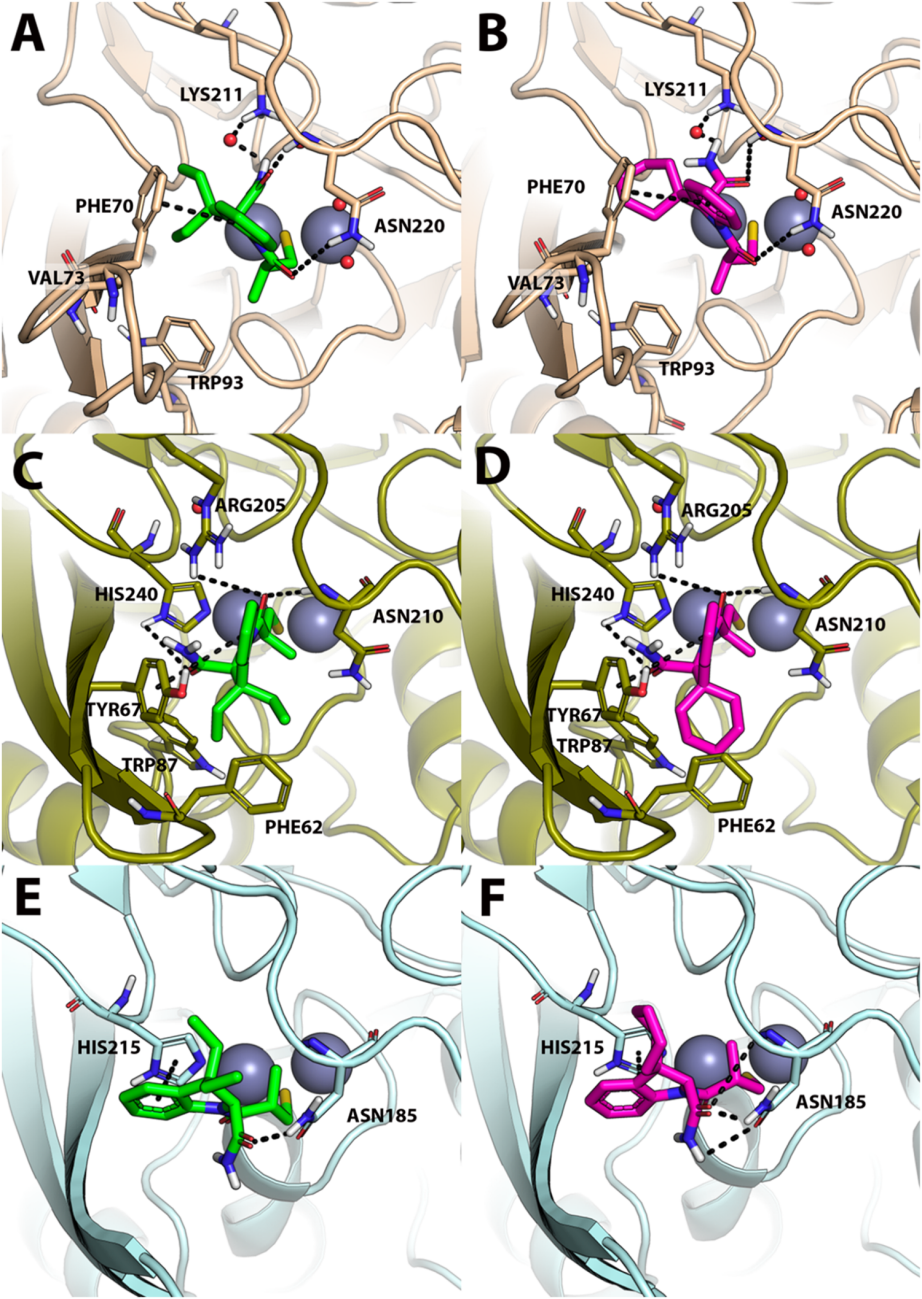
Predicted binding mode of **6c** and **6e** against (A, B) NDM-1 (colored beige; PDB-ID: 5ZJ2), (C, D) VIM-2 (colored dark green; PDB-ID: 6JN6) and (E, F) IMP-1 (colored light blue, PDB-ID: 7YHA). **6c** is shown as green sticks, while **6e** is shown as magenta sticks, the two zinc atoms as a gray sphere. Polar interactions are highlighted by black dashed lines, residues contacted by the ligand are shown as sticks, while residues within about 5 Å from the ligands are shown as lines. Water molecules are represented by small red spheres.

Despite the known sequence and structural differences between MBL isoforms investigated in this work, docking results highlight a common interaction pattern for **6c**, **6d**, and **6e** within the catalytic site of the enzymes. Of note, MBL inhibitors bind the catalytic Zn­(II) ions as well as key residues that are involved in zinc coordination and in β-lactam substrate recognition and chemical transformation, providing structural hints that corroborate the broad-spectrum efficacy of **6c**–**6e** such as observed by experiments.

To assess the accuracy of docking poses and to evaluate their time persistency, MD simulations were performed on a representative system, i.e., the complex between **6d** and NDM-1. Unrestrained all-atom MD trajectories were generated for 500 ns in explicit water solvent and further analyzed for the root mean square deviation (RMSD) and MD frame clustering. The RMSD plot (Figure S3) shows that the interaction between **6d** and NDM-1 is stable over the simulation time.

The further visual inspection of the most representative MD frame (i.e., the centroid of the cluster with the highest frame population as extracted by cluster analysis) suggests that **6d** stably binds the catalytic site in a conformation that resembles the docking pose (Figure 8). In fact, Zn­(II) coordination by the sulfhydryl moiety and H-bond interactions with Lys211 and Asn220, as identified by docking, are conserved in the MD pose; similarly, the crystallographic water molecule bridging **6d** to Lys211 is stably found also in the MD trajectory (red sphere in Figure 8). Nevertheless, a slight conformational change in the catalytic site was observed by MD, which is mostly due to a reciprocal and lipophilic-based approach of the indole ring of **6d** and the hydrophobic portion of NDM-1 within the loop L3 composed of Met67, Phe70, Val73, and Trp93 (Figure S4).

**Figure 8:**
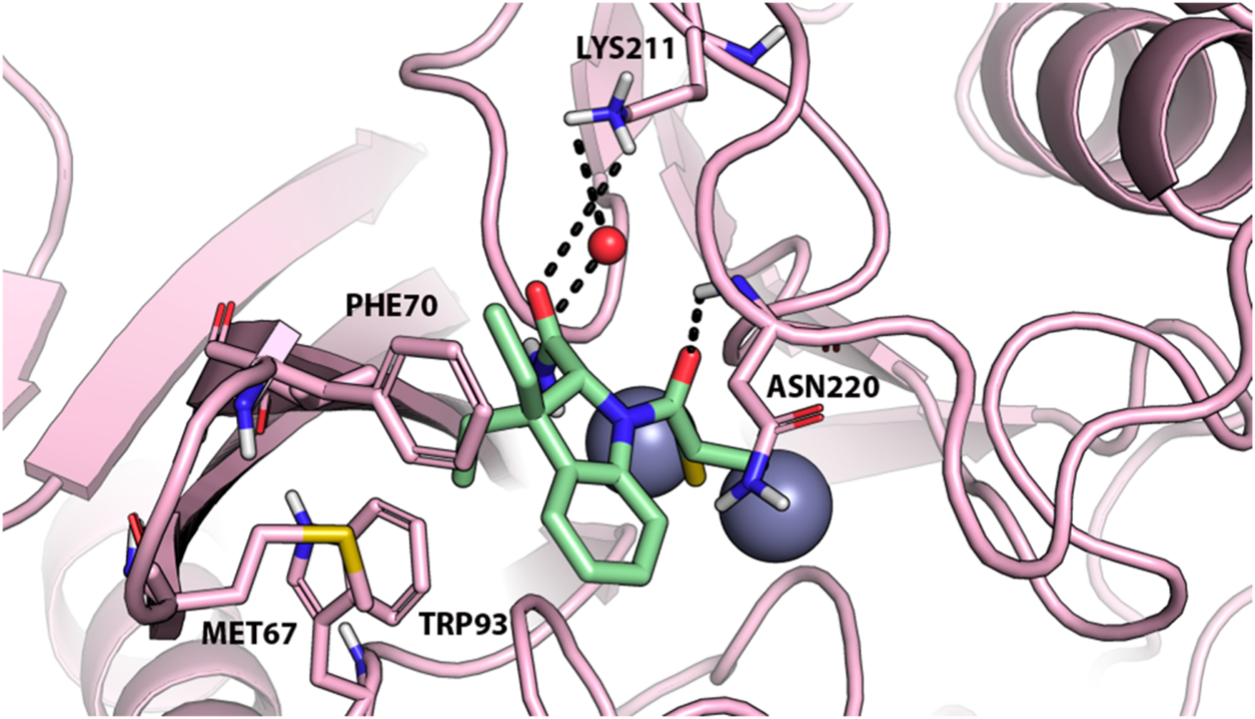
Representation of the most representative frame derived from the cluster analysis on the MD trajectory (cluster with the highest frame population), NDM-1 is represented by pink cartoon, while **6d** is shown as light-green sticks. The two zinc atoms are represented as a gray sphere. Polar interactions are highlighted by black dashed lines, residues contacted by the ligand are shown as sticks, while residues within about 5 Å from the ligands are shown as lines. Water molecules are represented by small red spheres (structure from molecular dynamics).

Overall, docking simulations coupled with MD provided a detailed picture of the interaction between small-molecule inhibitors and target MBLs, which paves the way to the further optimization of these derivatives as well as the design of additional chemotypes of MBL inhibitors.

## 3 Conclusions

In summary, the present study describes the design, synthesis, and biological evaluation of two indoline-based subseries of MBL inhibitors inspired by captopril drug. In order to develop an efficient, versatile, and sustainable synthetic protocol, the generation of the compounds employed a one-pot multicomponent reaction, implemented in both batch and flow modes. Most of the designed compounds showed improved NDM-1 inhibitory activity with respect to the captopril parent compound. Several indoline-based derivatives also presented broad-spectrum activity on clinically relevant MBL subtypes. Notably, for some of the compounds, a significant inhibition of IMP-type enzymes was observed, despite these enzymes being considered structurally divergent (in terms of active site features) from NDM- and VIM-type enzymes.

The inhibition kinetics were also elucidated, and the compounds appear to inhibit both NDM-1 and IMP-1 very quickly, but different behavior was observed with VIM-2. Indeed, using a direct competition assay, a time-dependent enzyme inhibition was observed with compounds **6d** and **6f**, allowing us to measure a pseudo-first-order rate of inactivation (*k*_inact_). The analysis of the inhibitor concentration dependence of the *k*_inact_ allowed determination of the rate constants characterizing the inhibitor-VIM-2 interaction.

Furthermore, the inhibitory activity of compound **6d**, one of the most potent MBL inhibitors of the series, was also assessed on the ACE-1 enzyme, the original target of l-captopril drug. Strikingly, this compound was completely inactive on ACE-1, thus averting this off-target effect for the newly conceived class of compounds.

When assessed for its synergistic activity in combination with imipenem on a panel of MBL-producing clinical isolates, compound **6d** showed a significant 4-fold reduction of the imipenem MIC in a VIM-1-producing *K. pneumoniae* isolate and in an IMP-1-producing *P. aeruginosa* isolate. On the contrary, compounds **6e,f**, despite a good *in vitro* inhibitory profile, proved unable to reduce the imipenem MIC, possibly in relation to their limited diffusion through the outer membrane, leading to periplasmic concentrations insufficient to inhibit the MBL in the bacterial cell, although other mechanisms, such as active efflux, could not be ruled out.

Growth inhibition assay in combination with imipenem demonstrated that compound **6d** was able to prolong the inhibitory potential of imipenem for several hours. Collected together, these data highlight the promising potential of **6d** and prompt a further optimization campaign in order to improve the potency and cell-penetrating profile of this new class of broad-spectrum MBL inhibitors.

In order to elucidate and unveil the interaction of the compounds at the molecular level, the possible binding mode of selected inhibitors, namely, **6c**, **6d**, and **6e**, against the catalytic site of selected NDM-, VIM-, and IMP-type MBLs was elucidated by detailed computational studies. Overall, docking simulations coupled with molecular dynamics provided a detailed picture of the interaction between the best-performing compounds of the series and the target MBL variants, which will pave the way for further optimization. Future activities aimed at improving the potency and expanding the spectrum of inhibition for this newly conceived captopril-inspired MBL inhibitors will include rational stepwise modification or replacement of the original captopril side chain as well as explorations of the nature and the length of the amide moiety of thiorphan-like series.

## 4 Experimental Section

### 4.1 Chemistry

#### 4.1.1 General Remarks

Unless otherwise specified, the materials were purchased from commercial suppliers and used without further purification. TLC analysis was conducted using aluminum foil-supported thin-layer silica gel chromatography plates (F254 indicator). Column chromatography was performed using 230–400 mesh and 60 Å pore diameter silica gel. For ^1^H NMR and ^13^C NMR measurements, an accurately weighed amount of analyte (about 5.0–10.0 mg) was dissolved in 600 μL of dimethyl sulfoxide (DMSO-*d*_6_). The mixture was transferred into a 5 mm NMR tube, and the spectra were acquired on a Bruker Advance 400 MHz spectrometer by using the residual signal of the deuterated solvent as internal standard. Splitting patterns are described as singlet (s), doublet (d), triplet (t), quartet (q), and broad (br); the values of chemical shifts (δ) are given in ppm and coupling constants (J) in Hertz (Hz). NMR data were processed with MestreNova (ver. 8.1.1, Mestrelab Research). ESI-MS spectral analysis was carried out on a mass spectrometer LTQ-XL. High-resolution ESI-MS spectra were performed on a Thermo LTQ Orbitrap XL mass spectrometer. The spectra were recorded by infusion into the ESI source using MeOH as the solvent. HPLC was performed with a Waters Model 510 pump equipped with a Waters Rheodyne injector and a differential refractometer, model 401. Luna 5 μm PFP (2) 100A HPLC Column 250 mm × 10 mm was employed. Purity of the compounds is >95%.

#### 4.1.2 Continuous Flow Synthesis of Indolenines **10a**–**c** (Scheme 1)

Chemical transformations in flow were realized using a self-made flow reactor: two Waters P510 HPLC pumps were connected, each to a Rheodyne 9010 injection valve, each equipped with a 1 mL sample loop (SL) made from PTFE tubing and an injection port for disposable syringes. The injection valves were further connected via a T-piece to a coiled PTFE tubing of a 15 mL volume. The coil was immersed in a silicone oil bath for controlling the reaction temperatures. The tubular reactor finished as a back pressure regulator (BPR), which was either spring mechanism-based (6.7 bar) or simply a PEEK capillary of defined length (1.7 bar). Product containing exiting streams were collected directly in a flask. An aliquot of the collected phase was used for analysis by GC-MS. Reagents were prepared for loading into the sample loops according to one of the following methods: 0.25 mmol aldehyde +0.25 mmol PhNHNH_2_·HCl in 1 mL of EtOH, heated to 50 °C for 5 min prior to loading into the SL; SL B: (equiv.-1) HCl in 1 mL of EtOH. 3,3-Dimethyl-3*H*-indole **10a**, 3,3-diethyl-3*H*-indole **10b**, spiro­[cyclohexane-1,3′-indole] **10c** were synthesized in according to a previously reported procedure.^38^

#### 4.1.3 Batch Synthesis of Key Intermediates **13a**–**c**, **16a**–**b** (General Procedure A)

Following the reported procedure in the literature,^39^ indolenine **10a**–**c** (3 mmol) were dissolved in DCM (0.3 M), followed by the addition of the corresponding amino acid (1.0 equiv) and isocyanide (1.0 equiv). The reaction mixture was stirred for 24–30 h at 50 °C in a sealed tube. Then, DCM was evaporated under vacuum to achieve the crude residue that was subjected to column chromatography (SiO_2_, corresponding eluent) and to HPLC to afford a diastereomeric mixture.

#### 4.1.4 Flow Synthesis of Key Intermediates **13a**–**c**, **16a**–**b** (General Procedure B)

Chemical transformations in flow were realized using a self-made flow reactor: Waters P510 HPLC pumps were connected to a Rheodyne 9010 injection valve, equipped with a 1 mL sample loop made from PTFE tubing and an injection port for disposable syringes. The injection valve was further connected to a coiled PTFE tubing of a 15 mL volume. The coil was immersed in a silicone oil bath for controlling reaction temperatures. The tubular reactor was fitted to a mechanical back pressure regulator (BPR) of approximately 7 bar. Products containing exiting streams were collected directly in a flask.

A solution of indolenines **10a**–**c** (0.25 mmol), the suitable acid (1 equiv), and the appropriate isocyanide (1 equiv) was prepared in EtOH (1 mL total solution volume). The flow reactor was heated to 80 °C. EtOH was used as solvent at a total flow rate of 0.2 mL/min to move the reaction mixture through the coil reactor for a residence time of 75 min.

#### 4.1.5 Batch Synthesis of Compounds **14a**–**c** (General Procedure C)

The multicomponent key intermediates **13a**–**c** were dissolved in DCM/TFA 1:1, and the reaction was stirred overnight at room temperature. The mixture was washed with NaHCO_3_ and extracted with DCM. The solvent was evaporated under a vacuum, and the reaction was purified by chromatography on silica gel.

#### 4.1.6 Batch Synthesis of Final Products **6a**–**f**, **7a**–**d** (General Procedure D)

The ester intermediates were dissolved in THF/NaOH (1:3, 0.5 mL/1.5 mL), and the reaction was stirred for 2 h at room temperature. The reaction was quenched with 2 N HCl and extracted with ethyl acetate. Then, the solvent was evaporated under vacuum, and the reaction was purified by chromatography on silica gel to afford the final title products.

#### 4.1.7 (2*S*)-3-((3,3-Dimethyl-2-(tritylcarbamoyl)­indolin-1-yl)-2-methyl-3-oxopropyl)­ethanethioate (**13a**)

According to general procedure A, a mixture of 3,3-dimethyl-3*H*-indole **10a**, (351 mg, 2.42 mmol), 3-(acetylthio)-2-methylpropanoic acid **12** (242 μL, 2.66 mmol), and trityl isocyanide **11** (716 mg, 2.66 mmol) was dissolved in DCM (5 mL) in a sealed tube at 50 °C for 30 h. The crude product was purified by chromatography on silica gel with hexane/EtOAc (85:15) to yield the multicomponent products in the form of a diastereomeric mixture. The diastereoisomers were subjected to reversed-phase HPLC using MeOH/H_2_O (85:15) as the eluent (flow rate 3.00 mL/min). Total yield: 26% (*dr* 1:1).

**(** ***S***, ***S*** **′)-13a**: Yellowish solid; *R*_f_ = 0.38 (H/E 7:3). ^1^H NMR (700 MHz, DMSO-*d*_6_) δ 9.27 (s, 1H), 7.90 (d, *J* = 8.0 Hz, 1H), 7.30–7.26 (m, 15H), 6.97 (m, 3H), 4.98 (s, 1H), 3.15 (dt, *J* = 13.6, 6.3 Hz, 2H), 2.89 (h, *J* = 6.8 Hz, 1H), 2.29 (s, 3H), 1.29 (d, *J* = 6.9 Hz, 3H), 1.24 (s, 3H), 1.16 (s, 3H). ^13^C NMR (176 MHz, DMSO-*d*_6_) δ: 195.8, 168.5, 144.6, 129.2, 128.8, 128.0, 127.7, 127.0, 126.7, 71.2, 44.9, 31.5, 31.1, 29.5, 29.2, 20.4, 18.2.

*t*R HPLC: 12.57 min. [α]_D_^20^ = −311.54 (*c* 0.3, MeOH).

MS (ESI) *m*/*z* calc. [M + H]^+^ C_36_H_36_N_2_O_3_S^+^: 577.24475; found: 577.25128.

**(** ***S***, ***R*** **′)-13a**. Yellowish solid; *R*_f_ = 0.38 (H/E 7:3). ^1^H NMR (700 MHz, DMSO-*d*_6_) δ 9.18 (s, 1H), 7.94 (d, *J* = 8.0 Hz, 1H), 7.28 (m, 10H), 7.22–7.15 (m, 3H), 7.14–7.06 (m, 2H), 7.05 (d, *J* = 7.3 Hz, 1H), 6.99–6.92 (m, 2H), 4.98 (s, 1H), 3.09 (dd, *J* = 13.4, 6.2 Hz, 1H), 3.02 (dd, *J* = 13.3, 7.5 Hz, 1H), 2.81–2.75 (m, 2H), 2.39 (s, 2H), 1.27 (s, 3H), 1.24 (s, 3H), 1.00 (d, *J* = 3.9 Hz, 3H). ^13^C NMR (176 MHz, DMSO-*d*_6_) δ 195.5, 172.9, 168.70, 144.7, 142.3, 140.9, 128.8, 128.0, 127.5, 126.9, 124.1, 121.9, 116.7, 72.4, 70.4, 45.1, 38.4, 33.2, 31.9, 31.1, 29.5, 21.5, 17.7.

tR HPLC: 11.49 min. [α]_D_^20^ = −21.32 (*c* 0.3, MeOH).

MS (ESI) *m*/*z* calc. [M + H]^+^ C_36_H_36_N_2_O_3_S^+^: 577.24475; found: 577.25128.

#### 4.1.8 (*S*)-3-((*S*)-2-Carbamoyl-3,3-(dimethylindolin-1-yl)-2-methyl-3-oxopropyl)­ethanethioate ((*S*,*S*′)-**14a**)

According to procedure C, (*S*,*S*′)-**13a** (90 mg, 0.15 mmol) was dissolved in DCM/TFA 1:1 (4.5 mL/4.5 mL) and the reaction was stirred overnight at room temperature. The mixture was washed with NaHCO_3_ and extracted with DCM. Then, the solvent was evaporated under vacuum, and the reaction was purified by chromatography on silica gel with hexane/EtOAc (1:1) to yield the product as a yellowish solid. Yield: 54% (27 mg). *R*_f_ = 0.17 (H/E 1:1).

^1^H NMR (700 MHz, DMSO-*d*_6_) δ 8.08 (d, *J* = 8.0 Hz, 1H), 7.78 (s, 1H), 7.38 (s, 1H), 7.19 (d, *J* = 7.4 Hz, 1H), 7.16 (t, *J* = 7.7 Hz, 1H), 7.02 (t, *J* = 7.4 Hz, 1H), 4.58 (s, 1H), 3.04 (dd, *J* = 13.2, 5.8 Hz, 1H), 2.99 (dd, *J* = 13.2, 8.6 Hz, 1H), 2.75 (dq, *J* = 12.7, 6.2 Hz, 1H), 2.33 (s, 3H), 1.32 (d, *J* = 18.2 Hz, 6H), 1.08 (d, *J* = 6.6 Hz, 3H). ^13^C NMR (176 MHz, DMSO-*d*_6_) δ: 195.0, 173.0, 170.4, 141.9, 140.1, 127.2, 123.7, 121.8, 116.3, 72.6, 43.2, 38.2, 32.7, 31.9, 30.5, 22.2, 17.1.

[α]_D_^20^ = −52.64 (*c* 0.3, MeOH).

MS (ESI) *m*/*z* calc. [M + H]^+^ C_17_H_22_N_2_O_3_S^+^: 335.14; found: 335.14

#### 4.1.9 (*S*)-1-((*S*)-3-Mercapto-2-methylpropanoyl)-3,3-dimethylindoline-2-carboxamide (**6a**)

According to procedure D, (*S*,*S*′**)-**14a** (20 mg, 0.06 mmol) was dissolved in THF/NaOH (1:3, 0.5 mL/1.5 mL) and the reaction was stirred for 2 h at room temperature. The mixture was quenched with 2 N HCl and extracted with ethyl acetate. Then, the solvent was evaporated under vacuum and the reaction was purified by chromatography on silica gel with hexane/EtOAc 4:6 to yield the product as a yellowish solid. Yield: 57% (10 mg). *R*_f_ = 0.37 (DCM/MeOH 95:5).

^1^H NMR (700 MHz, DMSO-*d*_6_) δ 8.11 (d, *J* = 8.0 Hz, 1H), 7.77 (s, 1H), 7.41 (s, 1H), 7.20 (d, *J* = 7.3 Hz, 1H), 7.17 (t, *J* = 7.4 Hz, 1H), 7.02 (t, *J* = 7.4 Hz, 1H), 4.81 (s, 1H), 2.73 (dt, *J* = 12.7, 7.5 Hz, 2H), 2.62–2.58 (m, 1H), 2.37 (t, *J* = 8.0 Hz, 1H), 1.34 (d, *J* = 22.8 Hz, 6H), 1.07 (d, *J* = 6.1 Hz, 3H). ^13^C NMR (176 MHz, DMSO) δ: 173.4, 170.6, 141.9, 140.1, 127.1, 123.5, 121.8, 116.2, 72.4, 43.1, 42.1, 32.0, 28.1, 22.3, 17.2.

[α]_D_^20^ = −178 (*c* 0.3, MeOH).

MS (ESI) *m*/*z* calc. [M + Na]^+^ C_15_H_21_N_2_O_2_S^+^: 315.12455; found: 315.11267.

#### 4.1.10 ((*S*)-3-((*R*)-2-Carbamoyl-3,3-dimethylindolin-1-yl)-2-methyl-3-oxopropyl)­ethanethioate ((*S*,*R*′)-**14a**)

According to procedure C, (*S*,*R*′)-**13a** (65 mg, 0.11 mmol) was dissolved in DCM/TFA 1:1 (4 mL/4 mL) and the reaction was stirred overnight at room temperature. The mixture was washed with NaHCO_3_ and extracted with DCM. Then, the solvent was evaporated under vacuum and the reaction was purified by chromatography on silica gel with hexane/EtOAc (6:4) to yield the product as a yellowish solid. Yield:80% (29 mg). *R*_f_ = 0.18 (H/E 4:6).

^1^H NMR (700 MHz, DMSO-*d*_6_) δ 8.05 (d, *J* = 8.0 Hz, 1H), 7.69 (s, 1H), 7.29 (s, 1H), 7.19–7.14 (m, 2H), 7.01 (d, *J* = 7.3 Hz, 1H), 4.51 (s, 1H), 3.02 (dd, *J* = 13.2, 5.6 Hz, 1H), 2.96 (dd, *J* = 13.2, 8.3 Hz, 1H), 2.67 (dq, *J* = 13.7, 6.8 Hz, 1H), 2.30 (s, 3H), 1.33 (s, 3H), 1.28 (s, 3H), 1.20 (d, *J* = 6.9 Hz, 3H). ^13^C NMR (176 MHz, DMSO-*d*_6_) δ: 195.6, 173.4, 170.6, 142.4, 140.4, 127.7, 124.1, 122.2, 116.6, 72.3, 43.6, 39.1, 32.30, 31.7, 31.0, 22.5, 18.2.

MS (ESI) *m*/*z* calc. [M + H]^+^ C_17_H_22_N_2_O_3_S^+^: 335.14; found: 335.14.

#### 4.1.11 (*R*)-1-((*S*)-3-Mercapto-2-methylpropanoyl)-3,3-dimethylindoline-2-carboxamide (**6b**)

According to procedure D, (*S*,*R*′)-**14a** (29 mg, 0.09 mmol) was dissolved in THF/NaOH (1:3, 0.5 mL/1.5 mL) and the reaction was stirred for 2 h at room temperature. The mixture was quenched with 2 N HCl and extracted with ethyl acetate. Then, the solvent was evaporated under vacuum and the reaction was purified by chromatography on silica gel with hexane/EtOAc 4:6 to yield the product as a yellowish solid. Yield: 33% (8.6 mg). *R*_f_ = 0.23 (H/E 2:8).

^1^H NMR (700 MHz, DMSO-*d*_6_) δ 8.07 (d, *J* = 8.0 Hz, 1H), 7.77 (s, 1H), 7.41 (s, 1H), 7.18 (d, *J* = 7.3 Hz, 1H), 7.17–7.14 (m, 1H), 7.03–7.00 (m, 1H), 4.57 (s, 1H), 2.76 (dt, *J* = 13.6, 7.0 Hz, 2H), 2.61–2.58 (m, 1H), 2.16–2.12 (m, 1H), 1.32 (d, *J* = 37.0 Hz, 6H), 1.20 (d, *J* = 6.7 Hz, 3H). ^13^C NMR (176 MHz, DMSO-*d*_6_) δ: 173.2, 170.5, 142.0, 139.9, 127.2, 123.6, 116.1, 71.9, 43.2, 42.2, 31.9, 30.7, 26.8, 22.1, 17.1.

[α]_D_^20^ = −52.64 (*c* 0.3, MeOH).

MS (ESI) *m*/*z* calc. [M + H]^+^ C_15_H_21_N_2_O_2_S^+^: 293.12; found: 293.13.

#### 4.1.12 ((2*S*)-3-(3,3-Diethyl-2-(tritylcarbamoyl)­indolin-1-yl)-2-methyl-3-oxopropyl)­ethanethioate (**13b**)

According to general procedure A, a mixture of 3,3-diethyl-3*H*-indole **10b**, (100 mg, 0.69 mmol), 3-(acetylthio)-2-methylpropanoic acid **12** (70 μ, 0.76 mmol), and trityl isocyanide **11** (205 mg, 0.76 mmol) was dissolved in DCM (5 mL) in a sealed tube at 50 °C for 30 h. The crude product was purified by chromatography on silica gel with hexane/EtOAc (85:15) to yield the diastereomeric mixture. The racemic products were subjected to HPLC with MeOH/H_2_O (85:15) as the eluent (flow rate 3.00 mL/min) to obtain pure diastereomers. Total yield: 47% (dr 1:1).

**(** ***S***, ***S′*** **)-13b**. Orange solid; *R*_f_ = 0.4 (H/E 7:3). ^1^H NMR (700 MHz, DMSO-*d*_6_) δ 9.01 (s, 1H), 7.93–7.88 (m, 1H), 7.24 (m, 16H), 6.98–6.91 (m, 3H), 5.07 (d, *J* = 8.3 Hz, 1H), 3.18 (dd, *J* = 9.4, 5.2 Hz, 2H), 3.09 (d, *J* = 7.0 Hz, 1H), 2.20 (dd, *J* = 31.3, 9.1 Hz, 4H), 1.34–1.22 (m, 6H), 0.74 (dd, *J* = 33.6, 7.4 Hz, 3H). ^13^C NMR (176 MHz, DMSO-*d*_6_) δ 195.7, 172.8, 168.0, 144.8 (2), 144.5 (2), 129.1 (6 C), 128.8 (6 C), 127.9 (3 C), 127.7, 126.9, 126.8, 123.5, 117.2, 70.2, 70.1, 51.9, 31.5, 31.0, 29.8, 21.8, 18.5, 17.8, 10.0, 7.7.

tR HPLC: 20.83 min.

MS (ESI) *m*/*z* calc. [M + H]^+^ C_38_H_40_N_2_O_3_S^+^ 605.27596; found: 605.28387.

**(** ***S***, ***R*** **′)-13b**. Orange solid; *R*_f_ = 0.4 (H/E 7:3). ^1^H NMR (700 MHz, DMSO-*d*_6_) δ 8.93 (s, 1H), 7.92 (d, *J* = 7.9 Hz, 1H), 7.32–7.25 (m, 8H), 7.22 (m, 7H), 7.12–7.09 (t, 1H), 7.06 (d, *J* = 7.2 Hz, 1H), 6.97–6.93 (t, 1H), 5.19 (s, 1H), 3.06 (d, *J* = 6.5 Hz, 2H), 2.94 (dq, *J* = 13.9, 7.5, 6.7 Hz, 1H), 2.33 (s, 3H), 1.74 (tt, *J* = 14.1, 7.1 Hz, 2H), 1.56–1.49 (m, 2H), 0.93 (dd, *J* = 16.9, 9.6 Hz, 4H), 0.67 (t, *J* = 7.2 Hz, 3H). ^13^C NMR (176 MHz, DMSO-*d*_6_) δ: 199.7 172.4, 168.5, 144.6 (4), 129.15, 128.79 (6), 128.1 (6), 127.8 (3), 127.1, 123.6, 123.5, 117.2, 71.4, 70.42, 52.2, 32.9, 31.0, 30.7, 23.4, 17.1, 10.2, 8.1.

tR HPLC: 17.86 min.

#### 4.1.13 ((*S*)-3-((*S*)-2-Carbamoyl-3,3-diethylindolin-1-yl)-2-methyl-3-oxopropyl)­ethanethioate ((*S*,*S*′)-**14b**)

According to procedure C, (*S*,*S*′)-**13b** (100 mg, 0.18 mmol) was dissolved in DCM/TFA 1:1 (4 mL/4 mL) and the reaction mixture was stirred overnight at room temperature. The mixture was washed with NaHCO_3_ and extracted with DCM. Then, the solvent was evaporated under vacuum and the reaction was purified by chromatography on silica gel with hexane/EtOAc (6:4) to yield the product as a yellowish solid. Yield: 86% (56 mg). *R*_f_ = 0.28 (H/E 6:4).

^1^H NMR (700 MHz, DMSO-*d*_6_) δ 8.10 (d, *J* = 7.9 Hz, 1H), 7.79 (s, 1H), 7.43 (s, 1H), 7.16 (t, *J* = 7.6 Hz, 1H), 7.12 (d, *J* = 7.3 Hz, 1H), 7.02 (t, *J* = 7.3 Hz, 1H), 4.64 (s, 1H), 3.01 (d, *J* = 7.0 Hz, 2H), 2.81 (q, *J* = 6.6 Hz, 1H), 2.31 (s, 3H), 1.93 (dq, *J* = 14.4, 6.9 Hz, 1H), 1.71 (dt, *J* = 14.6, 7.0 Hz, 1H), 1.62 (ddt, *J* = 20.5, 13.7, 6.8 Hz, 2H), 1.09 (d, *J* = 6.3 Hz, 3H), 0.92 (t, *J* = 7.3 Hz, 3H), 0.68 (t, *J* = 7.1 Hz, 3H). ^13^C NMR (176 MHz, DMSO-*d*_6_) δ: 194.9, 172.9, 170.6, 143.0, 136.9, 127.2, 123.3, 123.2, 116.3, 70.4, 50.6, 38.4, 33.2, 32.6, 30.5, 25.4, 17.3, 9.3, 7.8.

#### 4.1.14 (*S*)-3,3-Diethyl-1-((*S*)-3-mercapto-2-methylpropanoyl)­indoline-2-carboxamide (**6c**)

According to procedure D, (*S*,*S*′)-**14b** (48 mg, 0.13 mmol) was dissolved in THF/NaOH (1:3, 0.5 mL/1.5 mL) and the reaction was stirred for 2 h at room temperature. The mixture was quenched with 2 N HCl and extracted with ethyl acetate. Then, the solvent was evaporated under vacuum and the reaction was purified by chromatography on silica gel with hexane/EtOAc 1:1 to yield the product as a yellowish solid. Yield: 32% (8.6 mg). *R*_f_ = 0.41 (H/E 4:6).

^1^H NMR (700 MHz, DMSO-*d*_6_) δ 8.12 (d, *J* = 8.0 Hz, 1H), 7.77 (s, 1H), 7.47 (s, 1H), 7.16 (t, *J* = 7.7 Hz, 1H), 7.11 (d, *J* = 7.3 Hz, 1H), 7.01 (t, *J* = 7.3 Hz, 1H), 4.82 (s, 1H), 2.83 (dq, *J* = 12.8, 6.4 Hz, 1H), 2.70 (dt, *J* = 12.7, 9.0 Hz, 1H), 2.58 (ddd, *J* = 12.9, 7.5, 4.6 Hz, 1H), 2.32 (t, *J* = 8.3 Hz, 1H), 1.94 (dt, *J* = 14.6, 7.3 Hz, 1H), 1.70 (dq, *J* = 14.7, 7.3 Hz, 1H), 1.64 (q, *J* = 7.2 Hz, 2H), 1.06 (d, *J* = 6.6 Hz, 3H), 0.96 (t, *J* = 7.4 Hz, 3H), 0.68 (t, *J* = 7.3 Hz, 3H). ^13^C NMR (176 MHz, DMSO-*d*_6_) δ: 173.4, 170.8, 143.1, 137.2, 123.3, 123.0, 116.3, 70.6, 50.6, 42.1, 32.8, 30.7, 28.1, 25.1, 17.5, 9.4, 7.9.

MS (ESI) *m*/*z* calc. [M + H]^+^ C_17_H_25_N_2_O_2_S^+^: 321.16; found: 321.16.

#### 4.1.15 ((*S*)-3-((*R*)-2-Carbamoyl-3,3-diethylindolin-1-yl)-2-methyl-3-oxopropyl)­ethanethioate ((*S*,*R*′)-**14b**)

According to procedure C, (*S*,*R*′)-**13b** (95 mg, 0.17 mmol) was dissolved in DCM/TFA 1:1 (4 mL/4 mL) and the reaction was stirred overnight at room temperature. The mixture was washed with NaHCO_3_ and extracted with DCM. Then, the solvent was evaporated under vacuum and the reaction was purified by chromatography on silica gel with hexane/EtOAc (6:4) to yield the product as a yellowish solid. Yield: 65% (35 mg). *R*_f_ = 0.28 (H/E 6:4).

^1^H NMR (700 MHz, DMSO-*d*_6_) δ 8.12 (d, *J* = 8.0 Hz, 1H), 7.78 (s, 1H), 7.39 (s, 1H), 7.23 (t, *J* = 7.6 Hz, 1H), 7.17 (d, *J* = 7.3 Hz, 1H), 7.07 (t, *J* = 7.4 Hz, 1H), 4.61 (s, 1H), 3.08 (dd, *J* = 13.1, 5.5 Hz, 1H), 3.02 (dd, *J* = 13.2, 8.4 Hz, 1H), 2.82 (dq, *J* = 13.6, 6.7 Hz, 1H), 2.00 (tt, *J* = 10.8, 5.5 Hz, 1H), 1.76–1.67 (m, 2H), 1.59 (dq, *J* = 14.5, 7.3 Hz, 1H), 1.25 (d, *J* = 6.8 Hz, 3H), 1.03 (t, *J* = 7.4 Hz, 3H), 0.71 (t, *J* = 7.3 Hz, 3H). ^13^C NMR (176 MHz, DMSO-*d*_6_) δ 195.6, 173.2, 170.7, 143.4, 137.6, 128.2, 123.8, 123.6 116.7, 70.8, 51.0, 38.9, 32.8, 31.8, 30.9, 24.8, 18.2, 9.8, 8.2.

#### 4.1.16 (*R*)-3,3-Diethyl-1-((*S*)-3-mercapto-2-methylpropanoyl)­indoline-2-carboxamide (**6d**)

According to procedure D, (*S*,*R*′)-**14b** (35 mg, 0.10 mmol) was dissolved in THF/NaOH (1:3, 0.5 mL/1.5 mL) and the reaction was stirred for 2 h at room temperature. The mixture was quenched with 2 N HCl and extracted with ethyl acetate. Then, the solvent was evaporated under vacuum and the reaction was purified by chromatography on silica gel with hexane/EtOAc 1:1 to yield the product as a yellow solid. Yield: 21% (9 mg). *R*_f_ = 0.41 (H/E 4:6).

^1^H NMR (700 MHz, DMSO-*d*_6_) δ 8.08 (d, *J* = 8.0 Hz, 1H), 7.79 (s, 1H), 7.45 (s, 1H), 7.16 (t, *J* = 7.5 Hz, 1H), 7.11 (d, *J* = 7.3 Hz, 1H), 7.01 (t, *J* = 7.3 Hz, 1H), 4.60 (s, 1H), 2.75 (dt, *J* = 13.6, 7.2 Hz, 1H), 2.69 (dq, *J* = 12.7, 6.2 Hz, 1H), 2.47 (dd, *J* = 13.2, 7.2 Hz, 1H), 2.12 (t, *J* = 8.1 Hz, 1H), 1.95 (dt, *J* = 14.4, 7.2 Hz, 1H), 1.66 (ddt, *J* = 27.6, 14.0, 7.1 Hz, 3H), 1.56 (dq, *J* = 14.3, 7.3 Hz, 1H), 1.19 (d, *J* = 6.6 Hz, 3H), 0.97 (t, *J* = 7.3 Hz, 3H), 0.65 (t, *J* = 7.2 Hz, 3H). ^13^C NMR (176 MHz, DMSO-*d*_6_) δ: 173.5, 171.2, 143.5, 137.6, 127.6, 123.8, 123.5, 116.8, 70.9, 51.0, 42.5, 32.9, 27.5, 25.0, 17.5, 9.8, 8.2.

MS (ESI) *m*/*z* calc. [M + H]^+^ C_17_H_25_N_2_O_2_S^+^: 321.16; found: 321.16.

#### 4.1.17 ((2*S*)-2-Methyl-3-oxo-3-(2′-(tritylcarbamoyl)­spiro­[cyclohexane-1,3′-indolin]-1′-yl)­propyl)­ethanethioate (**13c**)

Chemical procedures and spectral data for (*S*,*R*′)-**13c**, (*S*,*S*′)-**13c** are reported in our previously published paper.^39^

#### 4.1.18 ((*S*)-3-((*S*)-2′-Carbamoylspiro­[cyclohexane-1,3′-indolin]-1′-yl)-2-methyl-3-oxopropyl)­ethanethioate ((*S*,*S′*)-**14c**)

According to procedure C, (*S*,*S*′)-**13c** (215 mg, 0.34 mmol) was dissolved in DCM/TFA 1:1 (4.5 mL/4.5 mL) and the reaction mixture was stirred overnight at room temperature. The mixture was washed with NaHCO_3_ and extracted with DCM. Then, the solvent was evaporated under vacuum, and the reaction was purified by chromatography on silica gel with hexane/EtOAc (6:4) to yield the product as a yellow solid. Yield: 78% (95 mg). *R*_f_ = 0.19 (H/E 6:4).

^1^H NMR (700 MHz, DMSO-*d*_6_) δ 8.21 (d, *J* = 7.9 Hz, 1H), 8.06 (s, 1H), 7.59 (s, 1H), 7.38 (d, *J* = 7.4 Hz, 1H), 7.33 (t, *J* = 7.6 Hz, 1H), 7.20 (t, *J* = 7.4 Hz, 1H), 5.01 (s, 1H), 3.28–3.23 (m, 1H), 3.20 (q, *J* = 9.4, 8.0 Hz, 2H), 2.50 (s, 3H), 1.84 (m, 8H), 1.56–1.46 (m, 2H), 1.29 (d, *J* = 6.0 Hz, 3H). ^13^C NMR (176 MHz, DMSO-*d*_6_) δ: 194.8, 172.7, 170.1, 142.1, 140.8, 127.0, 123.6, 121.9, 116.5, 69.4, 47.4, 37.9, 32.5, 30.5, 29.5, 25.1, 22.7, 21.9, 17.4 (2).

#### 4.1.19 (*S*)-1′-((*S*)-3-Mercapto-2-methylpropanoyl)­spiro­[cyclohexane-1,3′-indoline]-2′-carboxamide (**6e**)

According to procedure D, (*S*,*S*′)-**14c** (35 mg, 0.10 mmol) was dissolved in THF/NaOH (1:3, 0.5 mL/1.5 mL) and the reaction was stirred for 2 h at room temperature. The mixture was quenched with 2 N HCl and extracted with ethyl acetate. Then, the solvent was evaporated under vacuum and the reaction was purified by chromatography on silica gel with hexane/EtOAc 6:4 to yield the product as a yellow solid. Yield: 38% (25 mg). *R*_f_ = 0.56 (H/E 4:6).

^1^H NMR (700 MHz, DMSO-*d*_6_) δ 8.11 (d, *J* = 7.9 Hz, 1H), 7.93 (s, 1H), 7.50 (s, 1H), 7.26 (d, *J* = 7.3 Hz, 1H), 7.20 (t, *J* = 7.7 Hz, 1H), 7.06 (t, *J* = 7.7 Hz, 1H), 5.05 (s, 1H), 3.11–3.05 (m, 1H), 2.76 (dt, *J* = 13.0, 9.3 Hz, 1H), 2.67 (ddd, *J* = 12.9, 7.8, 5.0 Hz, 1H), 2.42 (t, *J* = 8.4 Hz, 1H), 1.98–1.91 (m, 1H), 1.86–1.80 (m, 2H), 1.79–1.70 (m, 4H), 1.62 (dt, *J* = 13.1, 3.5 Hz, 2H), 1.41–1.34 (m, 1H), 1.14 (d, *J* = 6.6 Hz, 3H). ^13^C NMR (176 MHz, DMSO-*d*_6_) δ: 173.7, 170.8, 142.7, 141.4, 127.4, 123.9, 122.4, 116.9, 69.8, 47.9, 42.1, 30.1, 28.5, 25.5, 23.3, 22.4, 18.0 (2).

MS (ESI) *m*/*z* calc. [M + H]^+^ C_18_H_25_N_2_O_2_S^+^: 333.15987; found: 333.16309.

#### 4.1.20 ((*S*)-3-((*R*)-2′-Carbamoylspiro­[cyclohexane-1,3′-indolin]-1′-yl)-2-methyl-3-oxopropyl)­ethanethioate ((*S*,*R*′)-**14c**)

According to procedure C, (*S*,*R′*)-**13c** (200 mg, 0.325 mmol) was dissolved in DCM/TFA 1:1 (4.5 mL/4.5 mL) and the reaction was stirred overnight at room temperature. The mixture was washed with NaHCO_3_ and extracted with DCM. Then, the solvent was evaporated under vacuum and the reaction was purified by chromatography on silica gel with hexane/EtOAc (6:4) to yield the product as a yellowish solid. Yield: 60% (72 mg). *R*_f_ = 0.19 (H/E 6:4).

^1^H NMR (700 MHz, DMSO-*d*_6_) δ 7.99 (d, *J* = 7.9 Hz, 1H), 7.87 (s, 1H), 7.30 (s, 1H), 7.17 (d, *J* = 7.4 Hz, 1H), 7.13 (t, *J* = 7.7 Hz, 1H), 6.99 (t, *J* = 7.2 Hz, 1H), 4.72 (s, 1H), 3.03 (dd, *J* = 13.2, 5.5 Hz, 1H), 2.98 (dd, *J* = 13.1, 8.6 Hz, 1H), 2.89 (dq, *J* = 13.7, 6.8 Hz, 1H), 2.31 (s, 3H), 1.92–1.79 (m, 2H), 1.74 (d, *J* = 12.6 Hz, 2H), 1.70–1.61 (m, 4H), 1.55 (t, *J* = 14.2 Hz, 2H), 1.19 (d, *J* = 6.8 Hz, 3H). ^13^C NMR (176 MHz, DMSO-*d*_6_) δ 195.5, 173.1, 170.2, 142.7, 141.2, 127.4, 124.0, 122.3, 116.8, 69.0, 47.8, 39.2, 31.7, 30.9, 29.9, 25.6, 23.2, 22.2, 18.6 (2).

#### 4.1.21 (*R*)-1′-((*S*)-3-Mercapto-2-methylpropanoyl)­spiro­[cyclohexane-1,3′-indoline]-2′-carboxamide (**6f**)

According to procedure D, (*S*,*R*′)-**14c** (72 mg, 0.20 mmol) was dissolved in THF/NaOH (1:3, 1.2 mL/3.5 mL) and the reaction was stirred for 2 h at room temperature. The mixture was quenched with 2 N HCl and extracted with ethyl acetate. Then, the solvent was evaporated under vacuum and the reaction was purified by chromatography on silica gel with hexane/EtOAc 6:4 to yield the product as a yellowish solid. Yield: 33% (20 mg). *R*_f_ = 0.56 (H/E 4:6).

^1^H NMR (700 MHz, DMSO-*d*_6_) δ 8.08 (d, *J* = 8.0 Hz, 1H), 8.06 (d, *J* = 8.0 Hz, 1H), 8.03 (s, 1H), 8.01 (s, 1H), 7.52 (s, 1H), 7.48 (s, 1H), 7.24 (d, *J* = 7.3 Hz, 2H), 7.19 (t, *J* = 7.7 Hz, 2H), 7.06 (t, *J* = 7.3 Hz, 2H), 4.85 (s, 1H), 4.83 (s, 1H), 3.16 (dd, *J* = 12.8, 7.7 Hz, 1H), 3.14–3.08 (m, 1H), 2.90 (dq, *J* = 13.6, 6.8 Hz, 2H), 2.87–2.80 (m, 2H), 2.55–2.51 (m, 1H), 2.19 (t, *J* = 8.2 Hz, 1H), 1.94 (dt, *J* = 15.7, 8.4 Hz, 2H), 1.87–1.78 (m, 5H), 1.79–1.68 (m, 8H), 1.66–1.55 (m, 5H), 1.30 (d, *J* = 6.5 Hz, 3H), 1.26 (d, *J* = 6.7 Hz, 3H). ^13^C NMR (176 MHz, DMSO-*d*_6_) δ: 173.0, 170.1, 142.2, 140.7, 127.0, 123.5, 121.9, 116.4, 68.7, 47.4, 42.1, 29.5, 26.9, 25.1, 22.8, 21.9, 18.0, 17.5.

MS (ESI) *m*/*z* calc. [M + H]^+^ C_18_H_25_N_2_O_2_S^+^: 333.16; found: 333.16.

#### 4.1.22 Ethyl (1-((*S*)-3-(Acetylthio)-2-methylpropanoyl)-3,3-dimethylindoline-2-carbonyl)­glycinate ((*S,S*)*-*16a, (*S,R*)-16a)

According to general procedure A, a mixture of 3,3-dimethyl-3*H*-indole **10a**, (150 mg, 1.03 mmol), 3-(acetylthio)-2-methylpropanoic acid **12** (103 μL, 1.33 mmol), and ethyl isocyanoacetate **15** (124 μL, 1.33 mmol) was dissolved in DCM (5 mL) in a sealed tube at 50 °C for 30 h. The crude product was purified by chromatography on silica gel with a hexane/EtOAc (85:15) to yield the diastereomeric mixture. The racemic products were subjected to HPLC with MeOH/H_2_O (65:35) as the eluent (flow rate 3.00 mL/min) to provide the pure diastereomers. Total yield: 32% (dr 1:1).

tR HPLC: 22.35; tR HPLC: 24.36.

^1^H NMR (600 MHz, DMSO-*d*_6_) δ 8.78 (d, *J* = 26.8 Hz, 2H), 8.07 (t, *J* = 8.8 Hz, 2H), 7.22–7.14 (m, 4H), 7.07–6.99 (m, 2H), 4.72 (s, 1H), 4.63 (s, 1H), 4.11–4.07 (m, 4H), 3.87,(m, 4H), 3.04 (m, 3H), 2.96 (m, 3H), 2.34 (s, 6H), 1.36–1.29 (m, 12H), 1.20–1.18 (m, 6H), 1.12 (d, *J* = 7.1 Hz, 6H).

#### 4.1.23 ((*S*)-1-((*S*)-3-Mercapto-2-methylpropanoyl)-3,3-dimethylindoline-2-carbonyl)­glycine (**7a**)

According to procedure D, (*S*,*S*′)-**16a** (75 mg, 0.18 mmol) was dissolved in THF/NaOH (1:3, 1.2 mL/3.5 mL) and the reaction was stirred for 2 h at room temperature. The mixture was quenched with 2 N HCl and extracted with ethyl acetate. Then, the solvent was evaporated under vacuum and the reaction was purified by chromatography on silica gel with DCM/MeOH 9:1 to yield the product as a white solid. Yield: 37% (23 mg). *R*_f_ = 0.37 (DCM/MeOH 8:2 + formic acid).

^1^H NMR (700 MHz, DMSO-*d*_6_) δ 10.22 (s, 1H), 8.59 (s, 1H), 8.10 (d, *J* = 7.9 Hz, 1H), 7.20–7.14 (m, 2H), 7.02 (t, *J* = 7.5 Hz, 1H), 4.96 (s, 1H), 3.78–3.69 (m, 2H), 2.83–2.76 (m, 1H), 2.69 (dt, *J* = 18.3, 9.1 Hz, 1H), 2.58 (dt, *J* = 13.0, 6.7 Hz, 1H), 2.40 (t, *J* = 8.2 Hz, 1H), 1.32 (s, 3H), 1.24 (s, 3H), 1.02 (d, *J* = 6.5 Hz, 3H). ^13^C NMR (101 MHz, DMSO-*d*_6_) δ: 174.0, 168.9, 168.9, 142.42, 140.81, 127.60, 124.03, 122.25, 116.74, 72.74, 44.06, 42.47, 32.41, 29.46, 28.77, 22.56, 17.79.

[α]_D_^20^ = −89.34 (*c* 0.3, MeOH).

MS (ESI) *m*/*z* calc. [M + H]^+^ C_17_H_23_N_2_O_4_S^+^: 351.13003; found: 351.13635.

#### 4.1.24 ((*R*)-1-((*S*)-3-Mercapto-2-methylpropanoyl)-3,3-dimethylindoline-2-carbonyl)­glycine (**7b**)

According to procedure D, (*S*,*R*′)-**16a** (61 mg, 0.15 mmol) was dissolved in THF/NaOH (1:3, 1.0 mL/3.0 mL) and the reaction was stirred for 2 h at room temperature. The mixture was quenched with 2 N HCl and extracted with ethyl acetate. Then, the solvent was evaporated under vacuum and the reaction was purified by chromatography on silica gel with DCM/MeOH 9:1 to yield the product as a white solid. Yield: 98% (49 mg). *R*_f_ = 0.27 (DCM/MeOH 8:2 + formic acid).

^1^H NMR (700 MHz, DMSO-*d*_6_) δ 10.28 (s, 1H), 8.50 (s, 1H), 8.15–8.11 (m, 1H), 7.22 (dt, *J* = 14.6, 7.1 Hz, 2H), 7.10–7.05 (m, 1H), 4.79 (s, 1H), 3.79–3.67 (m, 2H), 3.06 (dd, *J* = 13.1, 6.4 Hz, 1H), 2.84–2.75 (m, 2H), 2.73 (dt, *J* = 13.6, 7.0 Hz, 2H), 1.37 (t, *J* = 7.6 Hz, 6H), 1.31–1.26 (m, 3H). ^13^C NMR (176 MHz, DMSO-*d*_6_) δ: 174.0, 168.5, 168.5, 142.4, 140.5, 127.6, 124.0, 122.2, 116.6, 72.4, 55.4, 49.0, 42.7, 32.3, 27.5, 22.4, 17.5.

[α]_D_^20^ = −26.32 (*c* 0.3, MeOH).

MS (ESI) *m*/*z* calc. [M + H]^+^ C_17_H_23_N_2_O_4_S^+^: 351.13004; found: 351.13678.

#### 4.1.25 Ethyl (1-((*S*)-3-(Acetylthio)-2-methylpropanoyl)-3,3-diethylindoline-2-carbonyl)­glycinate (**16b**)

According to general procedure A, a mixture of 3,3-diethyl-3*H*-indole **10b**, (320 mg, 1.85 mmol), 3-(acetylthio)-2-methylpropanoic acid **12** (186 μL, 2.04 mmol), and ethyl isocyanoacetate **15** (223 μL, 2.04 mmol) was dissolved in DCM (5 mL) in a sealed tube at 50 °C for 30 h. The crude product was purified by chromatography on silica gel with hexane/EtOAc (9:1) to yield the diastereomeric mixture. The racemic products were subjected to HPLC with MeOH/H_2_O (85:15) as the eluent (flow rate 3.00 mL/min) to obtain the pure diastereomers. Total yield: 23% (dr 1:1).

**(** ***S***, ***S*** **)-16b**. Yellow solid; *R*_f_ = 0.28 (H/E 7:3).

^1^H NMR (400 MHz, DMSO-*d*_6_) δ 8.75 (s, 1H), 8.06 (d, J = 7.9 Hz, 1H), 7.22–7.09 (m, 2H), 7.02 (t, *J* = 7.6 Hz, 1H), 4.68 (s, 1H), 4.09 (q, *J* = 7.1 Hz, 2H), 3.91 (dd, *J* = 17.4, 5.5 Hz, 1H), 3.78 (dd, *J* = 17.4, 5.6 Hz, 1H), 2.97 (d, *J* = 6.9 Hz, 2H), 2.74 (q, *J* = 6.7 Hz, 1H), 2.30 (s, 3H), 1.61 (ddq, *J* = 49.5, 14.6, 6.8 Hz, 4H), 1.19 (t, *J* = 6.6 Hz, 6H), 0.94 (t, *J* = 7.2 Hz, 3H), 0.66 (t, *J* = 7.2 Hz, 3H). ^13^C ^13^C NMR (101 MHz, DMSO) δ 195.3, 172.8, 169.2, 168.9, 142.8, 137.1, 127.2, 123.3, 123.2, 116.3, 70.2, 60.5, 50.9, 40.8, 38.2, 32.1, 31.4, 30.4, 24.2, 17.4, 14.0, 9.23, 7.7.

**(** ***S***, ***R*** **)-16b**. Yellow solid; *R*_f_ = 0.28 (H/E 7:3).

^1^H NMR (400 MHz, DMSO-*d*_6_) δ 8.79 (s, 1H), 8.09 (d, *J* = 8.1 Hz, 1H), 7.18 (t, *J* = 7.6 Hz, 1H), 7.13 (d, *J* = 7.3 Hz, 1H), 7.03 (t, *J* = 7.5 Hz, 1H), 4.78 (s, 1H), 4.09 (dtt, *J* = 10.8, 7.3, 3.7 Hz, 2H), 3.86 (t, *J* = 5.4 Hz, 2H), 3.01 (d, *J* = 6.8 Hz, 2H), 2.85 (dq, *J* = 13.6, 6.5 Hz, 1H), 2.32 (s, 3H), 1.91 (dq, *J* = 14.9, 7.4 Hz, 1H), 1.71–1.59 (m, 3H), 1.18 (t, *J* = 7.1 Hz, 3H), 1.05 (d, *J* = 6.5 Hz, 3H), 0.89 (t, *J* = 7.4 Hz, 3H), 0.70 (t, *J* = 7.2 Hz, 3H). ^13^C NMR (101 MHz, DMSO) δ: 195.3, 173.4, 169.8, 169.6, 143.3, 137.5, 127.7, 123.8, 123.8, 117.0, 70.9, 61.0, 51.5, 41.3, 38.7, 33.2, 33.1, 31.0, 25.3, 17.8, 14.5, 9.7, 8.3.

#### 4.1.26 ((*S*)-3,3-Diethyl-1-((S)-3-mercapto-2-methylpropanoyl)­indoline-2-carbonyl)­glycine (**7c**)

According to procedure D, (*S*,*S*′)-**16b** (50 mg, 0.11 mmol) was dissolved in THF/NaOH (1:3, 1.5 mL/4.5 mL) and the reaction was stirred for 2 h at room temperature. The mixture was quenched with 2 N HCl and extracted with ethyl acetate. Then, the solvent was evaporated under vacuum and the reaction was purified by chromatography on silica gel with DCM/MeOH 9:1 to yield the product as a yellow solid. Yield: 95% (40 mg). *R*_f_ = 0.32 (DCM/MeOH 8:2 + formic acid).

^1^H NMR (400 MHz, DMSO-*d*_6_) δ 8.48 (s, 1H), 8.12 (d, *J* = 7.9 Hz, 1H), 7.17 (t, *J* = 7.6 Hz, 1H), 7.11 (t, *J* = 7.5 Hz, 1H), 7.02 (t, *J* = 7.3 Hz, 1H), 5.00 (s, 1H), 3.71 (s, 2H), 2.69–2.62 (m, 1H), 2.57 (dd, *J* = 12.7, 7.3 Hz, 1H), 2.44–2.30 (m, 1H), 1.66 (tt, *J* = 14.4, 7.4 Hz, 4H), 1.01 (d, *J* = 6.4 Hz, 3H), 0.91 (d, *J* = 7.3 Hz, 3H), 0.69 (t, *J* = 7.3 Hz, 3H). ^13^C NMR (101 MHz, DMSO-*d*_6_) δ: 174.0, 169.2, 162.7, 143.4, 137.8, 127.5, 123.8, 123.5, 116.8, 71.01, 51.3, 36.7, 36.2, 32.9, 31.2, 25.2, 18.1, 9.6, 8.3.

MS (ESI) *m*/*z* calc. [M + H]^+^ C_19_H_27_N_2_O_4_S^+^: 379.16; found: 379.07.

#### 4.1.27 ((*R*)-3,3-Diethyl-1-((*S*)-3-mercapto-2-methylpropanoyl)­indoline-2-carbonyl)­glycine (**7d**)

According to procedure D, (*S*,*R*′)-**16b** (45 mg, 0.11 mmol) was dissolved in THF/NaOH (1:3, 1.5 mL/4.5 mL) and the reaction was stirred for 2 h at room temperature. The mixture was quenched with 2 N HCl and extracted with ethyl acetate. Then, the solvent was evaporated under vacuum and the reaction was purified by chromatography on silica gel with DCM/MeOH 9:1 to yield the product as a yellow solid. Yield: 97% (37 mg). *R*_f_ = 0.32 (DCM/MeOH 8:2 + formic acid).

^1^H NMR (400 MHz, DMSO-*d*_6_) δ 8.43 (s, 1H), 8.07 (d, *J* = 7.6 Hz, 1H), 7.20–7.13 (m, 1H), 7.11 (d, *J* = 6.6 Hz, 1H), 7.02 (d, *J* = 7.1 Hz, 1H), 4.82 (s, 1H), 3.73 (s, 2H), 2.99 (d, *J* = 9.6 Hz, 1H), 2.79–2.64 (m, 2H), 1.69–1.61 (m, 2H), 1.57 (dd, *J* = 13.9, 7.6 Hz, 2H), 1.20 (s, 3H), 0.94 (s, 4H), 0.66 (t, *J* = 6.8 Hz, 3H). ^13^C NMR (101 MHz, DMSO-*d*_6_) δ: 173.6, 169.4, 168.7, 143.4, 137.7, 127.5, 123.7, 123.5, 116.7, 70.8, 51.2, 32.7, 29.4, 27.6, 24.8, 22.5, 17.76, 9.72, 8.23.

MS (ESI) *m*/*z* calc. [M + H]^+^ C_19_H_27_N_2_O_4_S^+^: 379.16; found: 379.07.

#### 4.1.28 Stereochemical Characterization of Compound **14b**

The absolute stereochemistry of compound **14b** was investigated by a combination of experimental and computational CD spectroscopy, in accordance with well-established protocols.^44^

A preliminary exploration of the conformational space of (*S*,*S*′)-**14b** and (*S*,*R*′)-**14b** was achieved using the molecular mechanics (MM) methods implemented in RDKit.^45^ Conformers were generated with the ETKDG algorithm,^46^ using 2000 initial structures and 100 trial attempts. Energy minimization was performed with the MMFF94s force field,^47^ allowing up to 2000 iterations, and conformers were clustered based on a 0.1 Å RMSD threshold (heavy atoms only).

Conformers within a 20 kJ mol^–1^ energy window relative to the MM global minimum—80 for (*S*,*S*′)-**14b**, 70 for (*S*,*R*′)-**14b**—were subjected to DFT geometry optimizations and vibrational frequency calculations, which were carried out using the B97D3 functional,^48^ the def2-TZVP basis set,^49,50^ the density fitting approximation,^51,52^ and the IEFPCM solvent model for methanol.^53^ Conformers with imaginary frequencies or redundant geometries (RMSD < 0.01 Å for heavy atoms) were excluded from further analysis.

Subsequent TDDFT calculations were performed on the optimized geometries using the PBE0-1/3 functional,^54^ in combination with the def2-TZVPD basis set^49,55,56^ and IEFPCM solvation for methanol. For each structure, excitation energies (λ_*j*_), oscillator strengths (*f*_*j*_), and rotational strengths in the dipole length representation (*R*_*j*_) were computed for the lowest-energy 40 excited states.

The theoretical UV and CD spectra of **14b** stereoisomers were generated by applying Gaussian broadening (Δσ = 0.3 eV) to the computed *f*_*j*_ and *R*_*j*_ values, summing over all excited states, and averaging across conformers according to their Boltzmann-weighted populations derived from relative SCF energies (Δ*E*_SCF_); at this stage, only conformers contributing cumulatively to more than 90% of the total Boltzmann distribution were considered–11 for (*S*,*S*′)-**14b**, 12 for (*S*,*R*′)-**14b**. All DFT and TDDFT calculations were performed using the Gaussian 16 software package.^57^ Full results are provided in Tables S1–S5 in the Supporting Information.

The experimental UV and CD spectra of (*S*,*S*′)-**14b** and (*S*,*R*′)-**14b** were acquired on a Jasco J-715 spectropolarimeter, employing a Suprasil quartz cell (Hellma Analytics) with a 1 mm optical path length. Measurements were carried out on 0.1 mM solutions in methanol over the 350–200 nm spectral range; a spectral bandwidth of 1 nm, a scanning speed of 50 nm min^–1^, a data integration time of 2 s, a data pitch of 1 nm, and an accumulation cycle of 3 scans were used. The resulting spectra were solvent-corrected, converted to molar units, and compared to the theoretical spectra of **14b** stereoisomers (Figure 3).

### 4.2 Biological Assays

#### 4.2.1 Enzyme Assays

The inhibitory activity of the compounds was determined using enzyme assays with recombinant NDM-1, VIM-1, VIM-2, IMP-1, and IMP-7. These enzymes were produced in *E. coli* and purified as previously described.^43,58^ The fluorogenic substrate fluorocillin was synthesized according to the procedure described by Rukavishnikov et al.^43^ The assays were performed in 96-well format using black polystyrene plates (Corning, Corning, NY) at room temperature, and the measurements were carried out on the Tecan spectrophotometer Infinite F200 PRO (Tecan, Männedorf, Switzerland). The protein stock solutions were diluted in assay buffer (HEPES 50 mM, pH 7.5 containing 0.01%Triton X-100, buffer HX) to reach final concentrations of VIM-1–4 nM, IMP-7–0.1 nM; NDM-1–3 nM. Subsequently, 89 μL of the protein solution was mixed with 1 μL of the inhibitor diluted in DMSO (final DMSO concentration 1% at final assay volume of 100 μL) and incubated for 30 min. A 10 μL substrate solution in assay buffer (888 nM fluorocillin, HEPES 50 mM, pH 7.5; 0.01%Triton X-100) was added, and fluorescence increase (excitation at 495 nm and emission at 525 nm) was measured for 30 cycles of 1 min each. Negative controls were measured in the absence of enzyme (89 μL of assay buffer), whereas the positive controls were measured in the presence of enzyme and in the absence of inhibitors (1 μL of DMSO). Each measurement was performed in triplicate in three independent experiments. IC_50_ values were calculated using data obtained from measurements with at least eight different inhibitor concentrations, applying a sigmoidal dose–response (variable slope with four parameters) equation using GraphPad Prism 5 (GraphPad Software, La Jolla, CA) software. In contrast, the inhibitory activity was measured using a direct competition assay in which the rate of hydrolysis of 150 μM imipenem (as the reporter substrate, prepared extemporaneously, Merck cat. No. PHR1796) in buffer HX was measured in the absence and in the presence of various concentrations of the inhibitor. First, these assays were performed at a final concentration of the inhibitor in the reaction mixture of 50 μM, without preincubation, and the percentage of inhibition was computed as 100 × (1 – [*v_i_*/*v*_0_]), where *v_i_* and *v*_0_ are the rates of hydrolysis of the reporter substrate with and without the inhibitor, respectively. For compounds showing a significant inhibitory activity, the *K*_*i*_ values were determined using a competitive model of inhibition by measuring the rate of hydrolysis in the presence of varying concentrations of inhibitor, as previously described.^58^ All assays were performed in at least in triplicate. For inhibitors behaving as inactivators (i.e., showing a measurable decrease of the rate of hydrolysis of the reporter substrate during the reaction), the pseudo-first order inactivation rate (*k*_inact_) was measured and data processed as previously described [doi: 10.1021/bi971056h], by investigating the dependence of *k*_inact_ on the inhibitor concentration.

To assess the selectivity of the compounds, their inhibitory activity on human angiotensin-converting enzyme 1 (ACE-1) was performed using the ACE Activity Assay Kit (Sigma-Aldrich cat. No. CS0002). Briefly, the hydrolysis rate of an internally quenched fluorogenic substrate was determined in both the absence and presence of 50 μM inhibitor, in black 96-well plates (PerkinElmer Optiplate) and an Envision multitechnology plate reader (PerkinElmer, Whaltam) equipped with monochromators (excitation and emission wavelengths, 332 and 398 nm, respectively). Captopril (racemic mixture, d- and l-stereoisomers) was used as control in these experiments.

#### 4.2.2 Microbiological Assays

*In vitro* antimicrobial susceptibility testing was performed according to established standards. The synergistic activity of the compounds was investigated by measuring the minimal inhibitory concentrations (MICs) of imipenem monohydrate (Sigma-Aldrich, Steinheim, Germany) in the absence and presence of a fixed concentration (32 or 128 μg/mL) of the compounds on metallo-β-lactamase-producing clinical isolates using the broth microdilution method (Clinical and Laboratory Standards Institute) (CLSI). The strains used in the study were described elsewhere.^58^

#### 4.2.3 Growth Inhibition Assay

A computerized incubator (Tecan Infinite M200 pro, Crailsheim, Germany) was used to obtain the growth curves over a time course. Optical density (OD_600_) was measured for evaluating bacterial (i.e., *K. pneumoniae* NDM-1, *P. aeruginosa* IMP-1, *K. pneumoniae* VIM-1) density in suspension. Prior to each experiment, bacteria were cultured to the exponential phase (OD_600_ = 0.6–0.8) in cation-adjusted Mueller Hinton broth (Becton Dickinson, Heidelberg, Germany). 5 μL of diluted bacterial suspension and 100 μL of cation-adjusted Mueller Hinton broth either with or without imipenem (Arcos Organics, Schwerte, Germany) and/or the MBL inhibitor **6d** were added into wells of a 96-well plate to give a final bacterial inoculum of approximately 5 × 10^5^ CFU/mL. The temperature was adjusted to 37 °C. A permanent shaking speed of 450 rpm was used and was only interrupted before OD_600_ measurement. The optical densities of samples were recorded online every 10 min for 24 h.

### 4.3 Computational Studies

#### 4.3.1 Molecular Docking

The crystallographic structures of NDM-1 in complex with d-captopril at 1.10 Å resolution (PDB-ID: 5ZJ2),^34^ VIM-1 in complex with (**2**M)-4′-methyl-2-(2*H*-tetrazol-5-yl)­[1,1′-biphenyl]-3-sulfonamide at 1.13 Å resolution (PDB-ID: 7UP2),^59^ VIM-2 in complex with inhibitor MS19 at 1.60 Å resolution (PDB-ID: 6JN6),^60^ and IMP-1 in complex with (3-(4-(*p*-tolyl)-1*H*-1,2,3-triazol-1-yl)­benzyl)­phosphonic acid (PDB-ID: 7YHA)^61^ were retrieved from the Protein Data Bank (RCBS.org, PDB).^62^ The structure of *P. aeruginosa* IMP-7 is not available in PDB, and it was generated by homology modeling based on structural templates described in the literature (PDB-ID: 7YHA).^63^ The sequence of IMP-7 was retrieved from UniProtKB^64^ (ID: Q93AU3). The target (IMP-7) and the template sequences were aligned using the Clustal W method in Prime.^65,66^ The homology model of IMP-7 was finally built using Prime in the Schrodinger Suite 2022–4.^67–70^ The modeled 3D structure of IMP-7 was further minimized for 5000 iterations using Amber22,^71^ before being used as a receptor in docking simulations.

Small molecules **6c**, **6d**, and **6e** were sketched in 2D (Supporting Figures S3 and S4) with the Picto software version 4.5.4.1 (OpenEye Cadence Molecular Sciences, Santa Fe, NM)^72^ and converted into a 3D structure with OMEGA version 4.2.0.1 (OpenEye Cadence Molecular Sciences, Santa Fe, NM).^73,74^ The protonation state of the molecule was predicted at pH 7.4 using the p*K*_a_ prediction software MOKA,^75–77^ whereas the sulfhydryl group was considered as deprotonated to mimic Zn­(II) coordination. Ligand energy minimization was performed with SZYBKI version 2.5.0.1 (OpenEye Cadence Molecular Sciences, Santa Fe, NM)^78^ using the MMFF94S force field.^79^ SZMAP version 1.6.4.1. (OpenEye, Cadence Molecular Sciences, Santa Fe, NM)^80^ was used to assess the thermodynamic contribution of crystallographic water molecules, where available. Molecular docking was carried out with the GOLD program (The Cambridge Crystallographic Data Centre) version 2023.1.0 using the GOLDSCORE fitness function for docking and scoring purposes.^79,81^ The spherical binding site was centered within the two zinc atoms, and it had a radius of 14 Å. Water molecules identified by SZMAP were retained in the receptor structure during docking simulations. Ten docking runs for each ligand were stored and submitted to visual inspection.

#### 4.3.2 Molecular Dynamics

For MD purposes, the Zn­(II) coordination systems were prepared using a QM/MM strategy according to the MCPB.py approach.^82,83^ Accordingly, the Zn1 atom was bound to the three coordinating histidine residues, whereas the Zn2 atom was bound to histidine, cysteine, and aspartate. The ligand was modeled through a nonbonding approach. Ligand partial charges are assigned at the ami1-bcc level using antechamber.^84,85^ The ff19SB force field^86^ was used to parametrize the protein, and the general amber force field (GAFF)^87^ was used to parametrize the ligands. TIP3P-type water molecules and counterions were added to a rectangular box for solvating each complex with a buffer of 10 Å from the molecular system. MD simulations were run with Amber22.^71^ Briefly, for each system, the solvent was first energy minimized for 1500 steps using the steepest descent algorithm (SD), followed by 5000 steps with the conjugate gradient algorithm (CG) while the solute was frozen. The solvated solute was then energy minimized for 1500 steps with the SD and 5000 subsequent steps with the CG before being heated to 300 K for 1 ns at a constant volume using the Langevin thermostat. The system’s density was equilibrated for 1 ns by the Berendsen barostat at constant pressure, and then a preliminary equilibration of 50 ns was run before the final production of MD trajectories lasting 500 ns. The time step was 2 fs in all MD simulations. MD trajectories were analyzed by the CPPTRAJ software,^88^ and frame clustering was carried out with an agglomerative hierarchical algorithm.

## Supplementary Material







## Notes

Copies of ^1^ H and ^13^C NMR spectra; HRMS and LC-MS spectra; HPLC chromatograms; additional computational figures and 2D sketch presentations; and detailed results for the DFT and TDDFT computational study on the stereochemical characterization of compound **14b** (Tables S1–S5) (PDF)
Molecular formula strings (CSV)
XYZ structures of optimized conformers (provided as compressed folder) (ZIP)
