# Supplementary material for: Sustainable Joullié–Ugi and Continuous Flow Implementation Led to Novel Captopril-Inspired Broad-Spectrum Metallo-β-Lactamase Inhibitors
Source: J Med Chem. 2025 Aug 15;68(16):17236–57. doi: 10.1021/acs.jmedchem.5c00750 (PMC12406192; doi:10.1021/acs.jmedchem.5c00750)
Supplement: Supplementary file 1 [file jm5c00750_si_001.pdf]

SUPPLEMENTARY MATERIAL

Sustainable Joullié-Ugi and continuous flow implementation led to novel captopril-inspired broad-spectrum metallo-β-lactamase inhibitors

*Antonella Ilenia Alfano,[a] Sveva Pelliccia,[a,] Simona Barone,[a] Luigi Cutarella,[b] Sacha Michèle Idriss Cancade,[c] Valerio Baia,[a] Emilia Cassese,[a] Pasquale Russomanno,[d] Nicolò Messano,[b] Denia Frank,[e] Lilia Weizel,[f] Marco J. Rotter,[f] Steffen Brunst,[f] Thomas A. Wichelhaus,[e] Ewgenij Proschak,[f] Daniele Tedesco,[g] Mattia Mori,[b] Jean Denis Docquier,[c] Vincenzo Summa [a] and Margherita Brindisi,[a,]**

*To whom correspondence should be addressed. margherita.brindisi@unina.it

[a] Department of Pharmacy (Department of Excellence 2023-2027), University of Naples Federico II, via D. Montesano 49, 80131, Naples, Italy

[b] Department of Biotechnology, Chemistry and Pharmacy, University of Siena, via Aldo Moro 2, 53100, Siena, Italy.

[c] Department of Medical Biotechnologies, University of Siena, Viale Bracci 16, 53100 Siena, Italy

[d] Magnetic Resonance Centre (CERM), Consorzio Interuniversitario Risonanze Magnetiche di Metallo Proteine (CIRMMP) and Department of Chemistry "Ugo Schiff", University of Florence, Via L. Sacconi 6, Sesto Fiorentino, 50019, Italy

[e] Goethe University Frankfurt, University Hospital, Institute of Medical Microbiology and Infection Control, Paul-Ehrlich-Str. 40, 60596 Frankfurt am Main, Germany

[f] Institute of Pharmaceutical Chemistry, Goethe-University of Frankfurt, Max-von-Laue Str. 9, D-60438 Frankfurt am Main, Germany

[g] Institute for Organic Synthesis and Photoreactivity (ISOF), National Research Council of Italy (CNR), via P. Gobetti 101, 40129, Bologna, Italy.

Table of Contents

Figures S1 and S2………………………………………………………………. Page S3

Figures S3 and S4………………………………………………………………. Page S4

2D Sketch representation (Figures S5 and S6)……………………...……….. Page S5

^{1}H and ^{13}C NMR spectra ……………………………………………………………. Pages S6-S28

Stability studies for compound **7b**……………………………………………………. Page S29

HPLC chromatograms ………………………………………………….……… Page S30-S31

HRMS spectra ……………………………………………………………………. Pages S32-S42

Stereochemical characterization (Tables S1-S5) ……………………………… Pages S43-S49

References ……………………………………………………………………... Page S50

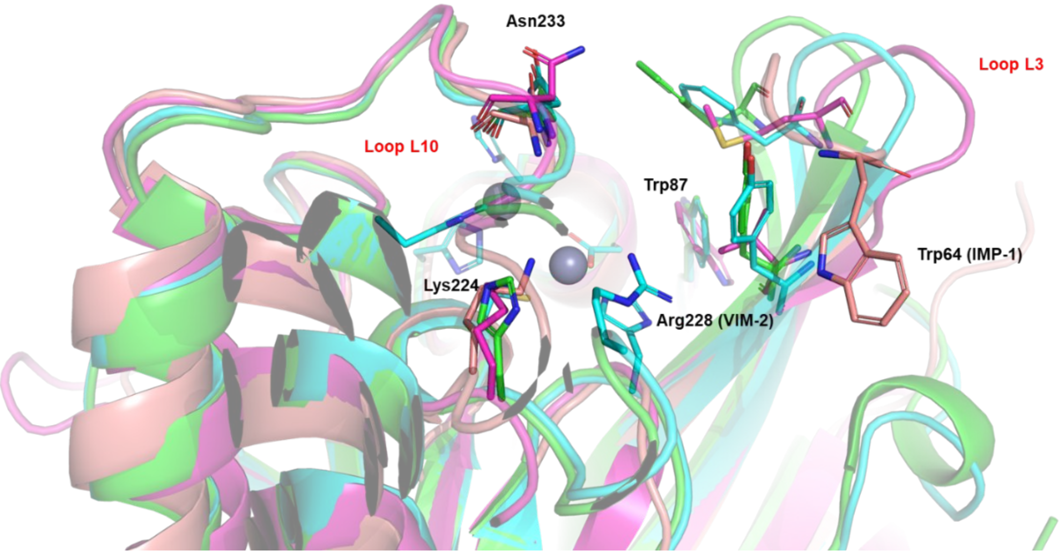

Figure S1. Comparison of the active site regions of IMP-1 (pink, PDB code 1DDK), VIM-1 (green, PSB code 5N5G), VIM-2 (cyan, PDB code 1KO3) and NDM-1 (magenta, PDB code 4EXS), showing the position of conserved residues, such as Trp87 or Asn233 (numbering according to the standard MBL numbering scheme), and the nature of the hydrophobic residues found in loop L3 (L1 in the standard numbering scheme).

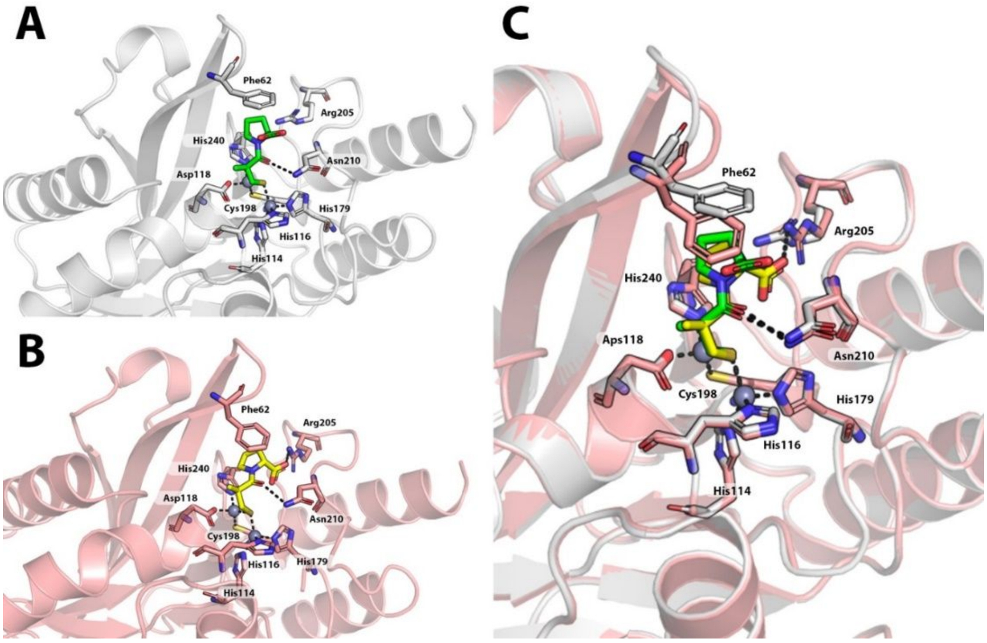

Figure S2. X-ray crystallography structure of VIM-2 in complex with L- and D-captopril. (A) X-ray crystallography complex of VIM-2 (grey cartoon and sticks) with L- captopril (green sticks) PDB-ID: 4C1D;[1] (B) X-ray crystallography complex of VIM-2 (dark pink cartoon and sticks) with D- captopril (yellow sticks) PDB-ID: 4C1E [ref];[1] (C) Superposition of VIM-2 structures in complex with L-captopril and D-captopril. Polar interactions are highlighted by black dashed lines. Residues involved in zinc coordination and in binding L- and D-captopril are shown as sticks and are labeled.

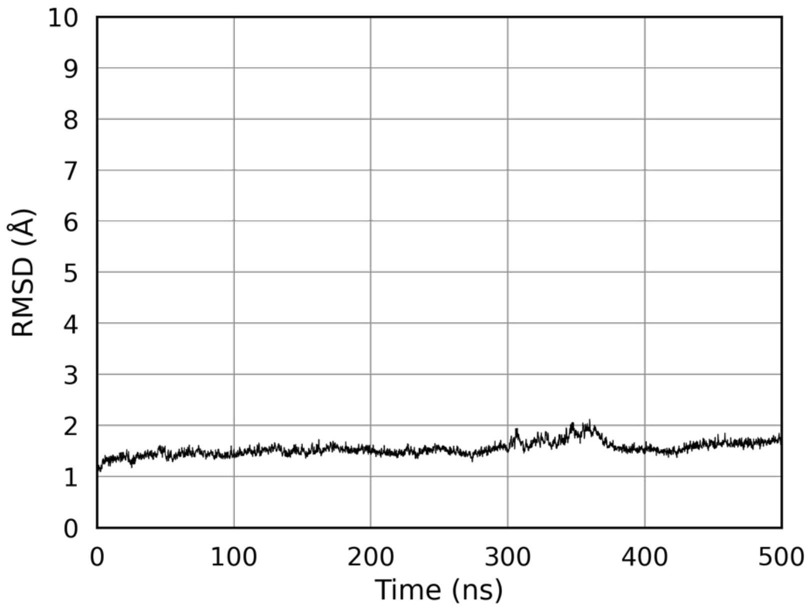

Figure S3. Root Mean Square Deviation (RMSD) plots of derivative **6d** with NMD-1.

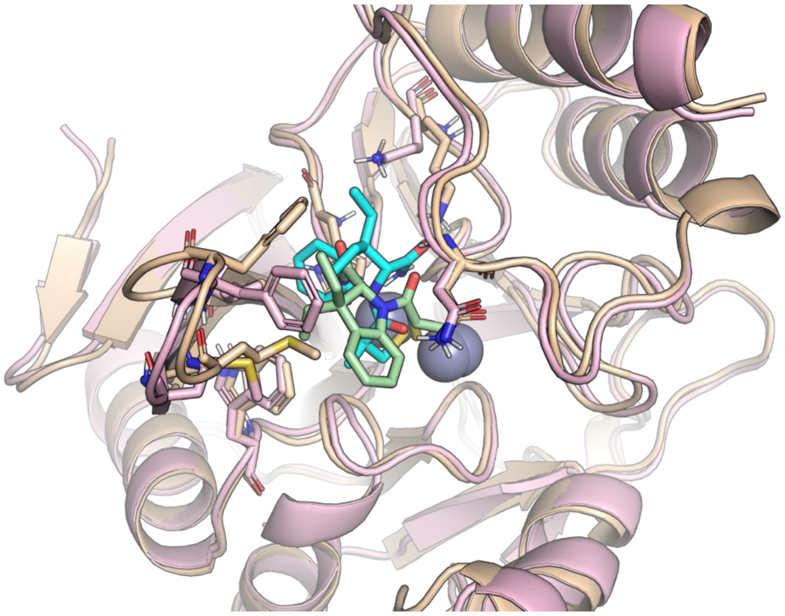

Figure S4. The overlap between the docking pose and the most representative frame of the MD trajectory obtained by clustering analysis of compound **6d**. The **6d** docking pose on NDM-1 (colored in beige) is represented as cyan sticks, while the most populated cluster pose of the **6d** MD trajectory on NDM-1 (colored in pink) is shown as light green sticks.

2D Sketch Representations

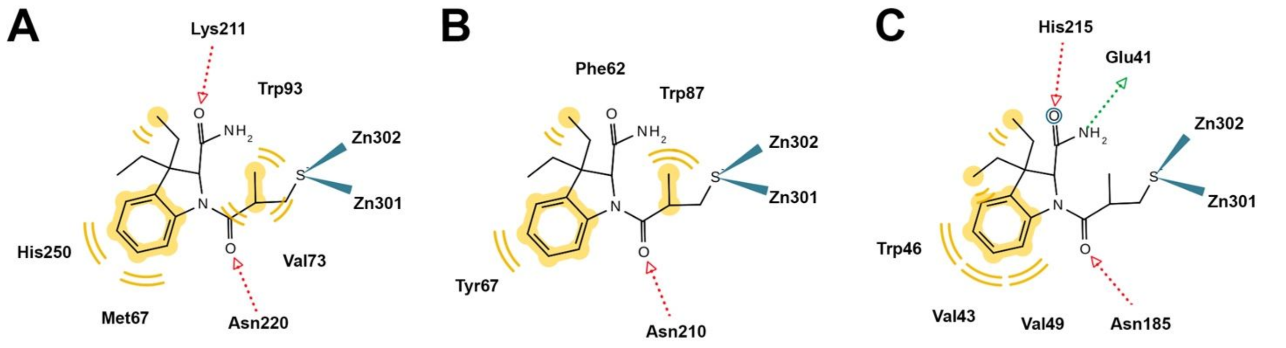

Figure S5. 2D representation of the pharmacophoric interactions between compound **6d** and NDM-1 (Panel A), VIM-1 (Panel B), and IMP-1 (Panel C). Red and green arrows indicate hydrogen bond interactions. The yellow-highlighted regions of the molecule denote hydrophobic interactions. The blue wedges represent the coordinating bonds to the catalytic zinc ions.

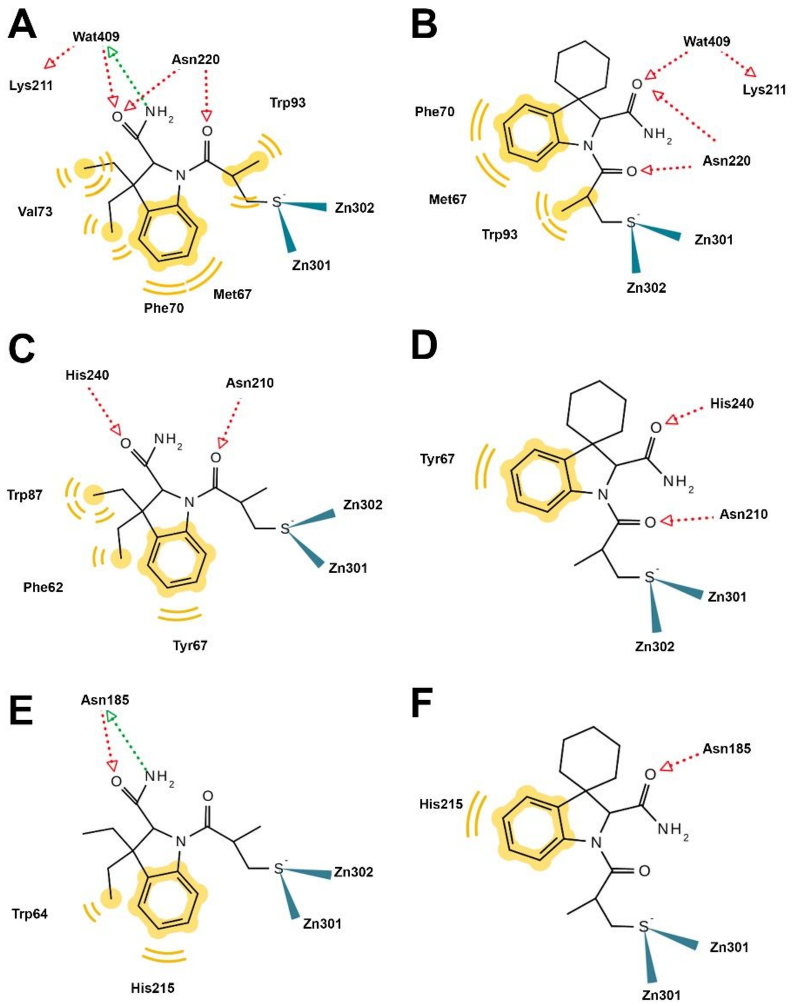

Figure S6. 2D representation of the pharmacophoric interactions between **6c** and **6e** and NDM-1 (Panel A and B respectively), VIM-1 (Panel C and D respectively) and IMP-1 (Panel E and F respectively). Red and green arrows indicate hydrogen bond interactions. The yellow-highlighted regions of the molecule denote hydrophobic interactions. The blue wedges represent the coordinating bonds to the catalytic zinc ions.

Characterization of compounds

^{1}H NMR of compound *(S,S')*-**13a**

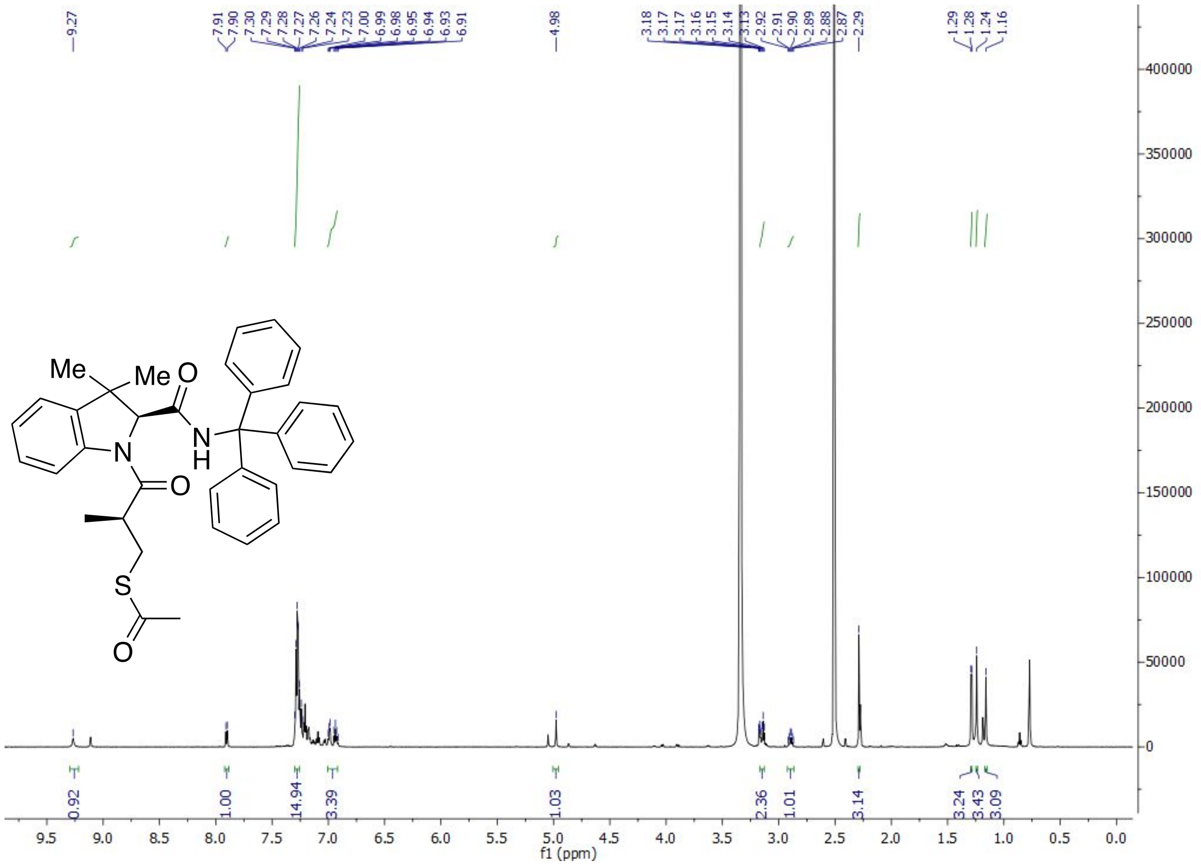

^{13}C NMR of compound *(S,S')*-**13a**

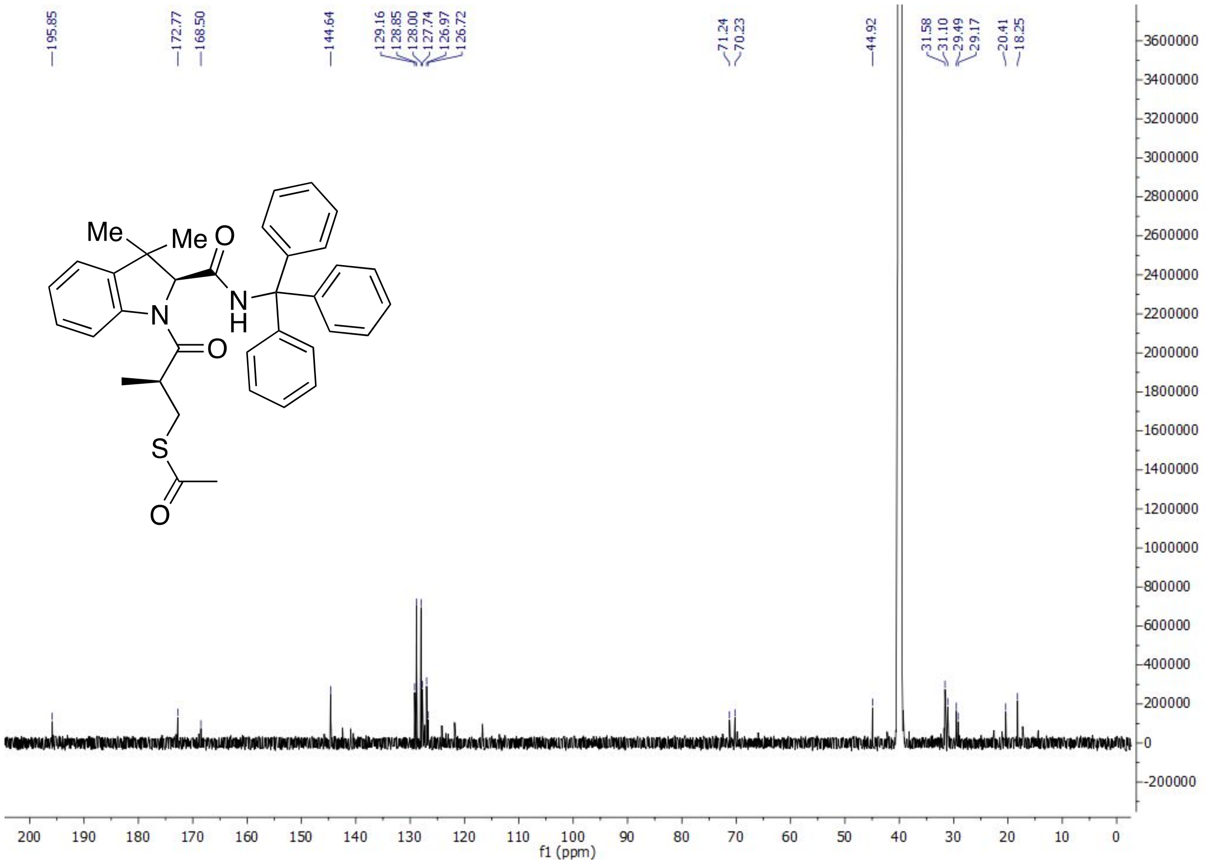

^{1}H NMR of compound *(S,R')*-**13a**

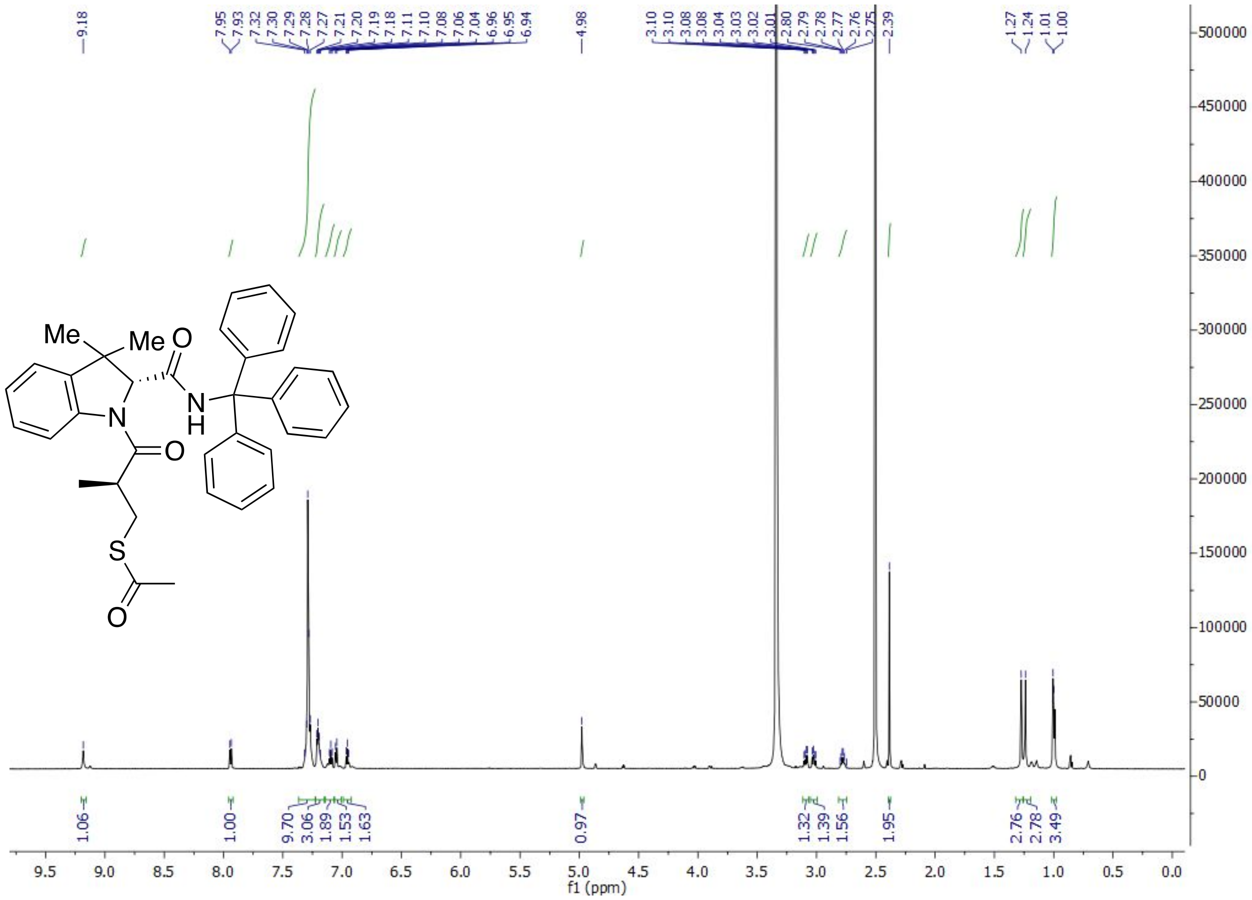

^{13}C NMR of compound *(S,R')*-**13a**

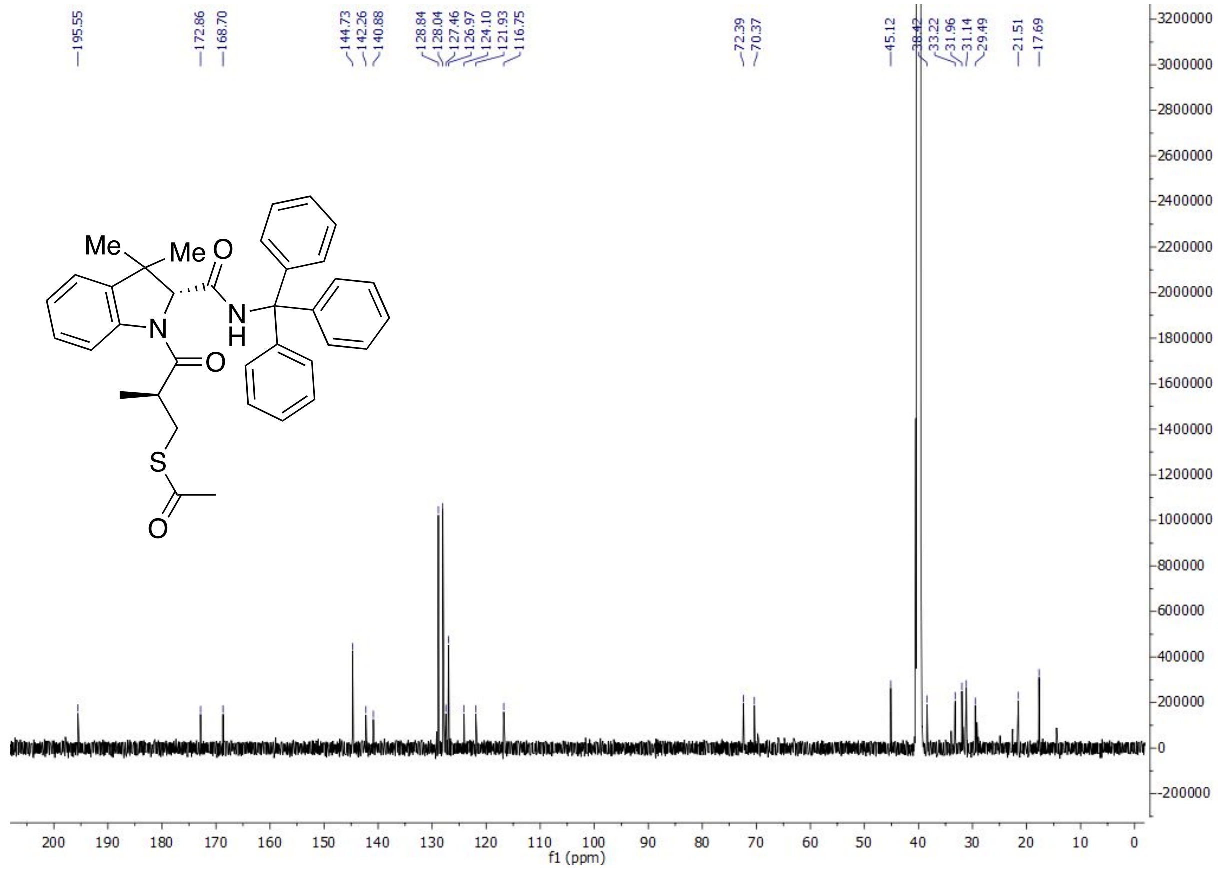

^{1}H NMR of compound *(S,S')*-**14a**

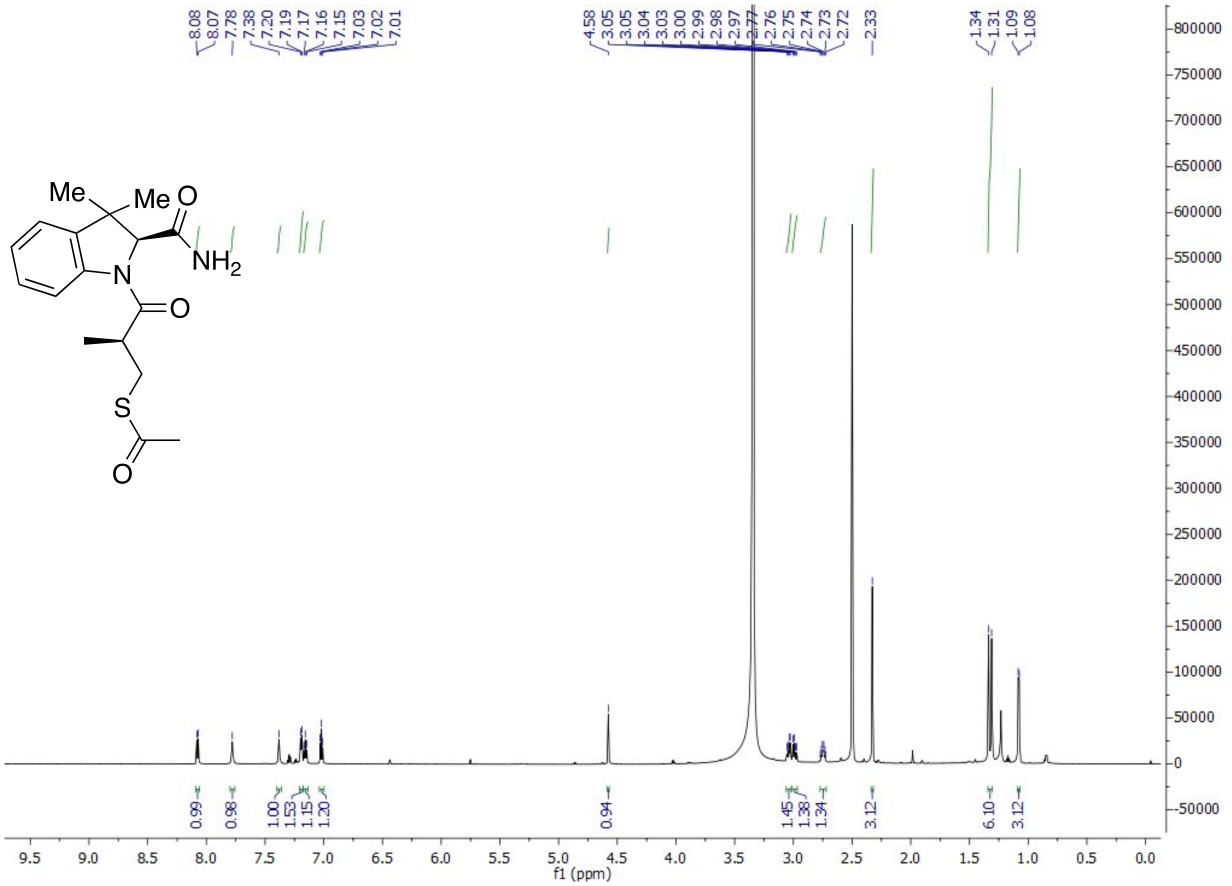

^{1}H NMR of compound *(S,S')*-**14a**

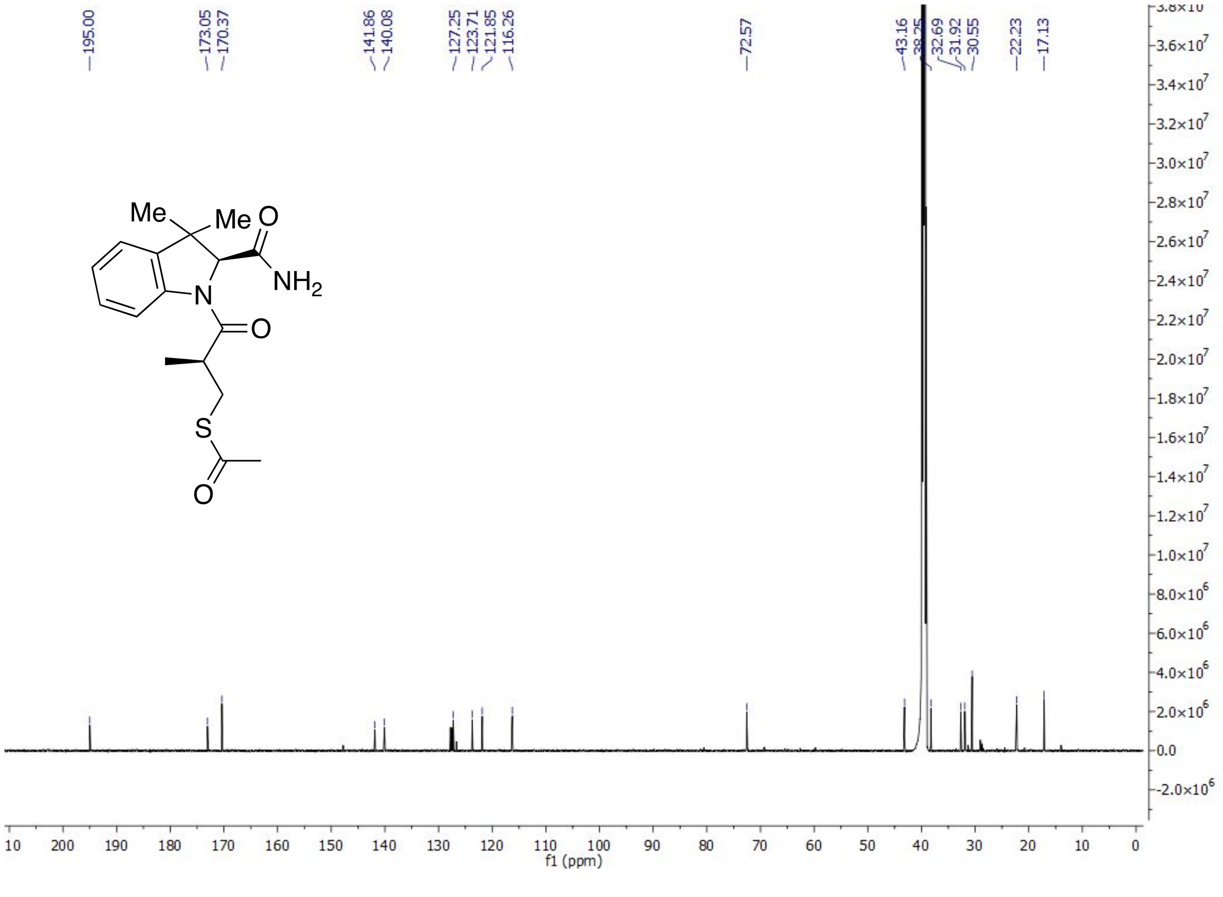

^{1}H NMR of compound **6a**

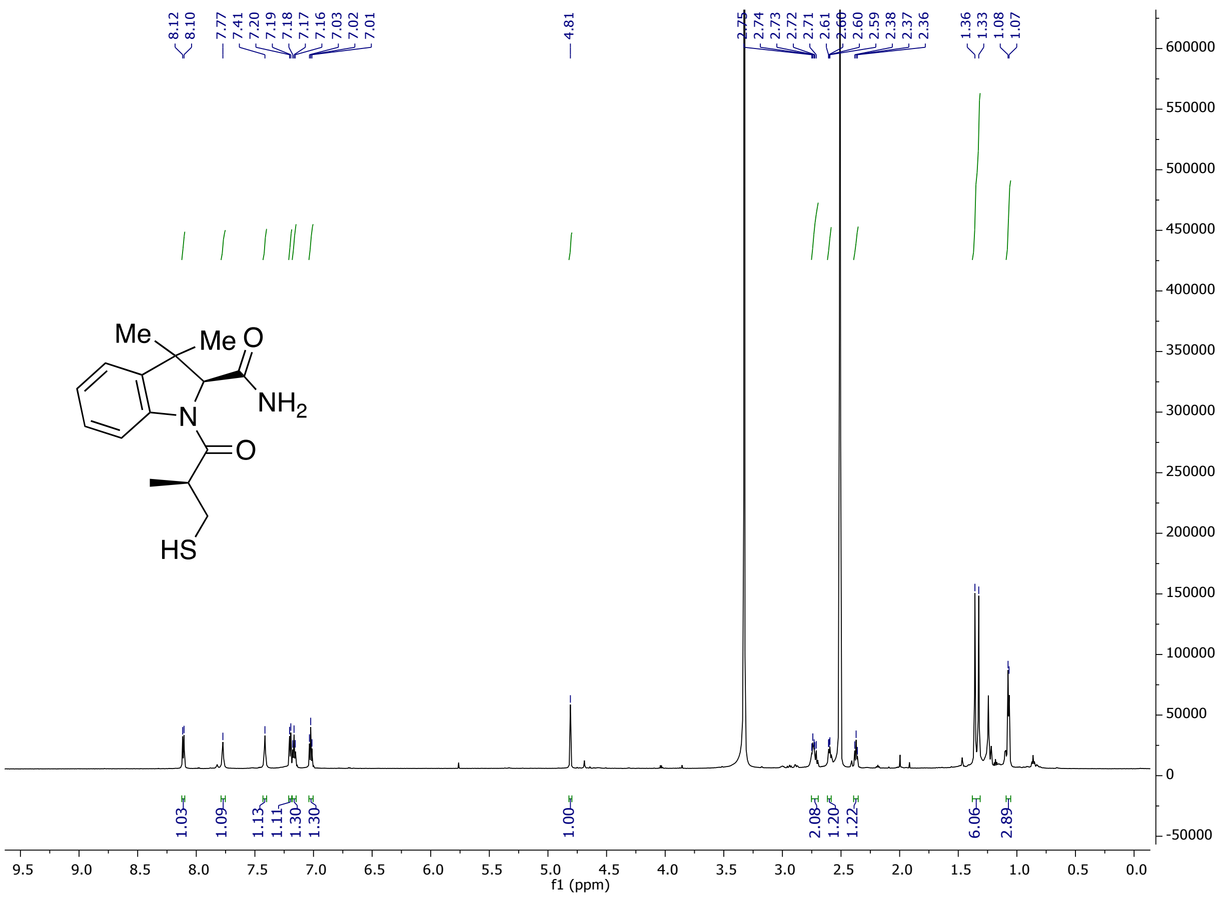

^{13}C NMR of compound **6a**

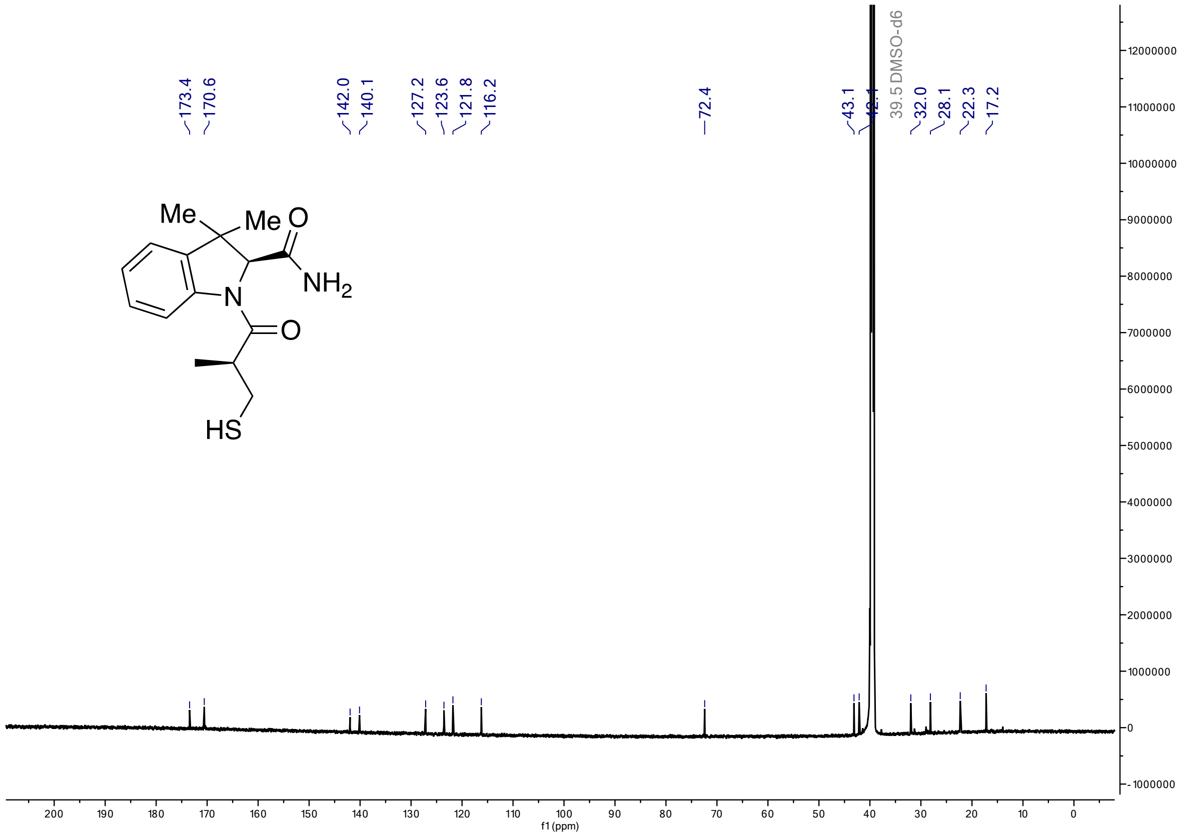

^{1}H NMR of compound *(S,R')*-**14a**

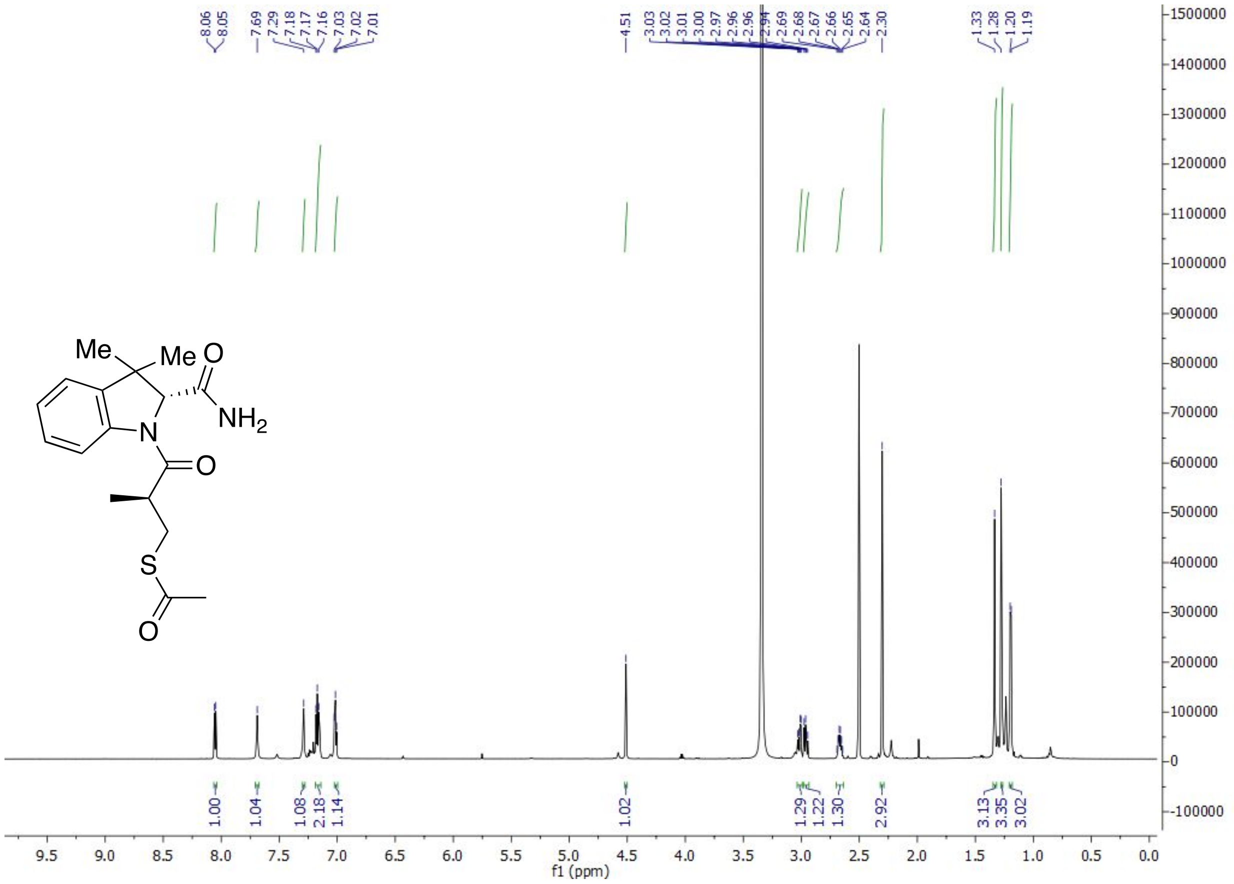

^{13}C NMR of compound *(S,R')*-**14a**

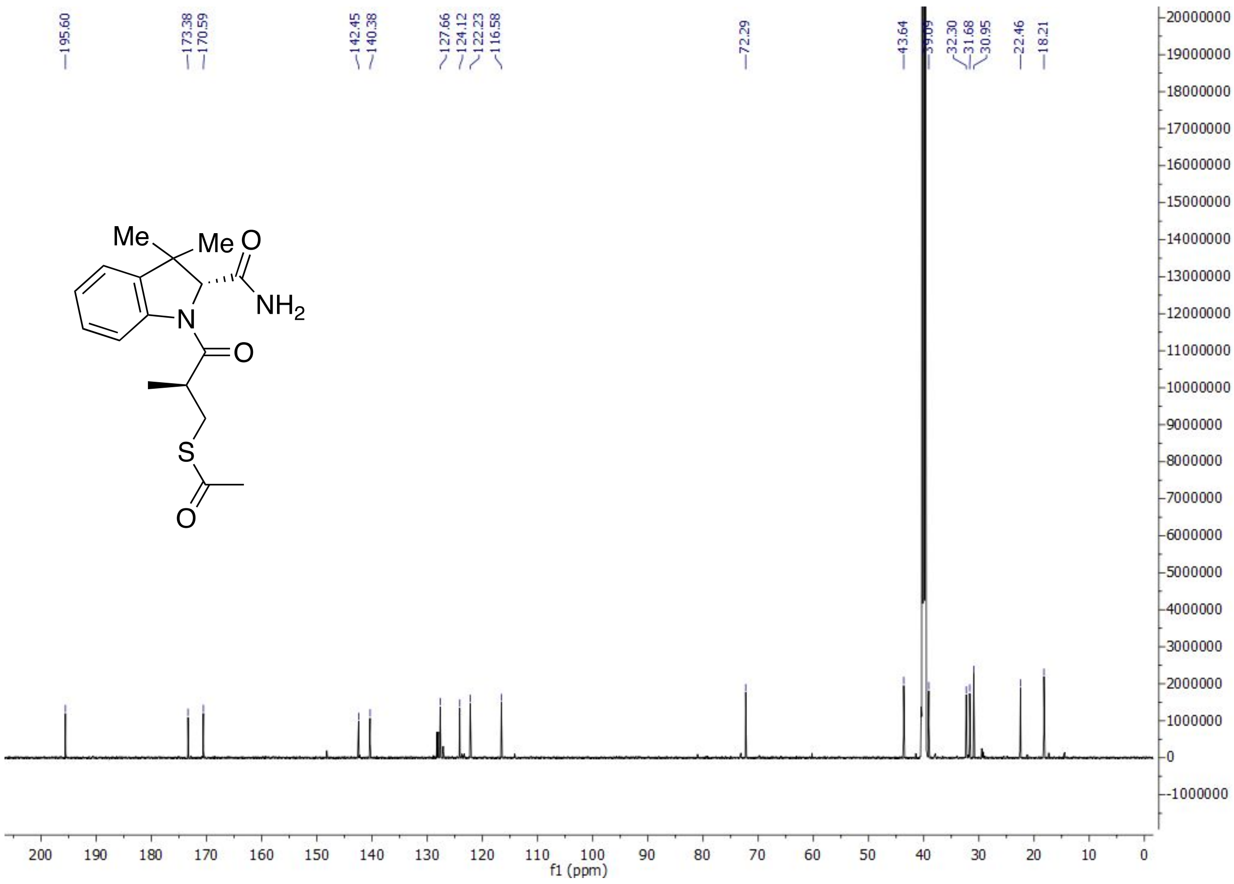

^{1}H NMR of compound **6b**

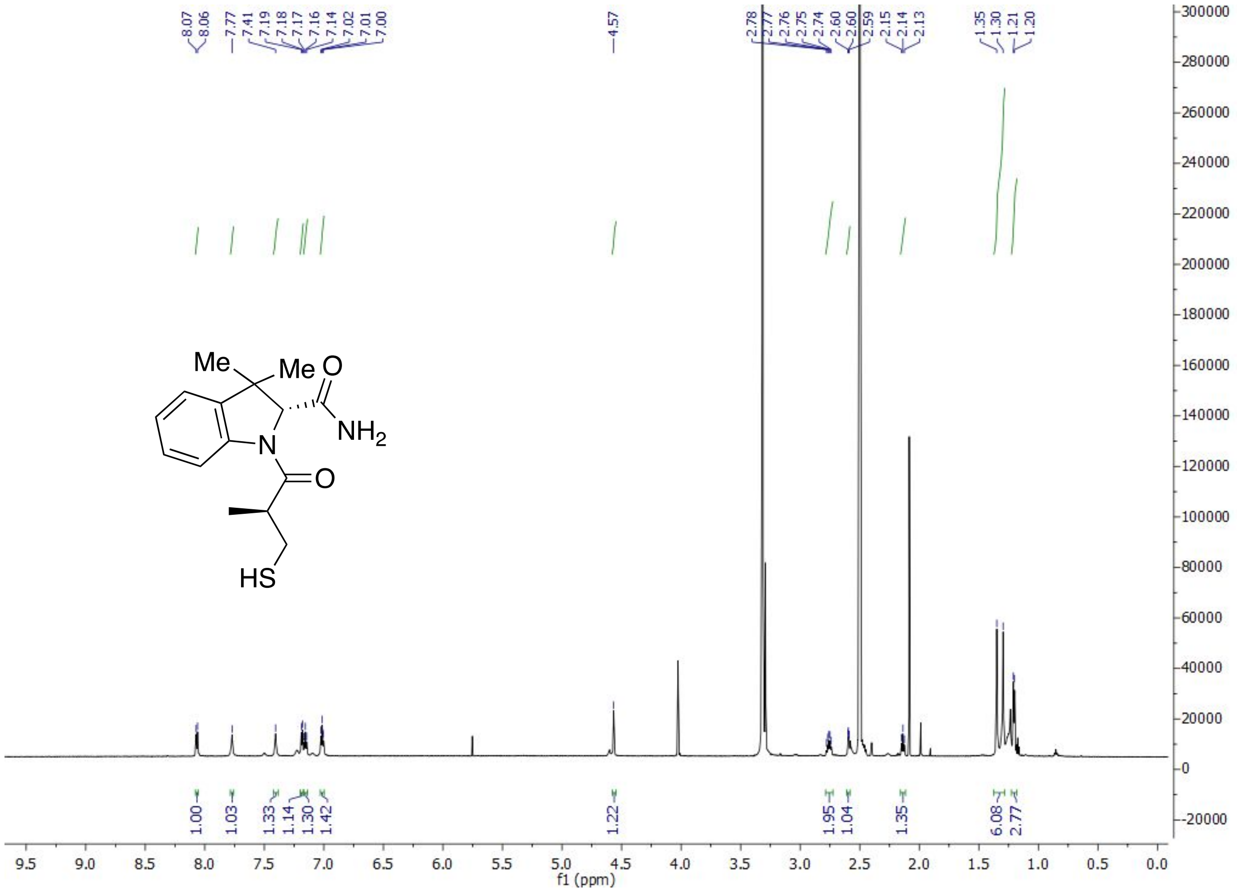

^{13}C NMR of compound *(S,R*)*-**6b**

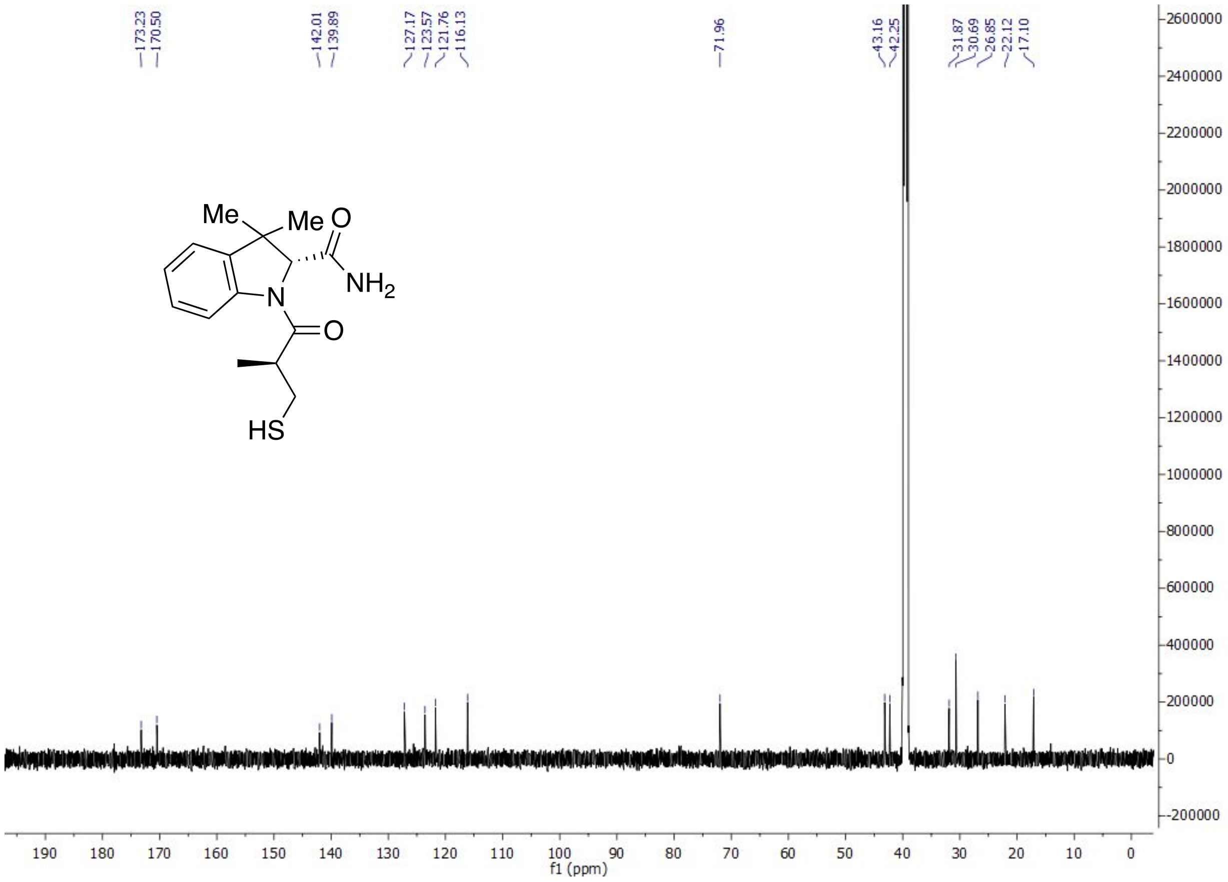

^{1}H NMR of compound *(S,S')*-**13b**

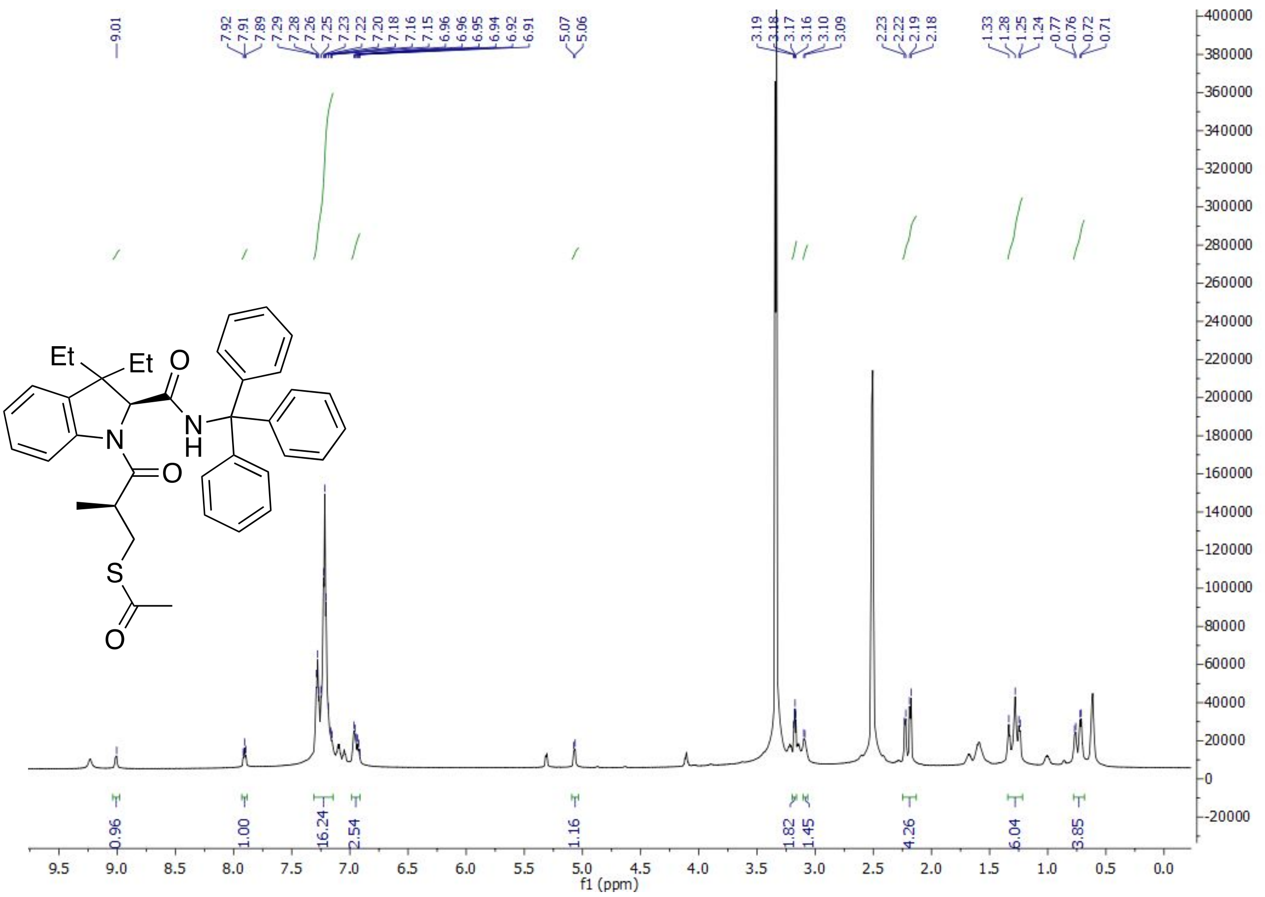

^{13}C NMR of compound *(S,S')*-**13b**

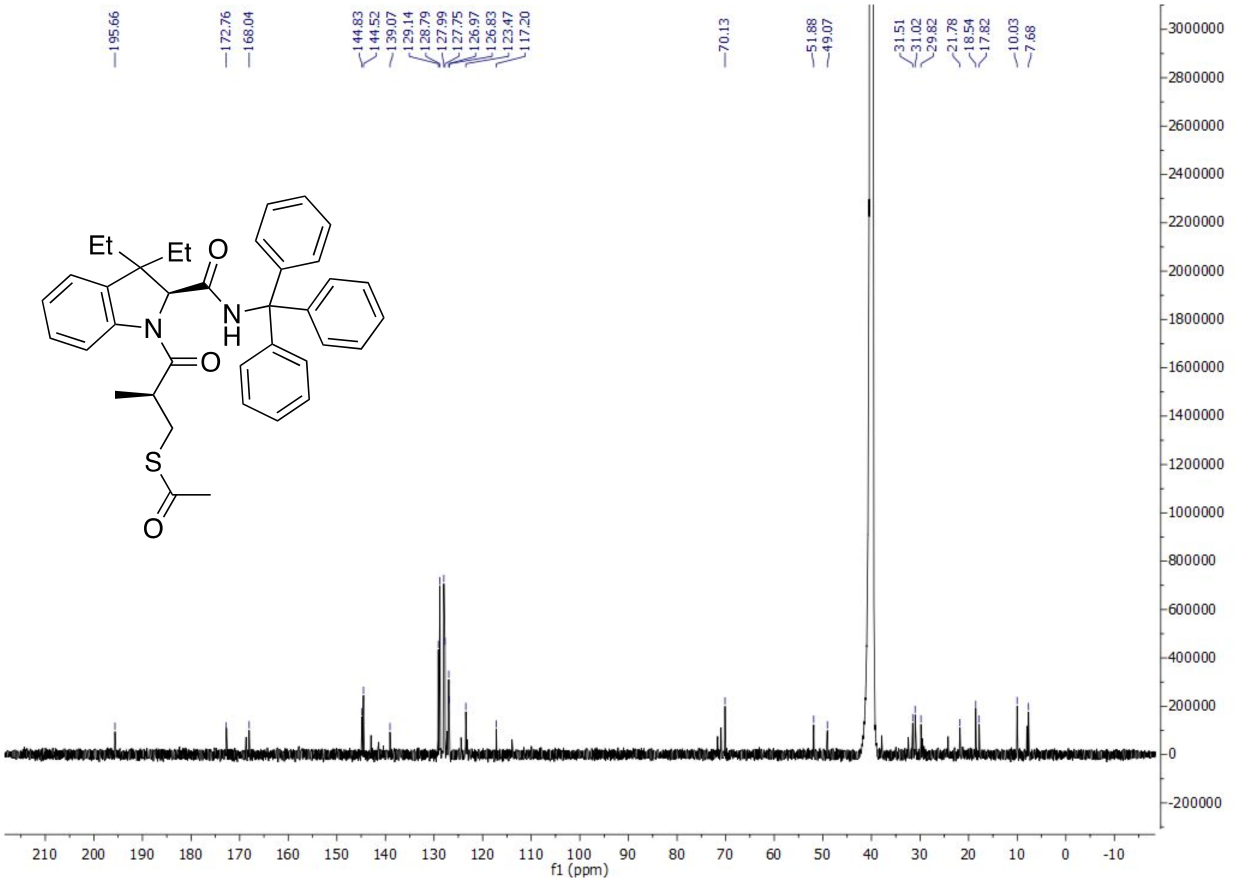

^{1}H NMR of compound *(S,R')*-**13b**

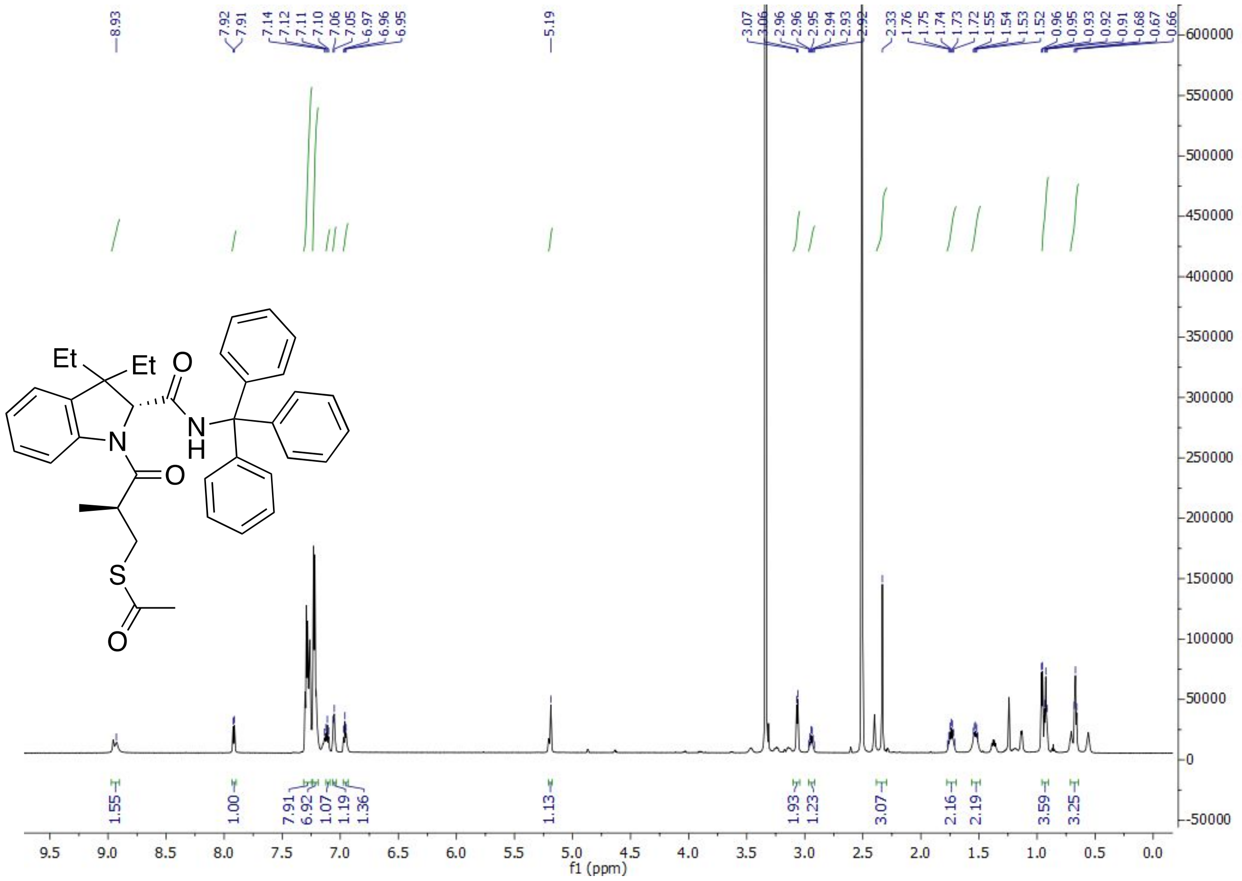

^{13}C NMR of compound *(S,R')*-**13b**

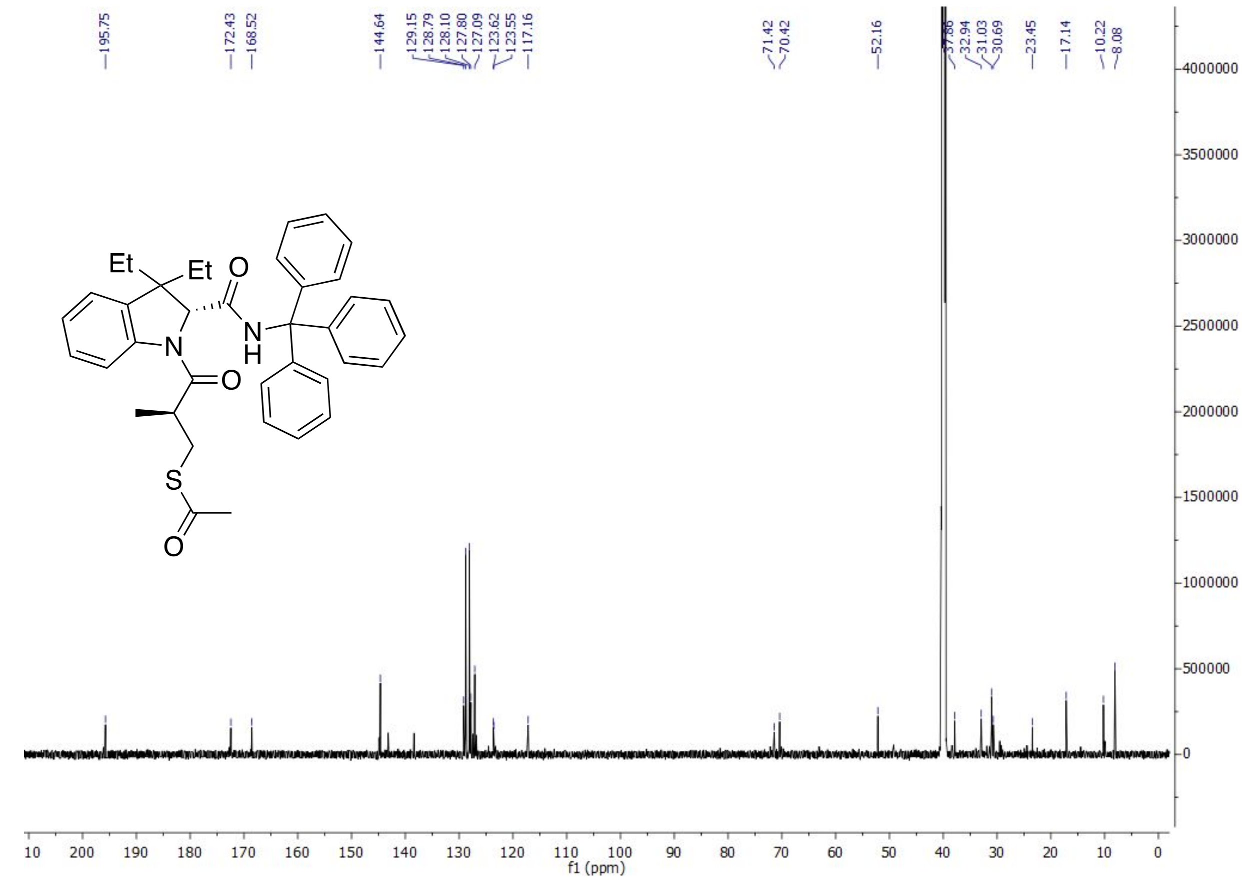

^{1}H NMR of compound *(S,S')*-**14b**

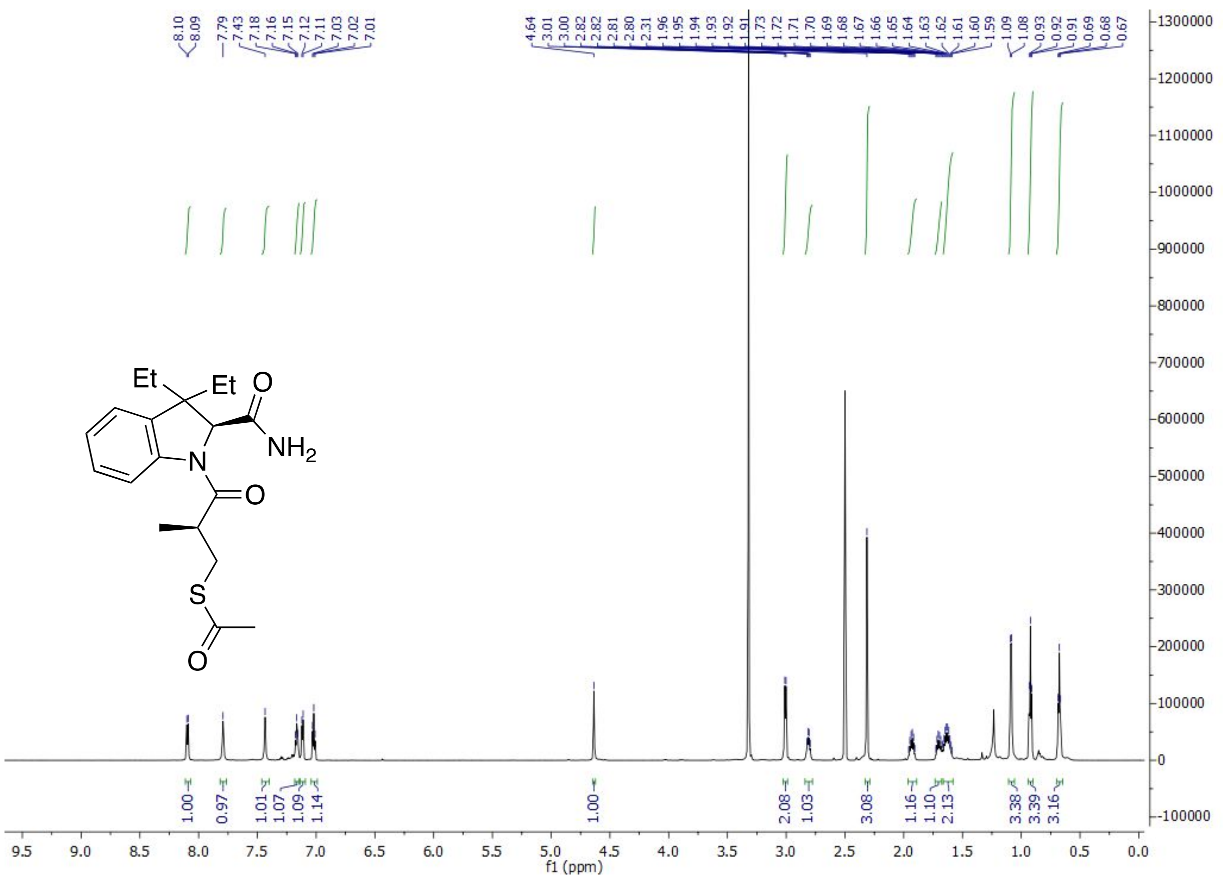

^{13}C NMR of compound *(S,S')*-**14b**

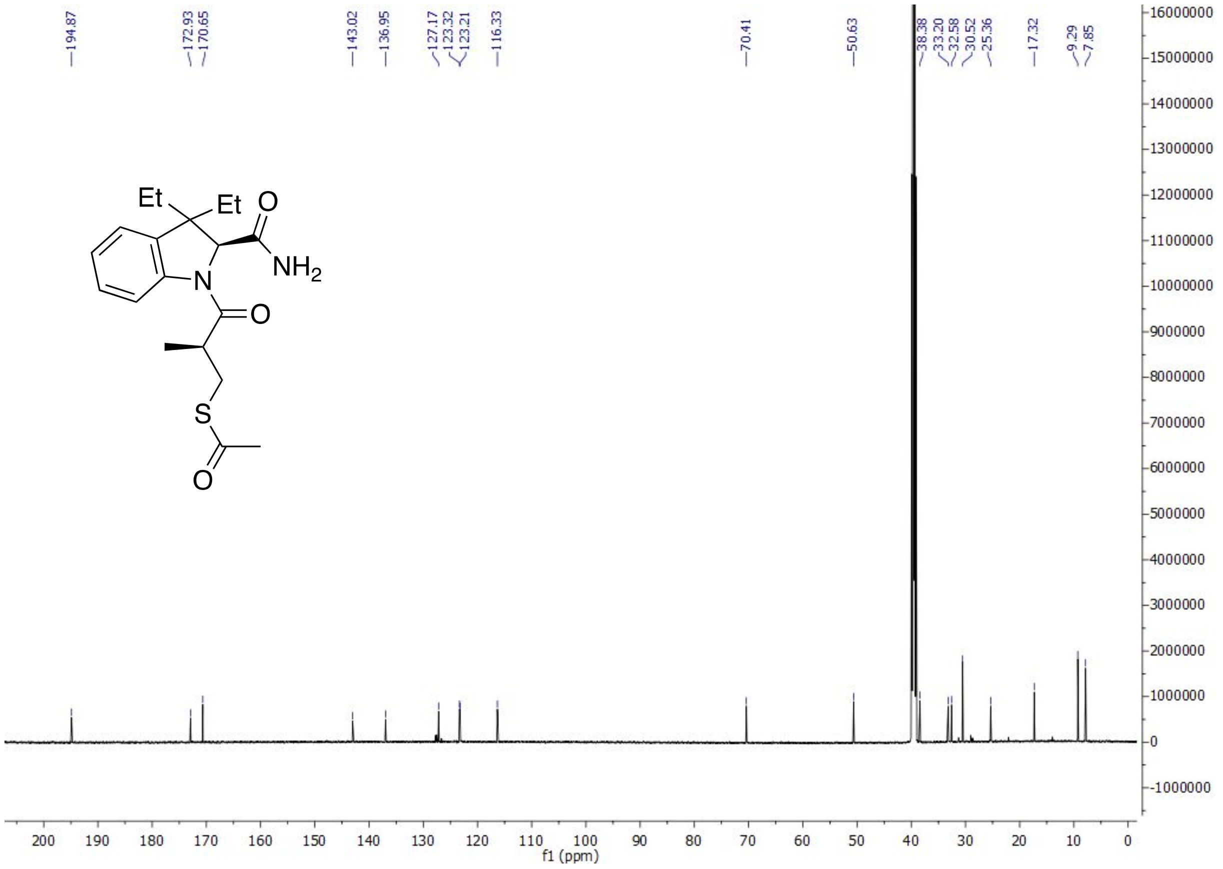

^{1}H NMR of compound **6c**

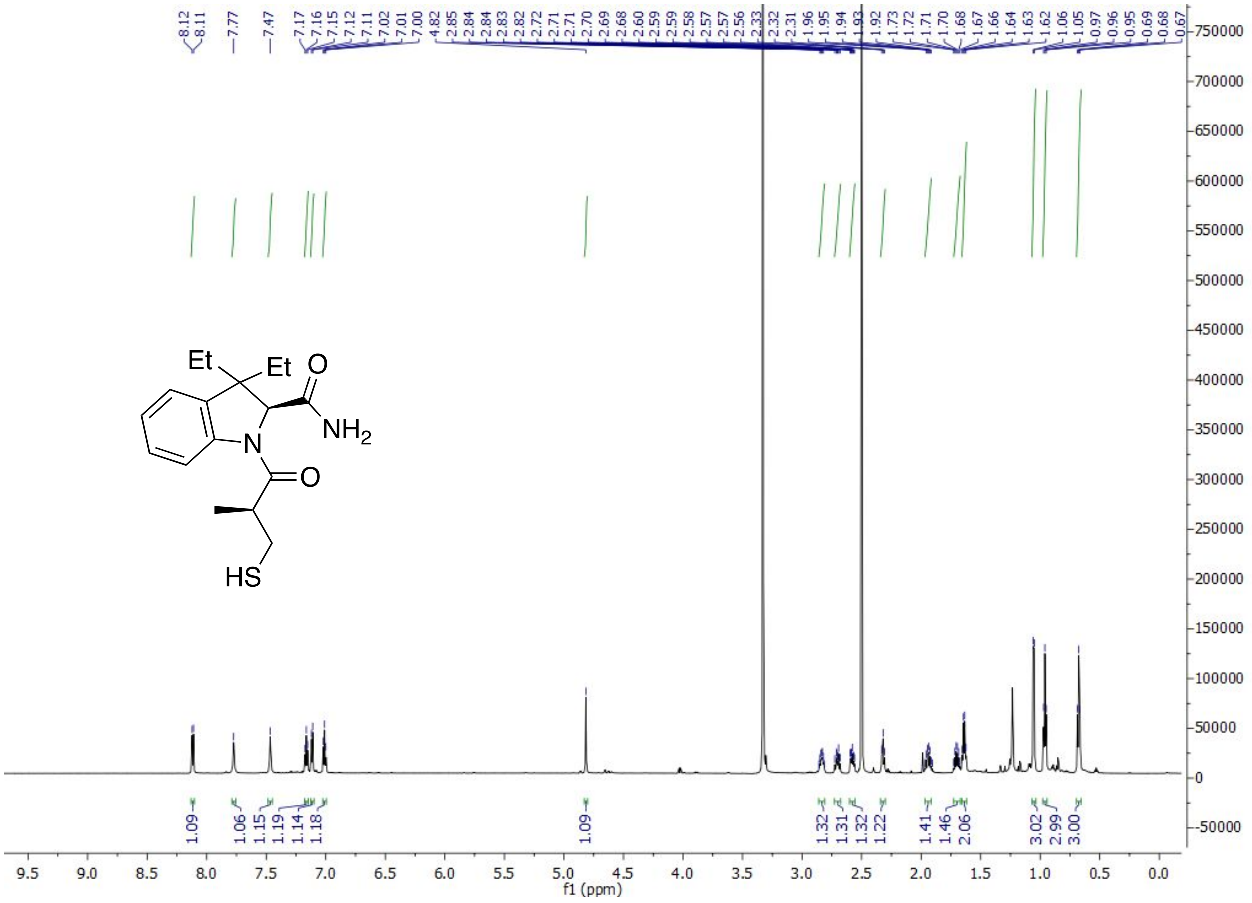

^{13}C NMR of compound **6c**

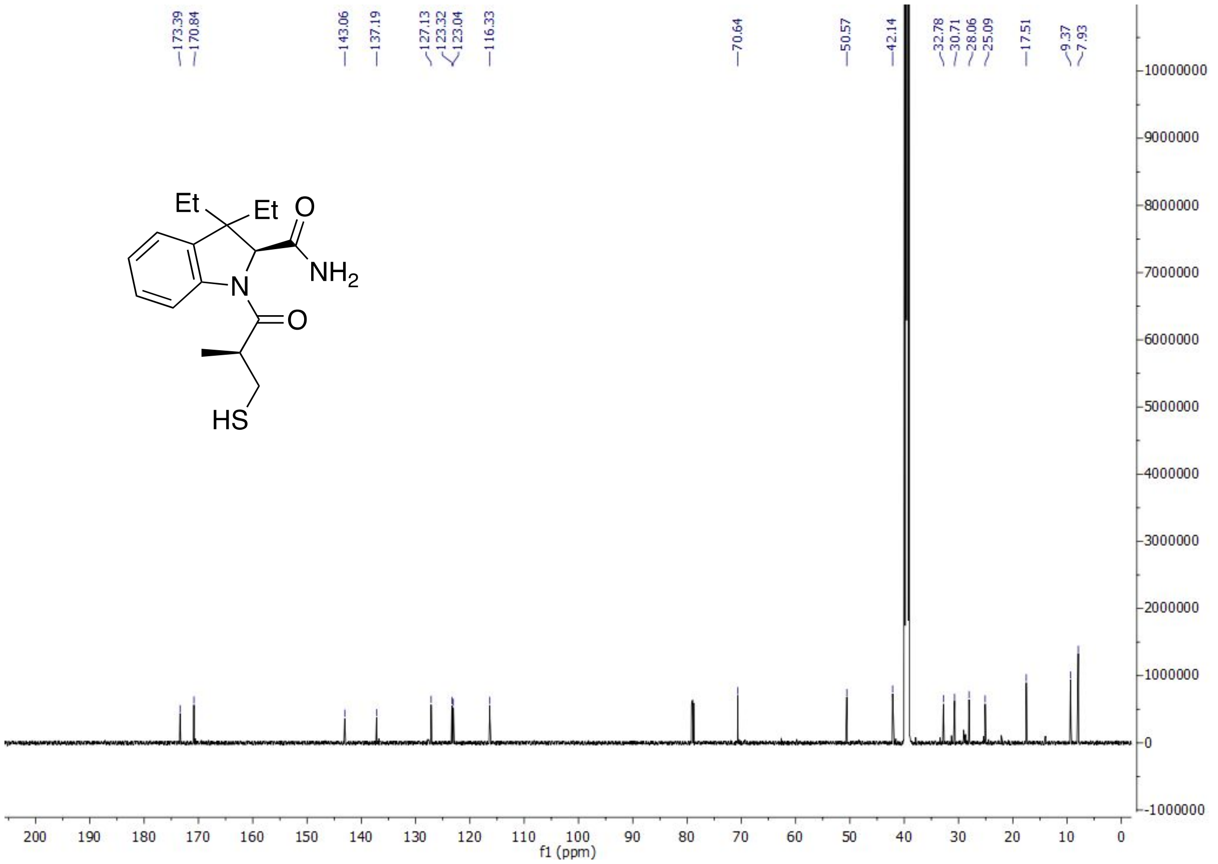

^{1}H NMR of compound *(S,R')*-**14b**

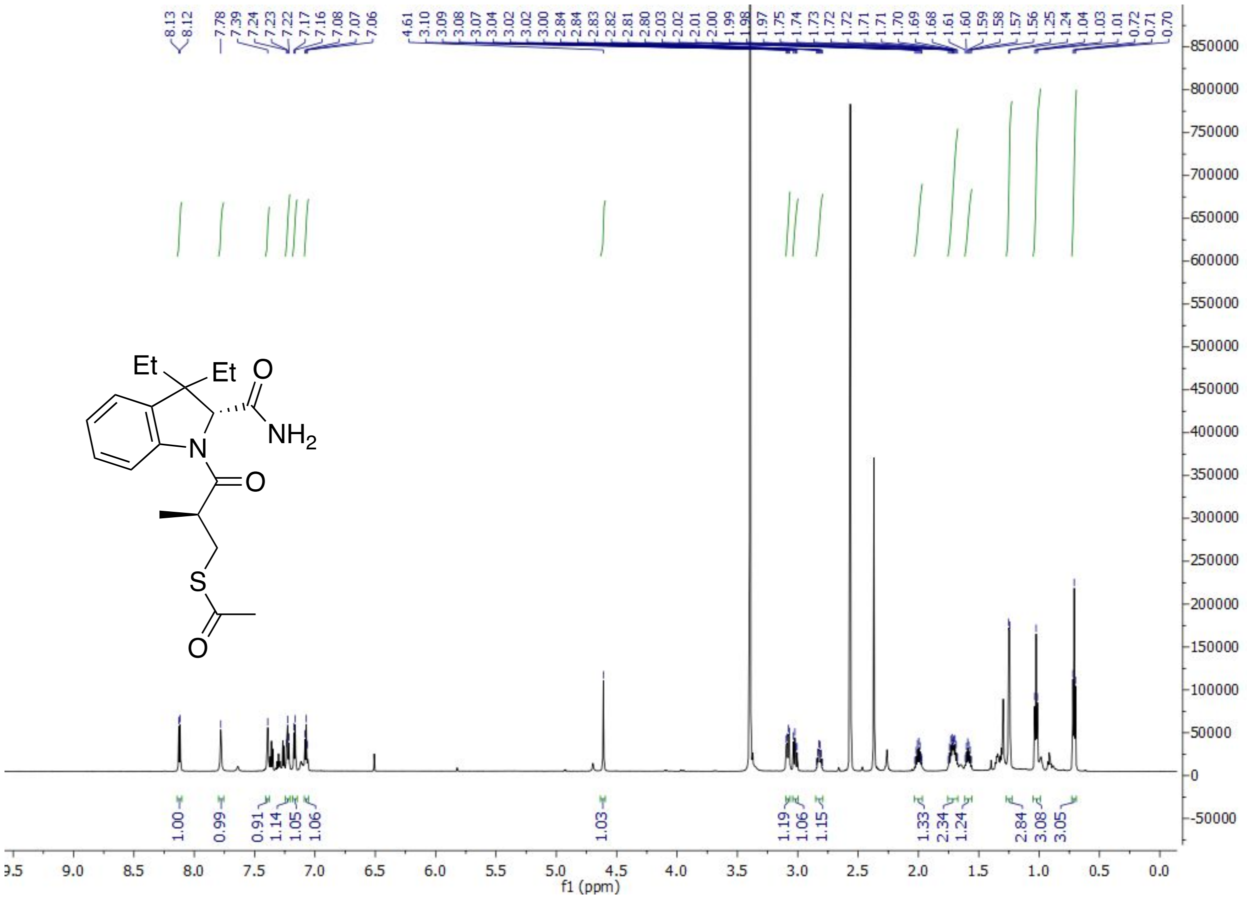

^{13}C NMR of compound *(S,R')*-**14b**

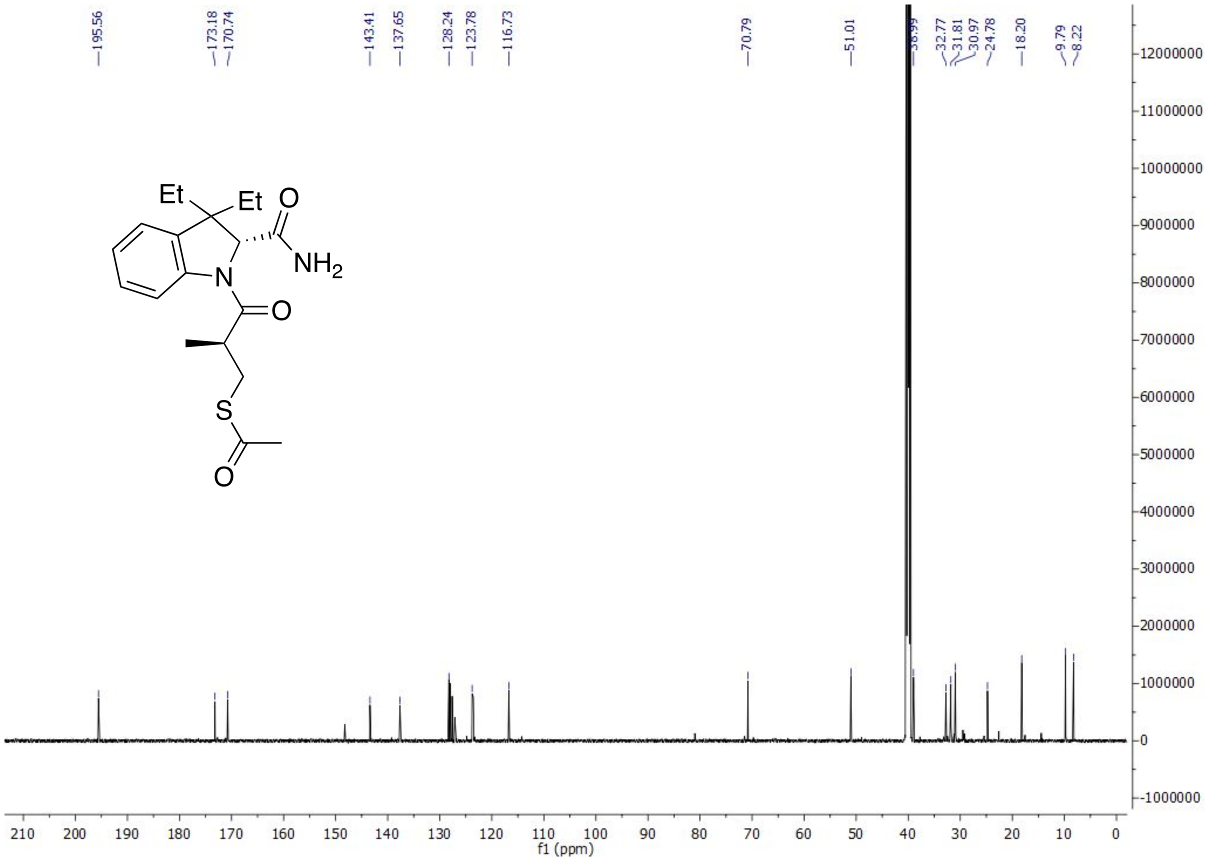

^{1}H NMR of compound **6d**

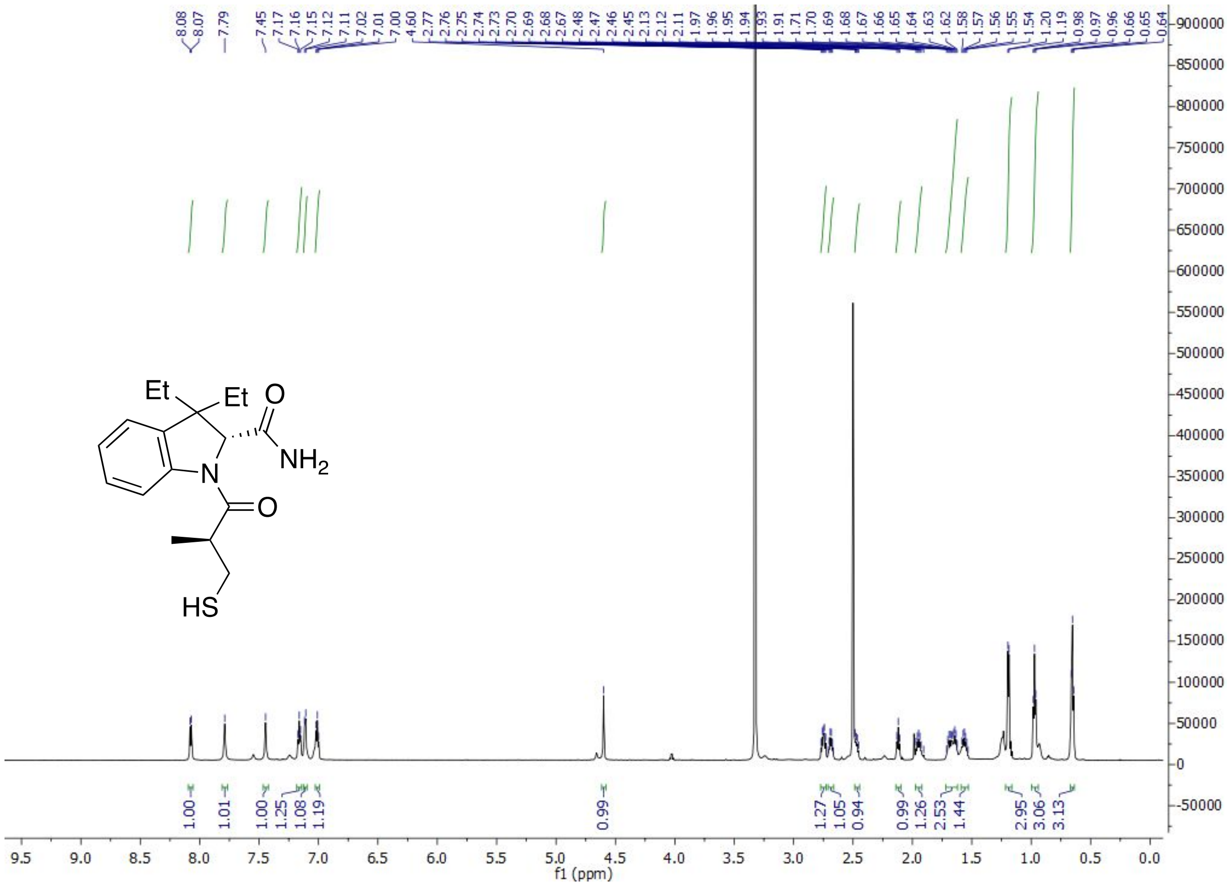

^{13}C NMR of compound **6d**

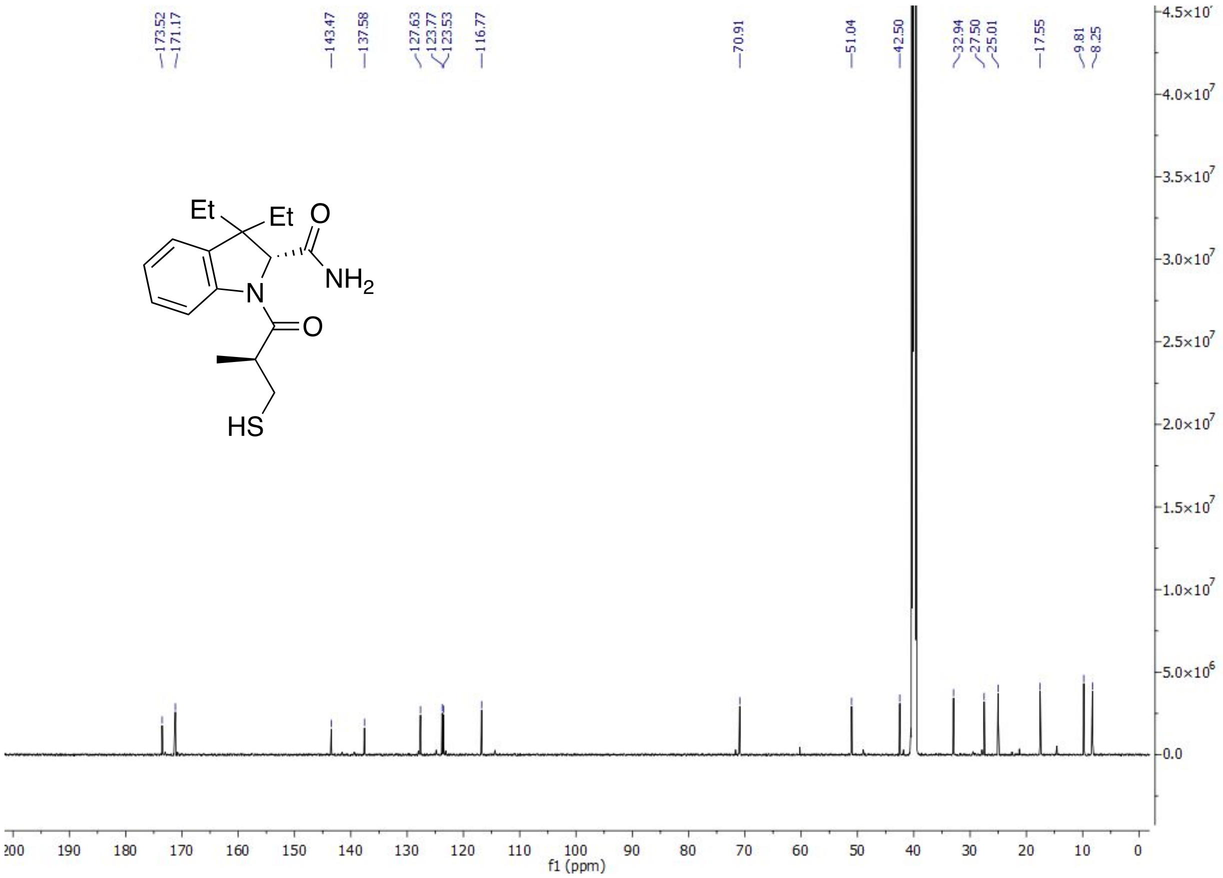

^{1}H NMR of compound *(S,S')*-**14c**

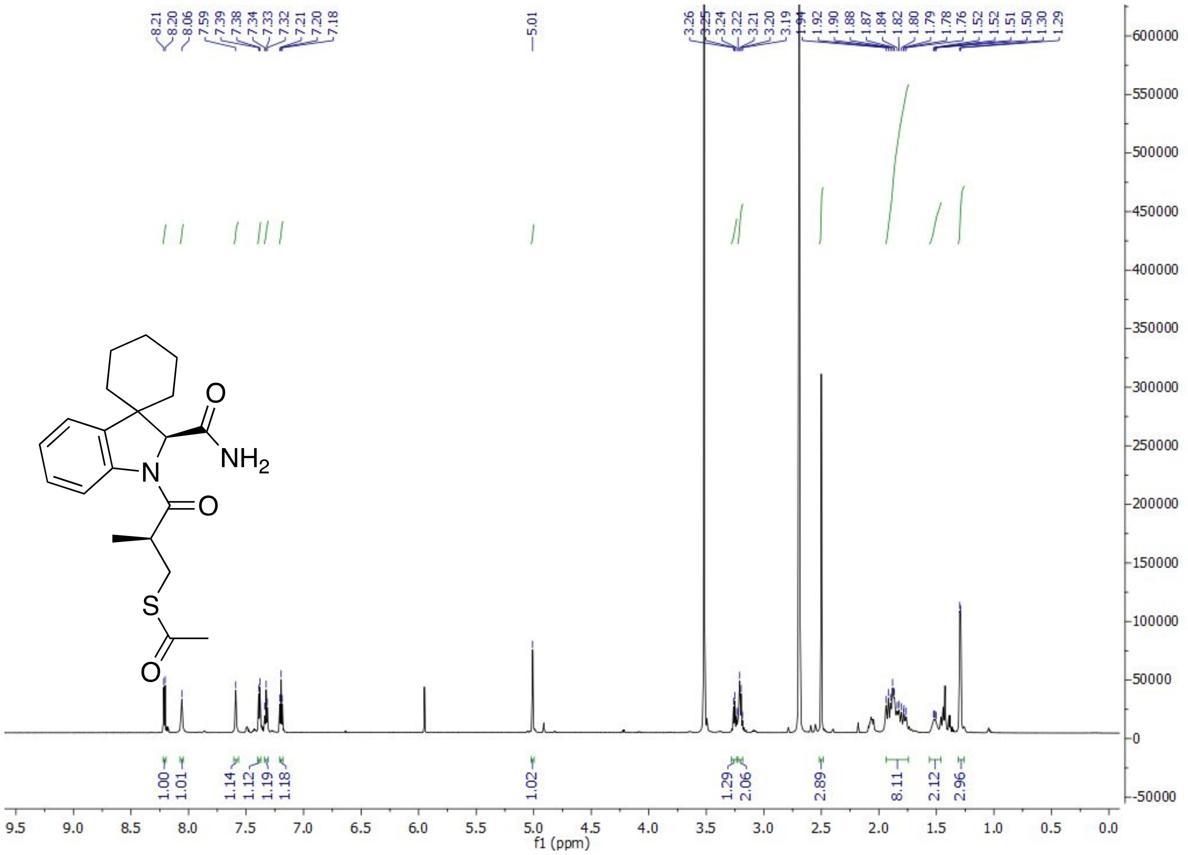

^{13}C NMR of compound *(S,S')*-**14c**

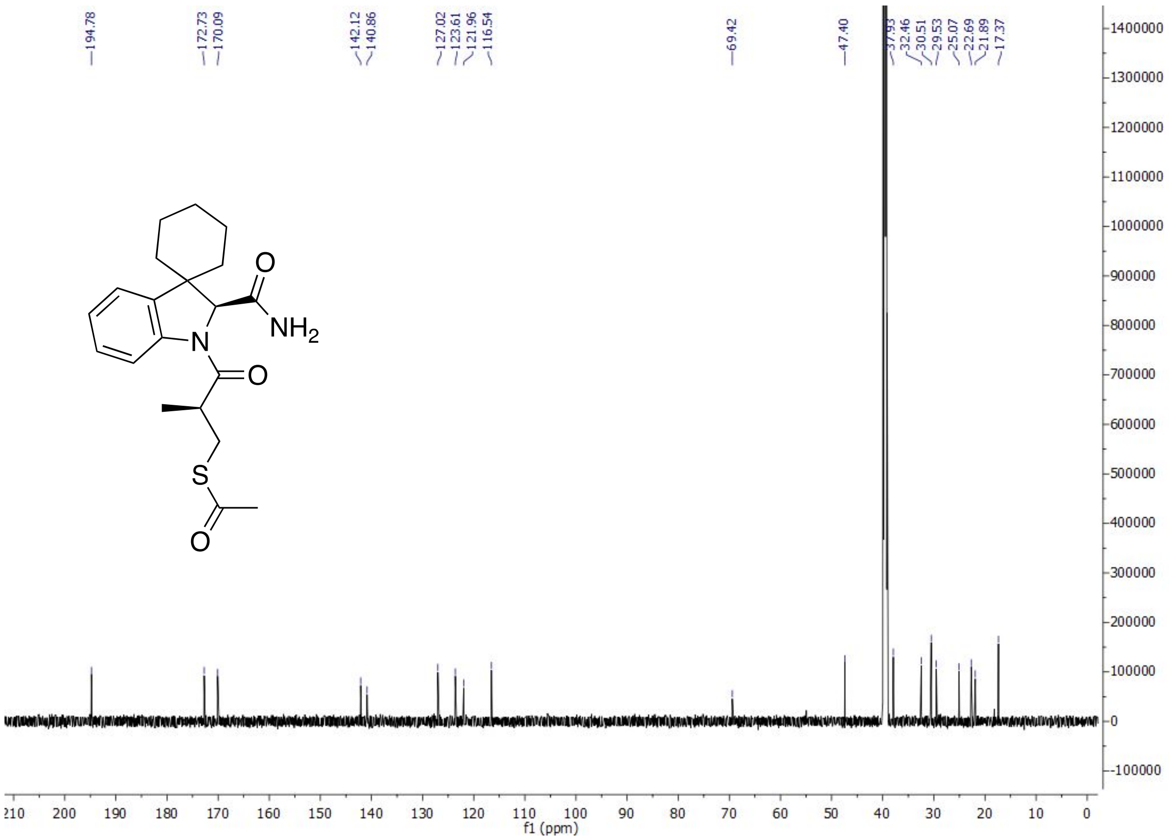

^{1}H NMR of compound **6e**

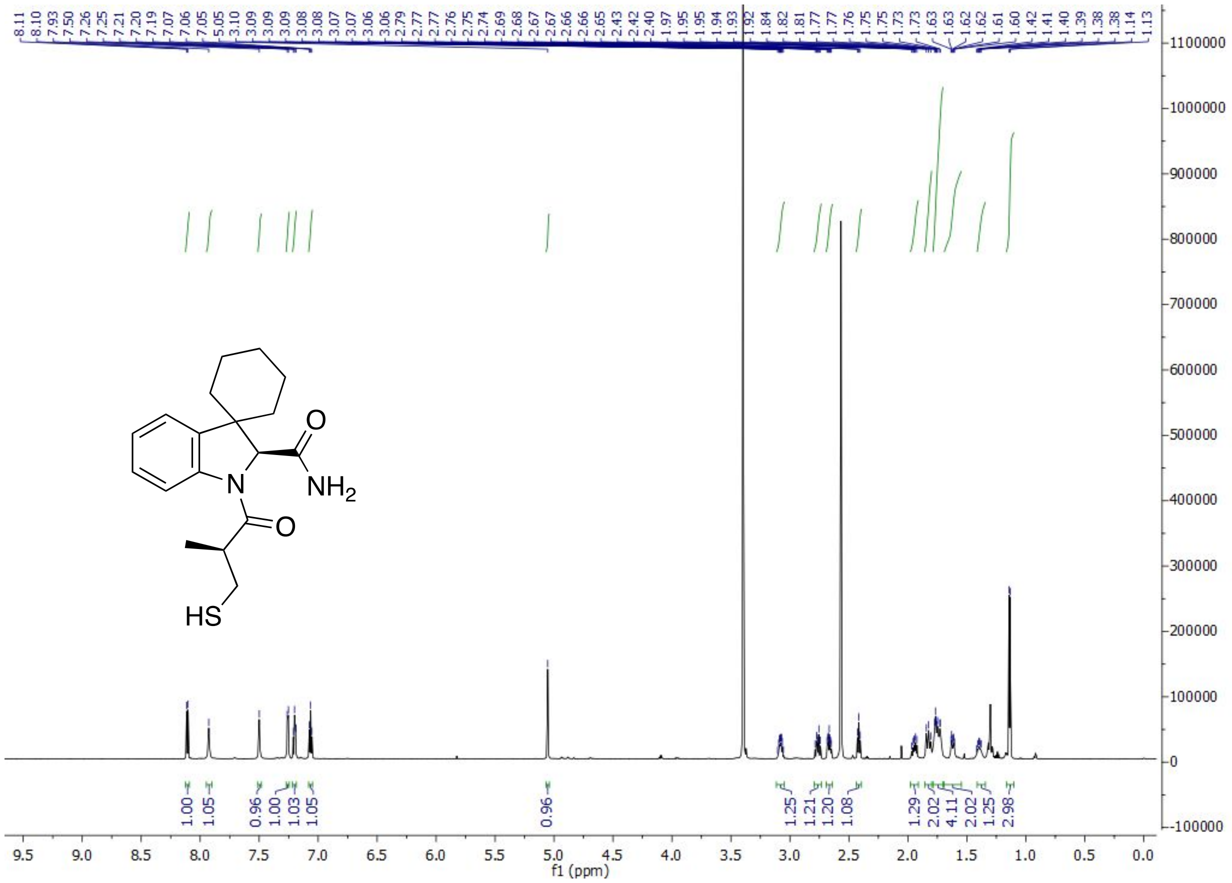

[13]C

^{13}C NMR of compound **6e**

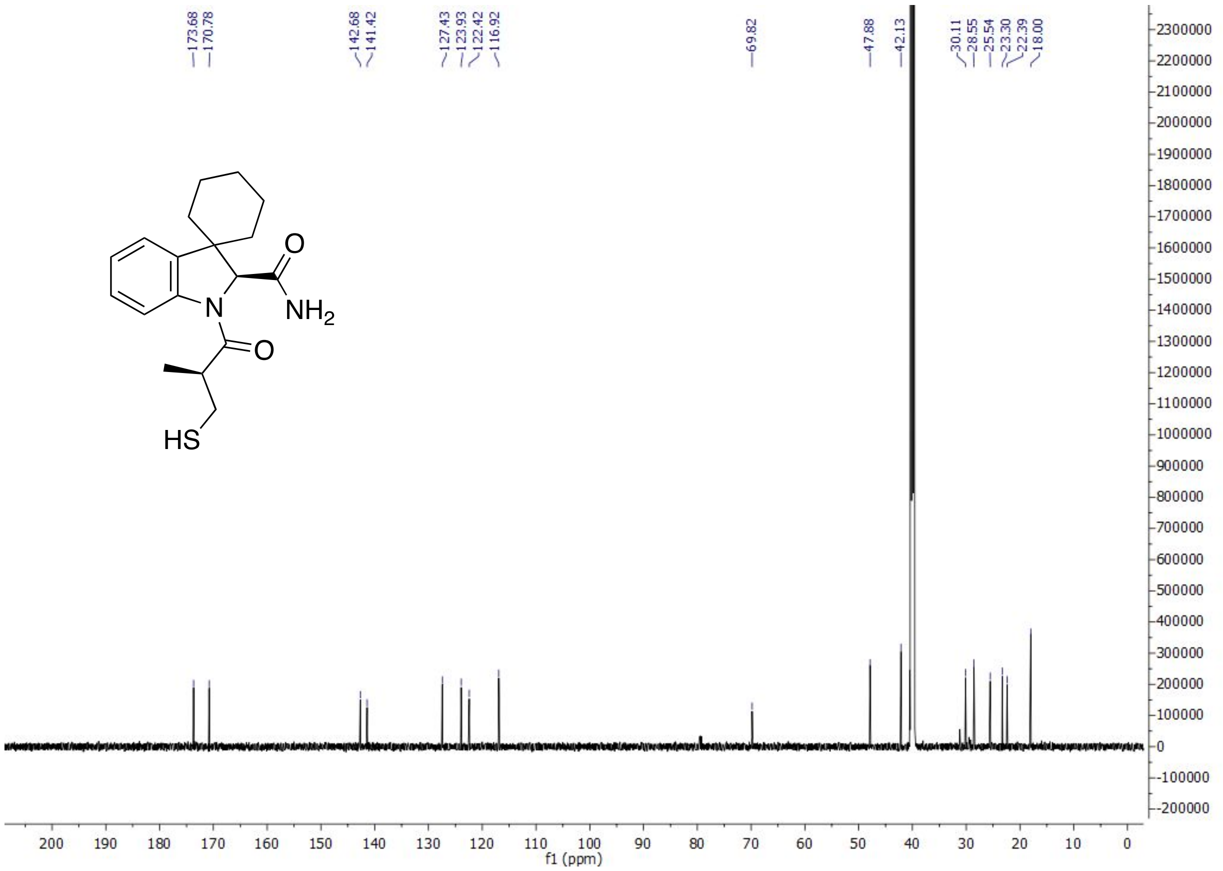

^{1}H NMR of compound *(S,R')*-**14c**

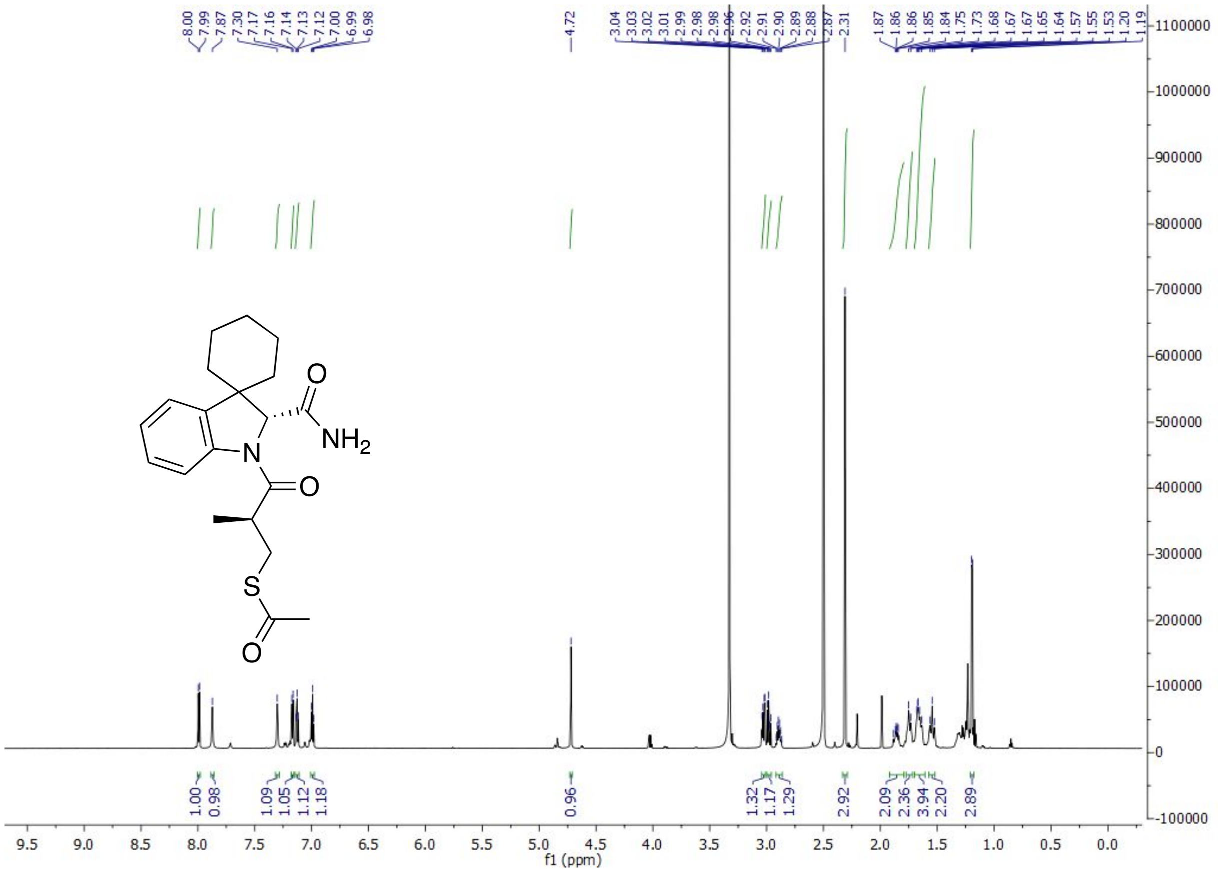

^{13}C NMR of compound *(S,R')*-**14c**

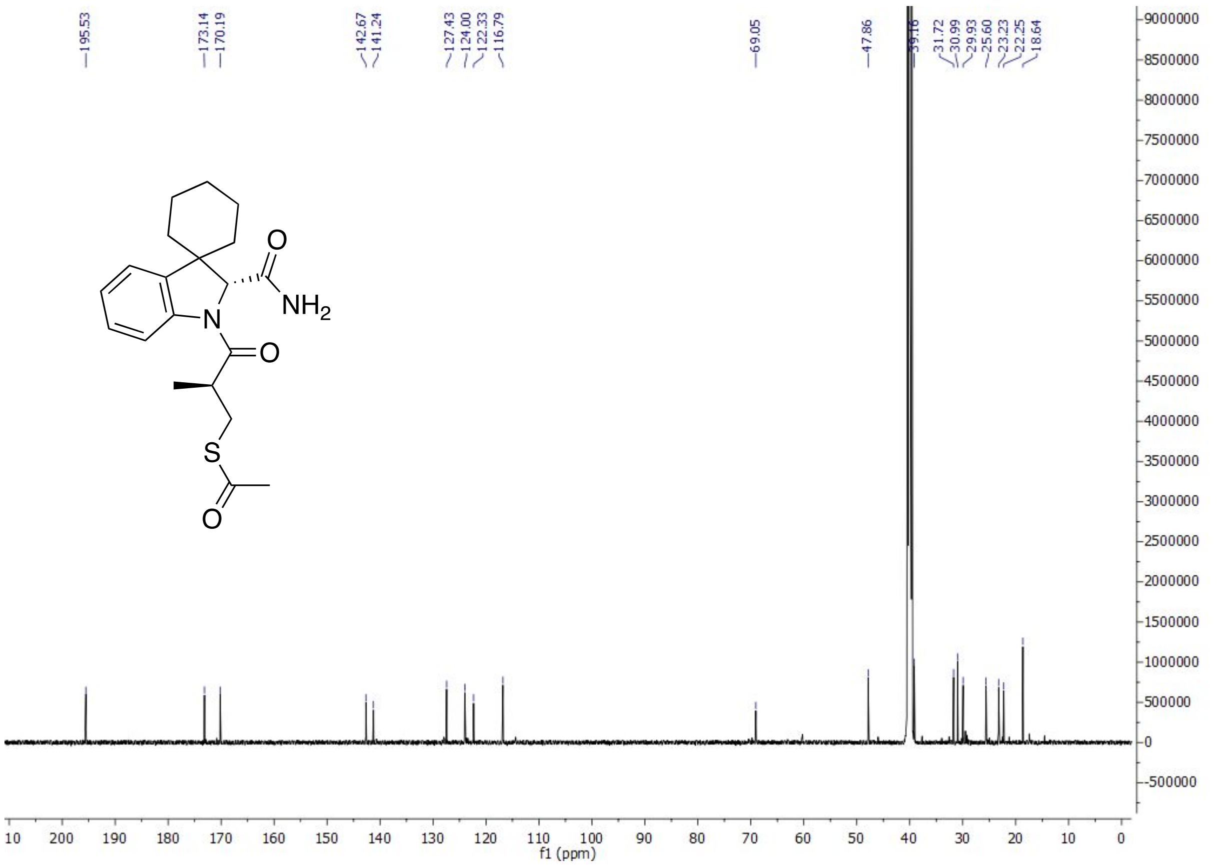

^{1}H NMR of compound **6f**

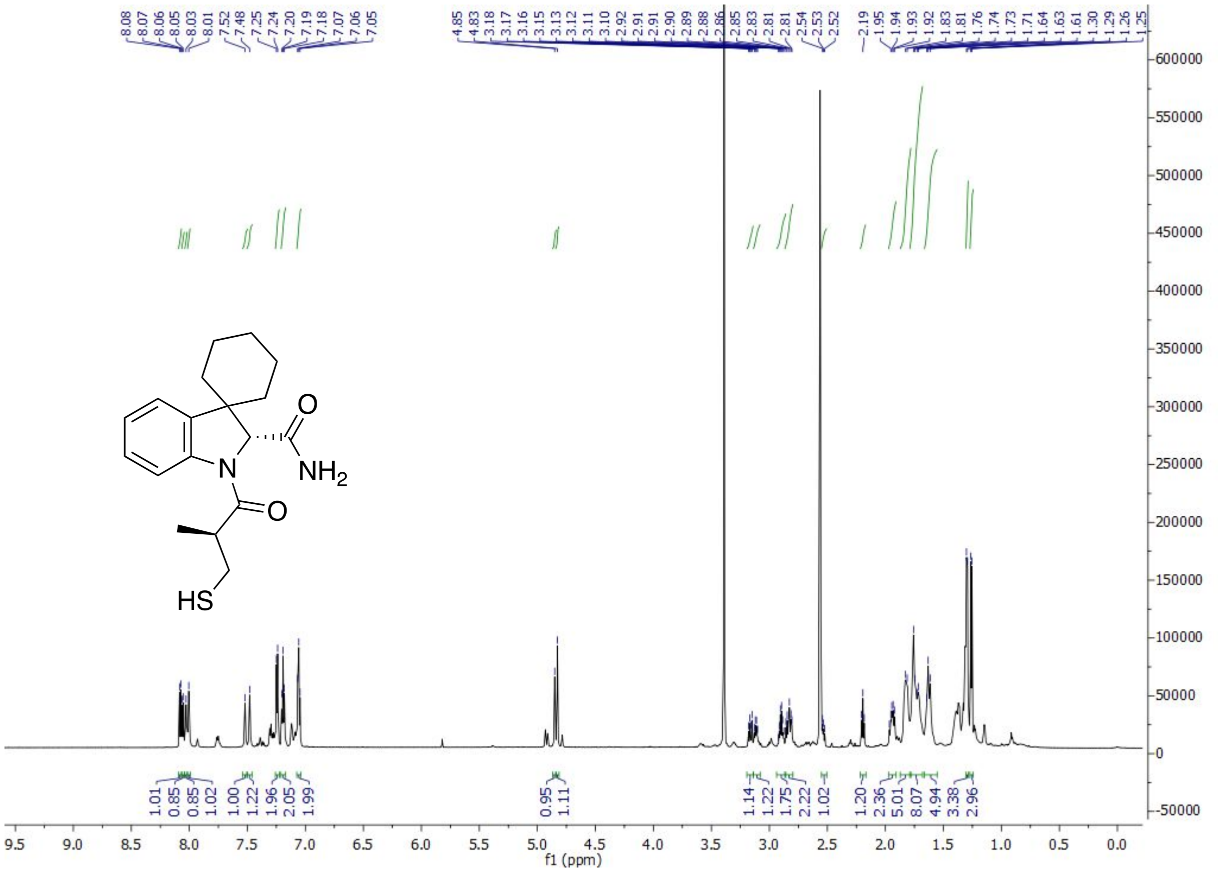

^{13}C NMR of compound **6f**

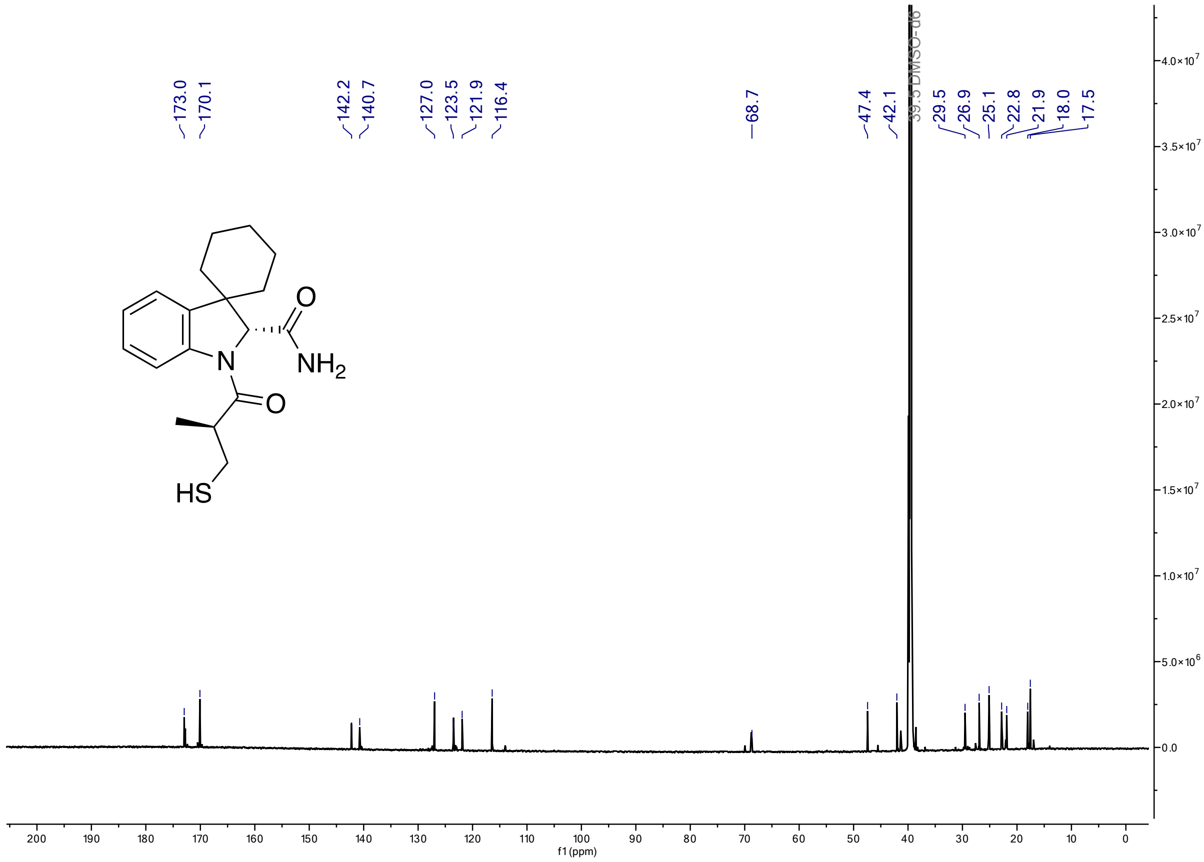

^{1}H NMR of compound *(S,S')*-**16a**, *(S,R')*-**16a**

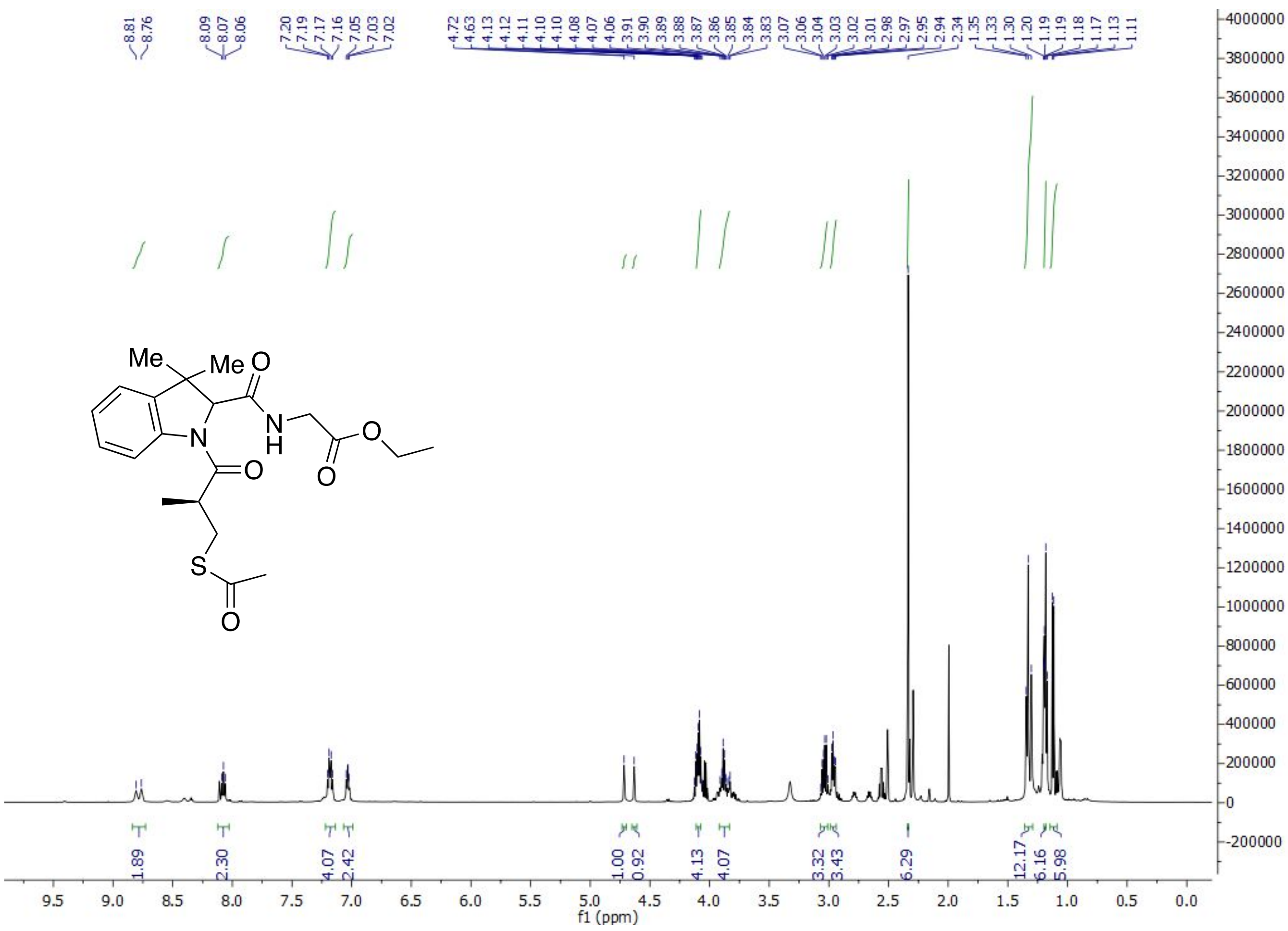

^{1}H NMR of compound **7a**

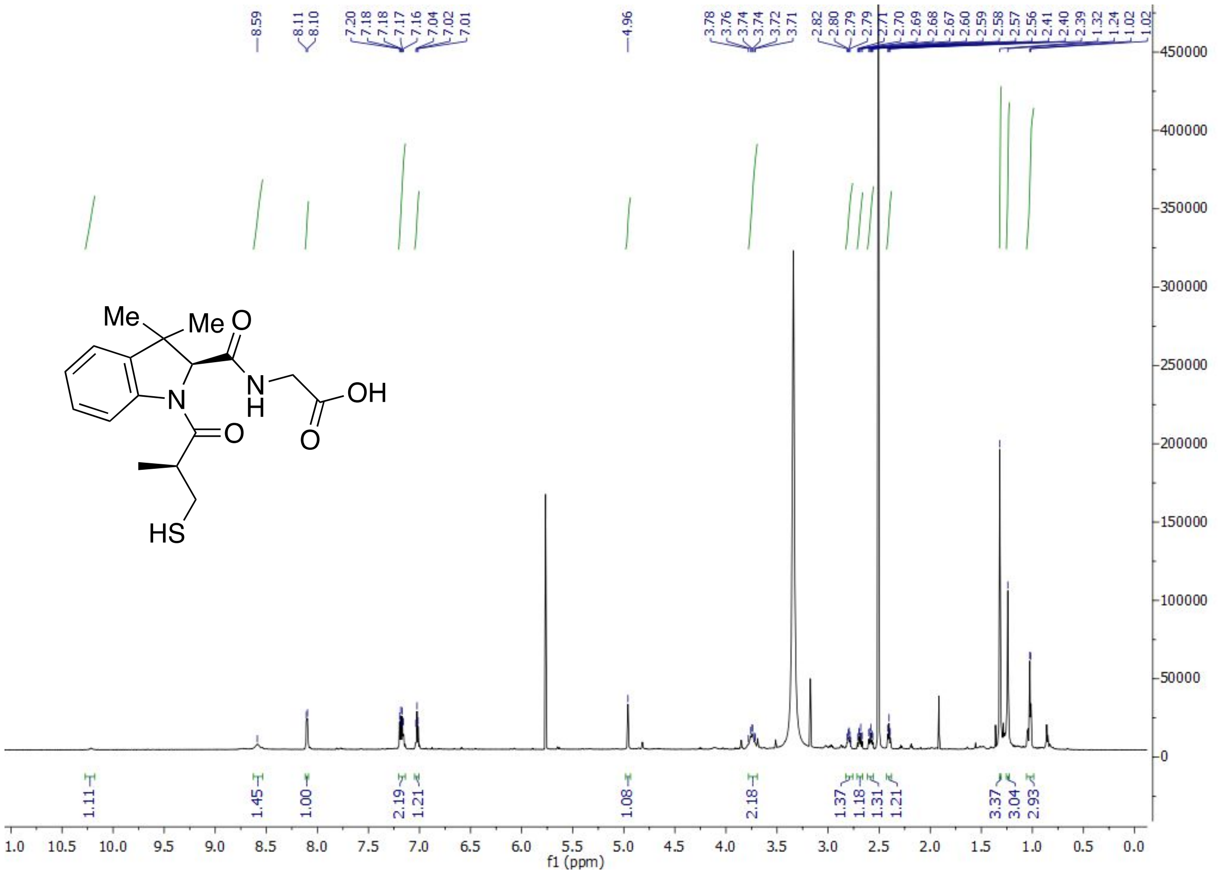

^{13}C NMR of compound **7a**

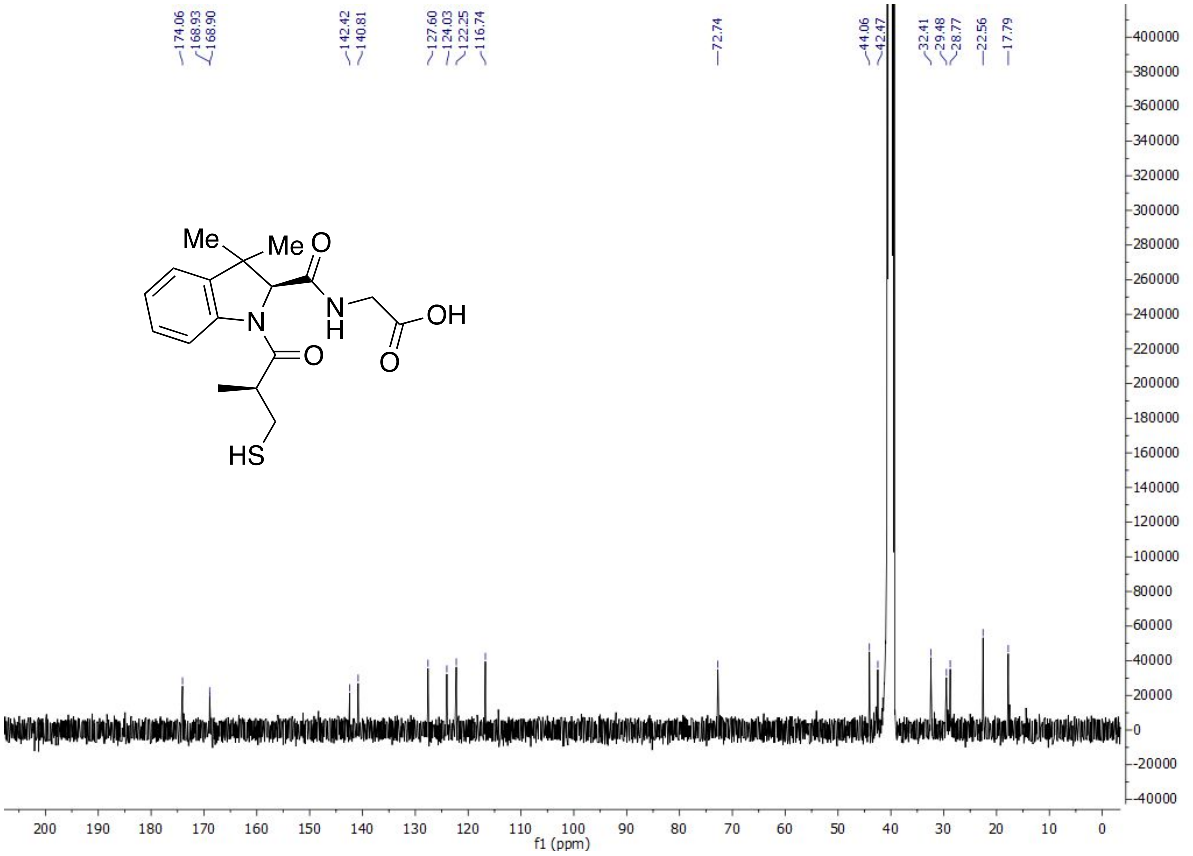

^{1}H NMR of compound **7b**

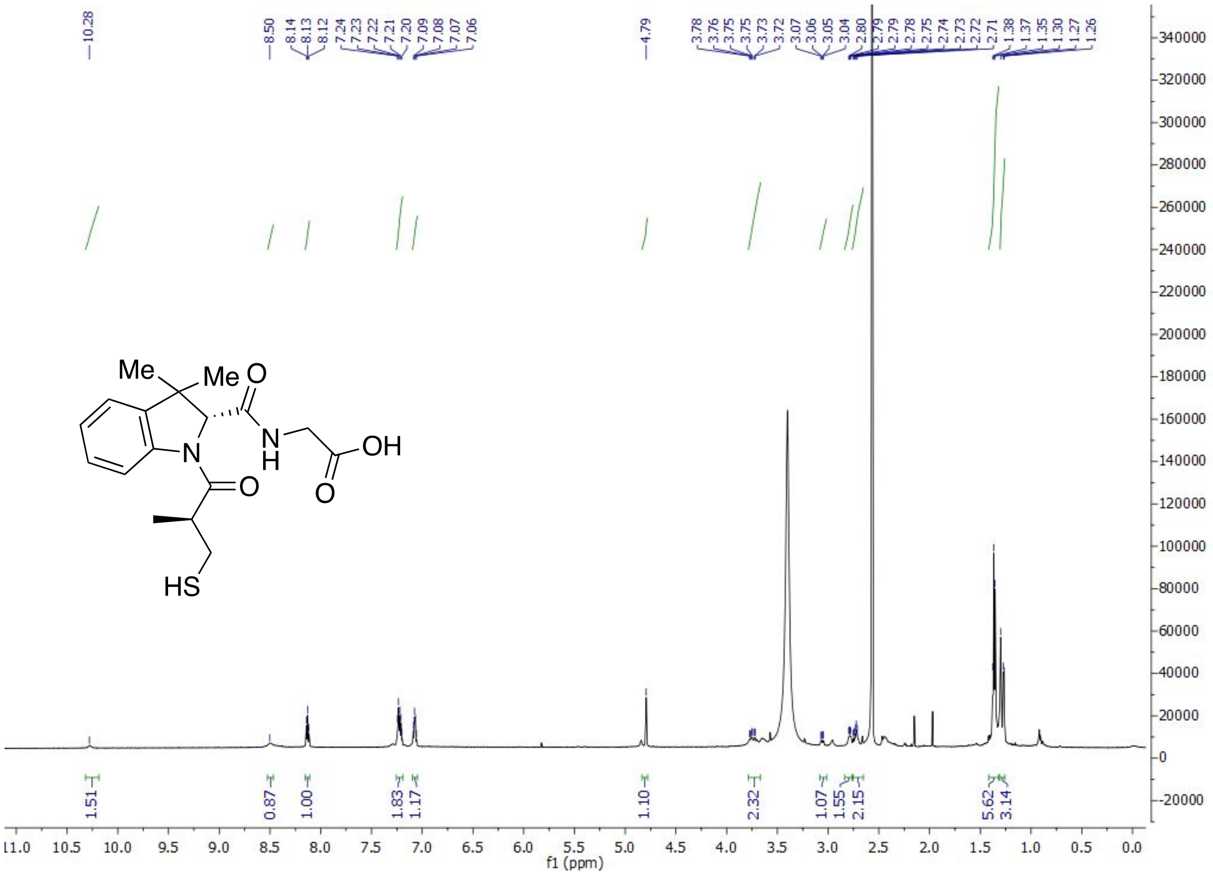

^{13}C NMR of compound **7b**

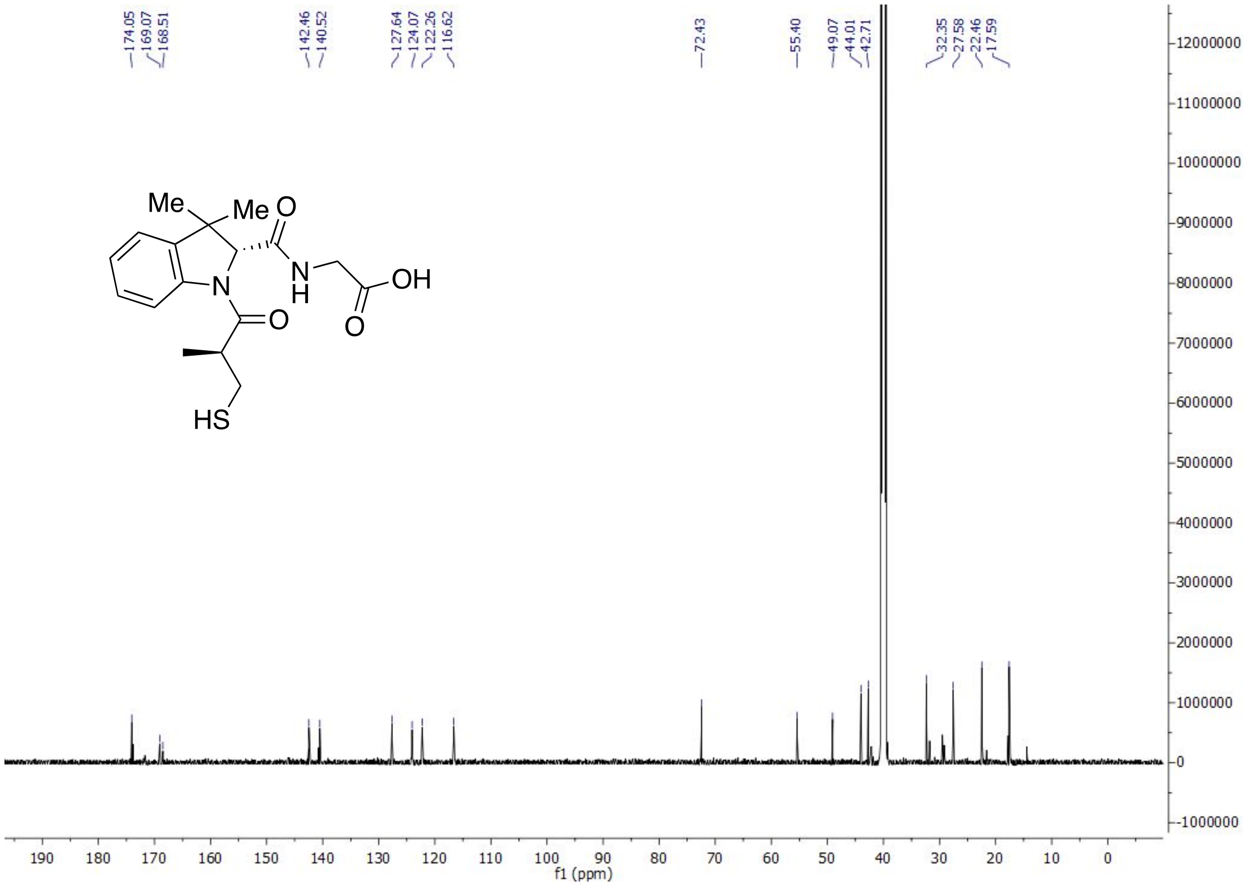

^{1}H NMR of compound *(S,S')*-**16b**

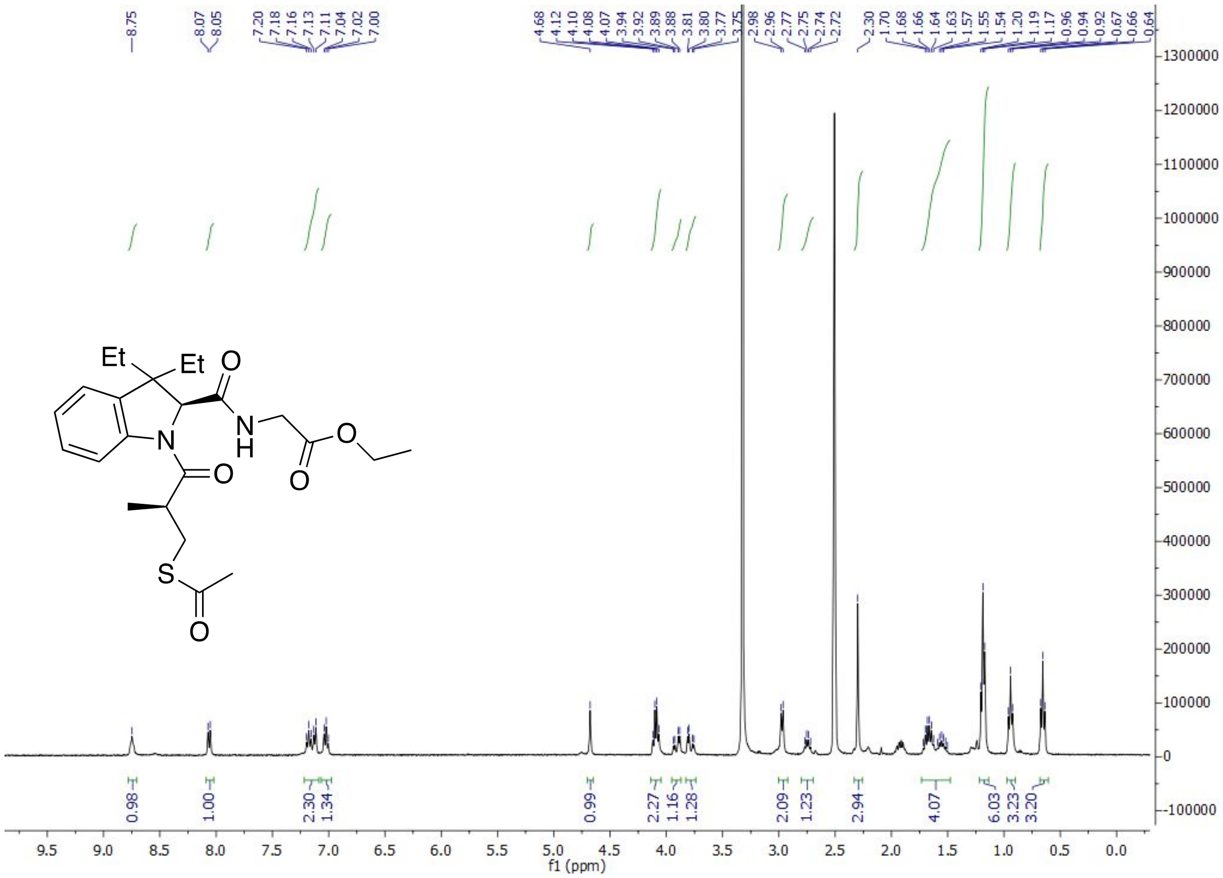

^{13}C NMR of compound *(S,S')*-**16b**

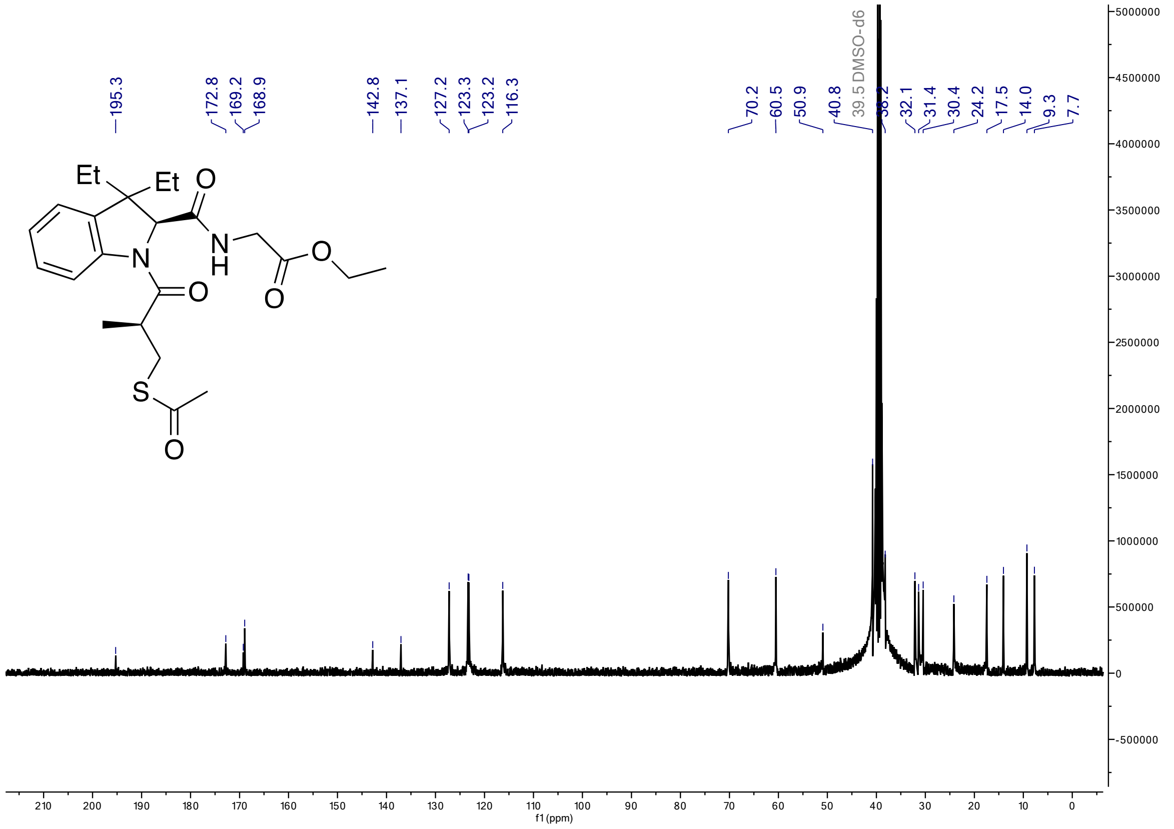

^{1}H NMR of compound *(S,R')*-**16b**

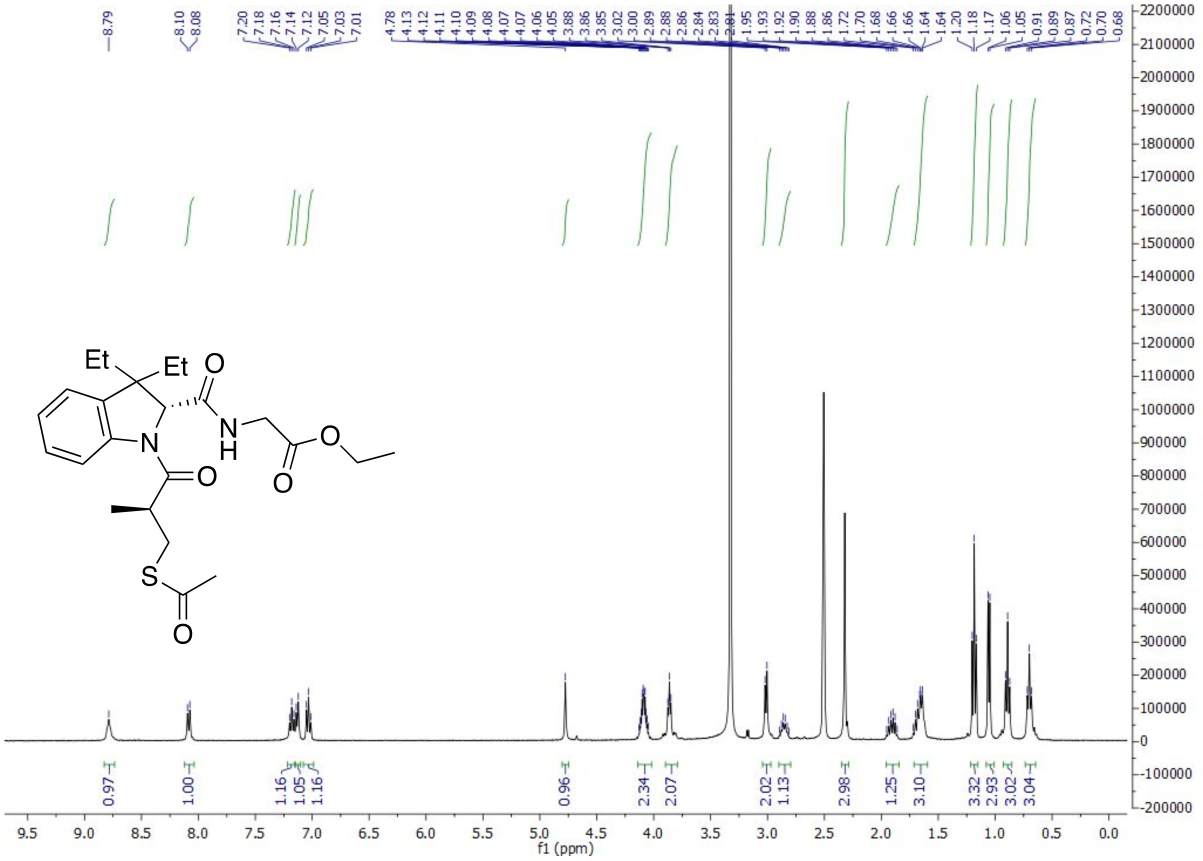

^{13}C NMR of compound *(S,R')*-**16b**

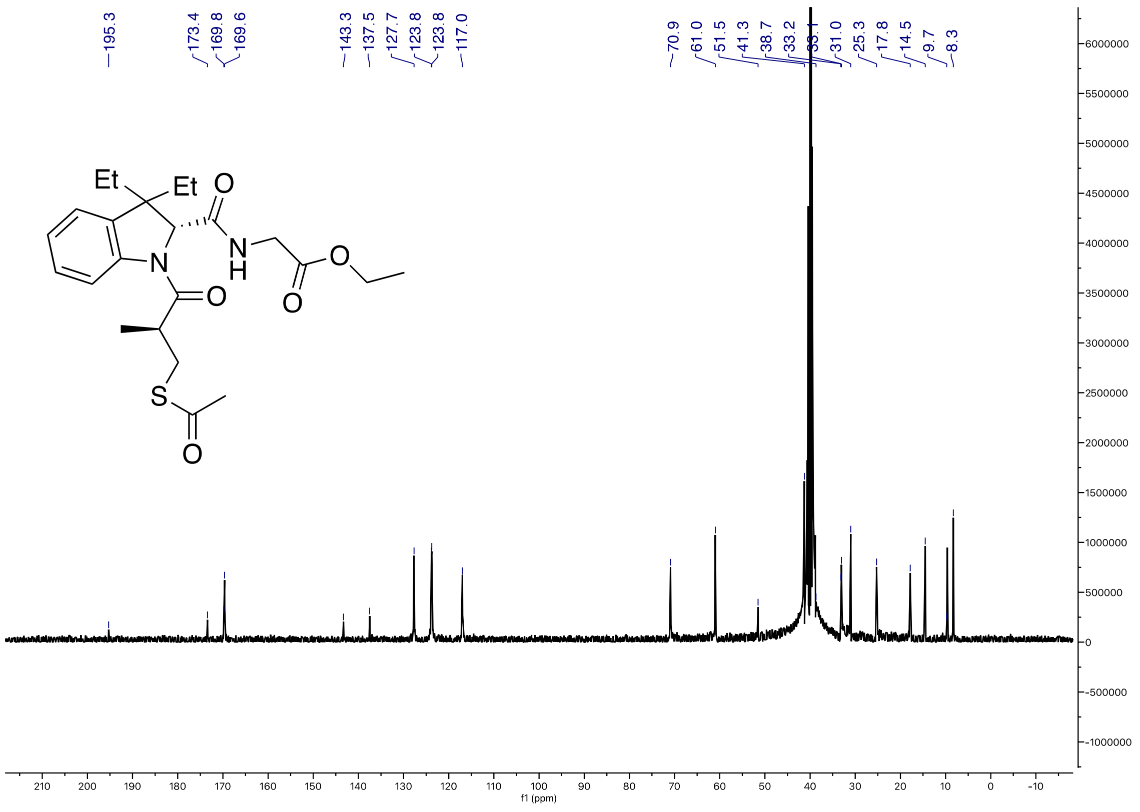

^{1}H NMR of compound **7c**

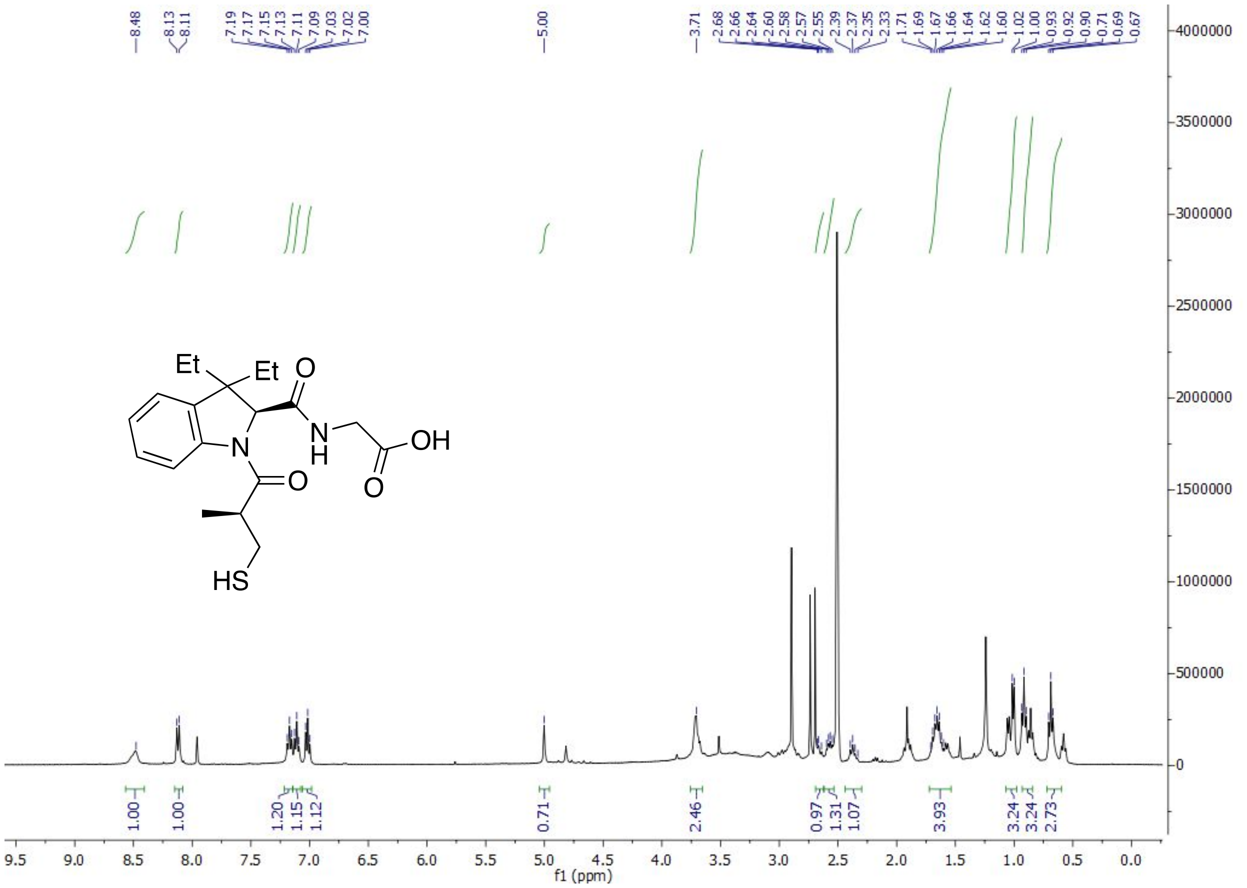

^{13}C NMR of compound **7c**

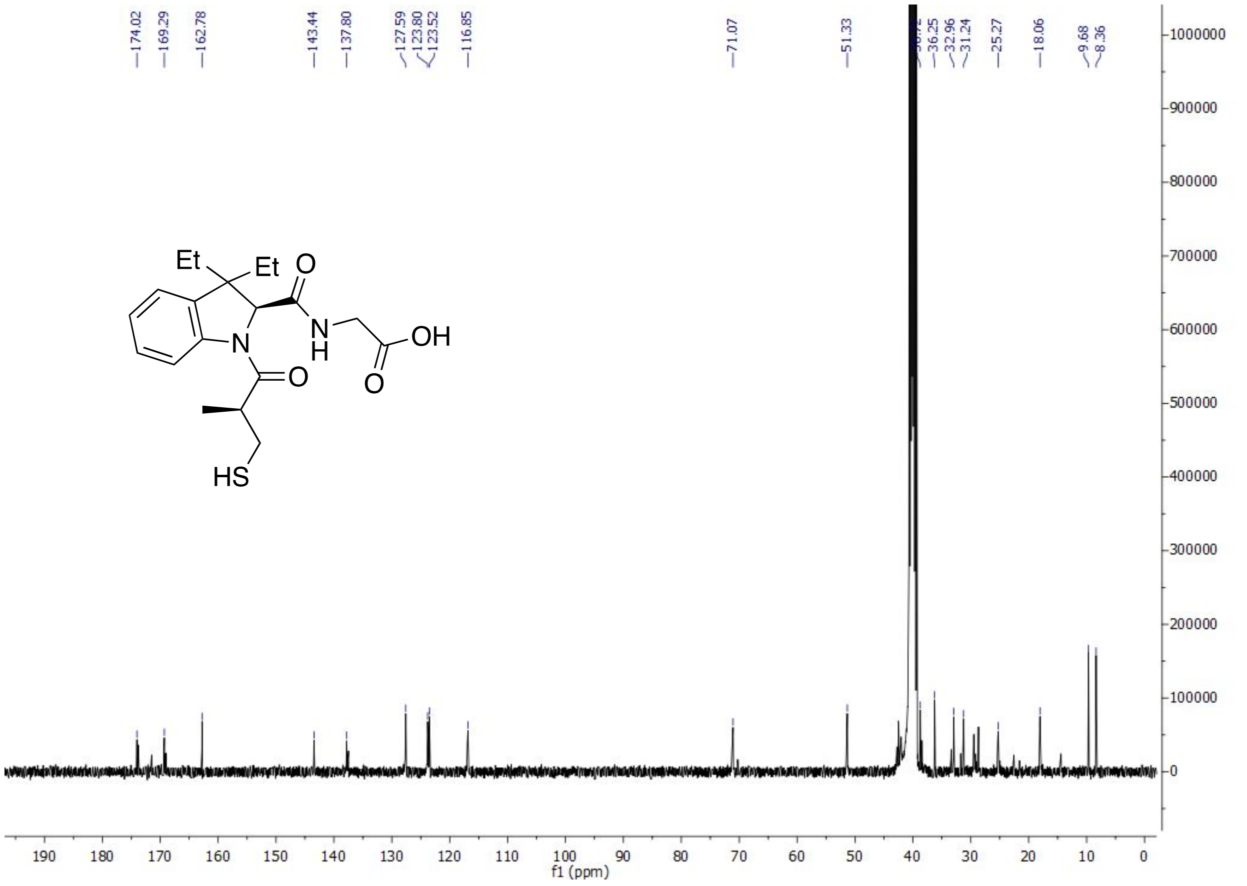

^{1}H NMR of compound **7d**

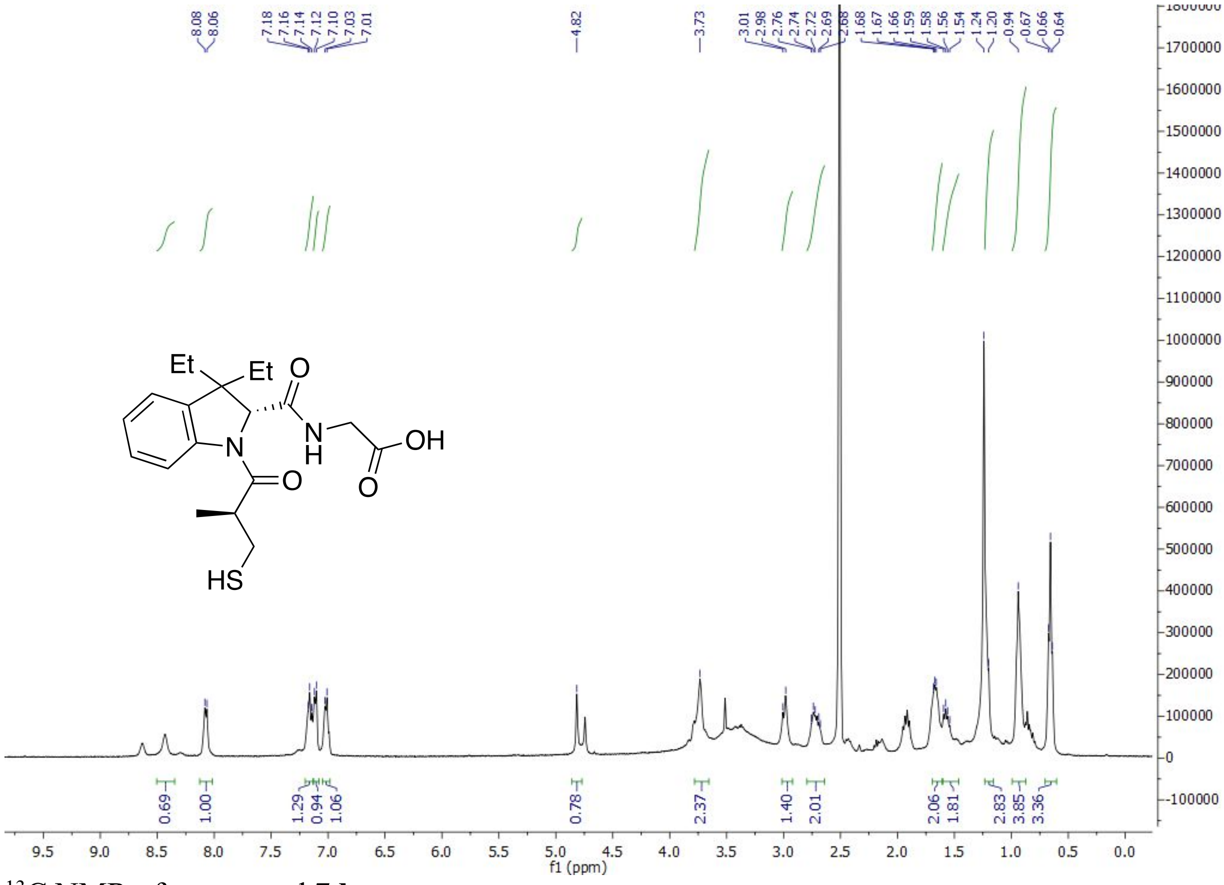

^{13}C NMR of compound **7d**

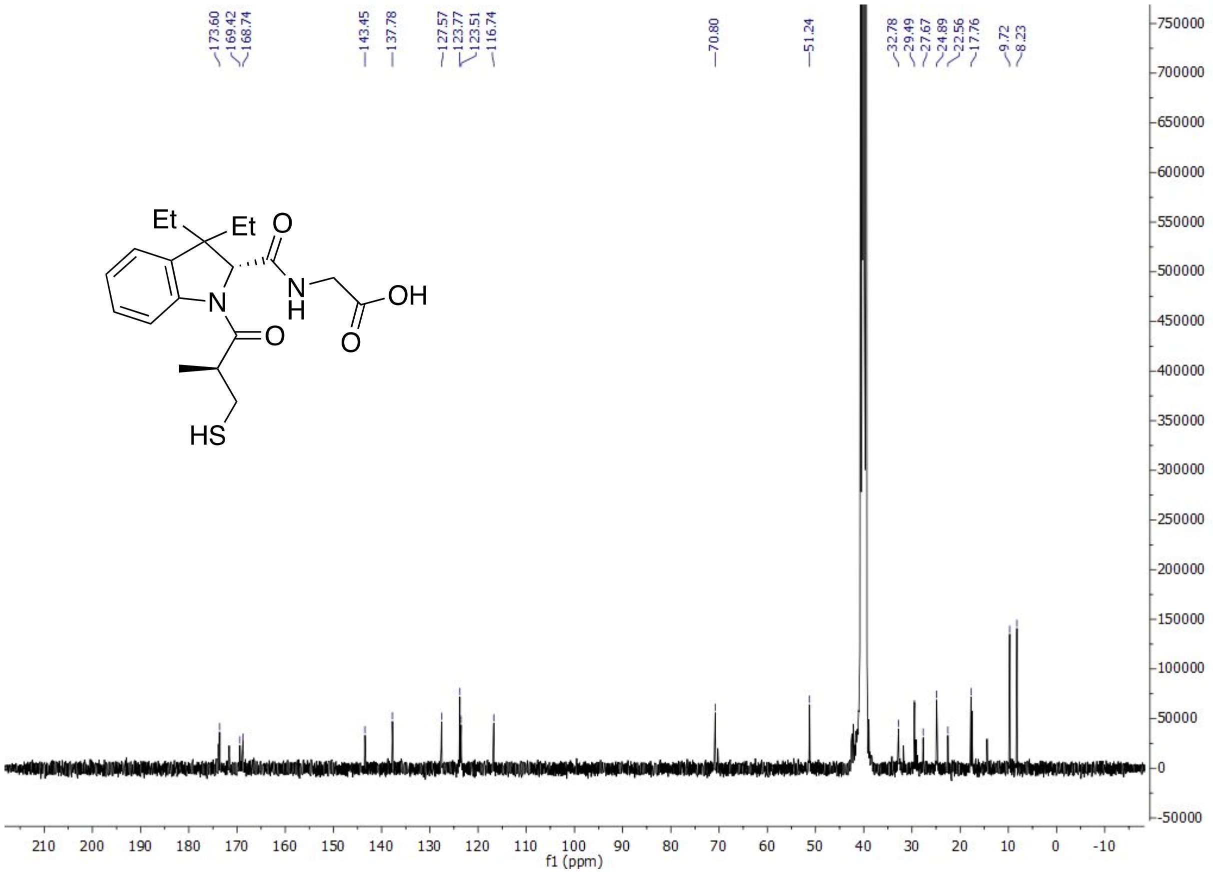

Stability studies for compound 7b

t = 0

t = 1h

6h

t = 12 h

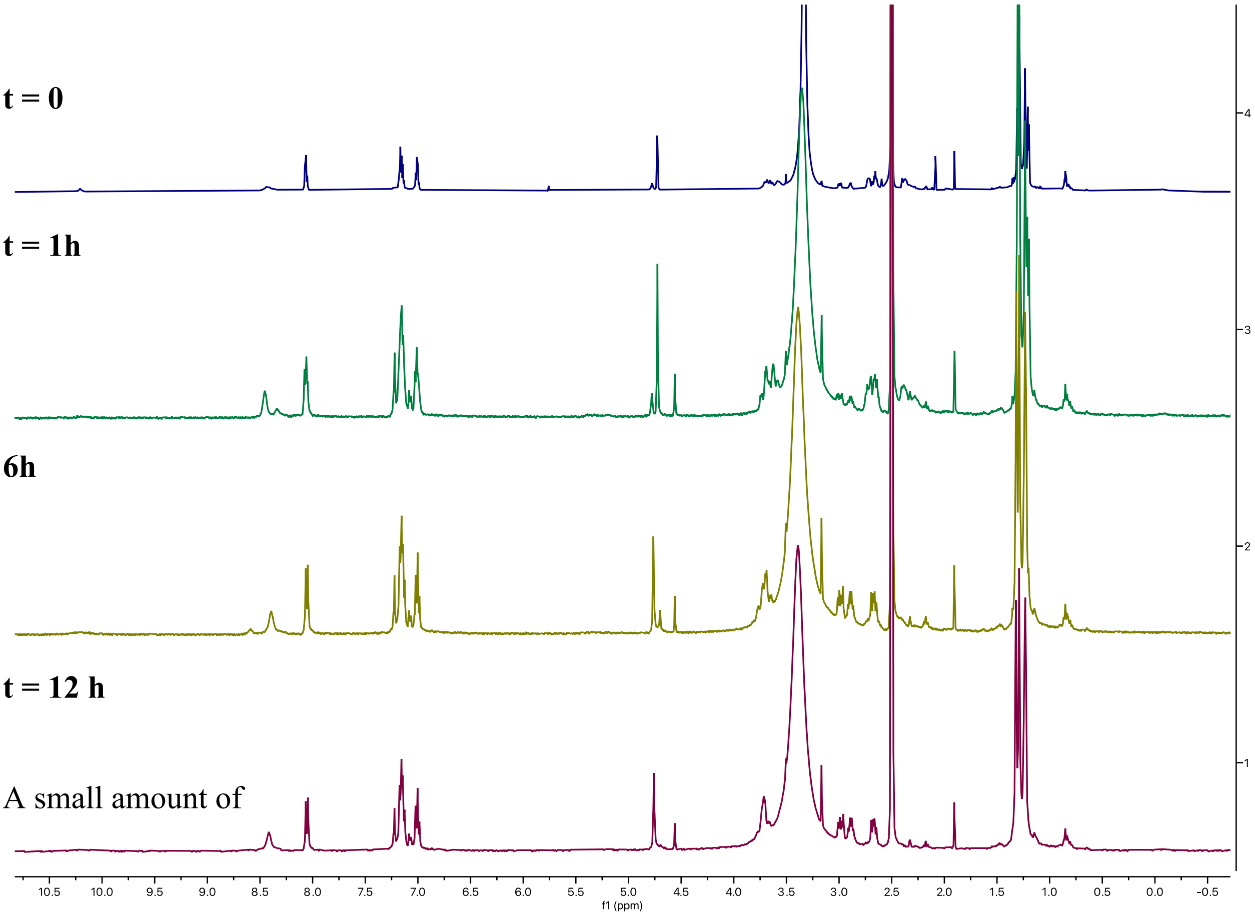

t =

A small amount of compound **7b** (2 mg) was placed in an NMR tube and monitored over time in the same DMSO-d_6 solution."

HPLC chromatograms

HPLC trace for compound **6a** (purity > 92%, compound not very stable)

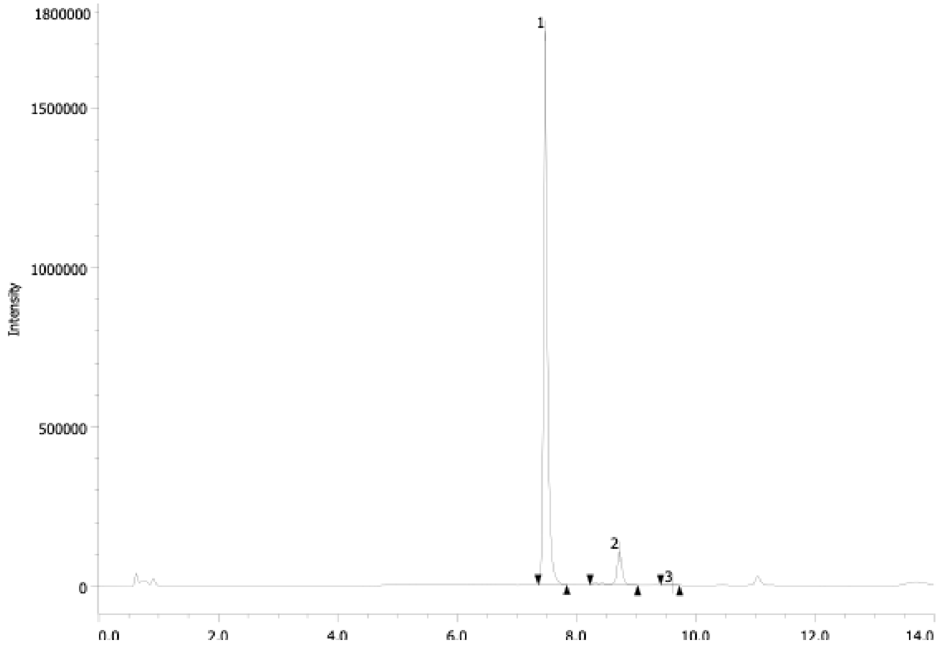

ESI full ms and UV of compound **6c** (purity > 95%)

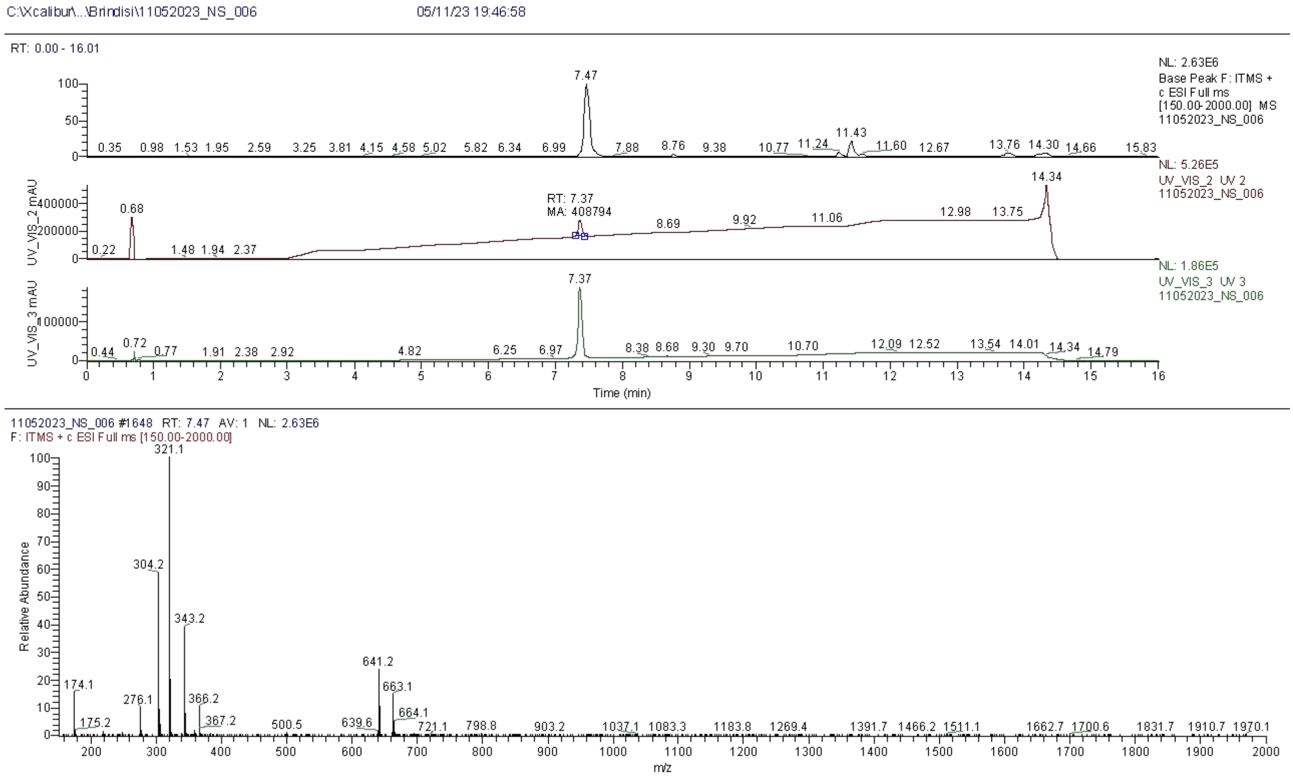

ESI full ms and UV of compound **6d** (purity > 95%)

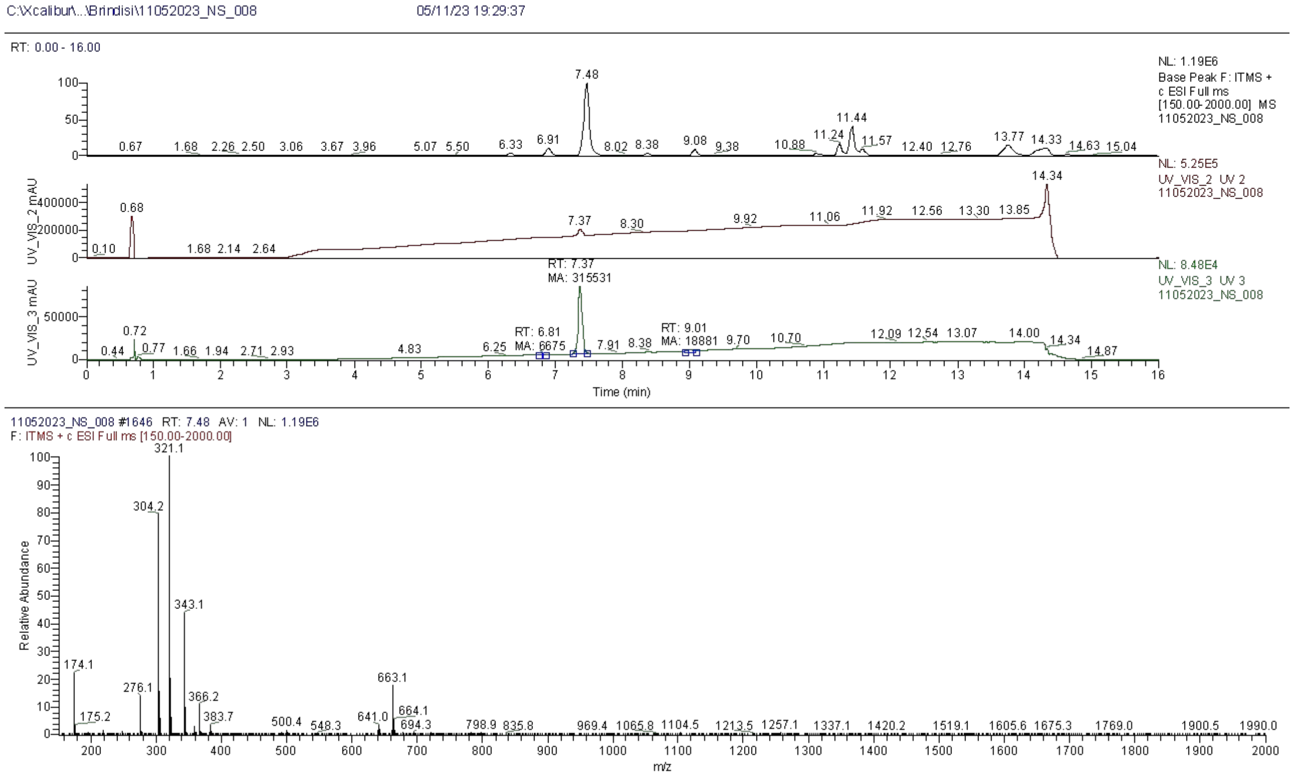

HPLC chromatogram of compound **6f** (purity > 95%)

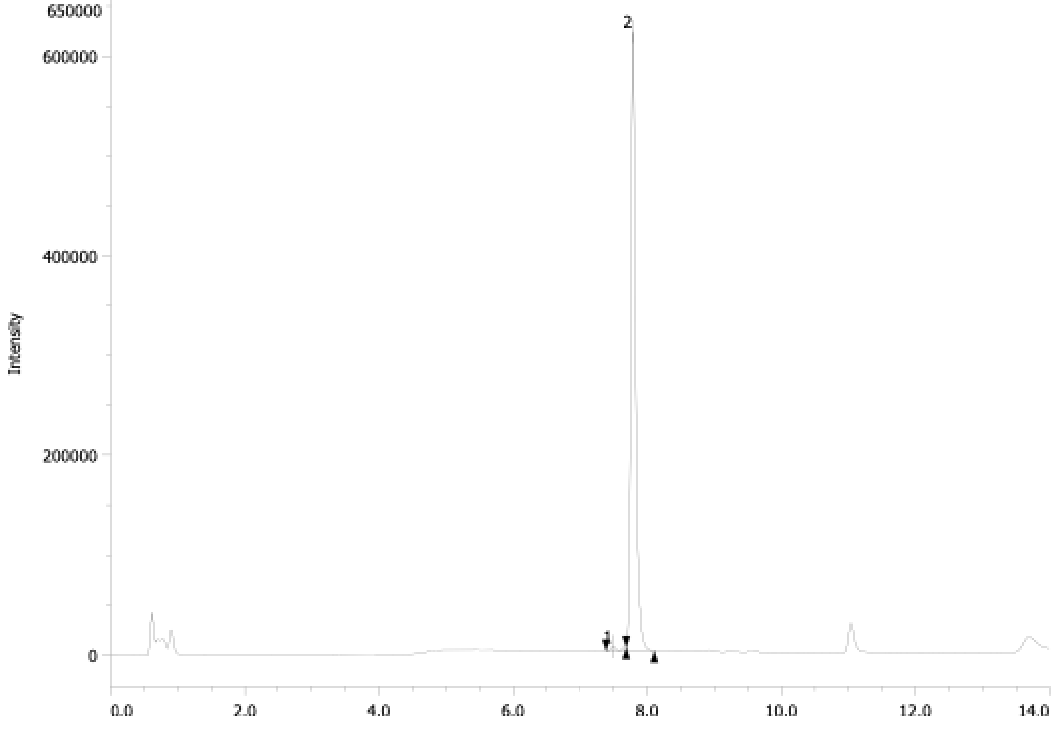

HRMS spectra

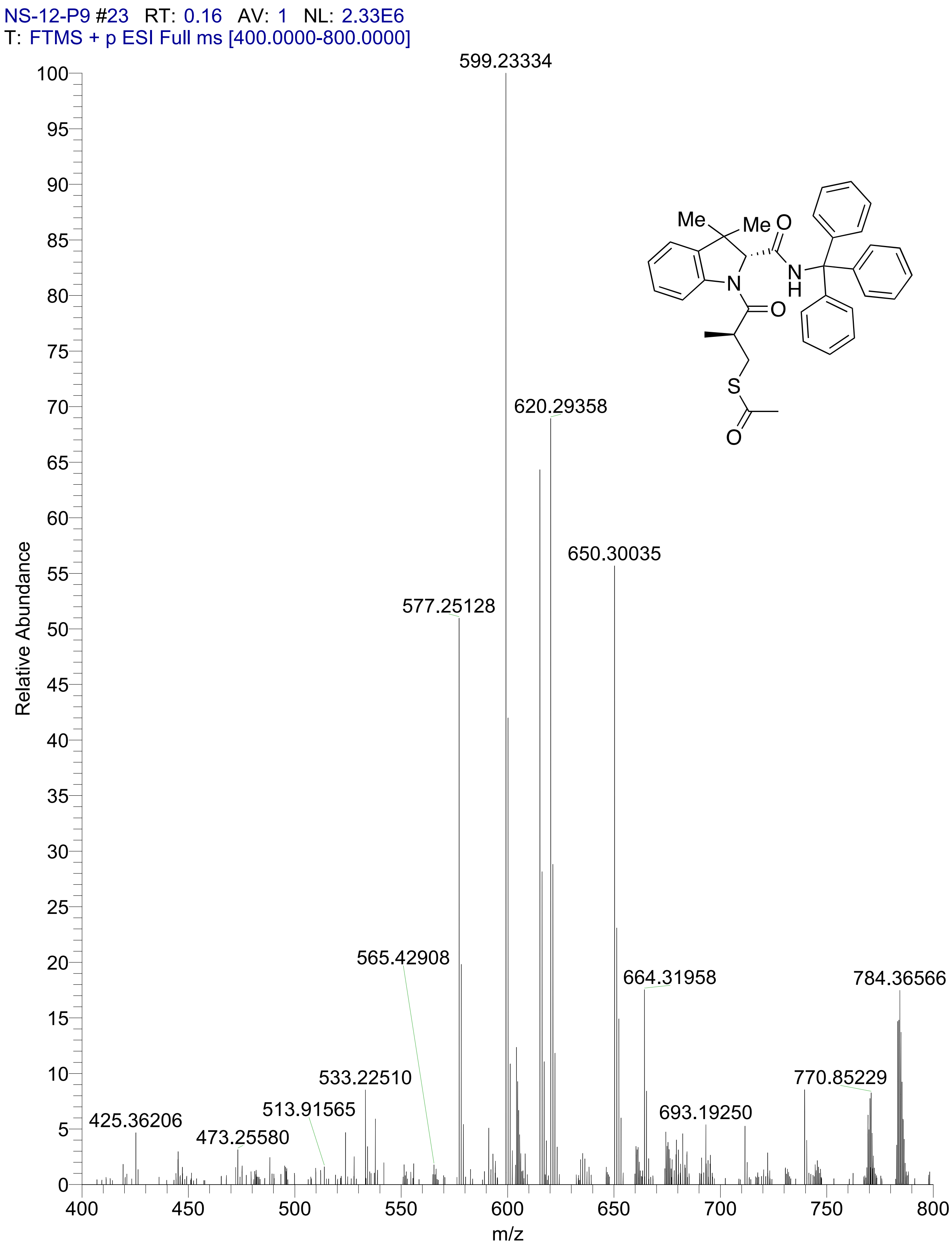

HRMS of compound *(S,R')*-**13a**

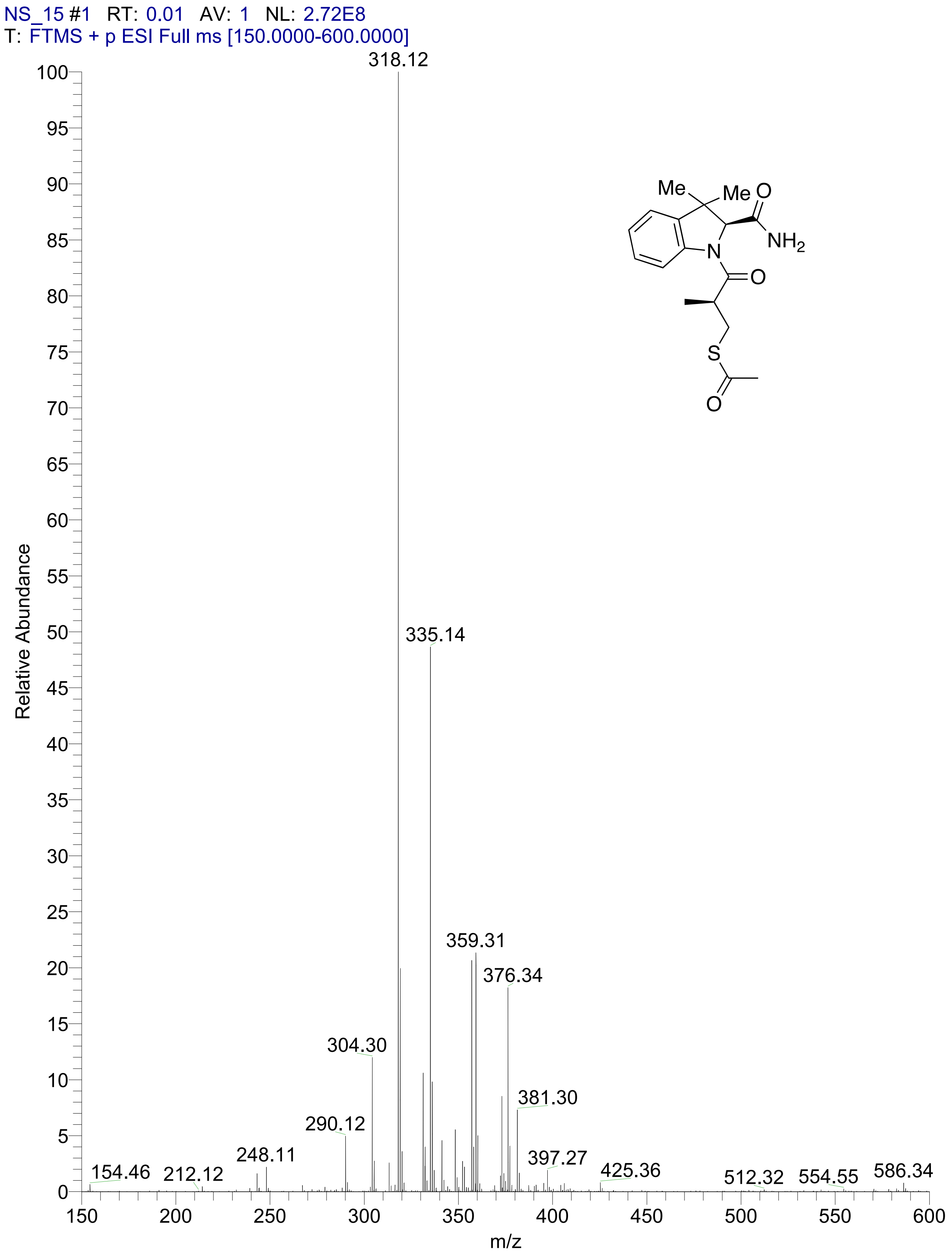

HRMS of compound *(S,S')*-**14a**

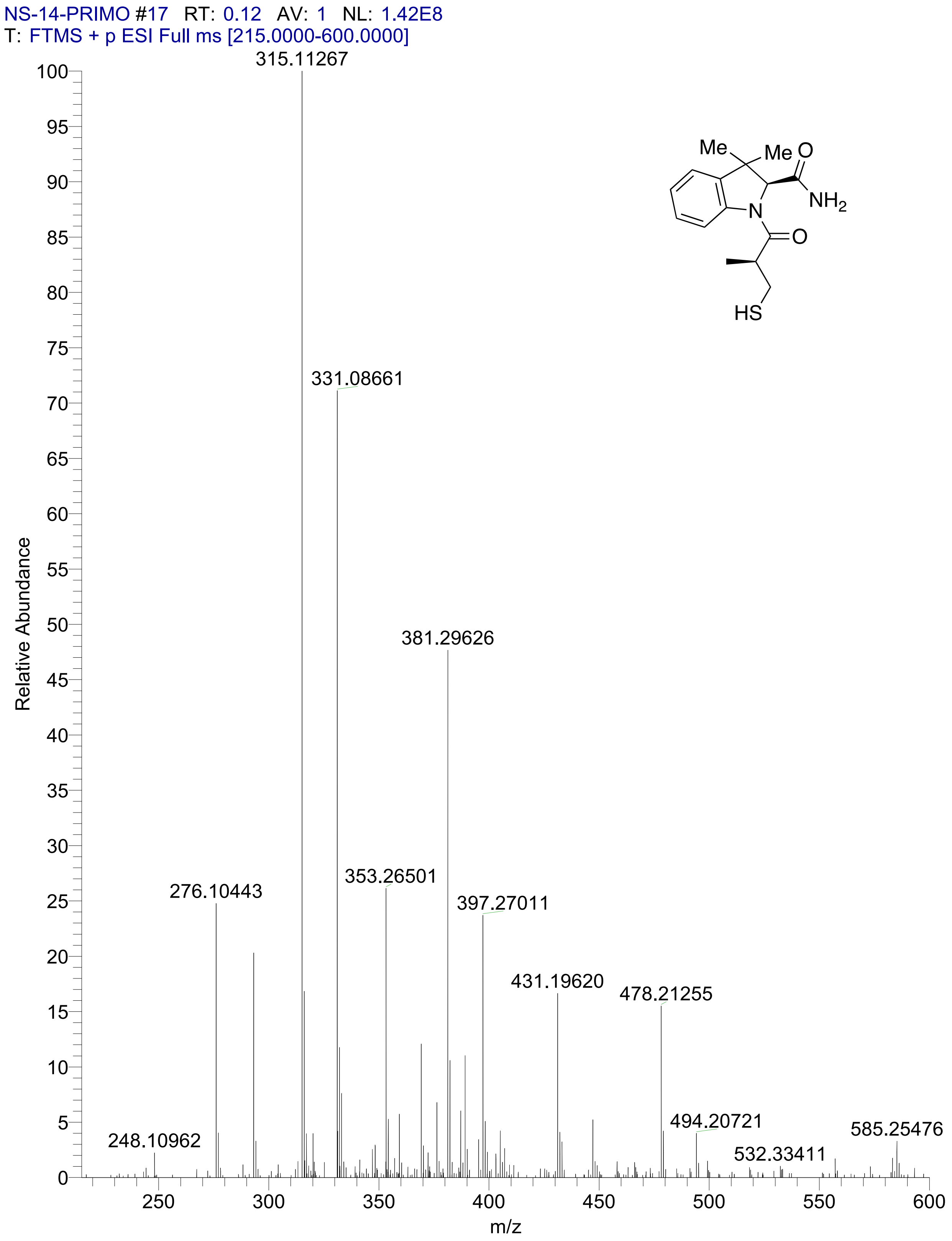

HRMS of compound **6a**

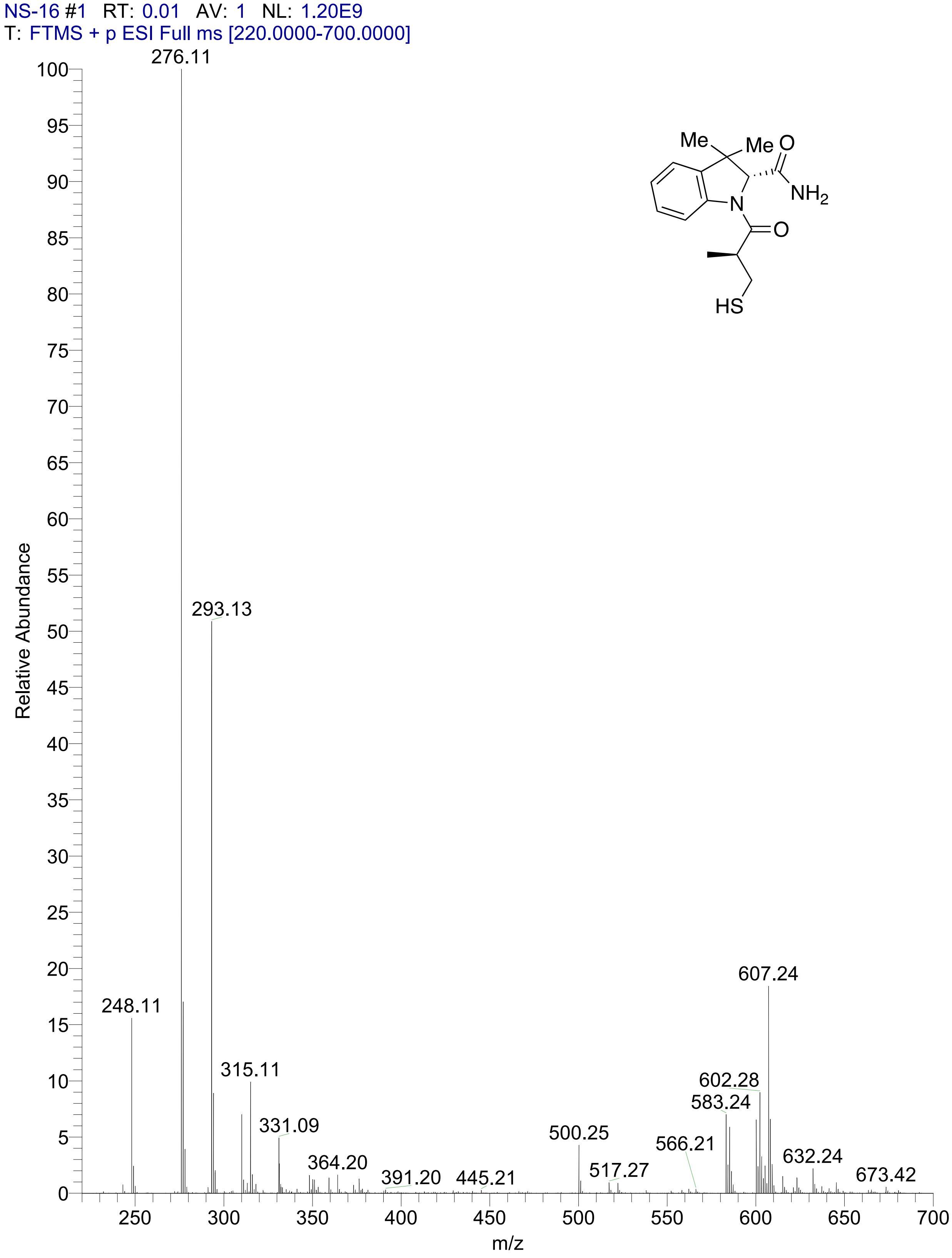

HRMS of compound **6b**

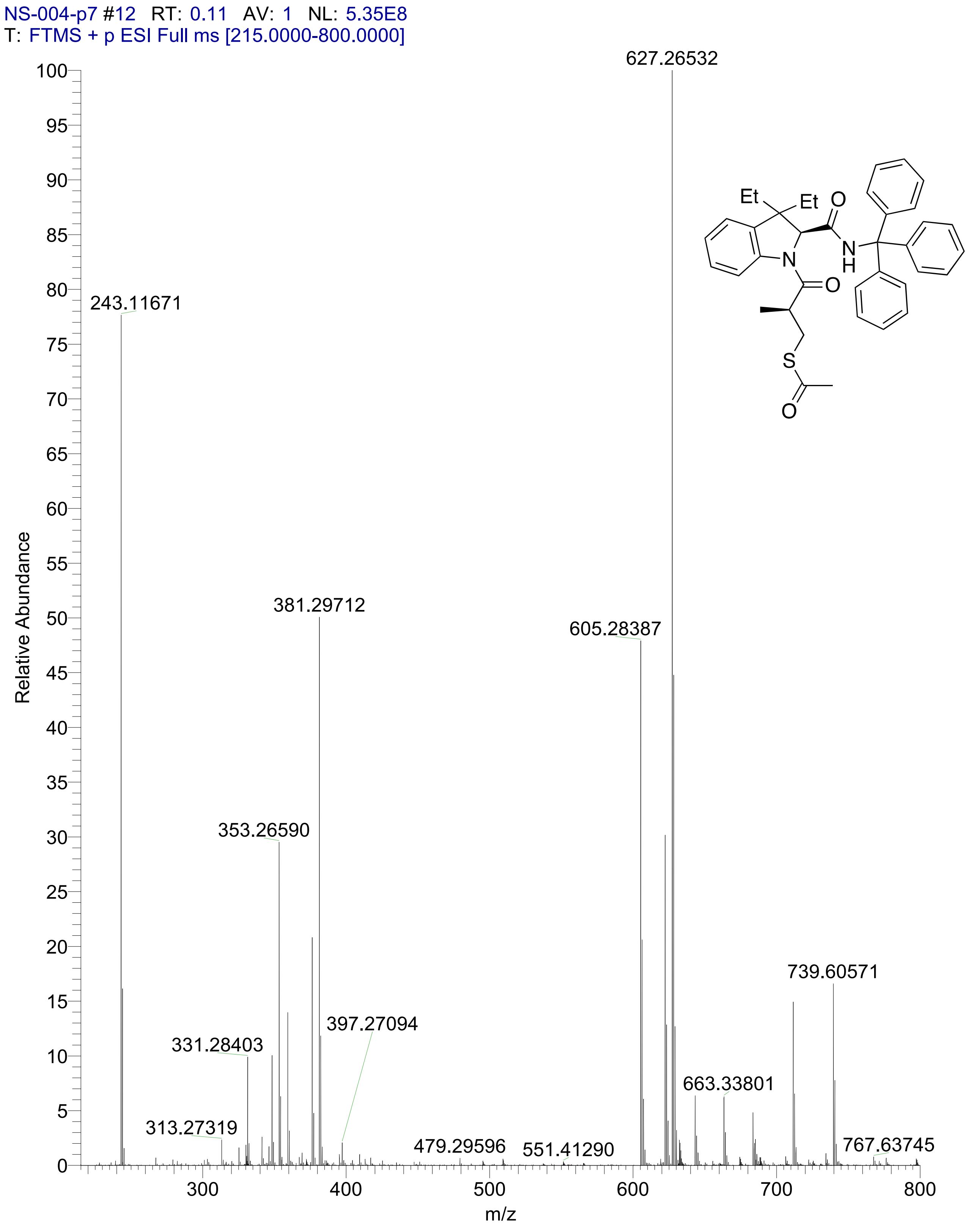

HRMS of compound *(S,S')*-**13b**

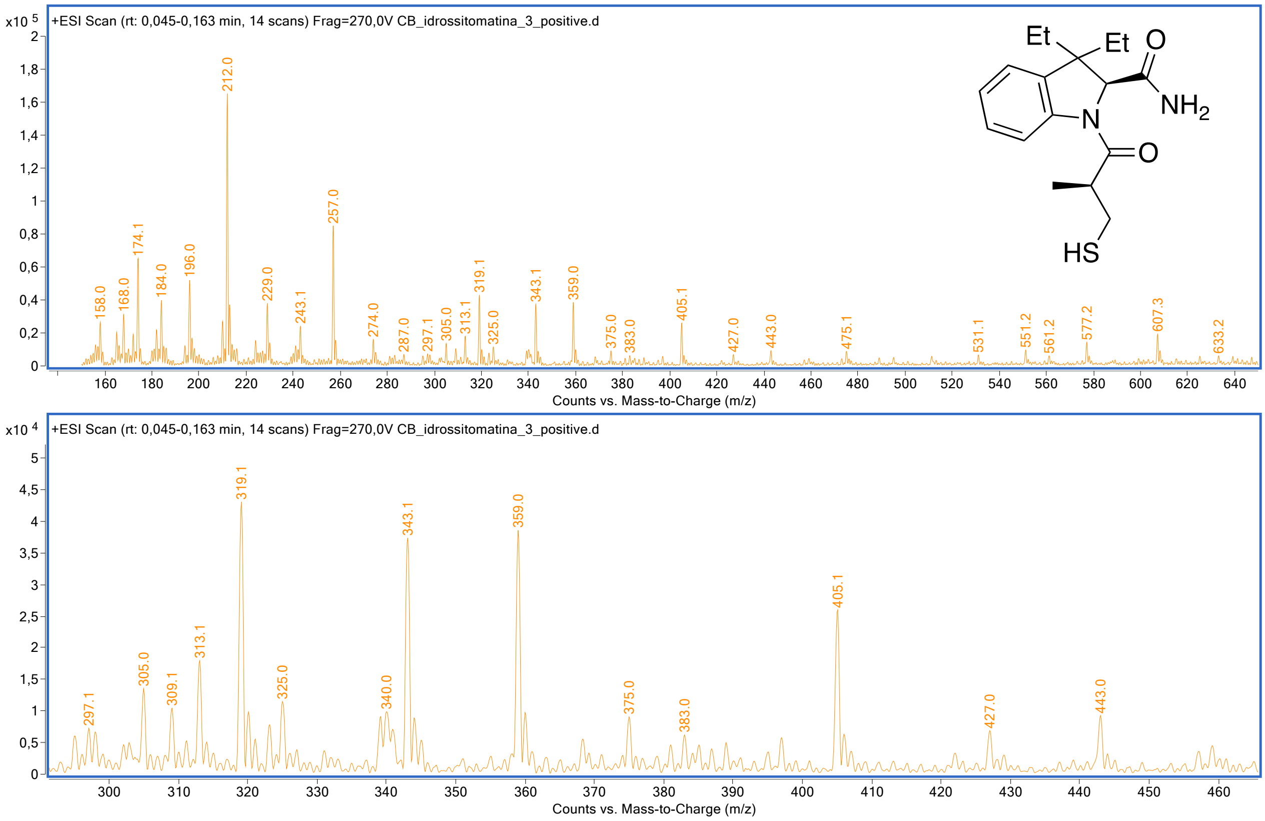

LC-MS of compound **6c**

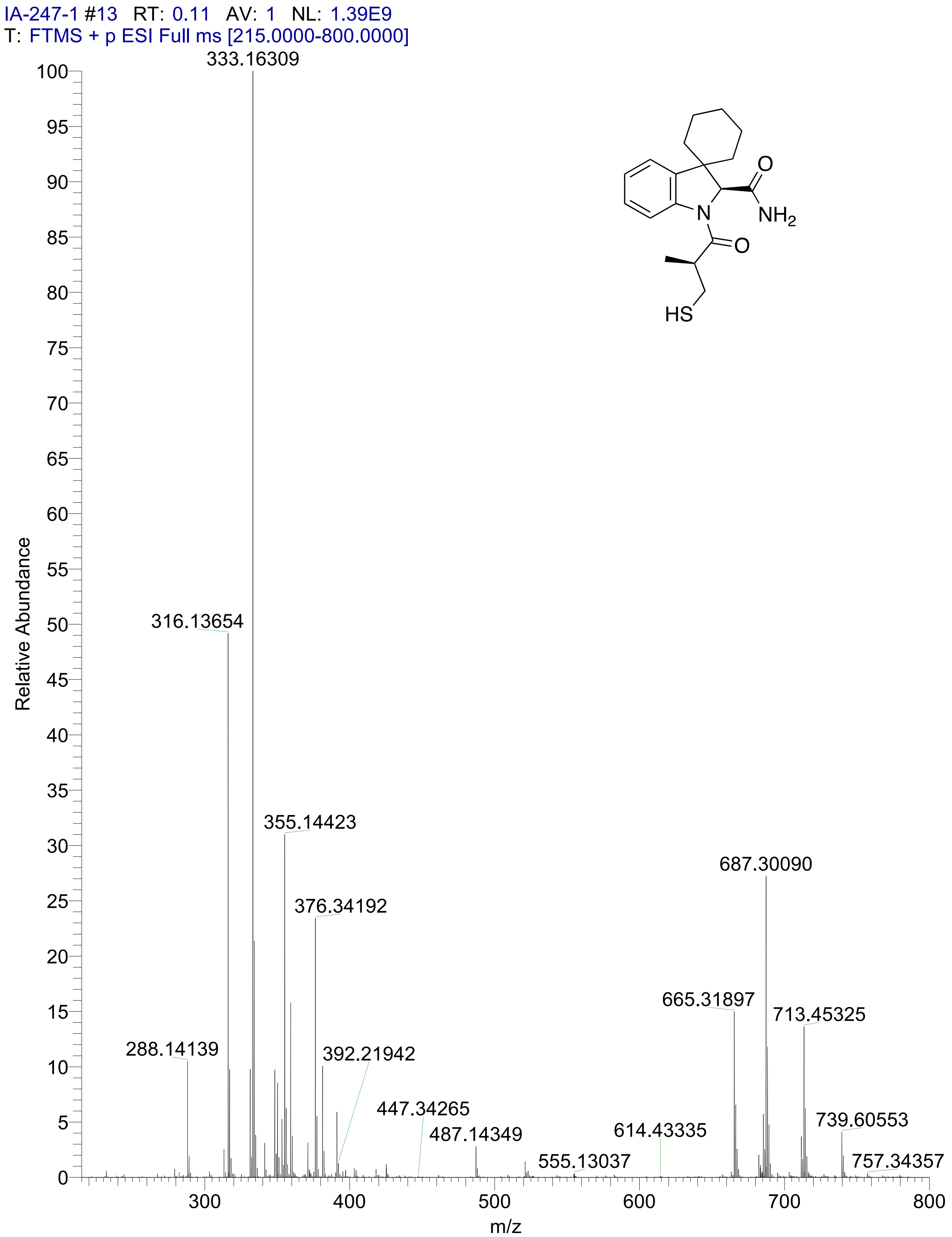

HRMS of compound **6e**

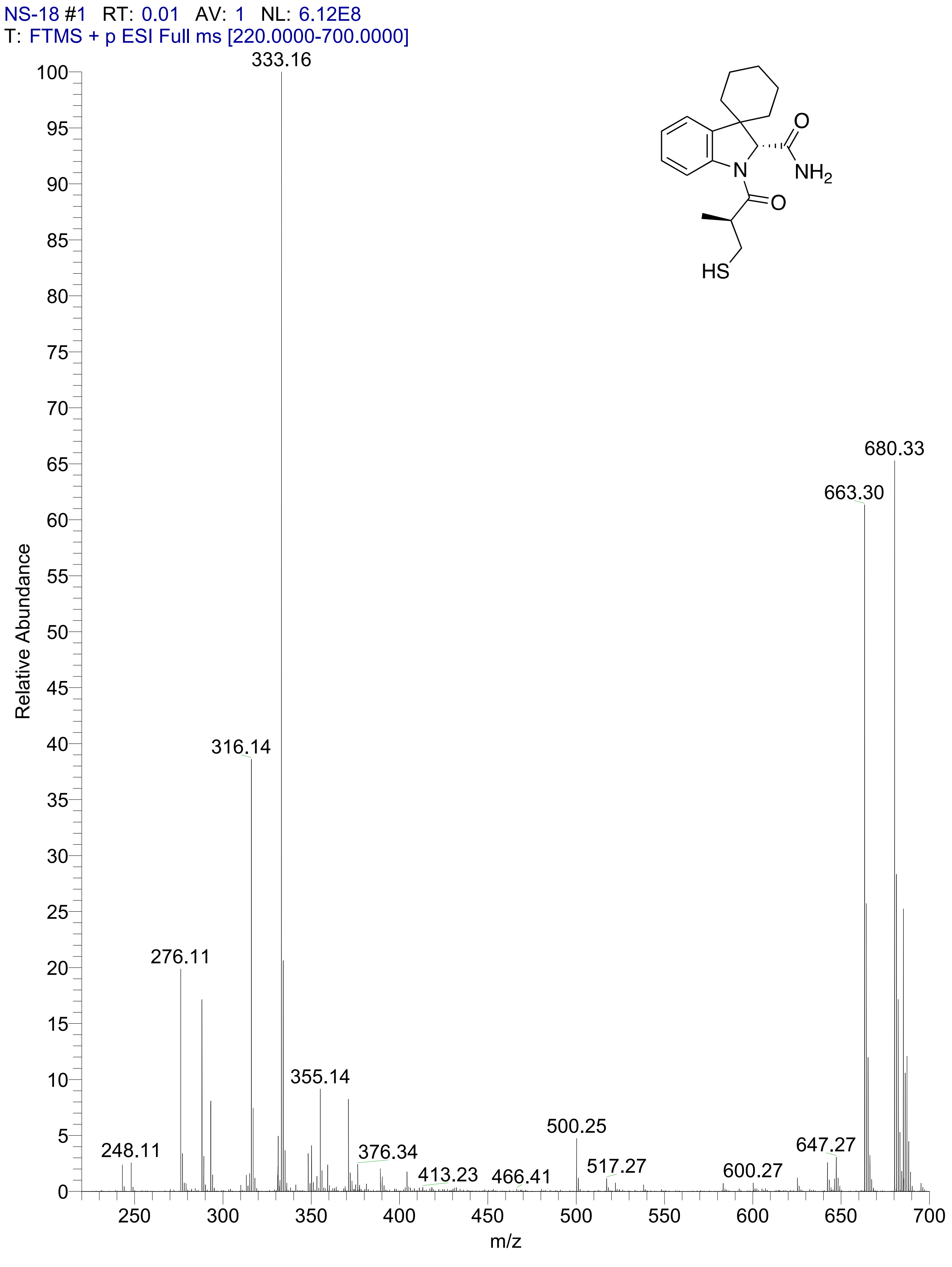

HRMS of compound **6f**

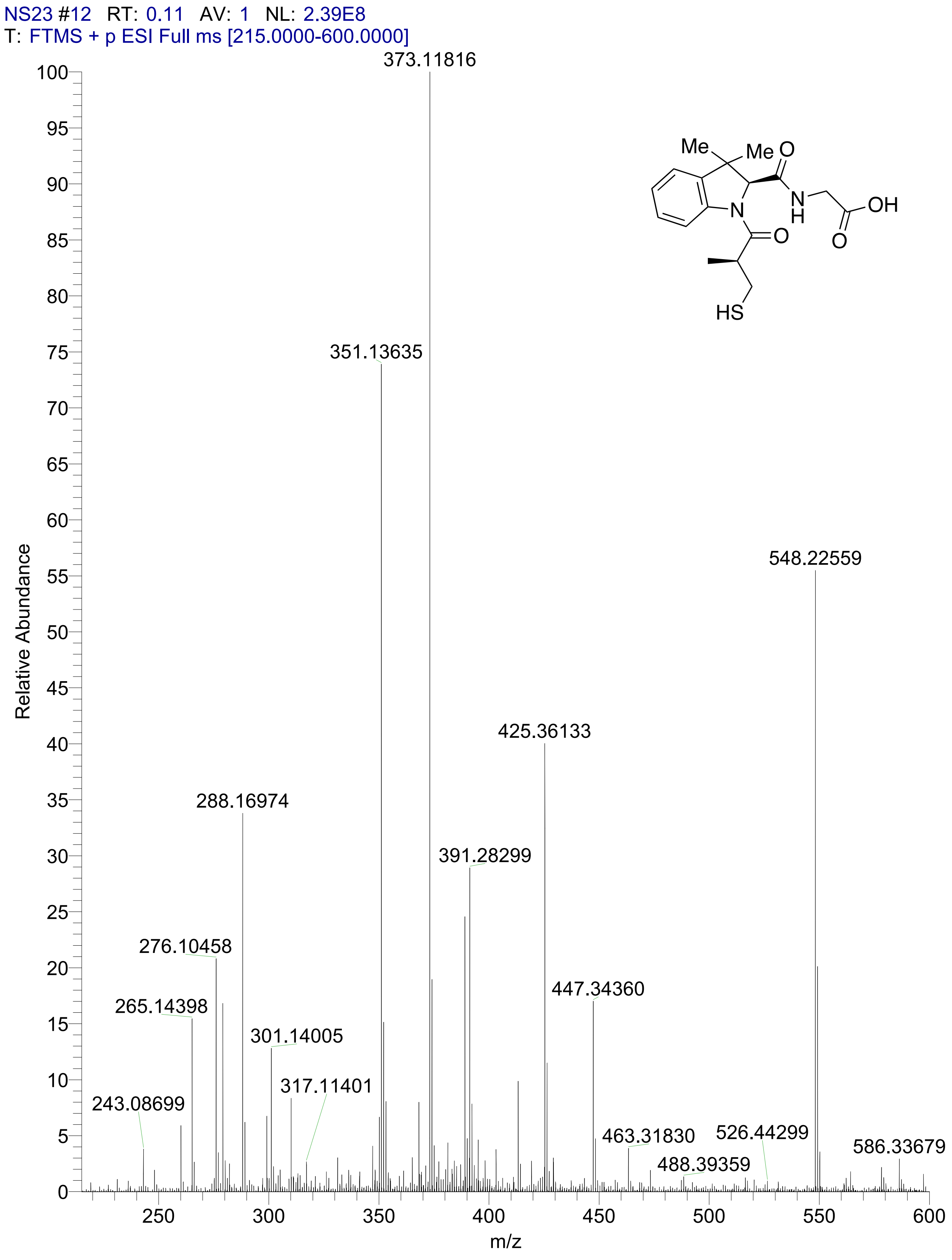

HRMS of compound **7a**

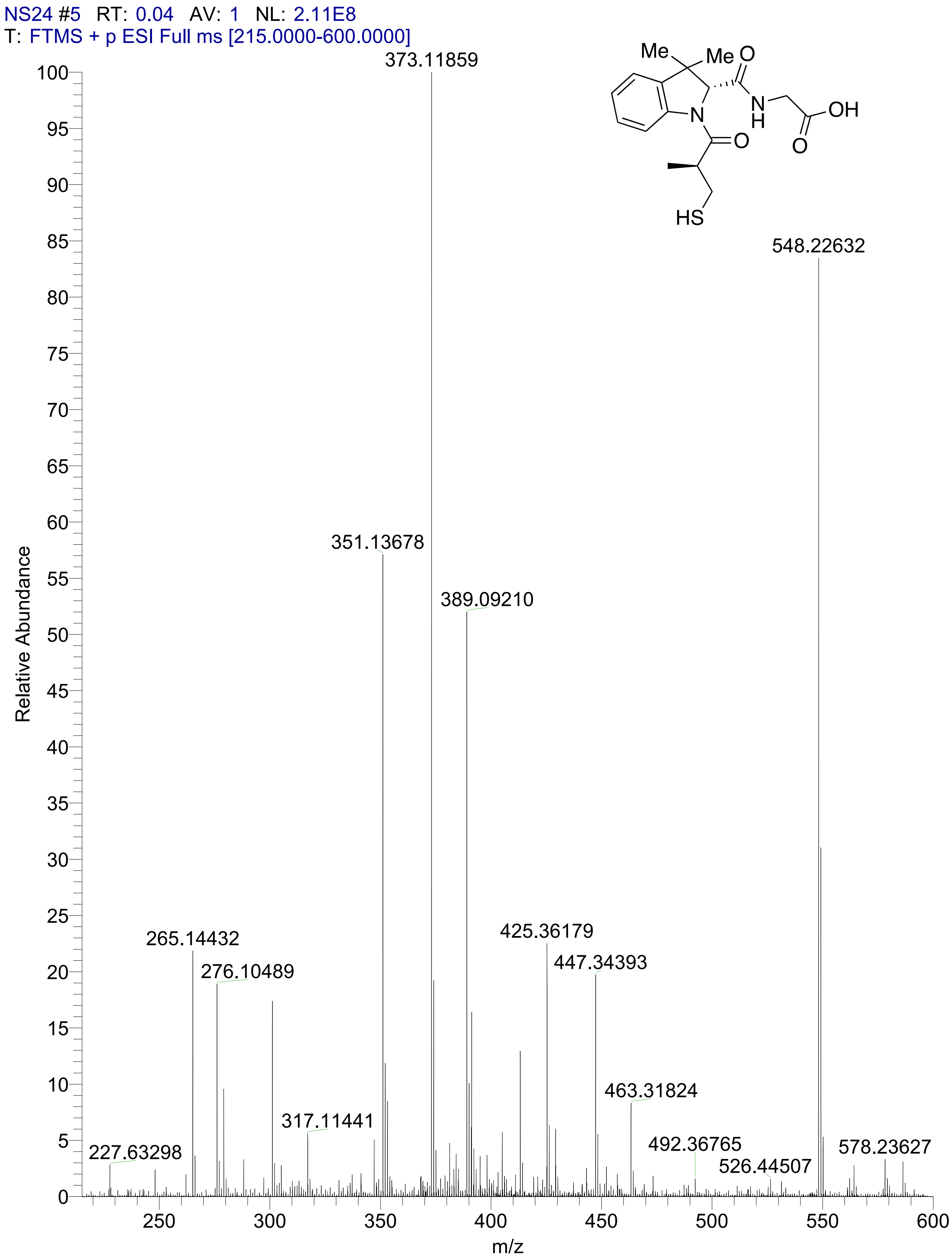

HRMS of compound **7b**

7c

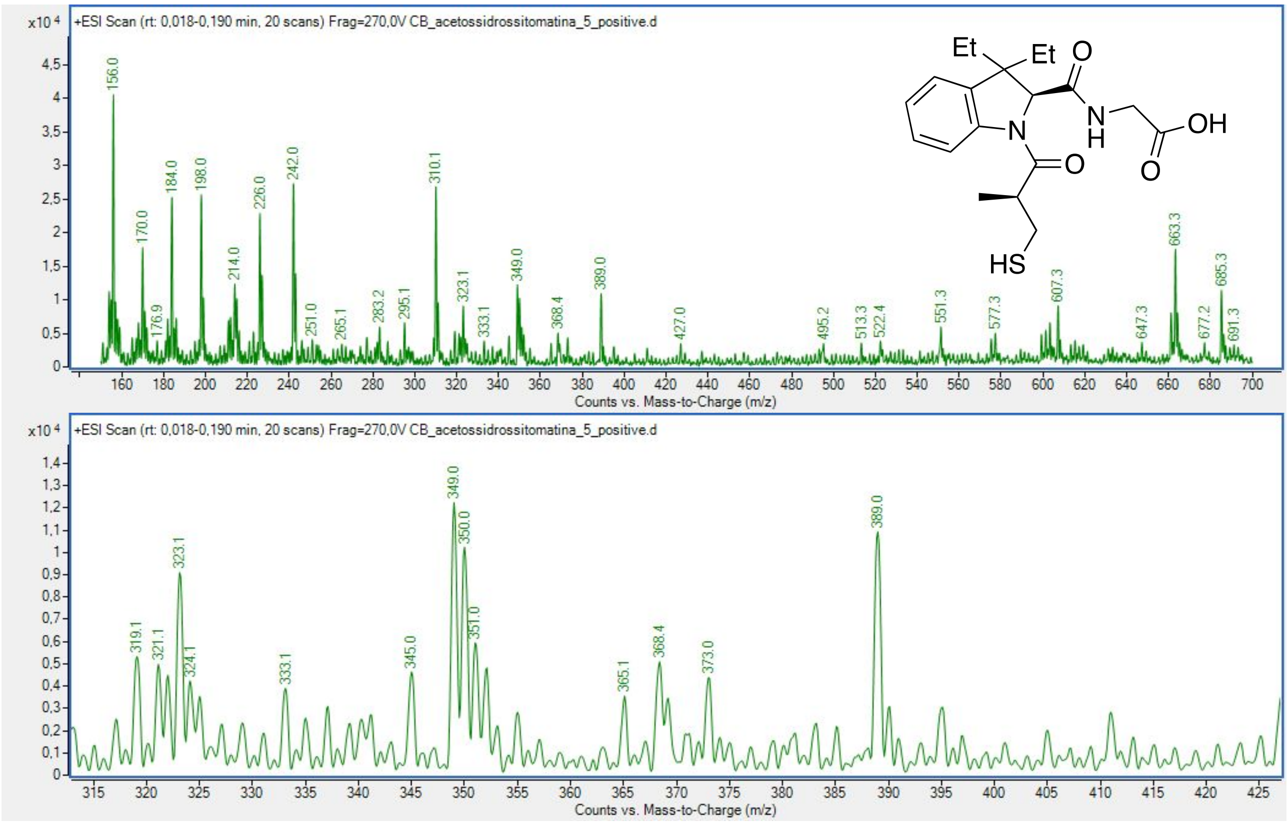

LC-MS of compound **7c**

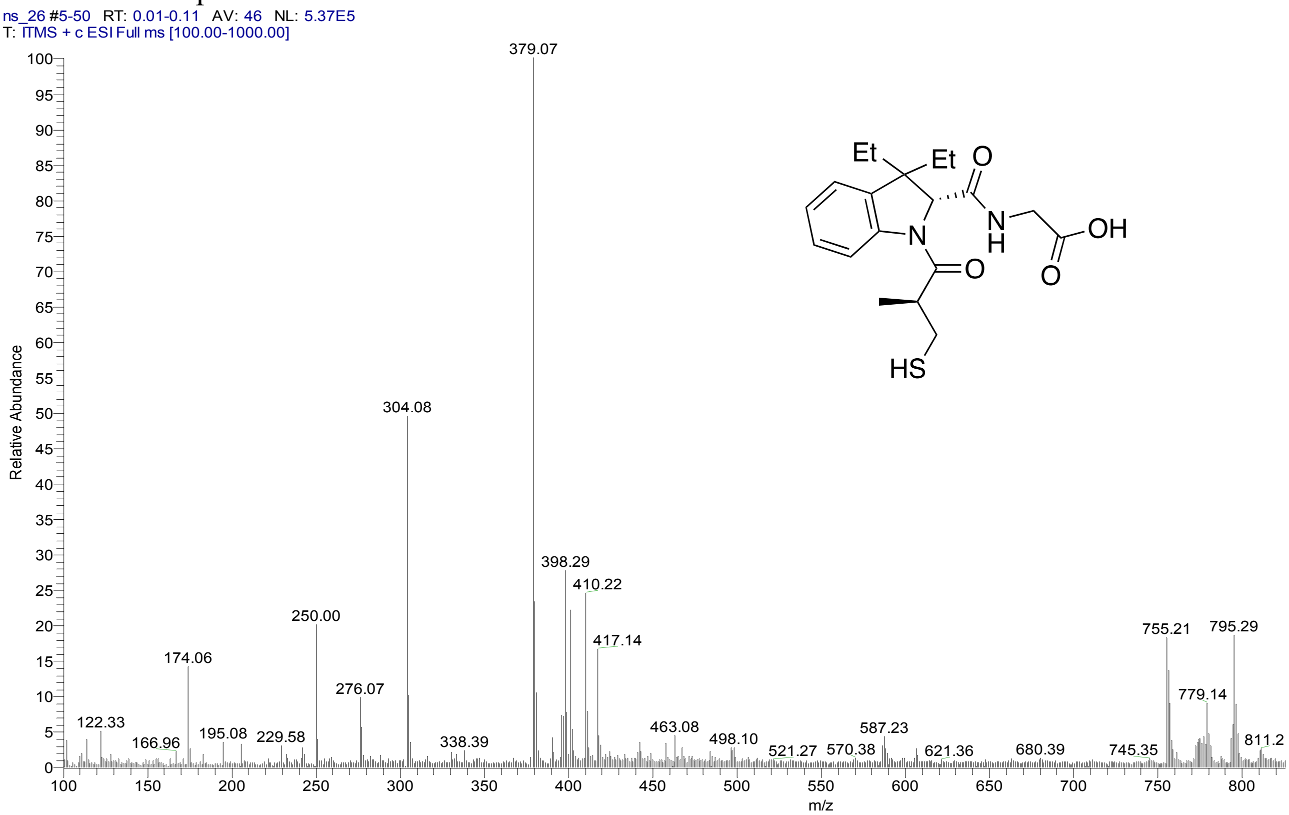

HRMS of compound **7d**

Stereochemical characterization

Table S1. Relative MM energies (ΔE_{MM}), absolute and relative SCF energies (E_{SCF} and ΔE_{SCF}), free energies (G) and Boltzmann populations based on SCF energies ($\chi_{E_{SCF}}$) for the lowest-energy conformers of compounds (*S*,*S*′)-**14b** and (*S*,*R*′)-**14b**, as obtained after DFT optimization at the B97D3/def2-TZVP/fit level (IEFPCM solvation model for methanol).

Conf. ID	ΔE_{MM} (kJ mol^{-1})	E_{SCF} (Ha)	G (Ha)	ΔE_{SCF} (kJ mol^{-1})	$\chi_{E_{SCF}}$ (%)
(*S*,*S*′)-**14b.03**	1.6899	−1472.90803625	−1472.548248	0.0000	33.5%
(*S*,*S*′)-**14b.42**	15.0582	−1472.90772428	−1472.548101	0.8191	24.0%
(*S*,*S*′)-**14b.33**	12.8152	−1472.90683149	−1472.547835	3.1631	9.3%
(*S*,*S*′)-**14b.49**	15.8009	−1472.90662810	−1472.547548	3.6971	7.5%
(*S*,*S*′)-**14b.21**	10.0458	−1472.90605259	−1472.546166	5.2081	4.1%
(*S*,*S*′)-**14b.11**	6.9902	−1472.90588771	−1472.546422	5.6410	3.4%
(*S*,*S*′)-**14b.29**	11.3094	−1472.90561350	−1472.546227	6.3609	2.6%
(*S*,*S*′)-**14b.10**	6.8450	−1472.90533276	−1472.546461	7.0980	1.9%
(*S*,*S*′)-**14b.01**	0.0000	−1472.90526190	−1472.546098	7.2841	1.8%
(*S*,*S*′)-**14b.54**	16.3088	−1472.90523888	−1472.546057	7.3445	1.7%
(*S*,*S*′)-**14b.06**	4.2401	−1472.90510896	−1472.546855	7.6856	1.5%
(*S*,*R*′)-**14b.12**	9.6253	−1472.90838283	−1472.553405	0.0000	39.6%
(*S*,*R*′)-**14b.06**	6.3199	−1472.90725816	−1472.546355	2.9528	12.0%
(*S*,*R*′)-**14b.07**	7.4797	−1472.90676518	−1472.547367	4.2471	7.1%
(*S*,*R*′)-**14b.08**	8.2534	−1472.90658443	−1472.546069	4.7217	5.9%
(*S*,*R*′)-**14b.01**	0.0000	−1472.90651554	−1472.547309	4.9026	5.5%
(*S*,*R*′)-**14b.23**	12.9223	−1472.90629037	−1472.547352	5.4938	4.3%
(*S*,*R*′)-**14b.46**	16.5950	−1472.90616962	−1472.546810	5.8108	3.8%
(*S*,*R*′)-**14b.02**	0.6004	−1472.90601821	−1472.547910	6.2083	3.2%
(*S*,*R*′)-**14b.10**	8.9479	−1472.90600187	−1472.547059	6.2512	3.2%
(*S*,*R*′)-**14b.38**	15.4143	−1472.90586857	−1472.546969	6.6012	2.8%
(*S*,*R*′)-**14b.60**	18.6799	−1472.90557590	−1472.545359	7.3696	2.0%
(*S*,*R*′)-**14b.15**	11.5834	−1472.90540631	−1472.545150	7.8149	1.7%

Table S2. Relative MM energies (ΔE_{MM}), absolute and relative SCF energies (E_{SCF} and ΔE_{SCF}) and free energies (G) for discarded conformers of compound (*S*,*S*')-**14b**, as obtained after DFT optimization at the B97D3/def2-TZVP/fit level (IEFPCM solvation model for methanol).

Conf. ID	ΔE_{MM} (kJ mol^{-1})	E_{SCF} (Ha)	G (Ha)	ΔE_{SCF} (kJ mol^{-1})	Notes
(*S*,*S*')-**14b.73**	19.1200	−1472.90497333	−1472.546509	8.0417	
(*S*,*S*')-**14b.02**	1.3753	−1472.90454186	−1472.545844	9.1745	
(*S*,*S*')-**14b.08**	6.3714	−1472.90436170	−1472.546314	9.6475	
(*S*,*S*')-**14b.66**	18.0916	−1472.90416821	−1472.545433	10.1555	
(*S*,*S*')-**14b.19**	9.8968	−1472.90402377	−1472.546245	10.5348	
(*S*,*S*')-**14b.12**	7.9764	−1472.90395614	−1472.546413	10.7123	
(*S*,*S*')-**14b.35**	12.8842	−1472.90378379	−1472.545940	11.1648	
(*S*,*S*')-**14b.39**	13.9599	−1472.90378290	−1472.545126	11.1672	
(*S*,*S*')-**14b.28**	11.1976	−1472.90364151	−1472.544390	11.5384	
(*S*,*S*')-**14b.13**	8.5584	−1472.90348758	−1472.543055	11.9425	
(*S*,*S*')-**14b.36**	12.9783	−1472.90338990	−1472.546128	12.1990	
(*S*,*S*')-**14b.47**	15.7490	−1472.90328912	−1472.542481	12.4636	
(*S*,*S*')-**14b.16**	9.0471	−1472.90322592	−1472.542599	12.6295	
(*S*,*S*')-**14b.55**	16.3381	−1472.90317771	−1472.545083	12.7561	
(*S*,*S*')-**14b.04**	3.9539	−1472.90313409	−1472.544736	12.8706	
(*S*,*S*')-**14b.34**	12.8767	−1472.90310815	−1472.544817	12.9387	
(*S*,*S*')-**14b.67**	18.0950	−1472.90292809	−1472.544883	13.4115	
(*S*,*S*')-**14b.75**	19.3359	−1472.90292311	−1472.545267	13.4245	
(*S*,*S*')-**14b.32**	12.4512	−1472.90291975	−1472.544516	13.4334	
(*S*,*S*')-**14b.41**	14.7536	−1472.90274601	−1472.543449	13.8895	
(*S*,*S*')-**14b.07**	4.7815	−1472.90274334	−1472.546331	13.8965	
(*S*,*S*')-**14b.22**	10.0575	−1472.90270590	−1472.544342	13.9948	
(*S*,*S*')-**14b.61**	17.0678	−1472.90259478	−1472.542391	14.2866	
(*S*,*S*')-**14b.23**	10.1295	−1472.90248777	−1472.544733	14.5675	
(*S*,*S*')-**14b.72**	18.6498	−1472.90244959	−1472.544020	14.6678	
(*S*,*S*')-**14b.63**	17.5720	−1472.90216266	−1472.544941	15.4211	
(*S*,*S*')-**14b.14**	8.8031	−1472.90203117	−1472.544101	15.7663	
(*S*,*S*')-**14b.53**	16.3055	−1472.90196717	−1472.543213	15.9344	
(*S*,*S*')-**14b.40**	14.7528	−1472.90194250	−1472.543638	15.9991	
(*S*,*S*')-**14b.48**	15.7942	−1472.90179627	−1472.543900	16.3831	
(*S*,*S*')-**14b.58**	16.9506	−1472.90170271	−1472.544268	16.6287	
(*S*,*S*')-**14b.74**	19.2569	−1472.90159328	−1472.542871	16.9160	
(*S*,*S*')-**14b.79**	19.9727	−1472.90146322	−1472.542752	17.2575	
(*S*,*S*')-**14b.52**	16.2030	−1472.90143726	−1472.541369	17.3256	
(*S*,*S*')-**14b.70**	18.2573	−1472.90139764	−1472.544033	17.4297	
(*S*,*S*')-**14b.59**	16.9749	−1472.90130761	−1472.543399	17.6660	
(*S*,*S*')-**14b.24**	10.4562	−1472.90125039	−1472.542304	17.8163	
(*S*,*S*')-**14b.26**	10.6868	−1472.90097633	−1472.542542	18.5358	
(*S*,*S*')-**14b.62**	17.4987	−1472.90093013	−1472.543483	18.6571	
(*S*,*S*')-**14b.76**	19.4150	−1472.90075356	−1472.542111	19.1207	
(*S*,*S*')-**14b.51**	16.0394	−1472.90070816	−1472.543265	19.2399	
(*S*,*S*')-**14b.77**	19.7405	−1472.90056607	−1472.542503	19.6130	
(*S*,*S*')-**14b.56**	16.3540	−1472.90044449	−1472.540784	19.9322	
(*S*,*S*')-**14b.46**	15.5770	−1472.90040077	−1472.541720	20.0469	
(*S*,*S*')-**14b.17**	9.4676	−1472.90032975	−1472.540586	20.2334	
(*S*,*S*')-**14b.80**	19.9999	−1472.90028694	−1472.541725	20.3458	
(*S*,*S*')-**14b.20**	9.9261	−1472.89999177	−1472.542312	21.1208	
(*S*,*S*')-**14b.68**	18.1109	−1472.89987365	−1472.541112	21.4309	
(*S*,*S*')-**14b.71**	18.4694	−1472.89973981	−1472.542683	21.7823	
(*S*,*S*')-**14b.50**	16.0000	−1472.89965809	−1472.541917	21.9969	
(*S*,*S*')-**14b.64**	17.7234	−1472.89958673	−1472.541702	22.1842	
(*S*,*S*')-**14b.37**	13.3629	−1472.89934909	−1472.540306	22.8081	
(*S*,*S*')-**14b.60**	17.0071	−1472.89894894	−1472.540983	23.8587	
(*S*,*S*')-**14b.78**	19.9158	−1472.89872990	−1472.540380	24.4338	
(*S*,*S*')-**14b.05**	4.1246	−1472.90526191	−1472.546106		clustered with (*S*,*S*')-**14b.01**
(*S*,*S*')-**14b.09**	6.7538	−1472.90454186	−1472.545842		clustered with (*S*,*S*')-**14b.02**
(*S*,*S*')-**14b.38**	13.4403	−1472.90395607	−1472.546405		clustered with (*S*,*S*')-**14b.12**
(*S*,*S*')-**14b.45**	15.4862	−1472.90378374	−1472.545943		clustered with (*S*,*S*')-**14b.35**
(*S*,*S*')-**14b.57**	16.7665	−1472.90378291	−1472.545130		clustered with (*S*,*S*')-**14b.39**
(*S*,*S*')-**14b.43**	15.1469	−1472.90331152	−1472.543255		imaginary frequency
(*S*,*S*')-**14b.15**	8.9236	−1472.90313408	−1472.544741		clustered with (*S*,*S*')-**14b.04**
(*S*,*S*')-**14b.69**	18.1514	−1472.90310798	−1472.544834		clustered with (*S*,*S*')-**14b.34**
(*S*,*S*')-**14b.44**	15.4833	−1472.90274601	−1472.543421		clustered with (*S*,*S*')-**14b.41**
(*S*,*S*')-**14b.25**	10.4838	−1472.90274324	−1472.546367		clustered with (*S*,*S*')-**14b.07**

Conf. ID	ΔE_{MM} (kJ mol⁻¹)	E_{SCF} (Ha)	G (Ha)	ΔE_{SCF} (kJ mol⁻¹)	Notes
(*S*,*S*')-**14b.18**	9.7056	−1472.90203124	−1472.544096		clustered with (*S*,*S*')-**14b.14**
(*S*,*S*')-**14b.31**	12.2788	−1472.90203121	−1472.544100		clustered with (*S*,*S*')-**14b.14**
(*S*,*S*')-**14b.65**	18.0849	−1472.90130762	−1472.543403		clustered with (*S*,*S*')-**14b.59**
(*S*,*S*')-**14b.30**	11.5985	−1472.89973765	−1472.539886		imaginary frequency

Table S3. Relative MM energies (ΔE_{MM}), absolute and relative SCF energies (E_{SCF} and ΔE_{SCF}) and free energies (G) for discarded conformers of compound (*S*,*R*')-**14b**, as obtained after DFT optimization at the B97D3/def2-TZVP/fit level (IEFPCM solvation model for methanol).

Conf. ID	ΔE_{MM} (kJ mol⁻¹)	E_{SCF} (Ha)	G (Ha)	ΔE_{SCF} (kJ mol⁻¹)	Notes
(*S*,*R*')-**14b.16**	11.6763	−1472.90516172	−1472.546287	8.4570	
(*S*,*R*')-**14b.21**	12.5884	−1472.90497074	−1472.545126	8.9584	
(*S*,*R*')-**14b.11**	9.4855	−1472.90489922	−1472.545896	9.1462	
(*S*,*R*')-**14b.27**	13.6386	−1472.90486902	−1472.545963	9.2255	
(*S*,*R*')-**14b.34**	14.2704	−1472.90429901	−1472.545510	10.7221	
(*S*,*R*')-**14b.03**	4.2564	−1472.90425745	−1472.546205	10.8312	
(*S*,*R*')-**14b.28**	13.6909	−1472.90396449	−1472.543497	11.6004	
(*S*,*R*')-**14b.29**	13.7306	−1472.90384508	−1472.545171	11.9139	
(*S*,*R*')-**14b.33**	14.2461	−1472.90383643	−1472.545895	11.9366	
(*S*,*R*')-**14b.04**	4.9267	−1472.90381261	−1472.545665	11.9991	
(*S*,*R*')-**14b.20**	12.5796	−1472.90375871	−1472.543665	12.1406	
(*S*,*R*')-**14b.36**	15.2222	−1472.90357475	−1472.545358	12.6236	
(*S*,*R*')-**14b.44**	16.5209	−1472.90338513	−1472.545227	13.1215	
(*S*,*R*')-**14b.39**	15.7402	−1472.90322669	−1472.544416	13.5374	
(*S*,*R*')-**14b.64**	19.5175	−1472.90316411	−1472.544649	13.7017	
(*S*,*R*')-**14b.57**	18.4431	−1472.90314199	−1472.544152	13.7598	
(*S*,*R*')-**14b.52**	18.3376	−1472.90310998	−1472.545756	13.8439	
(*S*,*R*')-**14b.19**	12.3411	−1472.90282159	−1472.543136	14.6010	
(*S*,*R*')-**14b.47**	16.5963	−1472.90272273	−1472.542734	14.8606	
(*S*,*R*')-**14b.53**	18.3473	−1472.90244710	−1472.541253	15.5843	
(*S*,*R*')-**14b.48**	16.6645	−1472.90239230	−1472.544086	15.7281	
(*S*,*R*')-**14b.30**	13.9461	−1472.90212563	−1472.544157	16.4283	
(*S*,*R*')-**14b.25**	13.0775	−1472.90208666	−1472.544113	16.5306	
(*S*,*R*')-**14b.37**	15.3226	−1472.90195144	−1472.543133	16.8856	
(*S*,*R*')-**14b.61**	18.8431	−1472.90184188	−1472.543986	17.1733	
(*S*,*R*')-**14b.24**	13.0691	−1472.90167531	−1472.543293	17.6106	
(*S*,*R*')-**14b.14**	11.3935	−1472.90161693	−1472.543370	17.7639	
(*S*,*R*')-**14b.26**	13.4633	−1472.90092807	−1472.543133	19.5725	
(*S*,*R*')-**14b.59**	18.5778	−1472.90092796	−1472.542466	19.5728	
(*S*,*R*')-**14b.69**	19.9066	−1472.90079796	−1472.542628	19.9141	
(*S*,*R*')-**14b.70**	19.9803	−1472.90073198	−1472.542953	20.0873	
(*S*,*R*')-**14b.54**	18.3774	−1472.90064496	−1472.542839	20.3158	
(*S*,*R*')-**14b.68**	19.8769	−1472.90063407	−1472.541278	20.3444	
(*S*,*R*')-**14b.43**	16.4812	−1472.90047873	−1472.542485	20.7522	
(*S*,*R*')-**14b.49**	17.6477	−1472.90046200	−1472.543304	20.7961	
(*S*,*R*')-**14b.65**	19.5640	−1472.90036986	−1472.541814	21.0380	
(*S*,*R*')-**14b.41**	15.9335	−1472.90029758	−1472.539742	21.2278	
(*S*,*R*')-**14b.56**	18.4088	−1472.90011737	−1472.541901	21.7010	
(*S*,*R*')-**14b.58**	18.5276	−1472.89977149	−1472.542797	22.6091	
(*S*,*R*')-**14b.67**	19.7786	−1472.89962817	−1472.541284	22.9854	
(*S*,*R*')-**14b.62**	18.9778	−1472.89850770	−1472.538292	25.9272	
(*S*,*R*')-**14b.22**	12.8771	−1472.89756852	−1472.541512	28.3930	
(*S*,*R*')-**14b.05**	5.4551	−1472.90651551	−1472.547318		clustered with (*S*,*R*')-**14b.01**
(*S*,*R*')-**14b.09**	8.3621	−1472.90601825	−1472.547888		clustered with (*S*,*R*')-**14b.02**
(*S*,*R*')-**14b.35**	14.5888	−1472.90489926	−1472.545887		clustered with (*S*,*R*')-**14b.11**
(*S*,*R*')-**14b.66**	19.6292	−1472.90429902	−1472.545521		clustered with (*S*,*R*')-**14b.34**
(*S*,*R*')-**14b.17**	11.9926	−1472.90425745	−1472.546201		clustered with (*S*,*R*')-**14b.03**
(*S*,*R*')-**14b.50**	17.8021	−1472.90383642	−1472.545897		clustered with (*S*,*R*')-**14b.33**
(*S*,*R*')-**14b.13**	10.0554	−1472.90381258	−1472.545674		clustered with (*S*,*R*')-**14b.04**
(*S*,*R*')-**14b.45**	16.5645	−1472.90322671	−1472.544414		clustered with (*S*,*R*')-**14b.39**
(*S*,*R*')-**14b.51**	17.8657	−1472.90322667	−1472.544414		clustered with (*S*,*R*')-**14b.39**
(*S*,*R*')-**14b.63**	19.2924	−1472.90208668	−1472.544117		clustered with (*S*,*R*')-**14b.25**
(*S*,*R*')-**14b.40**	15.8925	−1472.90167520	−1472.543260		clustered with (*S*,*R*')-**14b.24**
(*S*,*R*')-**14b.18**	12.0349	−1472.90161692	−1472.543367		clustered with (*S*,*R*')-**14b.14**
(*S*,*R*')-**14b.32**	14.2030	−1472.90161691	−1472.543374		clustered with (*S*,*R*')-**14b.14**
(*S*,*R*')-**14b.42**	16.2159	−1472.90146921	−1472.541981		imaginary frequency
(*S*,*R*')-**14b.31**	13.9511	−1472.90092807	−1472.543091		clustered with (*S*,*R*')-**14b.26**
(*S*,*R*')-**14b.55**	18.3970	−1472.90047869	−1472.542479		clustered with (*S*,*R*')-**14b.43**

Table S4. Rotational strengths in dipole length formalism (R_j), oscillator strengths (f_j) and excitation wavelengths (λ_j) for the first 40 electronic transitions of the conformers of compound (S,S^*)-**14b**, as obtained by TDDFT calculations at the PBE0-⅓/def2-TZVPD//B97D3/def2-TZVP/fit level (IEFPCM solvation model for methanol).

j	R_j, 10^{-40} erg cm^3; f_j (λ_j, nm)					
	(S,S^*)-**14b.01**	(S,S^*)-**14b.03**	(S,S^*)-**14b.06**	(S,S^*)-**14b.10**	(S,S^*)-**14b.11**	(S,S^*)-**14b.21**
1	−12.4013; 0.0003 (257.31)	1.8606; 0.0006 (258.62)	59.9295; 0.2299 (255.36)	2.7366; 0.0004 (258.77)	3.8948; 0.0004 (258.74)	13.1016; 0.0015 (260.29)
2	9.7172; 0.2469 (253.61)	9.0826; 0.2430 (254.13)	−39.6550; 0.0135 (254.83)	−13.7254; 0.1968 (254.24)	−13.5556; 0.1931 (252.77)	−35.6608; 0.1714 (256.48)
3	4.1932; 0.0710 (248.33)	−3.5890; 0.0593 (248.41)	−5.2429; 0.0443 (249.32)	−18.7246; 0.1333 (246.64)	−24.1284; 0.1447 (246.37)	30.1541; 0.0441 (248.57)
4	23.2690; 0.0042 (233.46)	19.3851; 0.0045 (232.90)	21.3798; 0.0052 (233.67)	34.8861; 0.0046 (233.76)	31.4343; 0.0043 (232.58)	6.0065; 0.0027 (237.23)
5	−59.0282; 0.0924 (226.66)	−9.9107; 0.0466 (227.72)	−33.4726; 0.0829 (226.75)	0.7240; 0.0224 (226.87)	1.6725; 0.0291 (227.04)	−146.3393; 0.0869 (232.32)
6	−3.8988; 0.0110 (222.03)	−75.3912; 0.0532 (224.39)	4.5214; 0.0215 (223.48)	−72.6587; 0.0524 (226.60)	−69.8389; 0.0496 (224.82)	10.5528; 0.0068 (224.02)
7	2.6108; 0.0855 (215.93)	18.8154; 0.0813 (216.53)	−6.1410; 0.0966 (216.12)	9.0266; 0.0799 (217.20)	16.0854; 0.0802 (216.47)	73.2698; 0.0711 (217.57)
8	88.2835; 0.0443 (208.07)	106.0025; 0.1248 (207.17)	99.8755; 0.0898 (207.98)	−14.4316; 0.0221 (206.77)	−21.2678; 0.0286 (207.37)	56.8742; 0.0844 (207.56)
9	22.1776; 0.2117 (205.95)	−21.7220; 0.1152 (206.18)	−14.2160; 0.1296 (206.76)	56.0640; 0.1965 (205.97)	84.1523; 0.2138 (205.42)	47.8880; 0.1083 (207.39)
10	−47.0550; 0.0546 (202.69)	−64.6084; 0.0540 (202.68)	−70.5895; 0.0611 (203.35)	−55.7640; 0.0291 (202.79)	−58.6654; 0.0356 (201.82)	−89.0228; 0.0941 (203.86)
11	−7.1709; 0.0035 (200.27)	9.7755; 0.0030 (202.38)	22.4073; 0.0146 (202.63)	8.9355; 0.0023 (202.43)	7.3117; 0.0012 (201.30)	−11.1592; 0.0026 (202.02)
12	−26.2895; 0.2046 (198.42)	−54.1960; 0.1955 (198.53)	−7.2622; 0.0045 (200.10)	−52.0247; 0.2048 (200.15)	−57.0220; 0.2259 (199.42)	−10.3644; 0.1279 (199.82)
13	−27.6086; 0.0151 (192.11)	−0.1665; 0.0318 (197.20)	−50.3878; 0.2351 (198.33)	−0.8010; 0.0137 (197.74)	0.0855; 0.0168 (196.99)	3.3474; 0.0199 (197.94)
14	21.0091; 0.0080 (191.55)	12.9413; 0.0174 (194.09)	0.1306; 0.0296 (195.72)	15.0266; 0.0420 (193.75)	12.8903; 0.0385 (193.63)	−42.6815; 0.0797 (197.76)
15	−20.5511; 0.1913 (190.90)	25.4354; 0.0695 (190.81)	13.6057; 0.0425 (191.27)	6.3998; 0.0360 (191.30)	3.0325; 0.0236 (190.83)	2.2587; 0.0054 (196.69)
16	24.3306; 0.0233 (187.98)	−30.4540; 0.0230 (189.69)	5.6863; 0.0177 (189.22)	−9.7325; 0.0071 (190.39)	−23.8984; 0.0375 (190.15)	−1.1978; 0.0083 (193.62)
17	0.7672; 0.0033 (186.80)	−16.8160; 0.0500 (188.84)	−36.5711; 0.0656 (188.93)	−52.7811; 0.0643 (189.48)	−15.7778; 0.0560 (189.59)	−19.7055; 0.0116 (192.69)
18	3.9383; 0.0037 (186.42)	18.7166; 0.0105 (188.41)	12.1044; 0.0113 (188.14)	44.2970; 0.0578 (188.53)	43.5217; 0.0486 (187.87)	13.3974; 0.0219 (190.13)
19	0.0937; 0.0074 (185.03)	−0.9119; 0.0003 (186.08)	−1.9100; 0.0015 (186.41)	3.1524; 0.0018 (185.39)	3.7281; 0.0014 (184.80)	−0.9791; 0.0031 (188.10)
20	3.4397; 0.0008 (183.58)	17.0898; 0.0156 (184.73)	2.7718; 0.0089 (185.77)	−3.5818; 0.0159 (184.71)	5.3178; 0.0063 (184.20)	28.1565; 0.2190 (187.33)
21	22.2256; 0.0946 (182.85)	−27.8903; 0.0231 (184.44)	−0.6750; 0.0030 (185.39)	2.5086; 0.0434 (184.04)	−18.4058; 0.0209 (183.91)	−0.0971; 0.0038 (184.22)
22	2.8031; 0.0079 (182.40)	33.1851; 0.0526 (183.59)	3.4500; 0.0103 (185.30)	49.4532; 0.0787 (183.85)	26.8830; 0.0212 (183.53)	4.5261; 0.0060 (183.99)
23	−2.8374; 0.0129 (182.07)	22.1592; 0.0343 (182.67)	16.6253; 0.0563 (183.31)	16.1529; 0.0093 (182.68)	58.0911; 0.0715 (182.89)	−0.7960; 0.0056 (182.73)
24	2.9074; 0.0048 (180.83)	−10.2243; 0.0247 (181.82)	1.2909; 0.0724 (182.39)	2.1561; 0.0199 (182.07)	−7.1169; 0.0203 (181.98)	−17.9354; 0.0188 (181.42)
25	−3.2137; 0.0029 (180.54)	−11.0956; 0.0449 (181.05)	−13.0119; 0.0023 (180.95)	23.5008; 0.0154 (181.47)	−6.9721; 0.0013 (181.35)	9.2189; 0.0224 (180.06)
26	14.8790; 0.0558 (179.71)	36.0702; 0.0076 (180.41)	61.6728; 0.0703 (180.45)	−66.1979; 0.0435 (181.20)	−34.0216; 0.0553 (180.84)	−59.5552; 0.0514 (179.84)
27	12.9130; 0.0225 (179.19)	5.5088; 0.0444 (179.75)	−0.1493; 0.0413 (180.02)	0.1140; 0.0178 (179.92)	−3.7485; 0.0182 (179.40)	19.9701; 0.0259 (179.26)
28	−34.5421; 0.0342 (178.45)	−1.8566; 0.0325 (179.09)	−3.6752; 0.0044 (179.26)	−4.2660; 0.0086 (179.61)	−3.4215; 0.0084 (178.88)	13.1243; 0.0063 (178.31)
29	13.7523; 0.0443 (178.12)	−18.8537; 0.0274 (178.47)	−35.9615; 0.0481 (178.56)	−2.8537; 0.0142 (178.95)	5.5884; 0.0041 (178.53)	−16.3375; 0.0190 (178.22)
30	−0.7115; 0.0007 (177.65)	−16.8733; 0.0065 (177.67)	−4.1071; 0.0024 (176.13)	−16.0008; 0.0112 (178.48)	−23.4277; 0.0261 (177.89)	30.6384; 0.0288 (178.09)
31	−12.0319; 0.0069 (176.23)	−12.1475; 0.0043 (176.98)	1.7421; 0.0094 (175.86)	−18.5105; 0.0141 (176.87)	3.6744; 0.0088 (176.68)	1.7347; 0.0029 (177.31)
32	12.0450; 0.0121 (175.54)	11.9058; 0.0145 (176.48)	6.3937; 0.0054 (174.72)	8.3980; 0.0122 (176.73)	3.7019; 0.0090 (176.46)	3.6382; 0.0069 (177.24)
33	−41.2756; 0.0543 (175.01)	−8.7528; 0.0143 (176.12)	−17.6793; 0.0179 (174.61)	15.3874; 0.0093 (176.25)	−3.2273; 0.0114 (176.03)	5.7468; 0.0067 (176.20)
34	3.6934; 0.0117 (174.53)	32.9255; 0.0172 (174.97)	13.9946; 0.0574 (173.88)	1.8805; 0.0071 (175.93)	19.5534; 0.0258 (175.84)	47.2615; 0.0471 (175.09)
35	48.4909; 0.0278 (174.29)	7.4205; 0.0090 (174.74)	16.1488; 0.0182 (173.31)	4.6322; 0.0032 (175.55)	24.2457; 0.0135 (174.89)	−24.1052; 0.1142 (173.76)
36	1.6952; 0.0064 (173.05)	−12.4574; 0.0508 (174.33)	5.5392; 0.0141 (173.10)	19.4027; 0.0752 (174.53)	−8.8467; 0.0704 (174.35)	6.6980; 0.0027 (173.61)
37	−24.8392; 0.0493 (171.75)	−0.0727; 0.0067 (172.75)	8.1470; 0.0032 (172.74)	−15.3836; 0.0171 (172.04)	−21.5966; 0.0159 (172.17)	24.6692; 0.0250 (172.63)

38	−5.1534; 0.0013 (171.41)	−0.5495; 0.0350 (171.62)	−28.9884; 0.0288 (172.18)	−18.3544; 0.0286 (171.22)	−3.7062; 0.0197 (171.45)	32.8170; 0.0117 (172.58)
39	3.1399; 0.0108 (170.42)	11.5339; 0.0094 (171.05)	2.1824; 0.0569 (171.15)	4.5381; 0.0172 (171.10)	26.1453; 0.0579 (170.38)	1.0301; 0.0035 (172.11)
40	−3.8220; 0.0027 (170.14)	25.6526; 0.0233 (170.32)	6.2441; 0.0022 (170.00)	25.5650; 0.0461 (170.30)	5.2953; 0.0093 (170.08)	40.9297; 0.0326 (171.14)

Table S4. (*continued*)

j	R_j, 10^{-40} erg cm^3; f_j (λ_j, nm)				
	(*S*,*S*')-14b.29	(*S*,*S*')-14b.33	(*S*,*S*')-14b.42	(*S*,*S*')-14b.49	(*S*,*S*')-14b.54
1	19.4274; 0.0017 (260.02)	4.6058; 0.0003 (258.90)	14.0849; 0.0016 (260.35)	16.2313; 0.0012 (259.96)	38.3921; 0.1782 (257.35)
2	−12.8670; 0.1566 (258.24)	−16.2105; 0.1579 (252.05)	−31.8486; 0.1567 (256.64)	−10.4573; 0.1471 (257.14)	−48.1087; 0.0043 (255.69)
3	−39.5118; 0.1423 (247.52)	−48.4273; 0.2033 (242.91)	−48.6997; 0.1016 (247.74)	−38.7930; 0.1615 (245.61)	−36.3201; 0.1117 (248.54)
4	−5.0986; 0.0051 (235.64)	38.8398; 0.0053 (231.69)	1.4682; 0.0106 (233.65)	−6.4335; 0.0050 (230.48)	19.4874; 0.0037 (230.44)
5	−15.2124; 0.0018 (227.74)	−43.8843; 0.0314 (226.93)	−2.4857; 0.0013 (229.38)	9.5769; 0.0017 (228.98)	2.6141; 0.0012 (228.99)
6	−21.7269; 0.0591 (227.06)	12.4229; 0.0168 (224.64)	−31.4731; 0.0545 (225.02)	−35.0599; 0.0424 (224.79)	−33.9710; 0.0703 (225.03)
7	34.6121; 0.0864 (216.88)	−3.0311; 0.0812 (216.71)	53.9753; 0.0780 (217.37)	27.4748; 0.0853 (216.78)	24.8939; 0.0702 (216.38)
8	10.9140; 0.0018 (208.65)	−30.7761; 0.0331 (206.44)	94.3494; 0.1940 (207.21)	78.7321; 0.0484 (206.83)	160.4034; 0.1747 (207.38)
9	98.4196; 0.1438 (206.27)	40.3904; 0.2256 (204.99)	22.4108; 0.0181 (206.14)	27.0638; 0.1504 (205.96)	−6.9502; 0.0296 (207.35)
10	−81.1891; 0.1455 (203.58)	−20.7463; 0.0606 (201.00)	−1.9899; 0.0018 (203.33)	−22.7930; 0.0146 (202.95)	−3.7807; 0.0015 (204.19)
11	38.2097; 0.0440 (200.84)	−9.9416; 0.0131 (200.27)	−94.0727; 0.1284 (202.29)	−50.1559; 0.1746 (202.30)	−64.5540; 0.1441 (202.65)
12	−85.9285; 0.1351 (200.62)	−39.9464; 0.1606 (200.05)	−42.4890; 0.0984 (198.43)	−53.5294; 0.1093 (198.62)	−9.9754; 0.0074 (200.65)
13	−1.5322; 0.0015 (197.07)	3.5509; 0.0149 (197.09)	10.9980; 0.0081 (197.40)	6.8974; 0.0017 (197.09)	−43.0989; 0.0985 (198.44)
14	10.9155; 0.0141 (196.35)	6.5609; 0.0568 (193.04)	−8.3089; 0.0249 (195.71)	−0.2572; 0.0036 (193.56)	1.8499; 0.0119 (194.57)
15	−25.6282; 0.0371 (196.04)	−2.3686; 0.0028 (191.08)	1.1171; 0.0027 (194.42)	4.7547; 0.0077 (192.69)	−20.3455; 0.1326 (192.19)
16	−6.5274; 0.0137 (195.75)	−4.7171; 0.0154 (190.98)	−13.0522; 0.1194 (192.03)	−12.7636; 0.1016 (191.31)	4.6916; 0.0313 (190.36)
17	8.0819; 0.0494 (192.70)	−37.9030; 0.0875 (189.78)	0.7403; 0.0107 (190.08)	−8.5147; 0.0213 (189.99)	−1.1279; 0.0157 (189.53)
18	−6.2984; 0.0733 (189.66)	48.2092; 0.0578 (187.42)	−4.6938; 0.0102 (189.73)	9.2240; 0.0080 (189.55)	−2.6517; 0.0040 (189.28)
19	0.1334; 0.0016 (186.92)	1.1066; 0.0026 (184.21)	8.4109; 0.0333 (188.44)	−1.7688; 0.0004 (188.77)	0.8671; 0.0022 (187.67)
20	44.6486; 0.2045 (185.82)	0.9364; 0.0007 (183.48)	−4.5371; 0.0097 (186.77)	18.9957; 0.1857 (185.37)	−8.0989; 0.0173 (187.61)
21	4.2694; 0.0099 (185.06)	3.8882; 0.0144 (183.28)	1.0634; 0.1346 (185.00)	−16.5070; 0.0408 (185.17)	5.5161; 0.1497 (185.27)
22	2.3217; 0.0062 (184.22)	−7.9742; 0.0155 (183.07)	4.0660; 0.0032 (183.64)	6.0245; 0.0036 (184.05)	3.1058; 0.0043 (184.37)
23	−0.8206; 0.0009 (183.02)	76.0329; 0.1059 (182.37)	1.3099; 0.0164 (183.09)	3.6524; 0.0061 (182.76)	−6.9888; 0.0874 (182.89)
24	15.6846; 0.0172 (181.02)	24.1568; 0.0090 (181.63)	−7.0935; 0.0690 (182.36)	−0.4081; 0.0568 (182.31)	0.9552; 0.0105 (182.62)
25	−5.1646; 0.0012 (180.34)	−25.5953; 0.0275 (181.52)	−0.3128; 0.0031 (180.16)	9.6300; 0.0013 (179.70)	15.6361; 0.0140 (180.69)
26	15.5655; 0.0252 (179.78)	−30.0497; 0.0397 (180.54)	33.4969; 0.0095 (179.18)	1.8076; 0.0006 (179.14)	17.5111; 0.0040 (179.73)
27	0.8642; 0.0043 (179.48)	−3.3924; 0.0012 (179.66)	−1.9773; 0.0051 (178.64)	−6.0237; 0.0314 (178.65)	4.6653; 0.0331 (178.85)
28	−5.3342; 0.0304 (179.02)	−0.2631; 0.0134 (179.32)	−17.4896; 0.0318 (178.10)	1.3175; 0.0034 (178.31)	−18.7642; 0.0093 (178.65)
29	−0.0242; 0.0019 (178.36)	−5.4685; 0.0025 (178.20)	17.2043; 0.0374 (177.92)	−0.3810; 0.0231 (177.90)	1.3195; 0.0182 (178.27)
30	−24.6894; 0.0205 (178.01)	−7.5075; 0.0044 (177.76)	6.1908; 0.0038 (176.80)	−3.3687; 0.0052 (176.28)	7.3723; 0.0072 (176.47)
31	−1.3045; 0.0025 (177.71)	0.6092; 0.0081 (176.65)	15.8333; 0.0074 (176.57)	−9.0068; 0.0284 (175.72)	−10.0786; 0.0364 (175.81)
32	1.9714; 0.0008 (176.79)	−1.3318; 0.0040 (176.22)	−30.4970; 0.0322 (175.69)	−18.5415; 0.0066 (175.00)	−3.4451; 0.0056 (175.41)
33	−9.0190; 0.0151 (176.74)	−9.0864; 0.0009 (175.96)	1.7058; 0.0021 (175.53)	−20.2455; 0.0080 (174.86)	−20.3479; 0.0083 (175.17)
34	54.2379; 0.0174 (174.56)	38.2223; 0.0170 (175.63)	−16.4865; 0.0106 (174.82)	−44.9110; 0.0374 (174.42)	−11.0104; 0.0051 (174.99)
35	−108.8397; 0.1288 (173.89)	19.0713; 0.0653 (174.76)	−7.8670; 0.0161 (173.85)	−5.5101; 0.0011 (174.27)	4.2961; 0.0083 (173.94)

**36**	0.3272; 0.0032 (173.18)	−1.2503; 0.0301 (174.39)	−43.6747; 0.1081 (173.73)	27.0512; 0.0776 (173.95)	37.6234; 0.0318 (173.09)
**37**	8.6480; 0.0189 (172.66)	−74.8554; 0.0589 (171.04)	7.8593; 0.0036 (171.82)	−20.3913; 0.0167 (173.51)	−25.6508; 0.0691 (172.19)
**38**	−4.5184; 0.0069 (172.02)	−4.5926; 0.0135 (170.60)	−6.0461; 0.0053 (171.46)	5.8589; 0.0128 (171.18)	−7.4254; 0.0108 (171.72)
**39**	−8.8179; 0.0213 (171.04)	15.4257; 0.0333 (170.51)	26.3461; 0.0144 (171.24)	4.5448; 0.0050 (169.82)	−2.0960; 0.0270 (171.36)
**40**	23.0051; 0.0098 (170.06)	45.2859; 0.0154 (170.14)	−3.0815; 0.0061 (170.83)	−1.3934; 0.0039 (169.41)	8.8093; 0.0056 (170.96)

Table S5. Rotational strengths in dipole length formalism (R_j), oscillator strengths (f_j) and excitation wavelengths (λ_j) for the first 40 electronic transitions of the conformers of compound (*S*,*R*′)-**14b**, as obtained by TDDFT calculations at the PBE0-⅓/def2-TZVPD//B97D3/def2-TZVP/fit level (IEFPCM solvation model for methanol).

j	R_j, 10^{-40} erg cm^3; f_j (λ_j, nm)					
	(*S*,*R*′)-**14b.01**	(*S*,*R*′)-**14b.02**	(*S*,*R*′)-**14b.06**	(*S*,*R*′)-**14b.07**	(*S*,*R*′)-**14b.08**	(*S*,*R*′)-**14b.10**
1	−42.7340; 0.0026 (256.44)	−75.9943; 0.0081 (256.69)	−200.8541; 0.2051 (258.97)	−1.2899; 0.0139 (257.33)	−191.8532; 0.2190 (259.04)	−24.9373; 0.0009 (256.99)
2	0.5977; 0.2727 (255.66)	30.1593; 0.2442 (256.27)	173.8950; 0.0625 (257.92)	−12.3556; 0.2272 (255.20)	157.0913; 0.0484 (257.76)	−11.4995; 0.2031 (254.32)
3	9.5421; 0.0429 (249.00)	11.6039; 0.0772 (248.76)	2.7927; 0.0274 (250.85)	11.8865; 0.0425 (249.14)	10.1487; 0.0449 (250.56)	30.8862; 0.1520 (245.07)
4	−42.3130; 0.0125 (236.54)	−42.9449; 0.0107 (236.98)	0.1107; 0.0027 (242.98)	−18.7531; 0.0046 (235.03)	−0.3863; 0.0067 (242.10)	−51.7981; 0.0136 (235.90)
5	20.8799; 0.0244 (229.66)	19.6143; 0.0156 (230.30)	−35.8801; 0.0169 (235.40)	−4.3407; 0.0095 (233.79)	−39.7605; 0.0117 (236.37)	10.4035; 0.0045 (229.78)
6	27.2469; 0.0579 (224.74)	27.9179; 0.0533 (225.44)	15.8314; 0.1037 (229.22)	52.4137; 0.0754 (225.52)	12.4100; 0.0833 (228.84)	10.3105; 0.0336 (224.30)
7	33.3725; 0.0866 (216.73)	34.1271; 0.0838 (216.94)	33.9541; 0.1008 (218.22)	1.0668; 0.0753 (217.15)	33.0970; 0.0964 (218.28)	31.8646; 0.0807 (216.88)
8	−91.2732; 0.0686 (208.41)	−104.6653; 0.1338 (207.75)	−7.7706; 0.0023 (210.71)	−88.2388; 0.0467 (208.04)	−2.2547; 0.0006 (209.45)	−19.9206; 0.0064 (207.44)
9	−37.8359; 0.1717 (207.27)	5.6983; 0.0943 (207.31)	−121.8027; 0.2093 (207.95)	−22.7235; 0.2025 (207.12)	−104.9327; 0.1765 (208.12)	−27.2078; 0.2345 (206.38)
10	−20.3420; 0.0074 (203.84)	−20.4794; 0.0084 (204.37)	76.9021; 0.0942 (204.32)	38.3418; 0.0355 (203.29)	88.7350; 0.0909 (205.47)	−19.9954; 0.0090 (204.06)
11	77.0645; 0.0763 (202.79)	76.7547; 0.0556 (203.27)	−3.3604; 0.0210 (200.97)	−23.1503; 0.0081 (201.59)	−1.2919; 0.0504 (202.03)	63.7792; 0.1155 (201.81)
12	60.8585; 0.1373 (199.17)	57.5842; 0.1397 (200.56)	28.8273; 0.0272 (199.90)	3.4908; 0.0012 (200.10)	42.5174; 0.0379 (200.69)	38.2864; 0.0618 (200.63)
13	6.6576; 0.1006 (193.03)	−14.2130; 0.1684 (193.22)	21.5263; 0.0523 (199.12)	67.1326; 0.2061 (198.40)	3.7100; 0.0351 (199.20)	−4.2846; 0.0900 (192.95)
14	−37.2590; 0.0901 (191.44)	−24.3396; 0.0330 (191.80)	−0.2022; 0.0592 (197.31)	11.0132; 0.0148 (195.16)	0.8916; 0.0400 (196.44)	5.4869; 0.0316 (192.31)
15	−0.8128; 0.0092 (190.21)	−0.6603; 0.0107 (191.04)	−19.7642; 0.0818 (194.38)	0.6982; 0.0017 (194.33)	−29.8369; 0.1123 (195.17)	−34.6029; 0.1018 (190.98)
16	9.8042; 0.0103 (189.09)	16.7200; 0.0295 (189.25)	5.0845; 0.0880 (192.24)	6.9830; 0.0944 (192.02)	17.3643; 0.0602 (191.68)	32.4318; 0.0327 (190.07)
17	−20.1626; 0.0257 (188.18)	−3.5476; 0.0073 (188.29)	−0.4117; 0.0003 (191.71)	−31.1175; 0.0471 (189.24)	6.6527; 0.0416 (191.45)	−5.7977; 0.0062 (187.29)
18	25.3306; 0.0163 (187.22)	20.1246; 0.0165 (187.03)	29.1722; 0.0300 (190.41)	−4.4242; 0.0078 (188.48)	16.4872; 0.0190 (190.22)	11.1750; 0.0053 (186.20)
19	−1.0812; 0.0282 (186.06)	−7.1793; 0.0412 (186.27)	−20.3992; 0.0208 (186.41)	−8.7188; 0.0109 (188.18)	−27.1094; 0.0980 (186.46)	−6.3413; 0.0628 (185.43)
20	4.9587; 0.0065 (185.04)	4.3534; 0.0068 (185.25)	−49.4396; 0.1366 (185.40)	15.3482; 0.0086 (187.50)	−46.6118; 0.0700 (185.40)	15.9729; 0.0169 (184.37)
21	6.8206; 0.0363 (184.30)	18.1513; 0.0339 (184.39)	−1.6247; 0.0060 (184.96)	2.8845; 0.0096 (186.01)	−0.2612; 0.0090 (185.38)	−11.9275; 0.0131 (183.92)
22	0.6389; 0.0013 (183.98)	−18.4410; 0.0644 (184.24)	−1.5252; 0.0057 (183.12)	−2.4421; 0.0065 (184.68)	4.5305; 0.0022 (183.39)	14.1401; 0.0435 (183.64)
23	−39.0289; 0.0655 (183.58)	−39.0939; 0.0266 (182.69)	6.9150; 0.0043 (182.90)	0.7863; 0.1207 (182.10)	−2.6763; 0.0018 (182.35)	−47.1261; 0.0129 (182.14)
24	48.6584; 0.0348 (181.10)	−12.0462; 0.0024 (181.07)	3.5512; 0.0051 (181.94)	−0.3120; 0.0064 (181.19)	−27.3642; 0.0474 (182.15)	−3.2888; 0.0197 (181.73)
25	−46.4566; 0.0195 (180.92)	22.3823; 0.0472 (180.70)	−60.6170; 0.0463 (181.63)	−25.3178; 0.0033 (180.98)	−5.5063; 0.0036 (181.88)	9.5789; 0.0376 (180.20)
26	−14.7483; 0.0833 (180.42)	2.0361; 0.0240 (180.63)	2.9556; 0.0024 (181.28)	12.5562; 0.0742 (180.29)	1.6377; 0.0009 (181.37)	−9.5396; 0.0162 (179.97)
27	14.0394; 0.0259 (180.01)	14.4781; 0.0141 (180.24)	37.8840; 0.0157 (180.93)	−9.8767; 0.0113 (180.02)	32.2050; 0.0168 (181.12)	29.7441; 0.0104 (179.82)
28	17.5101; 0.0071 (179.95)	11.5079; 0.0457 (180.09)	72.1645; 0.0471 (179.22)	26.9308; 0.0341 (178.51)	44.5956; 0.0458 (179.58)	2.3749; 0.0018 (178.81)
29	2.5370; 0.0171 (178.27)	6.1439; 0.0150 (178.58)	1.3907; 0.0530 (179.04)	−6.3834; 0.0287 (178.08)	12.9997; 0.0054 (179.43)	−8.8309; 0.0167 (178.59)
30	17.1567; 0.0083 (176.78)	7.6800; 0.0059 (176.97)	1.3754; 0.0089 (178.80)	−6.1243; 0.0008 (177.11)	3.2537; 0.0127 (178.74)	7.6363; 0.0131 (177.13)
31	2.1699; 0.0068 (175.68)	6.5901; 0.0057 (176.43)	−5.4677; 0.0083 (178.36)	−6.5047; 0.0170 (176.85)	−8.9416; 0.0155 (177.72)	7.1040; 0.0046 (176.48)
32	−5.6049; 0.0069 (174.39)	−5.4014; 0.0029 (174.06)	−12.5148; 0.0179 (177.41)	6.7598; 0.0128 (175.50)	14.7485; 0.0203 (177.67)	26.1380; 0.0334 (173.73)
33	−14.3244; 0.0114 (174.15)	13.5577; 0.0133 (173.69)	9.3896; 0.0060 (175.89)	8.8326; 0.0064 (175.16)	4.9144; 0.0020 (176.12)	−6.4214; 0.0031 (173.55)
34	3.2909; 0.0006 (173.84)	−15.9317; 0.0053 (173.48)	−0.3069; 0.0050 (175.04)	44.5696; 0.0101 (174.14)	−5.7001; 0.0039 (175.77)	3.0540; 0.0016 (173.28)
35	18.7726; 0.0272 (173.16)	16.0007; 0.0310 (173.25)	−33.3468; 0.0275 (173.82)	−59.4302; 0.0711 (173.02)	−24.8829; 0.0169 (173.31)	−2.5752; 0.0017 (172.24)
36	25.6347; 0.0077 (171.63)	−2.0732; 0.0021 (172.63)	4.0976; 0.0053 (172.52)	−30.5610; 0.0301 (171.96)	20.8977; 0.0089 (172.72)	−44.3592; 0.0072 (171.93)
37	−10.3891; 0.0062 (171.55)	40.8789; 0.0081 (171.62)	28.0334; 0.0070 (171.94)	33.3962; 0.0523 (171.77)	10.8077; 0.0126 (171.64)	21.8356; 0.0399 (171.62)

38	−23.3252; 0.0622 (171.30)	−43.9387; 0.0804 (171.24)	11.4206; 0.0268 (171.27)	−15.0967; 0.0175 (171.17)	3.6076; 0.0710 (171.10)	54.3619; 0.1108 (171.13)
39	8.2300; 0.0036 (170.72)	27.7377; 0.0060 (170.75)	−21.5627; 0.0059 (170.68)	2.7791; 0.0018 (171.10)	−27.9805; 0.0610 (170.14)	−3.0461; 0.0010 (170.66)
40	6.6575; 0.0094 (170.23)	20.1066; 0.0061 (170.20)	−10.1895; 0.0280 (170.30)	−17.2195; 0.0124 (170.25)	8.5833; 0.0034 (169.86)	14.2586; 0.0221 (170.01)

Table S5. (*continued*)

j	R_j, 10^{-40} erg cm^3; f_j (λ_j, nm)					
	(*S*,*R*′)-14b.12	(*S*,*R*′)-14b.15	(*S*,*R*′)-14b.23	(*S*,*R*′)-14b.38	(*S*,*R*′)-14b.46	(*S*,*R*′)-14b.60
1	−3.2988; 0.0010 (260.42)	−121.5320; 0.0226 (257.76)	−0.6457; 0.0005 (258.99)	−1.7249; 0.0008 (260.59)	−0.8940; 0.0033 (256.51)	−57.8174; 0.0048 (257.45)
2	−37.0836; 0.2518 (255.66)	89.5885; 0.2499 (256.46)	−36.4710; 0.2185 (253.75)	−42.0927; 0.2199 (255.65)	−14.0086; 0.1618 (252.95)	21.3300; 0.2278 (255.03)
3	7.3899; 0.0409 (248.94)	17.2461; 0.0623 (249.54)	19.0678; 0.1130 (246.84)	10.5137; 0.0960 (247.82)	53.7854; 0.1716 (244.27)	28.0120; 0.1266 (247.37)
4	−23.9552; 0.0043 (234.31)	1.2042; 0.0029 (241.64)	−34.2670; 0.0049 (234.01)	−31.6593; 0.0045 (235.16)	−3.9632; 0.0024 (234.10)	3.0132; 0.0067 (238.44)
5	3.9585; 0.0069 (232.80)	−42.2354; 0.0127 (235.39)	6.5669; 0.0067 (230.39)	1.3297; 0.0021 (232.19)	−32.4904; 0.0060 (233.95)	−44.3758; 0.0135 (234.72)
6	45.5252; 0.0837 (225.74)	12.4031; 0.0811 (227.61)	34.8036; 0.0615 (225.19)	46.4931; 0.0711 (226.19)	18.7671; 0.0339 (224.82)	−1.1633; 0.0584 (226.33)
7	−3.3803; 0.0798 (216.63)	27.6002; 0.0991 (218.07)	−4.4767; 0.0794 (216.20)	3.9340; 0.0775 (216.90)	−2.9008; 0.0769 (216.42)	29.0359; 0.0959 (217.72)
8	−138.3973; 0.1295 (207.84)	14.9740; 0.0161 (210.77)	37.8712; 0.0273 (207.76)	52.4438; 0.0868 (207.34)	16.3215; 0.0115 (207.75)	36.6204; 0.0237 (209.29)
9	47.6514; 0.1231 (206.83)	−140.5106; 0.1537 (206.76)	−110.6287; 0.2182 (205.95)	−106.2542; 0.1309 (206.69)	−64.9481; 0.2493 (205.71)	−89.6364; 0.0539 (207.58)
10	−2.9776; 0.0013 (203.32)	86.9653; 0.1128 (204.20)	−26.4585; 0.0029 (203.20)	46.4504; 0.0248 (203.82)	1.7610; 0.0015 (201.55)	16.6262; 0.1879 (205.23)
11	19.3781; 0.0419 (202.77)	0.7930; 0.0578 (201.50)	36.4383; 0.0224 (202.37)	−23.4772; 0.0038 (203.43)	−15.5374; 0.0444 (201.12)	−4.0436; 0.0905 (201.99)
12	56.9018; 0.1829 (198.96)	39.8789; 0.0375 (200.31)	62.8909; 0.2158 (199.93)	50.0466; 0.2063 (200.56)	39.7493; 0.1385 (200.38)	50.3802; 0.0533 (200.83)
13	−1.7938; 0.0306 (195.54)	10.3114; 0.0485 (199.15)	−2.8361; 0.0204 (194.87)	0.4263; 0.0101 (194.99)	29.2143; 0.0464 (198.82)	0.5282; 0.0355 (198.05)
14	3.7463; 0.0032 (194.13)	7.6441; 0.0530 (196.31)	2.9966; 0.0014 (194.03)	0.9666; 0.0014 (193.90)	3.0510; 0.0006 (197.06)	−2.4163; 0.0901 (195.79)
15	0.9282; 0.0014 (193.28)	−32.6667; 0.1389 (194.05)	−0.1259; 0.0047 (193.06)	1.5413; 0.0048 (193.63)	17.4491; 0.0144 (196.01)	−15.2986; 0.0729 (193.79)
16	−1.5424; 0.0864 (191.57)	4.5043; 0.0040 (191.54)	3.9327; 0.1214 (191.13)	−1.8511; 0.1448 (190.97)	14.1855; 0.1024 (191.69)	26.2609; 0.0787 (191.01)
17	−36.9567; 0.0677 (189.27)	12.1824; 0.0270 (190.79)	−27.5774; 0.0235 (189.09)	14.8188; 0.0072 (189.91)	−11.7150; 0.0036 (188.56)	−1.9446; 0.0005 (190.58)
18	20.4589; 0.0052 (188.28)	16.4092; 0.0328 (189.65)	−0.4036; 0.0499 (187.97)	−6.9126; 0.0397 (188.33)	−1.5643; 0.0037 (188.29)	4.8521; 0.0203 (188.94)
19	−7.0476; 0.0156 (186.66)	−29.0132; 0.0829 (186.18)	−14.6128; 0.0176 (185.20)	−24.9495; 0.0184 (187.22)	−24.4241; 0.0551 (187.41)	−30.0029; 0.1090 (186.75)
20	1.5110; 0.0013 (186.50)	−55.5926; 0.0675 (185.98)	4.5168; 0.0018 (184.94)	−0.0606; 0.0010 (186.36)	14.7013; 0.0044 (186.69)	−30.1705; 0.0328 (184.38)
21	7.7303; 0.0462 (184.35)	2.3846; 0.0269 (184.36)	19.0183; 0.0050 (184.44)	10.2929; 0.0045 (185.11)	9.0433; 0.0266 (185.96)	0.9195; 0.0061 (183.57)
22	3.1973; 0.0437 (183.12)	4.0799; 0.0050 (183.23)	−10.2500; 0.0386 (183.12)	−11.1039; 0.0539 (183.55)	1.7532; 0.0051 (183.53)	5.5258; 0.0114 (182.53)
23	2.5027; 0.0428 (182.32)	21.0755; 0.0097 (182.64)	−55.9606; 0.0569 (182.04)	−28.4071; 0.0817 (182.51)	−18.7555; 0.0038 (182.24)	4.0057; 0.0193 (182.31)
24	4.4532; 0.0021 (180.89)	6.0722; 0.0059 (182.49)	54.6673; 0.0311 (181.47)	20.9070; 0.0059 (181.09)	−25.4017; 0.1314 (181.43)	0.2155; 0.0059 (182.05)
25	25.0848; 0.0141 (180.76)	−23.3645; 0.0367 (181.75)	−11.4095; 0.0063 (179.79)	1.8999; 0.0022 (180.38)	0.2899; 0.0012 (180.12)	−5.1859; 0.0120 (181.60)
26	−51.1207; 0.0786 (180.33)	20.4917; 0.0191 (181.51)	7.3790; 0.0053 (179.70)	16.0550; 0.0172 (180.29)	3.2466; 0.0175 (179.93)	30.1331; 0.0169 (181.45)
27	−14.1417; 0.0264 (179.97)	13.0235; 0.0150 (180.38)	0.8003; 0.0007 (179.38)	19.8509; 0.0578 (179.64)	12.0905; 0.0155 (179.57)	−2.6245; 0.0056 (180.86)
28	−0.9851; 0.0045 (179.66)	−3.9034; 0.0016 (179.26)	26.8882; 0.0437 (178.88)	−0.8036; 0.0322 (179.33)	4.9946; 0.0051 (177.97)	−6.4550; 0.0022 (179.27)
29	32.7731; 0.0385 (178.32)	47.4468; 0.0221 (178.76)	17.3458; 0.0676 (178.41)	−0.8919; 0.0005 (178.28)	2.1746; 0.0212 (177.87)	27.4604; 0.0149 (179.06)
30	1.7731; 0.0325 (177.31)	14.2899; 0.0215 (178.45)	−20.5748; 0.0261 (177.97)	−2.8753; 0.0283 (177.24)	1.1685; 0.0177 (176.64)	23.2712; 0.0065 (177.94)
31	−4.6118; 0.0045 (175.90)	2.9461; 0.0139 (178.16)	5.0909; 0.0078 (175.62)	5.7535; 0.0084 (176.13)	−20.2763; 0.0114 (176.54)	26.0952; 0.0148 (177.32)
32	−2.3226; 0.0017 (175.21)	−6.4250; 0.0141 (176.75)	−5.4431; 0.0021 (175.44)	−4.1459; 0.0025 (175.59)	15.2390; 0.0054 (174.82)	1.0872; 0.0028 (176.33)
33	24.5601; 0.0148 (174.56)	3.8628; 0.0044 (176.07)	10.8641; 0.0064 (174.74)	5.4699; 0.0016 (174.62)	1.3487; 0.0088 (174.56)	−19.7575; 0.0182 (175.31)
34	−36.7981; 0.0070 (173.08)	−3.1538; 0.0013 (175.29)	−21.5119; 0.0366 (172.90)	11.3911; 0.0055 (173.44)	13.1645; 0.0977 (173.26)	−1.5669; 0.0023 (174.41)
35	5.5088; 0.0020 (172.62)	−32.7430; 0.0200 (173.48)	0.3443; 0.0136 (172.05)	−30.5142; 0.0336 (173.14)	−7.6907; 0.0098 (172.10)	−41.2460; 0.0194 (173.30)

36	32.5131; 0.0474 (172.39)	25.0557; 0.0136 (172.43)	27.4059; 0.0325 (171.91)	−1.1226; 0.0020 (172.59)	−33.6515; 0.0146 (171.76)	68.7161; 0.0296 (172.49)
37	−5.6480; 0.0131 (172.02)	35.7454; 0.0162 (171.87)	5.2547; 0.0403 (171.64)	31.9938; 0.0079 (172.28)	8.3731; 0.0328 (171.21)	−3.1365; 0.0092 (171.50)
38	−6.3168; 0.0375 (171.38)	0.2106; 0.0786 (171.17)	1.2467; 0.0194 (170.86)	5.2681; 0.0847 (171.56)	5.3552; 0.0011 (171.15)	13.2549; 0.0134 (171.04)
39	0.5619; 0.0013 (170.93)	2.3423; 0.0132 (170.71)	8.8814; 0.0051 (170.70)	31.3379; 0.0169 (170.85)	78.6121; 0.0674 (170.72)	1.0433; 0.0069 (170.72)
40	−13.6487; 0.0125 (170.36)	−3.5761; 0.0127 (170.39)	1.1383; 0.0029 (170.34)	0.0181; 0.0007 (170.48)	−73.0884; 0.0534 (170.41)	−2.3985; 0.0258 (170.54)

References

1) Brem J, van Berkel SS, Zollman D, Lee SY, Gileadi O, McHugh PJ, Walsh TR, McDonough MA, Schofield, Structural Basis of Metallo-β-Lactamase Inhibition by Captopril Stereoisomers. *Antimicrob. Agents Chemother*. **2015**, 60, 142-50.
